# Polygenic Contribution to Sensorineural Hearing Loss Implicates Novel Risk Loci and Convergence with Congenital Hearing Loss Genes

**DOI:** 10.1007/s10162-026-01044-0

**Published:** 2026-03-16

**Authors:** Royce E. Clifford, Jacquelyn A. Johnson, Caroline E. Mackey, Elizabeth A. Mikita, Allen F. Ryan, Adam X. Maihofer, Caroline M. Nievergelt

**Affiliations:** 1 Research Service, Veterans Affairs San Diego Healthcare System, San Diego, CA USA; 2 Department of Otolaryngology – Head and Neck Surgery, University of California San Diego, La Jolla, CA USA; 3 Department of Psychiatry, University of California San Diego, La Jolla, CA USA

**Keywords:** Sensorineural hearing loss, Genome-wide association studies, Congenital hearing loss, Million Veteran Program

## Abstract

**Purpose:**

Disabling sensorineural hearing loss (SNHL) affects 5% of the global population. While congenital (CHL) and non-congenital sensorineural hearing loss (SNHL) are both strongly heritable, SNHL is a polygenic disorder, consisting of common genetic variants which individually confer small risks, requiring large studies for significance.

**Methods:**

We present the first report of a SNHL genome-wide association study (GWAS) from the Million Veteran Program (MVP) (210,240 cases and 265,275 controls), including multi-ancestry analysis, and then combine and contrast this data with a United Kingdom Biobank (UKB) self-reported hearing loss study (87,056 cases and 163,333 controls). We perform functional mapping and annotation, gene prioritization, gene-based and gene-set analysis, and cochlear cell type enrichment, including human single-cell expression data.

**Results:**

A total of 108 significant loci are identified, including 54 loci containing novel prioritized genes and/or protein-coding genes and implicating 17 known CHL genes. SNP-based partitioned heritability estimates show a 3.26-fold enrichment of CHL relative to other genes. Substantial genetic overlap is seen between MVP and UKB despite differences in phenotypes, demographics, and environmental exposures.

**Conclusion:**

In this multi-ancestry GWAS, we identify 108 loci with 54 novel genes. Despite the enrichment of CHL genes, 97% of the risk for adult-onset SNHL is captured by SNPs outside of monogenic hearing loss genes. Although SNHL in the UKB and MVP were assessed using different phenotypes, genetic signals between the two cohorts are predominantly shared, and locus discovery is boosted through increased sample size in meta-analysis.

**Supplementary Information:**

The online version contains supplementary material available at 10.1007/s10162-026-01044-0.

## Introduction

The effects of permanent sensorineural hearing loss (SNHL) range from annoyance at a partner’s “mumbling” to a disabling disorder that currently affects 1.6 billion people globally, a number that is predicted to increase by greater than 50% over the next three decades [1]. On a personal level, SNHL is associated with isolation, depression, cognitive decline, frailty, and dementia [2–4].

The most common causes of SNHL are aging and noise exposure [5]. Twin studies have calculated a heritability for SNHL of 0.35–0.65 [6, 7], indicating that a large portion is accounted for by genetic factors. Although early-onset hearing impairment has been linked to roughly 200 human congenital hearing loss (CHL) genes [8], in contrast, findings from genome-wide association studies (GWAS) on adult-onset SNHL demonstrate a highly polygenic genetic architecture [9, 10], characterized by common genetic variants individually conferring small risks.

Whereas the technology to cure hearing impairment caused by monogenic disorders utilizing gene therapy is rapidly advancing with some syndromes currently undergoing clinical trials [11], ultimate treatment of the more common polygenic forms of SNHL will require identification and characterization of multiple genes, variants, and pathways using technology yet to be determined. Given that statistical power to identify risk loci for polygenic disorders mandates large genetic studies, much of the otologic research has focused on the use of one or two self-reported items for disease identification [10, 12]. While the audiogram remains the gold standard for an objective measure of hearing loss, self-reported hearing loss questions have provided a satisfactory measure to elicit relevant loci from large genome-wide association studies (GWAS) [13–15]. The largest self-reported SNHL GWAS meta-analysis to date, including the UK Biobank (UKB) and 8 smaller cohorts of clinically diagnosed and self-reported hearing impairment on 723,266 individuals from the EARGEN consortium, identified 48 loci [16].

Here we report on a phenotype based on ICDs (international classification of disease) that include the most common forms of SNHL, i.e., age-related and noise-induced, typically ascertained by audiograms, from the Million Veteran Program (MVP), comparing the limitations of measuring a complex and clinically heterogeneous trait based on self-report to a clinical diagnosis of SNHL derived from more precise auditory measurements.

MVP consists of an aging population currently with over one million participants, who, during their military career, have been exposed to multiple sources of loud noise [17]. We contrast and compare two different phenotypes, the GWAS based on clinical diagnoses from MVP, with a GWAS based on self-reported hearing loss in the United Kingdom Biobank (UKB), which has been included in most of the recent GWAS meta-analyses [10, 12, 16, 18–22]. Our findings indicate that despite the differences in phenotype, demographics, and noise exposure of the two cohorts, they share a large proportion of their genetic architecture. Combination in meta-analysis elicits a total of 108 loci in individual ancestry GWAS and meta-analysis with 54 novel genes from prioritized analysis and 100 significant novel genes from gene-based examination. These loci contain inferences of 17 congenital hearing loss genes as well as multiple other ear-related genes, and we suggest a plausible explanation for a mechanism of injury.

## Methods

### SNHL Phenotype in MVP

MVP provided data from 963,753 participants (658,038 with genotype data) in version 23_1 (released Apr 29, 2024), recruited from 2011 through Sep 30, 2023, consisting of ICD records from the US Veterans Administration Corporate Data Warehouse [23]. Information was linked to individual, de-identified electronic health records (EHR).

Hearing loss was assessed using ICD9/ICD10 codes; participants with the following codes were excluded from analysis: otosclerosis (H80.0, H80.1, H80.2, H80.8, H80.9, 387.0, 389.1, 387.2, 387.8, and 387.9), Meniere’s disease (H81.0 and 386.0), pulsatile and objective tinnitus (H93.A, 388.32), conductive hearing loss (H90.0, H90.1, H90.2, H90.A1, 389.0), mixed hearing loss (H90.6, H90.7, H90.8, H90.A, H90.A3, 389.2), unilateral sensorineural hearing loss (H90.4, H90.A2, 389.13, 389.15, 389.17), ototoxicity (H91.0), sudden hearing loss (H91.2, 388.2), congenital malformations and anomalies (Q16, Q17.2, Q17.4, Q17.8, Q17.9, 744.0, 733.23, 744.29), intraoperative and postprocedural complications and disorders of ear and mastoid process, including mastoiditis and related conditions (H95, 383.3, 383.8, 383.89), certain degenerative and vascular disorders of the ear (H93.0, 388.0, 388.00, 388.02), disorders of the acoustic and cranial nerves (D33.3, H93.3, 225.1, 388.5), acoustic neuritis in infectious and parasitic diseases (H94.0), in addition to deaf non-speaking and other specified forms of hearing loss (H91.3, H91.8, H94.8, 389.7, 389.8).

From the remaining participants, SNHL cases (*N* = 236,597) had to have a diagnosis of sensorineural hearing loss (H90.3, H90.5, H91.1, H91.10, H91.11, H91.12, H91.13, 388.01, 389.1, 389.10, 389.11, 389.12, 389.14, 389.16, and 389.18), while controls (*N* = 296,284) consisted of those who had no evidence of the above ICDs nor ICDs for unspecified hearing loss (H91.9, H91.90. H91.91, H91.92, H91.93), or for the fitting, adjustment, presence, or management of a hearing aid or device (Z45.3, Z46.1, Z96.2, Z97.4, 389.9, and V53.2). All participants provided written informed consent, and the study was approved by the University of California San Diego and VA Central Institutional Review Boards.

### Genotyping, Quality Control, and Imputation

Analyses were based on release 4 of the MVP imputed genotype data containing 658,038 participants. Details of the genotyping, quality control, and imputation procedures used have been reported in detail [23]. In brief, MVP samples were genotyped on a customized version of the Affymetrix Axiom biobank array and standard genotype quality control procedures were followed. Genotype data was phased using Eagle version 2.4 [24] and imputed using minimac version 4 [25] with the Haplotype Reference Consortium [26] reference panel.

### Assessment of Ancestry

Ancestry was determined using SNPweights [27] with a global reference panel [28], including 93,172 SNPs that overlap between the reference data and MVP genotype data. Reference SNPs were selected to have MAF > 1% in reference populations and are in low LD (based on independent pairwise LD pruning, using a 1000-kb window, 50 SNP step size, and *r*-squared of 0.2, as calculated in PLINK [29]. Participants were placed into the three following large groupings: (1) European ancestry (EUA; individuals with ≥ 90% European ancestry); (2) African ancestry (AFA; individuals with ≥ 5% African ancestry, < 90% European ancestry, < 5% East Asian, Native American, Oceanian, and Central-South Asian ancestry, or individuals with ≥ 50% African ancestry, < 5% Native American, Oceanian, and < 1% Asian ancestry); and (3) Indigenous American Ancestry (IAA; individuals with ≥ 5% Native American ancestry, < 90% European, and < 5% African, East Asian, Oceanian, and Central-South Asian ancestry).

### Calculation of Relatedness and Principal Components (PCs)

Relatedness within samples was estimated using KING [30]. For each pair of related participants (kinship coefficient > 0.0884), one was removed, giving preference to retain cases. If case status was identical between the pair, one of the pair was removed at random (*N* = 21,923 excluded). PCs were calculated within unrelated subjects of the same ancestry using FlashPCA2 [31]. SNPs were excluded for MAF < 1%. Remaining SNPs were pruned for LD over 1-MB windows stepped over 50 variants at a time with an *r*^2^ threshold of 0.05.

### GWAS in MVP Based on EHR Data

GWAS for SNHL was performed for each of the 3 ancestry groups in MVP separately with PLINK 2 [29], using logistic regression including sex, age, age^2^, and 10 PCs as covariates. Only SNPs with MAF > 1% and imputation info score > 0.6 (calculated within analytic sample) were analyzed. Genome-wide significance (GWS) was assessed at a *p* value < 5 × 10^–8^.

### GWAS in UK Biobank (UKB) Based on Self-Reported Hearing Difficulty

GWAS summary results from Wells et al. [12] for self-reported hearing difficulty in the UKB were downloaded from the UKB Returned Datasets Catalog. The cohort consisted of white British ancestry (EUA only) recruited from 2007 to 2013 who volunteered to participate and completed a questionnaire including field code 2247 and 2257 regarding hearing difficulties and difficulties hearing in ambient noise. GWAS cases were those who answered “yes” to both questions (“Yes, diagnosed by doctor or health professional” or “Yes, not diagnosed by health professional”; *N* = 87,056 cases), controls were those who answered “no” to both (*N* = 163,333 controls). Association analysis was performed using a linear mixed-effects model implemented in BOLT-LMM v.228 including age, sex, genotyping platform, and 10 PCs (see [12] for additional details).

### Meta-analysis

For compatibility with MVP, UKB GWAS summary statistics were converted to the log odds ratio scale [32] and inverse variance weighted fixed effects meta-analysis was performed in METAL [33]. Only SNPs present in two or more input datasets were included in meta-analyses. Three meta-analyses were performed in total: (1) MVP multi-ancestry (EUA, AFA, IAA) meta-analysis; (2) European ancestry meta-analysis in MVP and UKB; (3) meta-analysis of the full sample, including MVP multi-ancestry + UKB EUA GWAS.

### Functional Mapping and Annotation

Functional annotation of the GWAS results was performed with FUMA v1.6.1 [34] using default settings and the human genome assembly GRCh37 (hg19). The SNP2Gene module was used to define independent genomic risk loci and variants in LD with lead SNPs (*r*^2^ > 0.6, calculated using ancestry-appropriate 1KGPp3 reference genotypes (EUR, AMR, AFR). Genomic risk loci were combined to have non-overlapping base pair positions, and SNPs were annotated to the nearest gene. Functional consequences of SNPs were obtained by mapping SNPs on their chromosomal position and reference alleles to databases containing known functional annotations, including ANNOVAR [35], Combined Annotation Dependent Depletion [36] and RegulomeDB [37].

### Gene Prioritization

Gene prioritization was performed using FLAMES version 1.1.2 [38]. To generate credible sets, Bayesian fine-mapping of risk loci was performed using the coloc R package [39]. Convergence-based gene prioritization scores were calculated using Polygenic Priority Scores v0.2 [40], using as inputs MAGMA gene-level *z*-scores and pathway naïve scores. FLAMES annotations were generated for each credible set, using functional annotation data as input, Polygenic Priority Score output data, and FUMA-generated MAGMA gene and gene-tissue analysis. Note, while FUMA positionally maps SNPs to genes based on their proximity to gene boundaries (within 10 kb), FLAMES annotates risk loci to a potentially larger set of genes because, in addition to positional mapping, it incorporates several other SNP-to-gene annotation databases. FLAMES scoring was performed using the annotation data. Only genes above the previously calibrated cumulative 75% precision threshold are reported.

### Hearing Genes from HereditaryHearingLoss.org

We extracted 196 protein-coding genes associated with non-syndromic and syndromic hearing loss from the Hereditary Hearing Loss database (https://hereditaryhearingloss.org, accessed 8/9/2024) [8]. This excluded Mitochondrial Non-syndromic Hearing Loss genes and *MIR96*, which encodes microRNA. Protein-coding genes were aligned with genes from GWS genomic risk loci and gene-based analysis (MAGMA). Genes with multiple symbol aliases were consolidated using the NIH gene database (https://www.ncbi.nlm.nih.gov/gene/; accessed 8/28/2024).

### Identification of Novel Genes

In order to identify novel, previously not reported genes, the following criteria were used.

For GWS loci from the GWAS: (1) The locus did not have any genes that were present in the HereditaryHearingLoss.org database (accessed 8/9/2024), (2) the locus did not have any genes that were present in the NHGRI-EBI GWAS Catalog database (https://www.ebi.ac.uk/gwas/home, accessed 7/2/2025; selected traits: sensory perception of sound, hearing loss, sensorineural hearing loss, hearing threshold trait, sensorineural hearing impairment, hearing physiology trait, noise-induced hearing loss, age-related hearing impairment, hearing process quality, able to hear with hearing aids, deafness, but excluding chemoradiation-induced hearing loss in nasopharyngeal carcinoma and speech-in-noise perception traits, as well as SNP-by-SNP and SNP-by-environment interaction studies and intergenic loci), (3) the locus does not overlap with a GWS locus from Wells 2019 [12] hearing difficulty (since this GWAS was used for the meta-analysis), (4) the locus did not overlap with something that was disqualified due to #1, 2, or 3, and (5) the locus had at least one protein-coding gene in the locus and/or a FLAMES prioritized gene.

For genes from the gene-based analysis: points 1–3 were applied.

### Regional Association Plots

Regional visualizations of GWS loci were produced using LocusZoom 1.4 [41] (Supplementary File 3). Linkage Disequilibrium (LD) was calculated using 1KGPp3 data, using ancestry-specific reference data (EUR, AMR, AFR).

### Gene-Based and Gene Set Analyses with MAGMA

The Multi-marker Analysis of GenoMic Annotation (MAGMA) v1.08 [42] tool implemented in FUMA was used to perform gene-based, gene-pathway, and human-based tissue enrichment analyses. For gene-based analysis, SNPs were mapped to 18,809 protein-coding genes. For each gene, its association with SNHL was determined as the weighted mean squared test statistic of SNPs mapped to the gene, where LD patterns were calculated using ancestry-appropriate 1KGPp3 reference genotypes. The significance of genes was set at a Bonferroni-corrected threshold of *p *< 2.658e−06 (0.05/18,809). To identify specific biological pathways implicated, gene-based test statistics were used to perform a competitive set-based analysis of 17,012 pre-defined curated gene sets and GO terms obtained from MsigDB [43]. The significance of pathways was set at a Bonferroni-corrected threshold of *p* < 2.94e−06 (0.05/17,012).

### Cochlear Cell Type Enrichment Analyses in Human

To test if tissue-specific gene expression was associated with SNHL, enrichment analyses were performed via MAGMA gene-tissue analysis of the EUA gene-based GWAS results. Since human inner ear cell type data has not yet been integrated in the FUMA tissue collection, we paired the collection with human adult inner ear tissue [44]. Data was converted from h5ad to h5seurat using SeuratDisk (0.0.0.9021). The R package Seurat v5.3.0 function AggregateExpression was used to log normalize and return pseudobulk gene expression across each cell type. MAGMA v1.10 was used to annotate the SNPs based on GRCh37 and perform gene-set analysis with GTEx v8 specific and general gene expression datasets downloaded from FUMA [34]. Gene-set analyses were conditioned on the mean of the normalized gene expressions (reads per kilo base per million, RPKM) of the GTEx v8 tissues.

### Cochlear Cell Type Enrichment Analyses in Mice

Cochlear cell type enrichment analyses were performed via MAGMA gene-tissue analysis of the EUA gene-based GWAS results paired with mice expression data. Ten-month-old mouse gene expression data was derived from cochlear cells and nuclei from Boussaty et al. [45]. Pseudobulk gene expression was generated across all 58 identified cell types using the same methods as described for human inner ear tissue. Mouse gene Ensembl IDs were converted to human Entrez IDs using a homology map from the Mouse Genome Database [46]. Only genes with one-to-one mappings were kept. MAGMA v1.10 was used to annotate the SNPs based on GRCh37 and perform gene-set analysis. Gene-set analyses were conditioned on the log2 mean expression across the cochlear cell types for each gene.

### SNP-Based Heritability and Genetic Correlation

SNP-based heritability (*h*^2^_SNP_) was evaluated in EUA data using LD score regression (LDSC) [47]. Input LD scores were computed from 1KGPp3 EUA samples. The LDSC intercept was used to test for artifactual inflation of test statistics, and the attenuation factor was used to estimate the proportion of inflation coming from polygenic signal. The *h*^2^_SNP_ was converted to the liability scale [48]. Cross-trait LDSC [47] was used to estimate the genetic correlation (*r*_g_) between datasets.

### Univariate and Bi-variate Gaussian Mixer Model (MiXeR) Analysis

We used univariate MiXeR v1.3 [49] to estimate the genetic architecture of MVP EUA and UKB SNHL phenotypes. MiXeR estimates SNP-based heritability and two subcomponents whose product is proportional to heritability: the proportion of non-null SNPs (polygenicity) and variance of effect sizes of non-null SNPs (discoverability). MiXeR was applied to GWAS summary statistics under the default settings with the supplied EUA LD reference panel. The results for the number of influential variants reflect the number of SNPs necessary to explain 90% of SNP-based heritability. Bivariate MiXeR [50] was used to estimate phenotype-specific polygenicity and the shared polygenicity between phenotypes. Goodness-of-fit of the MiXeR model relative to simpler models of polygenic overlap was assessed using AIC values. Heritability, polygenicity, and discoverability estimates were contrasted between datasets using the *z*-test.

### Polygenic Risk Scores (PRS)

SNHL PRS were calculated in EUA MVP samples based on the UKB SNHL GWAS and vice versa. GWAS summary statistics were filtered to common (MAF > 1%), well-imputed variants (INFO > 0.8). Indels and ambiguous SNPs were removed. PRS–continuous shrinkage [51] (PRS-CS) was used to infer posterior effect sizes of SNPs, using the 1000 Genomes Project Phase 3 EUR-based LD reference panel supplied with the program, with the global shrinkage parameter set to 0.01, 1000 MCMC iterations with 500 burn-in iterations and the Markov chain thinning factor set to 5. PRS were calculated using the --score option in PLINK 2, using the imputed genotype data of target samples, where for each SNP the risk score was estimated as the posterior effect size multiplied by the number of copies of the risk allele. PRS were estimated as the sum of risk scores over all SNPs. PRS were used to predict hearing status under logistic regression, adjusting for the same covariates as were used in GWAS. The proportion of variance explained by PRS for each study was estimated as the difference in Nagelkerke’s *R*^*2*^ between a model containing PRS plus covariates and a model with only covariates.

## Results

### Ancestry-Specific GWAS and Multi-ancestry Meta-analysis for SNHL in MVP

**Fig. 1:**
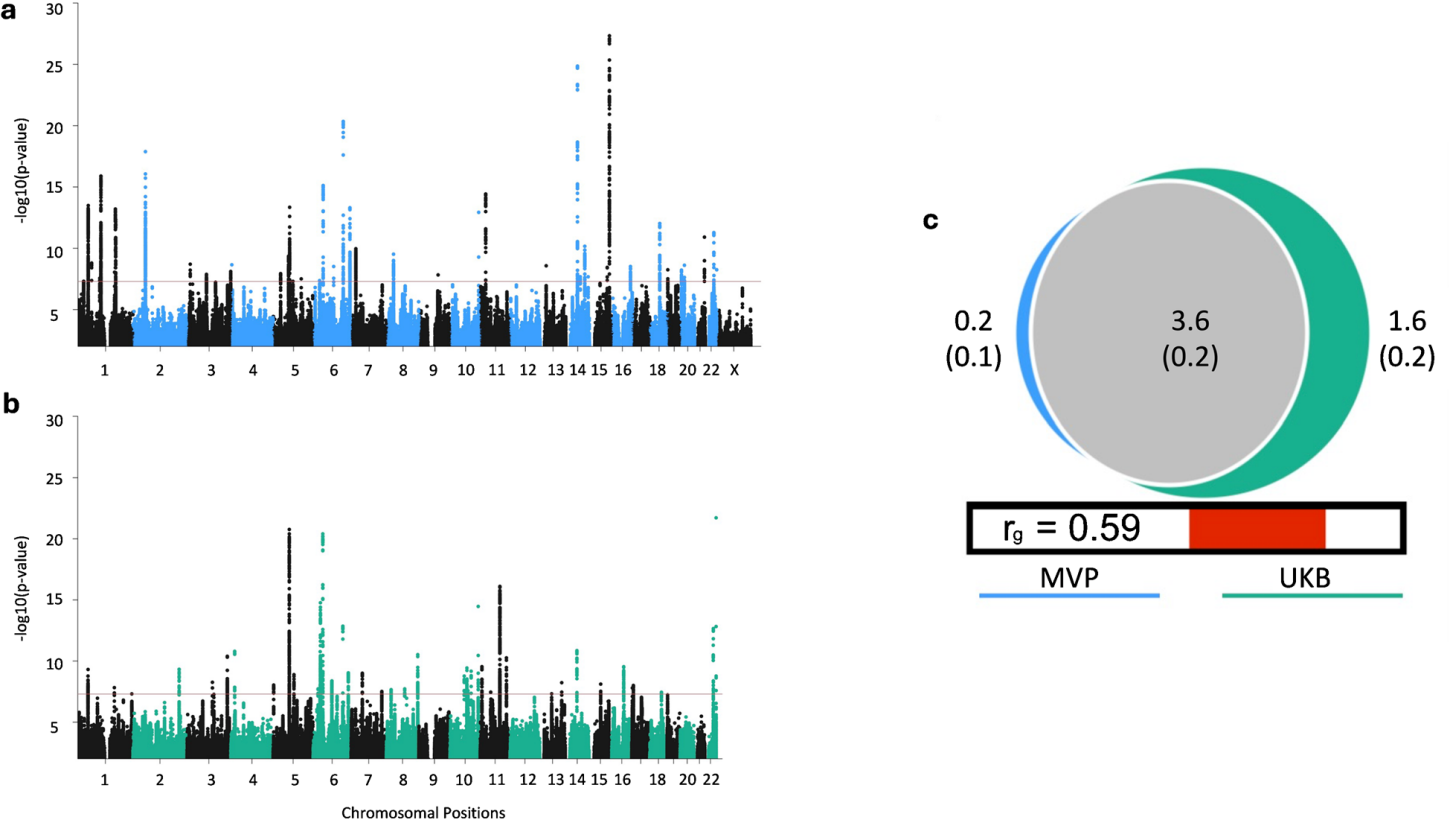
Comparison of SNHL between the clinically diagnosed MVP and self-reported UKB GWAS. **a**, **b** Manhattan plots of sensorineural hearing loss (SNHL) GWAS. The red line represents genome-wide significance (GWS) at p < 5 × 10^−8^. **a** MVP GWAS of clinically diagnosed SNHL in European ancestry participants (176,393 cases and 172,117 controls) identified 43 GWS loci. **b** UKB GWAS of self-reported SNHL (87,056 cases and 163,333 controls) identified 41 GWS loci. The X chromosome was not analyzed in the UKB GWAS. **c** Quantification of the polygenic overlap between SNHL in the MVP and UKB. Bivariate MiXeR modeling estimated 3624 causal variants are shared between cohorts (center, gray). There was only a small amount of polygenicity unique to MVP (left, blue), such that shared polygenicity accounted for 95.6% of the causal variants influencing MVP. The UKB had a larger unique polygenic component (right, green), such that shared polygenicity accounted for 69.2% of the causal variants influencing UKB. The numbers (in thousands, with standard errors in parenthesis) in the Venn diagram indicate the estimated quantity of causal variants per component. The size of the circles reflects the degree of polygenicity. Genetic correlation (*r*_g_) estimated between the two phenotypes is shown below the Venn diagram

GWAS in the MVP was performed based on ICD diagnosis of SNHL from the electronic health record. After excluding participants for mixed, conductive, and uncommon forms of SNHL (e.g., Meniere’s disease and ototoxicity), 210,240 cases and 265,275 controls were available for GWAS. The MVP cohort is predominantly (91%) male with an average age of 62.2 (13.9 SD) years at enrollment (Supplementary Table 1). GWAS were stratified into three ancestry groups. GWAS of European ancestry participants (MVP EUA; 176,393 cases and 172,117 controls) identified 43 genome-wide significant (GWS) loci (Supplementary Table 2, Fig. 1a). The inflation of test statistics (genomic control *λ* = 1.33) was almost entirely accounted for by polygenic effects (LDSC attenuation ratio: 14.7%; LDSC intercept = 1.06; SE = 0.009; Supplementary Table 7). Protein-coding genes within GWS loci were identified using functional annotation with FUMA and further prioritized using FLAMES, identifying 29 genes. GWAS of African ancestry participants (MVP AFA; 22,757 cases and 74,842 controls) identified one GWS locus (rs59174224, *p* = 4.12E−08) within the gene *TUB* (Fig. S1b). *Tub* is a known murine gene leading to profound hearing loss. A modifier *Moth1* protects hearing, and *Mtap1* then mediates *Moth1* at the synaptic terminal [52]. GWAS of MVP indigenous American ancestry participants (MVP IAA; 11,090 cases, 18,316 controls) did not identify any GWS loci (Fig. S1c). Meta-analysis of the EUA, AFA, and IAA GWAS (MVP meta-analysis; *N* = 475,515 subjects) identified 52 loci; 14 of which were not significant in individual ancestry analyses (Supplementary Table 2, Fig. S1d). Gene prioritization using FLAMES identified 36 genes in the 52 loci. Annotations for SNPs in LD with significant SNPs in the MVP multi-ancestry meta-analysis are summarized in Supplementary Table 3. Of the 52 loci, 13 contained a GWS SNP with a CADD score predicted to be deleterious to function (> 12.37) that was within the exon region of a gene. A total of 57 independent GWS loci were identified across the four MVP GWAS performed. Regional association plots comparing ancestry-specific analyses for these loci are shown in Supplementary Data 1.

### Gene-Based, Gene-Set, and Gene-Tissue Analyses in MVP

To aggregate genetic markers at the level of genes and allow integration with gene expression data, we performed gene-based analyses using MAGMA [42]. Gene-based analyses identified a total of 76 GWS genes across the EUA, AFA, IAA, and meta-analyses (Supplementary Table 4, Fig. S2 and S3a), several of which had not already been implicated by the positional mapping of SNPs to genes. To ascertain specific biological pathways, a competitive set-based analysis of 17,012 pre-defined curated gene sets and gene ontology (GO) terms obtained from MsigDB was performed in the MVP meta-analysis (Supplementary Table 5). Significant curated gene sets identified were in the gene ontology biological processes area, i.e., GOBP_EAR_DEVELOPMENT (*p* = 1.52E−07), GOBP_SENSORY_PERCEPTION_OF_MECHANICAL_STIMULUS (*p* = 3.19E−07), and NIKOLSKY_BREAST_CANCER_14Q22_AMPLICON (*p* = 2.50E−06). We note that the Nikolsky breast cancer amplicon pathway significance level was primarily driven by one locus with two mapped genes with SNPs in partial LD, *NID2* (*p*-value 2.61E−14) and *C14orf116* (*FOXN3*, *p* = 7.25E−10) (Supplementary Table 6).

### Genetic Comparisons of MVP and UKB SNHL GWAS

We performed a comparison of the genetic architecture of SNHL based on the GWAS using ICD codes in MVP with a similarly sized GWAS (cases: *N* = 87,056; controls: *N* = 163,333) based on self-report in the previously published UKB GWAS [12], which had identified 41 GWS loci (Fig. 1b). Only data of EUA individuals were included.

The *h*^2^_SNP_ estimated from the MVP was 0.0761 (SE = 0.0039, on the liability scale at 30% population prevalence) (Supplementary Table 7A). In contrast, *h*^2^_SNP_ estimated from the UKB was higher at 0.1162 (SE = 0.0059, liability scale at 30% population prevalence). Differences in genetic architecture were further explored with univariate MiXeR, noting that *h*^2^_SNP_ is proportional to the number of causal variants and the effect sizes of these variants. Univariate MiXeR analyses identified greater polygenicity within UKB (5240 causal variants, SE = 250) than in MVP (3790 causal variants, SE = 198) (*z* = 4.5, *p* = 5.4 × 10^–6^). However, variant effect sizes were not significantly different between studies (*z*-test comparing causal effect size variance estimates: *z* = 1.3, *p* = 0.18) (Fig. 1c and Supplementary Table 8a).

Shared and unshared genetic variation between studies was explored with bivariate MiXeR analyses. MiXeR estimated that MVP and UKB shared 3624 (SE = 204) causal variants, comprising 95.6% of the total MVP causal variants, but only 69.2% of the total UKB causal variants (Fig. 1c and Supplementary Table 8b). Correspondingly, MiXeR estimated that 1617 (SE = 235) variants were unique to the UKB, but only 167 (SE = 126) variants were unique to MVP.

Genetic correlation among shared variants was *r*_g_ = 0.73 (SE = 0.03), which was higher than the standard genome-wide estimate of genetic correlation *r*_g_ = 0.59 (SE = 0.03) estimated by LDSC (Supplementary Table 7B). A comparison of only GWS loci showed a correlation of *r* = 0.77 between effect sizes of leading variants, further establishing that the identified GWS loci have similar effects across studies. In conclusion, while self-reported hearing loss in the UKB is influenced by a broader set of variations compared to the clinically diagnosed SNHL in MVP, there is a substantial core set of variation with comparable effect sizes across these cohorts. Of note, however, our analyses are not able to distinguish between differences in populations or the assessment tools used.

### Meta-analysis of MVP and UKB EUA GWAS

An inverse variance weighted fixed effects meta-analysis in 598,899 EUA individuals (cases: *N* = 263,449; controls: *N* = 335,450) of the EUA MVP and UKB GWAS identified 99 GWS loci (Supplementary Table 9). Of these, 43 were not previously identified in either the MVP or UKB GWAS. These data will be used in downstream analyses that are based on linkage disequilibrium (LD) patterns, as many of the non-EUA individuals in the MVP are admixed.

### Multi-ancestry Meta-analysis of MVP and UKB

**Fig. 2:**
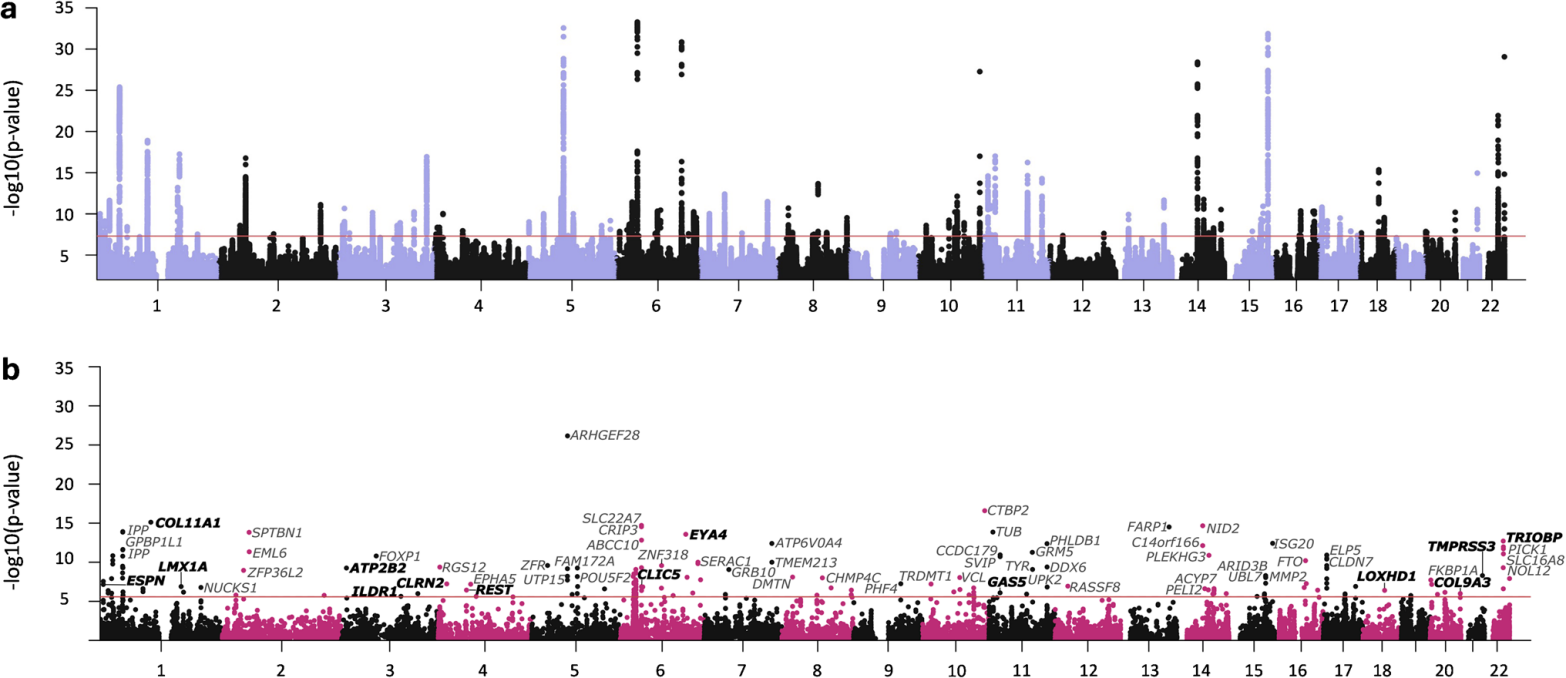
Risk loci (*N *= 108) and gene discovery (*N* = 192) for SNHL in a multi-ancestry meta-analysis. Manhattan plots of SNP (**a**) and gene-level (**b**) associations from the meta-analysis of the MVP multi-ancestry and UKB sensorineural hearing loss (SNHL) GWAS (297,296 cases and 428,608 controls). The red line represents genome-wide significance at *p* < 5 × 10^−8^ (**a**) and gene-wide significance *p* < 2.66 × 10^−6^ (**b**, Bonferroni correction for 18,809 genes tested), respectively. A selection of GWS genes is labeled, and genes listed in HereditaryHearingLoss.org are labeled in bold font

A meta-analysis of the MVP multi-ancestry GWAS and the UKB GWAS identified a total of 108 GWS loci in 725,904 individuals; 54 of these loci have a novel protein-coding gene and/or FLAMES prioritized gene within the locus (see definition of “novel” in methods), while two loci (locus 15 and 49) did not include a gene (Table 1, Supplementary Table 10 and Fig. 2b). Regional association plots comparing ancestry-specific analyses for these loci are shown in Supplementary Data 1. Functional mapping and annotation of risk loci using the FUMA pipeline are summarized in Supplementary Table 11. Of the 108 loci, 26 (24%) contained an exonic GWS SNP with a predicted deleterious CADD score (> 12.37).

**Table 1:** Genome-wide significant loci in meta-analysis of sensorineural hearing loss (*N* = 725,904 MVP multi-ancestry and UKB individuals)

Locus	Chr	Start^a^	End^a^	Lead SNP	A1	A2	A1 Freq	Beta	SE	*p* value^b^
1	1	6,355,065	6,505,159	rs112725535	A	G	0.18	−0.032	0.005	**9.86E−11**
2	1	16,367,202	16,424,085	rs2863455	T	C	0.68	−0.026	0.004	**7.48E−10**
3	1	25,580,095	25,863,694	rs2281179	T	C	0.54	−0.025	0.004	**2.33E−12**
4	1	45,768,792	46,644,026	rs201377643	C	G	0.32	0.042	0.004	**4.20E−26**
5	1	61,858,621	61,868,766	rs2499507	T	C	0.46	0.023	0.004	**3.71E−09**
7	1	102,902,818	103,757,081	rs12740738	A	G	0.45	−0.034	0.004	**1.27E−19**
8	1	165,086,665	165,126,202	rs7525101	T	C	0.42	0.028	0.004	**6.53E−14**
9	1	168,745,350	168,902,339	rs7555365	T	C	0.62	0.035	0.004	**5.66E−18**
10	1	170,058,829	170,196,129	rs501925	A	G	0.61	−0.025	0.004	**1.78E−11**
11	1	205,663,993	205,757,824	rs823116	A	G	0.52	−0.021	0.004	**2.94E−08**
13	2	43,450,843	43,602,415	rs67278917	CCA	C	0.76	−0.026	0.004	**2.56E−09**
14	2	54,687,254	55,062,865	rs2941580	A	G	0.47	−0.031	0.004	**1.72E−17**
15	2	112,046,160	112,069,485	rs2341098	T	G	0.44	−0.023	0.004	**2.74E−08**
16	2	208,000,822	208,118,374	rs2216374	T	C	0.40	0.026	0.004	**7.82E−12**
17	3	10,411,827	10,521,964	rs511273	T	C	0.34	0.025	0.004	**2.15E−09**
18	3	13,831,209	13,855,799	rs877306	T	C	0.40	−0.026	0.004	**2.21E−11**
19	3	24,267,011	24,267,842	rs6779690	T	C	0.81	−0.028	0.005	**2.13E−08**
20	3	71,353,372	71,628,286	rs75175086	T	C	0.77	−0.028	0.004	**7.02E−11**
23	3	121,351,315	121,729,262	rs3915060	T	C	0.72	−0.025	0.004	**2.59E−09**
24	3	127,710,474	128,095,278	rs4857868	A	T	0.84	−0.032	0.005	**1.21E−09**
25	3	156,243,368	156,303,912	rs9856073	T	G	0.80	0.030	0.005	**5.93E−11**
26	3	181,935,178	182,210,347	rs7649191	A	G	0.28	−0.036	0.004	**1.18E−17**
28	4	3,285,389	3,433,782	rs3129327	A	G	0.62	0.022	0.004	**2.86E−08**
30	4	5,860,906	5,919,670	rs6446406	T	C	0.36	0.022	0.004	**5.53E−09**
31	4	10,011,996	10,412,191	rs10489074	A	C	0.25	0.025	0.004	**4.34E−09**
32	4	17,517,558	17,530,692	rs13147559	C	G	0.87	−0.037	0.006	**9.45E−11**
33	4	57,709,860	57,903,482	rs13152711	T	C	0.27	−0.024	0.004	**1.13E−08**
34	5	2,549,211	2,564,723	rs7712395	T	C	0.12	0.034	0.006	**9.64E−10**
35	5	32,323,623	32,449,816	rs2963985	A	G	0.42	−0.024	0.004	**9.74E−11**
36	5	68,047,930	68,253,056	rs4246760	T	C	0.66	0.028	0.004	**1.16E−12**
37	5	72,823,715	73,199,539	rs6453022	A	C	0.52	0.044	0.004	**2.75E−33**
38	5	82,299,375	82,547,602	rs2386237	A	G	0.61	−0.020	0.004	**4.38E−08**
40	5	92,684,177	93,571,190	rs167570	T	C	0.13	−0.035	0.006	**9.83E−11**
42	5	150,939,707	150,966,388	rs9688110	A	G	0.31	0.024	0.004	**2.63E−09**
43	5	168,841,685	168,896,564	rs3074558	A	AAG	0.63	−0.024	0.004	**6.70E−10**
44	6	7,066,525	7,153,152	rs11243143	A	G	0.60	0.022	0.004	**1.39E−08**
45	6	21,960,455	21,970,417	rs1928176	A	G	0.53	−0.021	0.004	**8.99E−09**
46	6	26,354,100	29,607,101	rs66868086	A	T	0.90	0.038	0.006	**1.77E−09**
47	6	43,201,763	43,434,705	rs2242416	A	G	0.43	−0.045	0.004	**5.21E−34**
48	6	45,942,037	45,979,229	rs3777621	T	G	0.64	0.021	0.004	**4.91E−08**
49	6	80,052,589	80,087,011	rs2038297	T	C	0.81	0.026	0.005	**2.31E−08**
50	6	83,978,225	84,409,255	rs217308	T	C	0.58	−0.024	0.004	**4.72E−11**
51	6	90,645,615	90,673,538	rs9451298	T	C	0.72	0.027	0.004	**3.53E−11**
52	6	133,273,015	133,869,809	rs9493627	A	G	0.34	0.044	0.004	**1.38E−31**
53	6	147,946,333	147,986,716	rs9390488	T	C	0.59	−0.021	0.004	**2.62E−08**
54	6	158,497,717	158,619,409	rs3818457	T	C	0.48	−0.024	0.004	**5.69E−11**
55	6	163,789,736	164,005,951	rs1737332	T	C	0.74	−0.027	0.004	**2.95E−10**
56	7	19,548,842	19,654,201	rs10232942	A	G	0.39	−0.025	0.004	**9.44E−11**
57	7	50,734,630	50,882,081	rs1962390	C	G	0.23	−0.032	0.004	**3.99E−13**
58	7	86,198,818	86,340,497	rs274625	A	C	0.61	−0.021	0.004	**2.13E−08**
59	7	138,432,792	138,514,861	rs7780560	A	G	0.47	0.026	0.004	**3.28E−12**
61	8	21,904,891	21,944,508	rs113973451	T	G	0.21	−0.031	0.005	**2.04E−11**
62	8	30,271,637	30,518,967	rs3824107	A	G	0.23	−0.024	0.004	**1.81E−08**
63	8	71,467,435	72,012,927	rs62506938	T	G	0.92	−0.036	0.007	**3.62E−08**
64	8	74,206,582	74,254,612	rs7819550	A	G	0.19	0.026	0.005	**3.00E−08**
65	8	82,653,644	82,829,051	rs78724141	T	G	0.07	0.056	0.007	**2.21E−14**
66	8	99,925,359	100,804,670	rs11784152	A	G	0.25	−0.024	0.004	**1.84E−08**
67	8	141,604,684	142,018,581	rs12156228	T	G	0.60	−0.025	0.004	**2.99E−10**
68	9	84,736,303	85,036,608	rs1409880	A	G	0.62	0.022	0.004	**2.40E−08**
69	9	96,349,538	96,484,560	rs10821202	A	C	0.38	−0.021	0.004	**1.54E−08**
70	10	17,172,952	17,248,287	rs12355391	A	C	0.58	−0.023	0.004	**2.54E−09**
71	10	63,828,879	63,841,130	rs2393729	T	C	0.40	−0.023	0.004	**6.00E−10**
73	10	75,783,340	76,358,087	rs377150672	T	TA	0.60	−0.026	0.004	**5.92E−11**
74	10	80,273,287	80,610,121	rs149979867	A	G	0.20	−0.033	0.005	**7.27E−13**
75	10	82,379,843	82,409,617	rs71483314	A	G	0.02	0.082	0.015	**1.75E−08**
76	10	94,759,464	94,831,523	rs12771031	T	C	0.51	−0.023	0.004	**1.23E−09**
78	10	126,755,203	126,874,931	rs10901863	T	C	0.26	0.050	0.005	**5.45E−28**
79	11	8,053,304	8,085,652	rs4483583	A	C	0.80	0.036	0.005	**2.54E−15**
80	11	8,826,688	9,046,767	rs4910179	T	G	0.33	0.023	0.004	**6.30E−09**
81	11	13,178,285	13,197,316	rs10831990	T	C	0.74	0.030	0.004	**9.96E−12**
82	11	15,993,057	16,224,877	rs11023821	A	G	0.42	0.020	0.004	**3.59E−08**
83	11	22,797,251	22,880,918	rs11543287	A	G	0.39	−0.033	0.004	**9.83E−18**
85	11	88,358,544	89,204,911	rs1126809	A	G	0.28	0.035	0.004	**5.82E−17**
86	11	118,477,367	118,735,476	rs67307131	T	C	0.62	−0.030	0.004	**5.25E−15**
87	12	26,114,019	26,193,294	rs11048385	T	C	0.57	−0.021	0.004	**4.34E−08**
88	12	109,860,891	110,042,348	rs2111216	A	G	0.39	−0.021	0.004	**2.40E−08**
89	13	26,624,935	26,688,841	rs111516934	T	C	0.16	0.032	0.005	**1.19E−10**
91	13	99,050,232	99,075,551	rs7329659	A	G	0.67	−0.028	0.004	**2.17E−12**
92	14	52,453,659	52,542,480	rs1566129	T	C	0.42	0.041	0.004	**3.91E−29**
93	14	65,165,090	65,217,898	rs229650	T	C	0.75	−0.030	0.004	**1.78E−12**
94	14	73,961,821	74,018,555	rs35417585	A	G	0.76	0.025	0.005	**3.50E−08**
95	14	75,503,759	75,668,384	rs35499335	G	GT	0.58	0.022	0.004	**1.96E−08**
96	14	85,288,673	85,409,941	rs12588043	A	C	0.47	−0.022	0.004	**5.18E−09**
97	14	100,571,762	100,611,949	rs1951486	T	C	0.44	0.025	0.004	**2.99E−11**
99	15	57,132,863	57,586,614	rs59425097	CT	C	0.71	0.025	0.004	**1.32E−08**
100	15	74,713,300	75,029,407	rs11858525	T	G	0.84	0.038	0.006	**1.62E−09**
101	15	79,219,188	79,257,278	rs34593439	A	G	0.10	−0.044	0.006	**1.19E−11**
102	15	89,040,591	89,274,382	rs12594617	C	G	0.80	0.056	0.005	**1.36E−32**
103	16	51,163,406	51,189,169	rs11643654	A	C	0.57	−0.021	0.004	**3.89E−08**
104	16	53,797,908	53,845,487	rs62033400	A	G	0.62	0.025	0.004	**4.42E−11**
105	16	79,135,887	79,167,945	rs10492901	T	C	0.62	0.025	0.004	**4.91E−11**
106	16	81,587,072	81,616,668	rs59164848	T	TG	0.44	0.024	0.004	**4.76E−11**
107	17	2,473,537	2,612,584	rs734957	A	G	0.23	0.026	0.004	**2.44E−09**
108	17	6,193,984	8,130,452	rs34958987	T	TG	0.40	0.025	0.004	**1.53E−11**
109	17	17,628,678	18,140,308	rs117283876	A	G	0.02	0.081	0.013	**7.00E−10**
111	17	43,573,649	44,351,285	rs2458203	T	C	0.68	−0.027	0.004	**3.25E−10**
113	17	65,822,573	66,098,154	rs56305452	A	C	0.72	0.024	0.004	**1.22E−08**
114	17	79,585,385	79,689,749	rs58038388	T	C	0.03	0.085	0.016	**4.24E−08**
115	18	6,674,194	6,755,963	rs2228712	A	C	0.52	0.021	0.004	**2.02E−08**
116	18	41,994,736	42,298,323	rs2852779	T	G	0.84	−0.040	0.005	**4.52E−16**
117	18	52,573,169	52,669,732	rs11152090	A	C	0.79	−0.027	0.005	**5.30E−09**
118	18	53,210,302	53,466,338	rs1031829	A	G	0.83	0.032	0.005	**2.82E−10**
120	20	1,328,766	1,382,035	rs6041774	T	C	0.11	0.034	0.006	**1.39E−08**
122	20	3,217,989	3,415,566	rs6115827	A	G	0.67	0.022	0.004	**1.75E−08**
123	20	61,440,005	61,455,532	rs61734651	T	C	0.07	0.054	0.008	**6.33E−11**
124	21	43,809,092	43,831,005	rs45598239	T	C	0.05	0.067	0.008	**1.13E−15**
125	22	38,077,995	38,503,479	rs5756795	T	C	0.57	−0.036	0.004	**1.18E−22**
126	22	50,952,735	51,157,353	rs36062310	A	G	0.04	0.115	0.010	**8.65E−30**

^a^Locus start and end positions (in bp)

^b^Bolded *p* values indicate p values below genome-wide significance threshold (*p* < 5 × 10^−8^)

*GWS*, genome-wide significant (*p* < 5 × 10^−8^); *Locus*, the index number identifier used to refer to independent loci identified by this GWAS; *SNP*, single nucleotide polymorphism; *Chr*, chromosome; *Start*, locus start position in base pairs (GR37 Human Genome Build/hg19 coordinates); *Stop*, locus stop position; *A1*, coded allele (effects and allele frequencies are coded in terms of copies of this allele); *A2*, non-coded allele; *A*, adenosine; *C*, cytosine; *G*, guanidine; *T*, thymidine; *A1 Freq*, frequency of allele 1

Gene-based analyses (MAGMA) identified 192 genome-wide significant genes, of which 100 are novel (Supplementary Table 12). Competitive set-based analysis of pre-defined curated gene sets and gene ontology (GO) terms identified three significant gene sets, GOBP_SENSORY_PERCEPTION_OF_MECHANICAL_STIMULUS (*p* = 6.42E−09), GOMF_ACTIN_BINDING (*p* = 2.55E−06), and GOMF_CYTOSKELETAL_PROTEIN_BINDING (*p* = 1.63E−06) (Supplementary Tables 13 and 14).

To identify tissues and pathways underlying these gene-based associations, we performed a MAGMA tissue expression analysis with 31 general human tissue types, including human inner ear tissue from van der Valk et al. [44] and found significant enrichment in the inner ear (*p* = 2.66 × 10^–8^), nerve (*p* = 1.50 × 10^–5^), and pituitary (*p* = 8.66 × 10^–4^) of EUA MVP, UKB, and in meta-analysis. Analysis of 71 different tissue types identified significant enrichment in the cerebellum (*p* = 2.33 × 10^–5^), cerebellar hemisphere (*p* = 6.22 × 10^–5^), and tibial nerve (*p* = 3.65 × 10^–4^), as well as 13 inner ear cell types (Supplementary Table 15 and Fig. S4).

Please note that these gene-tissue analyses were based on GTEx v8 datasets, which do not include the human cochlea, the tissue most relevant to hearing loss. We enriched with human data of one subject only [44] which had been labeled “utricle”; however, this analysis identified cochlear cell types as well as vestibular tissue. Because of the paucity of human cochlear data, we performed additional enrichment analyses based on available mouse data from a recent, large study on cochlear cells including 58 cell types [45] (Supplementary Table 16 and Fig. S5). Hair cells, particularly outer hair cells, were significantly enriched when conditioned on the average expression of all cell types as the baseline.

### Polygenic Risk Scores (PRS)

As an additional means to validate overlapping genetic signal between the different SNHL GWAS, we performed cross-dataset genetic risk score predictions (PRS) for SNHL in EUA target samples. Using MVP as training sample, the SNHL PRS explained 0.8% of the phenotypic variation in the UKB target sample (liability scale, assuming a 30% population prevalence), and similarly, the UKB-based PRS explained 0.7% of the variability in MVP. Risk was significantly different across the range of SNHL PRS (Fig. S6). For example, in the UKB, individuals in the highest quintile of hearing PRS had 1.49 times the odds of SNHL (log odds s.e. = 0.013; 95% confidence interval (CI) = (1.45, 1.53); *p* = 1.27 × 10^−205^) than individuals in the lowest quintile. Similarly, in the MVP, individuals in the highest quintile of hearing PRS had 1.48 times the odds of SNHL (log odds s.e. = 0.011; 95% confidence interval (CI) = (1.44, 1.51); *p* = 2.16 × 10^−251^) than individuals in the lowest quintile.

### Examination of Implicated Genes in the Context of Congenital Hearing Loss

The Hereditary Hearing Loss database was used to compare findings from our analyses to genes identified in the context of congenital hearing loss. Of the 196 protein-coding genes (accessed August 9, 2024), 17 genes were implicated by our GWAS from either positional mapping of GWS loci or gene-based analyses (Supplementary Table 17). Twelve out of the 17 are members of significant gene ontology (GO terms) from our gene-set analysis.

Finally, we performed a partitioned heritability analysis for the SNHL EUA MVP and UKB meta-analysis (*N* = 598,899 European subjects from MVP and UKB), partitioning SNPs into 3 groups: SNPs in protein-coding genes (in the Hereditary Hearing Loss database), SNPs in protein-coding genes (not in the database), and SNPs outside of protein-coding genes (Table 2). SNP-based heritability estimates including 196 known CHL genes showed a 3.26-fold enrichment relative to all other protein-coding genes. However, the large majority of risk (99% of SNPs, explaining 97% of *h*^2^_SNP_) for polygenic SNHL is captured by SNPs outside of congenital hearing loss genes.

**Table 2:** Partitioned heritability estimates for sensorineural hearing loss GWAS (*N* = 598,899 European subjects from MVP and UKB)

Category	Proportion of all SNPs	Proportion of *h*^2^_SNP_	**SE**	Enrichment^a^
All SNPs	1.00	1.00	0.00	1.00
SNPs in protein coding genes (in HHLdb)	0.01	0.03	0.01	4.58
SNPs in protein coding genes (not in HHLdb)	0.38	0.54	0.02	1.40
SNPs outside of protein coding genes	0.61	0.43	0.02	0.71

^a^Calculated as the proportion of *h*^2^_SNP_ accounted for by the category divided by the proportion of the genome-wide set of SNPs that were annotated to the category

*HHLdb*, Hereditary Hearing Loss database; *h*^*2*^_SNP_, SNP-based heritability

## Discussion

### Million Veteran Program Multi-ancestry Analysis

This study introduces MVP hearing data based solely on a clinical diagnosis of sensorineural hearing loss. MVP GWAS alone elicits a total of 57 distinct loci with 76 genes in gene-based analysis and significant relevant otologic pathways implicated by gene-set analysis. We demonstrate that this phenotype approach is broadly successful for variant identification. A benefit of our method is the exclusion of many other diseases that lead to hearing loss, e.g., acoustic neuroma, otosclerosis, sudden hearing loss, etc., which may have distinct genetic architectures that can lead to inflation in the results.

In addition to a phenotypic difference between MVP and UKB, i.e., ICD versus self-report, the two populations have contrasting demographics, environmental exposures, and slightly different mean ages. Unlike the civilian population of the UKB, MVP is a group of ambulatory US Veterans consisting of greater than 90% males who have been exposed to noise levels during their military career [53] of up to 126 dB [17], as well as a 20% prevalence of head injury, another cause for hearing loss [54]. Although we cannot distinguish the effects of noise and head trauma from age in this study, the MVP cohort might have elicited the phenotypic expression of variants related to these differences.

Nevertheless, despite these dissimilarities, there appears to be a commonality of hearing loss genetic architecture between the MVP and UKB datasets where the studies estimated 3624 (67.0%) shared causal variants. Importantly, this similarity indicates that the data are suitable for aggregation, and meta-analysis of the two identified 108 loci [12, 16], 54 with a novel prioritized and/or protein-coding gene, and a total of 100 significant novel genes in gene-analysis.

### Novel Prioritized Genes

Among our newly reported genes, at least five of the “top ten hits” within novel prioritized genes are worth noting as cochlear-related (Supplementary Table 10). *DPT* has been identified in familial Meniere’s disease, a syndrome consisting of episodic hearing loss, vertigo, and tinnitus, and is an extracellular matrix protein that functions in cell matrix interactions and assembly [55]. *SETBP1* mutations are a cause of Schinzel-Giedion syndrome, characterized by distinct facial features, malformations, and SNHL [56]. *WNT7A* regulates axon outgrowth in the cochlea [57] during inner ear development and is responsible for planarity of hair cell bundles [58]. *EVL* localizes at the cuticular plate supporting inner ear hair cells [59]. Expression of the transcription factor *BACH2*, under control of *miR-96* in the murine model, is altered in noise trauma and plays a mediation role in oxidative stress [60].

### Hypothesis of Functionality and Future Directions

Partitioned SNP-based heritability based on our results of known congenital hearing loss genes (CHL) showed a 3.26-fold enrichment relative to other protein-coding genes. This is in agreement with other studies tying CHL genes to the polygenic disorder of adult SNHL, where polygenic risk scores from a self-reported hearing loss GWAS predicted higher odds of children’s hearing loss [61]. Ultrarare variants (MAF < 0.0001) in exomes of CHL have been posited to account for ~ 25% of severe adult hearing loss [62]. Separately, multiple studies of self-reported hearing difficulties have also identified an increased burden of rare variants within CHL genes [16, 20, 63–66]. Thus, while childhood hearing loss is known to be at least 50% monogenic in origin [67], it appears that adult-onset SNHL has genetic signals in common with CHL.

**Fig. 3:**
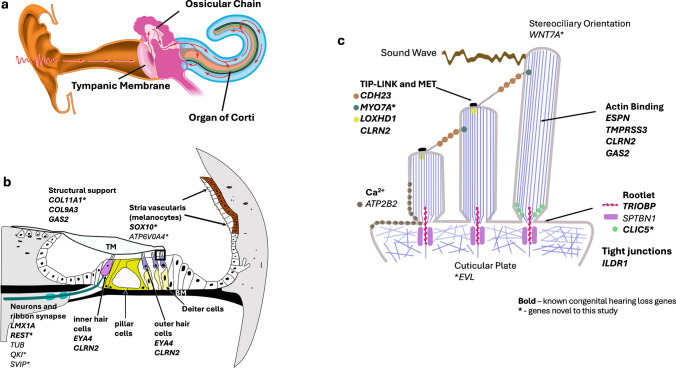
Putative influences of significant hearing loss genes on cochlear function. **a** Cross-section of ear. Sound waves travel through the external auditory canal, vibrating the tympanic membrane and ossicular chain, continuing as a fluid wave into the cochlea. The Organ of Corti (OC) is suspended within the center of the cochlea (green). **b** Cross-section of the OC, supported by the basilar membrane below (BM). Movement is restricted by the tectorial membrane above. The shearing motion of the fluid endolymph wave motion against OHC stereocilia induces a dampening, focusing the wave towards the locally juxtaposed inner hair cells (IHCs). IHCs then transduce this movement into an electric signal, releasing neurotransmitter at the ribbon synapse. Post-synaptic neurons of the cochlear nerve transmit signals to the auditory cortex. The stria vascularis maintains the electric potential of endolymph that bathes the apices of hair cells. **c** Expanded area of apex of hair cells from **b**, showing stereociliary movement of both OHCs and IHCs. Fluid/sound wave movement pushes stereocilia laterally, stretching tip-links attaching the taller stereocilium to its adjacent shorter one. The stereocilium’s parallel f-actin fibers are critical for stiffness and rigidity of the stereocilia, while allowing for bending at the reinforced rootlet at the attachment of the stereocilia to the apical surface of the hair cell. The rootlet is the focus area of vibration secondary to sound as measured by high-resolution optics^73^. Tip-links consist of chained protein threads connecting sequential rows of stereociliae, from the top of the shorter stereocilia to the side of the next taller structure. This movement opens a mechanoelectrical transduction (MET) channel at its tip (black), allowing influx of Ca++ and K+ and triggering depolarization at the base of the cell. Tight junctions between hair cells and supporting cells separate endolymph bathing the stereocilia from its cell bodies. Genes that are not color-coded are ubiquitous, involved in development and/or maintenance

To organize our results, it is helpful to examine the function of the genes identified as significant in this study, including known CHL genes that are directly related to inner ear physiology (see Fig. 3), particularly within the context of the stereocilia of the inner and outer hair cells. While individual studies have identified the function of these genes and all are proven to be associated with CHL, the theoretical basis for this discussion will require laboratory evidence and confirmation of loss of function.

Parallel actin fibers in stereocilia appear to be critical for stiffness in response to the fluid wave. Within these structures, *ESPN* encodes an actin-binding protein required for assembly and stabilization [68], *TMPRSS3* and *CLRN2* express stereociliary proteins critical for hair cell survival [69], and *WNT7A* directs the uniform 3-dimensional polarity of outer hair cells.

In response to the fluid/sound wave, stereociliae of hair cells bend at their rootlets (Fig. 3b, c). *TRIOBP*, *CLIC5*, and *ATP2B2*, all non-syndromic congenital hearing loss genes, are expressed in this subcellular area [70–73]. *TRIOBP* wraps around actin providing stiffness and support, and *CLIC5* is a chloride channel protein that stabilizes the attachment of the stereocilia plasma membrane to its base [74–76]. *ATP2B2* expresses a transmembrane ATPase at the lower half of the stereocilia and cuticular plate, where it regulates Ca^++^ concentration within the cytosol. Although not on the CHL list, *SPTBN1* is expressed as part of the support structure at the area of the rootlet as well. *SPTBN1* is associated with an autosomal dominant neurodevelopmental disorder which includes SNHL [77]. In support of the “sensory mechanical stimulus pathway” (*p-*value 6.42E−09), both human- and murine-based transcription analyses of the organ of Corti indicate hair cell enrichment, particularly outer hair cell in both MVP and UKB cohorts (Supplementary Table 15).

Bending at the rootlet allows for increased tension on tip-links, which in turn controls the opening of the mechanotransduction ion channel (MET). *CDH23*, prioritized by FLAME [38], constitutes the upper half of the tip-link, and *MYO7A* tethers this link to an actin bundle within the stereocilium. These genes are responsible for different forms of Usher’s syndrome, the most common genetic cause of congenital deafness. In this critical area of the sensory apparatus, both *CLRN2* and *LOXHD1* are engaged in MET maintenance at the tip of the stereocilia [78].

OHCs are tethered at their apex and base. At their apex, the tectorial membrane (TM) (Fig. 3b) [78] is a gelatinous structure providing a static force to the shearing motion of the sound wave. *COL11A1* and *COL9A3* code for two α-chain collagens providing upper support within the tectorial membrane and individually cause different variants of Stickler’s syndrome [79]. Other significant support genes include *GAS2*, identified in pillar and Deiters’ cells as a microtubule regulatory gene, maintaining arrangement of the actin skeleton [80].

In a separate micro-anatomical area, our human cochlear cell transcription analysis noted enrichment within melanocytes, located in the stria vascularis, and functioning to maintain the endocochlear potential. Although not on the CHL list, *ATP6V0A4,* identified within “sensory perception of mechanical stimulus,” is a known hearing loss gene [81] expressed in the basal stria cells, where it acts as a proton pump, maintaining the pH of endolymph bathing the apical region of the hair cell. *SOX10* (Waardenberg’s Syndrome), also found within the stria vascularis, expresses an aquaporin repressor [82].

Associated with the nerve signal through the spiral ganglion, other significant congenital hearing loss genes were identified related to neurons, the ribbon synapse, and the myelin sheath (Fig. 3b). Studies of both age-related and noise-induced SNHL have found that after injury, up to 50% of ribbon synapses can be lost prior to an objective audiogram threshold change [83, 84] and this finding has been correlated to loss of speech intelligibility [85, 86]. These synapses connect the IHC cells to Type I spiral ganglion nerve cells [87]. Located within this synaptic area are *LMX1A*, a neuro-restrictive silencer [88] expressed in Type I spiral ganglion nerves. *QKI* is identified in a murine model as essential for proper myelination of spiral ganglion neurons and auditory nerve fibers [86]. In aggregate, these genes identified as significant in prioritization and gene-based analysis provide putative points of injury to the hearing sensory mechanism to be explored in future studies.

The authors suggest this as one plausible approach to the role of congenital hearing loss genes in the polygenic sensorineural hearing disorder. To identify causative variants, genes, and pathways, further study with a combination of whole genome sequencing, copy number variations, epigenetic, human cochlear expression data, and laboratory proof-of-concept will be required.

## Limitations

This study has limitations. Although an ICD diagnosis is a step towards an objective phenotype, it does not describe the state of individual audiogram frequencies, and heritability studies have indicated that a good portion of the heritability of SNHL is frequency-specific [89]. Further, while the genes identified provide biologically plausible explanations for SNHL, until now, expression data in the human cochlea has not been available. While this study used expression data from one human inner ear sample, further study for causative variants requires whole genome sequencing (WGS) and multiple human cochlear methylation/expression studies.

Further, GWAS remains underpowered to detect rare variants with larger effect sizes, which has been noted to contribute to “missing heritability.” Particularly ultrarare variants in exomes (allele frequency < 0.0001) appear to account for ~ 25% of severe presbycusis [62]. Although the strength of GWAS is in its unbiased observational sampling of the entire genome, nevertheless, because of linkage disequilibrium, GWAS cannot resolve truly causal variants within a significant locus. While we performed FRAMES to prioritize the most-likely gene within each locus and examined functional approaches through MAGMA, further WGS of positive results will aid in identifying specific causal variants within exons, introns, transcription sites, intergenic regions, etc.

Although the ancestral make-up of MVP reflects its population, there continues to be reduced statistical power to discover significant variants in Indigenous/Latin American and African American ancestries. While we identified one locus in AFA with a plausible gene, *TUB*, these ancestries have high rates of age-related and noise-induced hearing loss as well, and larger cohorts of diverse ancestries are required for analysis and identification of unique significant variants.

## Conclusions

In GWAS of SNHL combining MVP and UKB, we have identified 108 loci containing 54 novel protein-coding genes and/or prioritized genes, 100 novel genes in gene-based analysis, and significant pathways related to the structure of the cochlea. Despite different phenotypes, demographics, and exposures, we find substantial overlapping genetic signals. Although over 95% of the risk for this polygenic disorder is captured by SNPs outside of congenital hearing loss genes, SNP-based heritability estimates 3.26-fold enrichment of these genes relative to all other protein-coding genes. These findings will provide new targets for investigation of druggable genes and pathways for treatment of this pervasive and currently permanent condition. Our next tasks are to analyze genetic signals from individual audiogram frequencies within larger studies and search for phenotypes with increased heritability of this pervasive disorder.

## Supplementary Information

Below is the link to the electronic supplementary material.

Supplementary file1 (XLSX 5.35 MB)

Supplementary file2 (DOCX 2.36 MB)

Supplementary file3 (PDF 68.3 MB)

## Data Availability

Summary statistics for MVP analyses will be deposited upon publication on dbGaP under accession number phs001672 (https://www.ncbi.nlm.nih.gov/projects/gap/cgi-bin/study.cgi?study_id=phs001672.v11.p1). MVP summary data access can be obtained by submitting a data access request through dbGaP; raw data are protected and are not available due to privacy reasons.

## Code Availability

The codes for the analysis are available on Github (https://github.com/nievergeltlab/).

## Abbreviations

AFA: African Americans
AIC: Akaike information criterion
ANNOVAR: Annotation variation software
BM: Basement membrane
CADD: Combined annotation dependent depletion
CHL: Congenital hearing loss
EUA: European ancestry
FUMA: Functional mapping and annotation
GO: Gene ontology
GWAS: Genome-wide association study
GWS: Genome-wide significant
*h*_*2*_SNP: Heritability of single nucleotide polymorphisms
IAA: Indigenous American ancestry
ICD: International classification of diseases
IHC: Inner hair cells
LD: Linkage disequilibrium
LDSC: Linkage disequilibrium score regression
MAF: Minor allele frequency
MAGMA: Multi-marker analysis of genomic annotation
MET: Mechanoelectrical transduction channels
MsigDB: Molecular signatures database
MVP: Million Veteran Program
OC: Organ of Corti
OHC: Outer hair cells
PC: Principal component
SNHL: Sensorineural hearing loss
SNP: Single nucleotide polymorphism
TM: Tectorial membrane
UKB: United Kingdom Biobank
WGS: Whole-genome sequencing
YLD: Years lived with disability

## References

[1] Nocini R, Henry BM, Lippi G, Mattiuzzi C (2023) Estimating the worldwide burden of health loss due to hearing loss. Eur J Public Health 33(1):146–148. 10.1093/eurpub/ckac171 36377968 10.1093/eurpub/ckac171 PMC9897997

[2] Dawes P, Emsley R, Cruickshanks KJ, Moore DR, Fortnum H, Edmondson-Jones M et al (2015) Hearing loss and cognition: the role of hearing AIDS, social isolation and depression. PLoS ONE 10(3):e0119616. 10.1371/journal.pone.0119616 25760329 10.1371/journal.pone.0119616 PMC4356542

[3] Yévenes-Briones H, Caballero FF, Struijk EA, Rey-Martinez J, Montes-Jovellar L, Graciani A et al (2021) Association between hearing loss and impaired physical function, frailty, and disability in older adults: a cross-sectional study. JAMA Otolaryngol Head Neck Surg 147(11):951–958. 10.1001/jamaoto.2021.2399 34554203 10.1001/jamaoto.2021.2399 PMC8461549

[4] Jafari Z, Kolb BE, Mohajerani MH (2019) Age-related hearing loss and tinnitus, dementia risk, and auditory amplification outcomes. Ageing Res Rev 56:100963. 10.1016/j.arr.2019.100963 31557539 10.1016/j.arr.2019.100963

[5] Dobie RA (2008) The burdens of age-related and occupational noise-induced hearing loss in the United States. Ear Hear 29(4):565–577. 10.1097/AUD.0b013e31817349ec 18469718 10.1097/AUD.0b013e31817349ec

[6] Duan H, Zhang D, Liang Y, Xu C, Wu Y, Tian X et al (2019) Heritability of age-related hearing loss in middle-aged and elderly Chinese: a population-based twin study. Ear Hear 40(2):253–259. 10.1097/aud.0000000000000610 29794565 10.1097/AUD.0000000000000610

[7] Bogo R, Farah A, Johnson AC, Karlsson KK, Pedersen NL, Svartengren M et al (2015) The role of genetic factors for hearing deterioration across 20 years: a twin study. J Gerontol A Biol Sci Med Sci 70(5):647–653. 10.1093/gerona/glu245 25665831 10.1093/gerona/glu245

[8] Hereditary Hearing Loss (n.d.) Homepage. https://hereditaryhearingloss.org. Accessed 1 Nov 2026

[9] Fransen E, Bonneux S, Corneveaux JJ, Schrauwen I, Di Berardino F, White CH et al (2015) Genome-wide association analysis demonstrates the highly polygenic character of age-related hearing impairment. Eur J Hum Genet 23(1):110–115. 10.1038/ejhg.2014.56 24939585 10.1038/ejhg.2014.56 PMC4266741

[10] De Angelis F, Zeleznik OA, Wendt FR, Pathak GA, Tylee DS, De Lillo A et al (2023) Sex differences in the polygenic architecture of hearing problems in adults. Genome Med 15(1):36. 10.1186/s13073-023-01186-3 37165447 10.1186/s13073-023-01186-3 PMC10173489

[11] Jiang L, Wang D, He Y, Shu Y (2023) Advances in gene therapy hold promise for treating hereditary hearing loss. Mol Ther 31(4):934–950. 10.1016/j.ymthe.2023.02.001 36755494 10.1016/j.ymthe.2023.02.001 PMC10124073

[12] Wells HRR, Freidin MB, Zainul Abidin FN, Payton A, Dawes P, Munro KJ et al (2019) GWAS identifies 44 independent associated genomic loci for self-reported adult hearing difficulty in UK Biobank. Am J Hum Genet 105(4):788–802. 10.1016/j.ajhg.2019.09.008 31564434 10.1016/j.ajhg.2019.09.008 PMC6817556

[13] McCullagh MC, Raymond D, Kerr MJ, Lusk SL (2011) Prevalence of hearing loss and accuracy of self-report among factory workers. Noise Health 13(54):340–347. 10.4103/1463-1741.85504 21959114 10.4103/1463-1741.85504

[14] Brennan-Jones CG, Taljaard DS, Brennan-Jones SE, Bennett RJ, Swanepoel dW, Eikelboom RH (2016) Self-reported hearing loss and manual audiometry: a rural versus urban comparison. Aust J Rural Health 24(2):130–135. 10.1111/ajr.12227 26311193 10.1111/ajr.12227

[15] Cherny SS, Livshits G, Wells HRR, Freidin MB, Malkin I, Dawson SJ et al (2020) Self-reported hearing loss questions provide a good measure for genetic studies: a polygenic risk score analysis from UK Biobank. Eur J Hum Genet 28(8):1056–1065. 10.1038/s41431-020-0603-2 32203203 10.1038/s41431-020-0603-2 PMC7382483

[16] Trpchevska N, Freidin MB, Broer L, Oosterloo BC, Yao S, Zhou Y et al (2022) Genome-wide association meta-analysis identifies 48 risk variants and highlights the role of the stria vascularis in hearing loss. Am J Hum Genet 109(6):1077–1091. 10.1016/j.ajhg.2022.04.010 35580588 10.1016/j.ajhg.2022.04.010 PMC9247887

[17] Jokel C, Yankaskas K, Robinette MB (2019) Noise of military weapons, ground vehicles, planes and ships. J Acoust Soc Am 146(5):3832. 10.1121/1.5134069 31795677 10.1121/1.5134069

[18] Praveen K, Dobbyn L, Gurski L, Ayer AH, Staples J, Mishra S et al (2022) Population-scale analysis of common and rare genetic variation associated with hearing loss in adults. Commun Biol 5(1):540. 10.1038/s42003-022-03408-7 35661827 10.1038/s42003-022-03408-7 PMC9166757

[19] Naderi E, Cornejo-Sanchez DM, Li G, Schrauwen I, Wang GT, Dewan AT et al (2023) The genetic contribution of the X chromosome in age-related hearing loss. Front Genet 14:1106328. 10.3389/fgene.2023.1106328 36896235 10.3389/fgene.2023.1106328 PMC9988903

[20] Ivarsdottir EV, Holm H, Benonisdottir S, Olafsdottir T, Sveinbjornsson G, Thorleifsson G et al (2021) The genetic architecture of age-related hearing impairment revealed by genome-wide association analysis. Commun Biol 4(1):706. 10.1038/s42003-021-02224-9 34108613 10.1038/s42003-021-02224-9 PMC8190123

[21] Liu W, Johansson A, Rask-Andersen H, Rask-Andersen M (2021) A combined genome-wide association and molecular study of age-related hearing loss in *H. sapiens*. BMC Med 19(1):302. 10.1186/s12916-021-02169-0 34847940 10.1186/s12916-021-02169-0 PMC8638543

[22] Kalra G, Milon B, Casella AM, Herb BR, Humphries E, Song Y et al (2020) Biological insights from multi-omic analysis of 31 genomic risk loci for adult hearing difficulty. PLoS Genet 16(9):e1009025. 10.1371/journal.pgen.1009025 32986727 10.1371/journal.pgen.1009025 PMC7544108

[23] Gaziano JM, Concato J, Brophy M, Fiore L, Pyarajan S, Breeling J et al (2016) Million veteran program: a mega-biobank to study genetic influences on health and disease. J Clin Epidemiol 70:214–223. 10.1016/j.jclinepi.2015.09.016 26441289 10.1016/j.jclinepi.2015.09.016

[24] Loh PR, Palamara PF, Price AL (2016) Fast and accurate long-range phasing in a UK Biobank cohort. Nat Genet 48(7):811–816. 10.1038/ng.3571 27270109 10.1038/ng.3571 PMC4925291

[25] Das S, Forer L, Schönherr S, Sidore C, Locke AE, Kwong A et al (2016) Next-generation genotype imputation service and methods. Nat Genet 48(10):1284–1287. 10.1038/ng.3656 27571263 10.1038/ng.3656 PMC5157836

[26] McCarthy S, Das S, Kretzschmar W, Delaneau O, Wood AR, Teumer A et al (2016) A reference panel of 64,976 haplotypes for genotype imputation. Nat Genet 48(10):1279–1283. 10.1038/ng.3643.PMID27548312 27548312 10.1038/ng.3643 PMC5388176

[27] Chen CY, Pollack S, Hunter DJ, Hirschhorn JN, Kraft P, Price AL (2013) Improved ancestry inference using weights from external reference panels. Bioinformatics 29(11):1399–1406. 10.1093/bioinformatics/btt144 23539302 10.1093/bioinformatics/btt144 PMC3661048

[28] Nievergelt CM, Maihofer AX, Klengel T, Atkinson EG, Chen CY, Choi KW et al (2019) International meta-analysis of PTSD genome-wide association studies identifies sex- and ancestry-specific genetic risk loci. Nat Commun 10(1):4558. 10.1038/s41467-019-12576-w 31594949 10.1038/s41467-019-12576-w PMC6783435

[29] Chang CC, Chow CC, Tellier LC, Vattikuti S, Purcell SM, Lee JJ (2015) Second-generation PLINK: rising to the challenge of larger and richer datasets. Gigascience 4:7. 10.1186/s13742-015-0047-8 25722852 10.1186/s13742-015-0047-8 PMC4342193

[30] Manichaikul A, Mychaleckyj JC, Rich SS, Daly K, Sale M, Chen WM (2010) Robust relationship inference in genome-wide association studies. Bioinformatics 26(22):2867–2873. 10.1093/bioinformatics/btq559.PMID20926424 20926424 10.1093/bioinformatics/btq559 PMC3025716

[31] Abraham G, Qiu Y, Inouye M (2017) FlashPCA2: principal component analysis of Biobank-scale genotype datasets. Bioinformatics 33(17):2776–2778. 10.1093/bioinformatics/btx299 28475694 10.1093/bioinformatics/btx299

[32] Cook JP, Mahajan A, Morris AP (2017) Guidance for the utility of linear models in meta-analysis of genetic association studies of binary phenotypes. Eur J Hum Genet 25(2):240–245. 10.1038/ejhg.2016.150 27848946 10.1038/ejhg.2016.150 PMC5237383

[33] Willer CJ, Li Y, Abecasis GR (2010) METAL: fast and efficient meta-analysis of genomewide association scans. Bioinformatics 26(17):2190–2191. 10.1093/bioinformatics/btq340 20616382 10.1093/bioinformatics/btq340 PMC2922887

[34] Watanabe K, Taskesen E, van Bochoven A, Posthuma D (2017) Functional mapping and annotation of genetic associations with FUMA. Nat Commun 8(1):1826. 10.1038/s41467-017-01261-5 29184056 10.1038/s41467-017-01261-5 PMC5705698

[35] Wang K, Li M, Hakonarson H (2010) ANNOVAR: functional annotation of genetic variants from high-throughput sequencing data. Nucleic Acids Res 38(16):e164. 10.1093/nar/gkq603 20601685 10.1093/nar/gkq603 PMC2938201

[36] Rentzsch P, Witten D, Cooper GM, Shendure J, Kircher M (2019) CADD: predicting the deleteriousness of variants throughout the human genome. Nucleic Acids Res 47(D1):D886–D894. 10.1093/nar/gky1016 30371827 10.1093/nar/gky1016 PMC6323892

[37] Dong S, Zhao N, Spragins E, Kagda MS, Li M, Assis P et al (2023) Annotating and prioritizing human non-coding variants with RegulomeDB v.2. Nat Genet 55(5):724–726. 10.1038/s41588-023-01365-3 37173523 10.1038/s41588-023-01365-3 PMC10989417

[38] Schipper M, de Leeuw CA, Maciel B, Wightman DP, Hubers N, Boomsma DI et al (2025) Prioritizing effector genes at trait-associated loci using multimodal evidence. Nat Genet 57(2):323–333. 10.1038/s41588-025-02084-7 39930082 10.1038/s41588-025-02084-7

[39] Hutchinson A, Watson H, Wallace C (2020) Improving the coverage of credible sets in Bayesian genetic fine-mapping. PLoS Comput Biol 16(4):e1007829. 10.1371/journal.pcbi.1007829 32282791 10.1371/journal.pcbi.1007829 PMC7179948

[40] Weeks EM, Ulirsch JC, Cheng NY, Trippe BL, Fine RS, Miao J et al (2023) Leveraging polygenic enrichments of gene features to predict genes underlying complex traits and diseases. Nat Genet 55(8):1267–1276. 10.1038/s41588-023-01443-6 37443254 10.1038/s41588-023-01443-6 PMC10836580

[41] Pruim RJ, Welch RP, Sanna S, Teslovich TM, Chines PS, Gliedt TP et al (2010) LocusZoom: regional visualization of genome-wide association scan results. Bioinformatics 26(18):2336–2337. 10.1093/bioinformatics/btq419 20634204 10.1093/bioinformatics/btq419 PMC2935401

[42] de Leeuw CA, Mooij JM, Heskes T, Posthuma D (2015) MAGMA: generalized gene-set analysis of GWAS data. PLoS Comput Biol 11(4):e1004219. 10.1371/journal.pcbi.1004219 25885710 10.1371/journal.pcbi.1004219 PMC4401657

[43] Liberzon A, Birger C, Thorvaldsdottir H, Ghandi M, Mesirov JP, Tamayo P (2015) The Molecular Signatures Database (MSigDB) hallmark gene set collection. Cell Syst 1(6):417–425. 10.1016/j.cels.2015.12.004 26771021 10.1016/j.cels.2015.12.004 PMC4707969

[44] van der Valk WH, van Beelen ESA, Steinhart MR, Nist-Lund C, Osorio D, de Groot J et al (2023) A single-cell level comparison of human inner ear organoids with the human cochlea and vestibular organs. Cell Rep 42(6):112623. 10.1016/j.celrep.2023.112623 37289589 10.1016/j.celrep.2023.112623 PMC10592453

[45] Boussaty EC, Tedeschi N, Novotny M, Ninoyu Y, Eric Du, Draf C, Zhang Y, Manor U, Scheuermann RH, Friedman R (2023) Cochlear transcriptome analysis of an outbred mouse population (CFW). Frontiers in Cellular Neuroscience. 17:17. PMID 1256619 10.3389/fncel.2023.1256619 PMC10716316 38094513

[46] Blake JA, Baldarelli R, Kadin JA, Richardson JE, Smith CL, Bult CJ et al (2021) Mouse genome database (MGD): knowledgebase for mouse-human comparative biology. Nucleic Acids Res 49(D1):D981–D987. 10.1093/nar/gkaa1083 33231642 10.1093/nar/gkaa1083 PMC7779030

[47] Bulik-Sullivan BK, Loh PR, Finucane HK, Ripke S, Yang J, Patterson N et al (2015) LD score regression distinguishes confounding from polygenicity in genome-wide association studies. Nat Genet 47(3):291–295. 10.1038/ng.3211 25642630 10.1038/ng.3211 PMC4495769

[48] Lee SH, Wray NR, Goddard ME, Visscher PM (2011) Estimating missing heritability for disease from genome-wide association studies. Am J Hum Genet 88(3):294–305. 10.1016/j.ajhg.2011.02.002 21376301 10.1016/j.ajhg.2011.02.002 PMC3059431

[49] Holland D, Frei O, Desikan R, Fan CC, Shadrin AA, Smeland OB et al (2020) Beyond SNP heritability: polygenicity and discoverability of phenotypes estimated with a univariate Gaussian mixture model. PLoS Genet 16(5):e1008612. 10.1371/journal.pgen.1008612 32427991 10.1371/journal.pgen.1008612 PMC7272101

[50] Frei O, Holland D, Smeland OB, Shadrin AA, Fan CC, Maeland S et al (2019) Bivariate causal mixture model quantifies polygenic overlap between complex traits beyond genetic correlation. Nat Commun 10(1):2417. 10.1038/s41467-019-10310-0 31160569 10.1038/s41467-019-10310-0 PMC6547727

[51] Ge T, Chen C-Y, Ni Y, Feng Y-CA, Smoller JW (2019) Polygenic prediction via Bayesian regression and continuous shrinkage priors. Nature communications 10(1):1776 30992449 10.1038/s41467-019-09718-5 PMC6467998

[52] Johnson KR, Zheng QY, Noben-Trauth K (2006) Strain background effects and genetic modifiers of hearing in mice. Brain Res 1091(1):79–88. 10.1016/j.brainres.2006.02.021 16579977 10.1016/j.brainres.2006.02.021 PMC2858224

[53] Yankaskas K (2013) Prelude: noise-induced tinnitus and hearing loss in the military. Hear Res 295:3–8. 10.1016/j.heares.2012.04.016 22575206 10.1016/j.heares.2012.04.016

[54] Chen JX, Lindeborg M, Herman SD, Ishai R, Knoll RM, Remenschneider A et al (2018) Systematic review of hearing loss after traumatic brain injury without associated temporal bone fracture. Am J Otolaryngol 39(3):338–344. 10.1016/j.amjoto.2018.01.018 29506762 10.1016/j.amjoto.2018.01.018

[55] Escalera-Balsera A, Roman-Naranjo P, Lopez-Escamez JA (2020) Systematic review of sequencing studies and gene expression profiling in familial Meniere disease. Genes. 10.3390/genes11121414 33260921 10.3390/genes11121414 PMC7761472

[56] Hoischen A, van Bon BW, Gilissen C, Arts P, van Lier B, Steehouwer M et al (2010) De novo mutations of SETBP1 cause Schinzel-Giedion syndrome. Nat Genet 42(6):483–485. 10.1038/ng.581 20436468 10.1038/ng.581

[57] Munnamalai V, Fekete DM (2013) Wnt signaling during cochlear development. Semin Cell Dev Biol 24(5):480–489. 10.1016/j.semcdb.2013.03.008 23548730 10.1016/j.semcdb.2013.03.008 PMC3690158

[58] Dabdoub A, Donohue MJ, Brennan A, Wolf V, Montcouquiol M, Sassoon DA et al (2003) Wnt signaling mediates reorientation of outer hair cell stereociliary bundles in the mammalian cochlea. Development 130(11):2375–2384. 10.1242/dev.00448 12702652 10.1242/dev.00448

[59] Yan K, Zhang H, Qu C, Sun Y, Sun X, Xu Z (2025) EVL is not essential for cuticular plate and stereocilia development in mouse auditory hair cells. FEBS Lett 599(3):330–339. 10.1002/1873-3468.15021.PMID39300480 39300480 10.1002/1873-3468.15021

[60] Xia R, Jin C, Fei S, Dong T, Wen T, Zhu F et al (2025) Therapeutic restoration of miR-96 prevents hearing loss in mice through modulation of noise-induced and genetic pathways. iScience 28(5):112355. 10.1016/j.isci.2025.112355 40641557 10.1016/j.isci.2025.112355 PMC12245441

[61] Wang J, He F, Shepherd DA, Li S, Lange K, Sung V et al (2024) Polygenic risk scores and hearing loss phenotypes in children. JAMA Otolaryngol Head Neck Surg. 10.1001/jamaoto.2024.3659 39509092 10.1001/jamaoto.2024.3659 PMC11544553

[62] Boucher S, Tai FWJ, Delmaghani S, Lelli A, Singh-Estivalet A, Dupont T et al (2020) Ultrarare heterozygous pathogenic variants of genes causing dominant forms of early-onset deafness underlie severe presbycusis. Proceedings of the National Academy of Sciences 117(49):31278–31289 10.1073/pnas.2010782117 PMC7733833 33229591

[63] Hui D, Mehrabi S, Quimby AE, Chen T, Chen S, Park J et al (2023) Gene burden analysis identifies genes associated with increased risk and severity of adult-onset hearing loss in a diverse hospital-based cohort. PLoS Genet 19(1):e1010584. 10.1371/journal.pgen.1010584 36656851 10.1371/journal.pgen.1010584 PMC9888707

[64] Lewis MA, Nolan LS, Cadge BA, Matthews LJ, Schulte BA, Dubno JR et al (2018) Whole exome sequencing in adult-onset hearing loss reveals a high load of predicted pathogenic variants in known deafness-associated genes and identifies new candidate genes. BMC Med Genomics 11(1):77. 10.1186/s12920-018-0395-1 30180840 10.1186/s12920-018-0395-1 PMC6123954

[65] Lewis MA, Schulte BA, Dubno JR, Steel KP (2022) Investigating the characteristics of genes and variants associated with self-reported hearing difficulty in older adults in the UK Biobank. BMC Biol 20(1):150. 10.1186/s12915-022-01349-5 35761239 10.1186/s12915-022-01349-5 PMC9238072

[66] Cornejo-Sanchez DM, Li G, Fabiha T, Wang R, Acharya A, Everard JL et al (2023) Rare-variant association analysis reveals known and new age-related hearing loss genes. Eur J Hum Genet 31(6):638–647. 10.1038/s41431-023-01302-2 36788145 10.1038/s41431-023-01302-2 PMC10250305

[67] Khela H, Kenna MA (2020) Genetics of pediatric hearing loss: a functional perspective. Laryngoscope Investig Otolaryngol 5(3):511–519. 10.1002/lio2.390 32596495 10.1002/lio2.390 PMC7314484

[68] Sekerkova G, Zheng L, Loomis PA, Mugnaini E, Bartles JR (2006) Espins and the actin cytoskeleton of hair cell stereocilia and sensory cell microvilli. Cell Mol Life Sci 63(19–20):2329–2341. 10.1007/s00018-006-6148-x 16909209 10.1007/s00018-006-6148-x PMC2522319

[69] Dunbar LA, Patni P, Aguilar C, Mburu P, Corns L, Wells HR et al (2019) Clarin‐2 is essential for hearing by maintaining stereocilia integrity and function. EMBO Mol Med 11(9):e10288. 10.15252/emmm.201910288 31448880 10.15252/emmm.201910288 PMC6728604

[70] Katsuno T, Belyantseva IA, Cartagena-Rivera AX, Ohta K, Crump SM, Petralia RS et al (2019) TRIOBP-5 sculpts stereocilia rootlets and stiffens supporting cells enabling hearing. JCI Insight. 10.1172/jci.insight.128561 31217345 10.1172/jci.insight.128561 PMC6629139

[71] Fasquelle L, Scott HS, Lenoir M, Wang J, Rebillard G, Gaboyard S et al (2011) Tmprss3, a transmembrane serine protease deficient in human DFNB8/10 deafness, is critical for cochlear hair cell survival at the onset of hearing. J Biol Chem 286(19):17383–17397. 10.1074/jbc.M110.190652 21454591 10.1074/jbc.M110.190652 PMC3089580

[72] Liu W, Löwenheim H, Santi PA, Glueckert R, Schrott-Fischer A, Rask-Andersen H (2018) Expression of trans-membrane serine protease 3 (TMPRSS3) in the human organ of Corti. Cell Tissue Res 372(3):445–456. 10.1007/s00441-018-2793-2 29460002 10.1007/s00441-018-2793-2 PMC5949142

[73] Pacentine I, Chatterjee P, Barr-Gillespie PG (2020) Stereocilia rootlets: actin-based structures that are essential for structural stability of the hair bundle. Int J Mol Sci. 10.3390/ijms21010324 31947734 10.3390/ijms21010324 PMC6981779

[74] Cooper NP, Vavakou A, van der Heijden M (2018) Vibration hotspots reveal longitudinal funneling of sound-evoked motion in the mammalian cochlea. Nat Commun 9(1):3054. 10.1038/s41467-018-05483-z 30076297 10.1038/s41467-018-05483-z PMC6076242

[75] Gagnon LH, Longo-Guess CM, Berryman M, Shin JB, Saylor KW, Yu H et al (2006) The chloride intracellular channel protein CLIC5 is expressed at high levels in hair cell stereocilia and is essential for normal inner ear function. J Neurosci 26(40):10188–10198. 10.1523/jneurosci.2166-06.2006 17021174 10.1523/JNEUROSCI.2166-06.2006 PMC6674616

[76] Silverstein RS, Tempel BL (2006) Atp2b2, encoding plasma membrane Ca2+-ATPase type 2, (PMCA2) exhibits tissue-specific first exon usage in hair cells, neurons, and mammary glands of mice. Neuroscience 141(1):245–257. 10.1016/j.neuroscience.2006.03.036 16675132 10.1016/j.neuroscience.2006.03.036

[77] Cousin MA, Creighton BA, Breau KA, Spillmann RC, Torti E, Dontu S et al (2021) Pathogenic SPTBN1 variants cause an autosomal dominant neurodevelopmental syndrome. Nat Genet 53(7):1006–1021. 10.1038/s41588-021-00886-z 34211179 10.1038/s41588-021-00886-z PMC8273149

[78] Wang P, Miller KK, He E, Dhawan SS, Cunningham CL, Grillet N (2024) LOXHD1 is indispensable for coupling auditory mechanosensitive channels to the site of force transmission. Res Sq. 10.21203/rs.3.rs-3752492/v1.PMID3826048039764105

[79] Shpargel KB, Makishima T, Griffith AJ (2004) Col11a1 and Col11a2 mRNA expression in the developing mouse cochlea: implications for the correlation of hearing loss phenotype with mutant type XI collagen genotype. Acta Otolaryngol 124(3):242–248. 10.1080/00016480410016162 15141750 10.1080/00016480410016162

[80] Chen T, Rohacek AM, Caporizzo M, Nankali A, Smits JJ, Oostrik J et al (2021) Cochlear supporting cells require GAS2 for cytoskeletal architecture and hearing. Dev Cell 56(10):1526-40 e7. 10.1016/j.devcel.2021.04.017 10.1016/j.devcel.2021.04.017 PMC8137675 33964205

[81] Stover EH, Borthwick KJ, Bavalia C, Eady N, Fritz DM, Rungroj N et al (2002) Novel ATP6V1B1 and ATP6V0A4 mutations in autosomal recessive distal renal tubular acidosis with new evidence for hearing loss. J Med Genet 39(11):796–803. 10.1136/jmg.39.11.796 12414817 10.1136/jmg.39.11.796 PMC1735017

[82] Szeto IYY, Chu DKH, Chen P, Chu KC, Au TYK, Leung KKH et al (2022) SOX9 and SOX10 control fluid homeostasis in the inner ear for hearing through independent and cooperative mechanisms. Proc Natl Acad Sci U S A 119(46):e2122121119. 10.1073/pnas.2122121119 36343245 10.1073/pnas.2122121119 PMC9674217

[83] Fernandez KA, Guo D, Micucci S, De Gruttola V, Liberman MC, Kujawa SG (2020) Noise-induced cochlear synaptopathy with and without sensory cell loss. Neuroscience 427:43–57. 10.1016/j.neuroscience.2019.11.051 31887361 10.1016/j.neuroscience.2019.11.051 PMC7450393

[84] Kujawa SG, Liberman MC (2015) Synaptopathy in the noise-exposed and aging cochlea: primary neural degeneration in acquired sensorineural hearing loss. Hear Res 330:191–199. 10.1016/j.heares.2015.02.009 25769437 10.1016/j.heares.2015.02.009 PMC4567542

[85] Bramhall NF, McMillan GP (2024) Perceptual consequences of cochlear deafferentation in humans. Trends Hear 28:23312165241239540. 10.1177/23312165241239541 38738337 10.1177/23312165241239541 PMC11092548

[86] Panganiban CH, Barth JL, Darbelli L, Xing Y, Zhang J, Li H et al (2018) Noise-induced dysregulation of quaking RNA binding proteins contributes to auditory nerve demyelination and hearing loss. J Neurosci 38(10):2551–2568. 10.1523/JNEUROSCI.2487-17.2018 29437856 10.1523/JNEUROSCI.2487-17.2018 PMC5858596

[87] Jean P, Ozcete OD, Tarchini B, Moser T (2019) Intrinsic planar polarity mechanisms influence the position-dependent regulation of synapse properties in inner hair cells. Proc Natl Acad Sci USA 116(18):9084–9093. 10.1073/pnas.1818358116 30975754 10.1073/pnas.1818358116 PMC6500111

[88] Aoki H, Hara A, Kunisada T (2015) White spotting phenotype induced by targeted REST disruption during neural crest specification to a melanocyte cell lineage. Genes Cells 20(5):439–449. 10.1111/gtc.12235 25818501 10.1111/gtc.12235

[89] Wingfield A, Panizzon M, Grant MD, Toomey R, Kremen WS, Franz CE et al (2007) A twin-study of genetic contributions to hearing acuity in late middle age. J Gerontol A Biol Sci Med Sci 62(11):1294–1299. 10.1093/gerona/62.11.1294 18000151 10.1093/gerona/62.11.1294 PMC2945698

