# Supplementary material for: Polygenic Contribution to Sensorineural Hearing Loss Implicates Novel Risk Loci and Convergence with Congenital Hearing Loss Genes
Source: J Assoc Res Otolaryngol. 2026 Mar 16;27(3):447–63. doi: 10.1007/s10162-026-01044-0 (PMC13237364; doi:10.1007/s10162-026-01044-0)
Supplement: Supplementary file 2 — Supplementary file2 (DOCX 2.36 MB) [file 10162_2026_1044_MOESM2_ESM.docx]

**Supplementary File 2**

**Genome-wide association study meta-analysis brings monogenic hearing loss genes into the polygenic realm**

Clifford Royce*^1,2^, Johnson Jacquelyn^1,3^, Mackey Caroline E^1,3^, Mikita Elizabeth A^1,3^, Ryan Allen F^1,2^, Million Veteran Program, Maihofer Adam X^1,3^, Nievergelt Caroline M*^1,3^

1. Veterans Affairs San Diego Healthcare System, Research Service, San Diego, CA, USA
2. University of California San Diego, Department of Otolaryngology – Head and Neck Surgery, La Jolla, CA, USA
3. University of California San Diego, Department of Psychiatry, La Jolla, CA, US

*Corresponding authors: Nievergelt ([cnievergelt@health.ucsd.edu](mailto:cnievergelt@health.ucsd.edu)) and Clifford

([reclifford@health.ucsd.edu](mailto:reclifford@health.ucsd.edu))

**Fig. S1 Ancestry-stratified GWAS and meta-analysis for SNHL in the MVP.**

Manhattan plots of sensorineural hearing loss (SNHL) GWAS conducted within each ancestry strata of the MVP and the multi-ancestry meta-analysis. The red line represents genome-wide significance (GWS) at p < 5 × 10−8. **a)** European ancestry (EUA) GWAS of 176,393 cases and 172,117 controls identified 43 GWS loci. **b)** African ancestry (AFA) GWAS of 22,757 cases and 74,842 controls identified 1 significant locus. **c)** Indigenous American (IAA) ancestry GWAS of 11,090 cases, 18,316 controls. **d)** Meta-analysis of the three ancestries identified 52 GWS loci.

**Fig. S2 Ancestry-stratified gene-based analyses of SNHL in the MVP.**

Manhattan plots of gene-based analysis of sensorineural hearing loss (SNHL) conducted within each ancestry strata of the MVP. The red line indicates the gene-wide significance threshold at p < 2.66 × 10−6 (Bonferroni correction for 18,809 genes tested). A selection of significant genes have been labeled. Significant genes that are also listed in HereditaryHearingLoss.org have been labeled in bold font. a) European ancestry (EUA) gene-based analysis of 176,393 cases and 172,117 controls identified 62 significant genes. b) African ancestry (AFA) gene-based analysis of 22,757 cases and 74,842 controls identified 2 significant genes. c) Indigenous American ancestry (IAA) gene-based analysis of 11,090 cases, 18,316 controls.

**Fig. S3 Gene-based analyses of the MVP multi-ancestry meta-analysis and the UKB SNHL GWAS.**

Manhattan plots of gene-based analysis of sensorineural hearing loss (SNHL). The red line indicates the gene-wide significance threshold at p < 2.66 × 10−6 (Bonferroni correction for 18,809 genes tested). A selection of significant genes have been labeled. Significant genes that are also listed in HereditaryHearingLoss.org have been labeled in bold. **a)** Gene-based analysis of the MVP multi-ancestry meta-analysis (210,240 cases and 265,275 controls) identified 64 significant genes. **b)** UKB gene-based analysis of 87,056 cases and 163,333 controls identified 97 significant genes. The X chromosome was not included in the UKB analysis.

**Fig. S4 Gene-tissue expression analyses of SNHL based on human data.**

MAGMA gene-tissue expression analyses for adult inner ear and GTEx v8 tissues in sensorineural hearing loss (SNHL), conducted in the MVP European ancestry (EUA), UKB, and meta-analysis of the MVP EUA and UKB. Bars denote -log10 p-values. Dotted lines indicate significance after Bonferroni-adjustment for the number of tissues tested (panel a, p < 1.61 × 10−3 for 31 general tissue types tested; panel b, p < 7.04 × 10−4 for 71 specific tissues tested). Bonferroni significant tissue associations are also indicated through red shading of bars. **a)** Gene-tissue analysis of 31 general tissue types indicates a significant enrichment of the inner ear in all three analyses, as well as the nerve in MVP EUA, and both the nerve and pituitary in the meta-analysis. **b)** Among the 71 specific tissues, 13 cochlear and vestibular cell types were significant in the meta-analysis, including 8 in UKB and 10 in MVP EUA. The cerebellum and cerebellar hemisphere are significant in both UKB and the meta-analysis (note: cerebellar hemisphere and cerebellum are the same tissue, with different RNA preservation after death).

**Fig. S5 Cochlear cell type enrichment analyses based on mouse data.**

**a)** Cross-section of the Organ of Corti. The Organ of Corti is supported by the basilar membrane (BM) below and movement is restricted by gelatinous tectorial membrane (TM) above. **b)** Cochlear cell type enrichment analyses based on mouse data (Boussaty et al., 2023) depicts results of conditional MAGMA gene-property analyses for 58 different cell types. Bars denote -log10 p-values. Dotted line indicates p < 0.05. Solid line indicates significance after Bonferroni-adjustment for multiple comparisons (p < 8.62 × 10−4 for 58 cell types tested).

**Fig. S5 Polygenic Risk Scores (PRS).**

Genetic risk score (PRS) predictions for SNHL comparing different training and target data. The *y* axis represents SNHL odds ratios relative to the lowest quintile of PRS. Cross-dataset predictions from the EUA MVP to UKB (blue circles) and UKB to EUA MVP (red circles) indicate similar prediction accuracies.

**VA Million Veteran Program:**

**Core Acknowledgements for Publications**

**May 2024**

**MVP Program Office**

Sumitra Muralidhar, Ph.D., Program Director

US Department of Veterans Affairs, 810 Vermont Avenue NW, Washington, DC 20420

Jennifer Moser, Ph.D., Associate Director, Scientific Programs

US Department of Veterans Affairs, 810 Vermont Avenue NW, Washington, DC 20420

Jennifer E. Deen, B.S., Associate Director, Cohort & Public Relations

**US Department of Veterans Affairs, 810 Vermont Avenue NW, Washington, DC 20420**

MVP Executive Committee

Co-Chair: Philip S. Tsao, Ph.D.

VA Palo Alto Health Care System, 3801 Miranda Avenue, Palo Alto, CA 94304

Co-Chair: Sumitra Muralidhar, Ph.D.

US Department of Veterans Affairs, 810 Vermont Avenue NW, Washington, DC 20420

J. Michael Gaziano, M.D., M.P.H.

VA Boston Healthcare System, 150 S. Huntington Avenue, Boston, MA 02130

Elizabeth Hauser, Ph.D.

Durham VA Medical Center, 508 Fulton Street, Durham, NC 27705

Amy Kilbourne, Ph.D., M.P.H.

VA HSR&D, 2215 Fuller Road, Ann Arbor, MI 48105

Michael Matheny, M.D., M.S., M.P.H.

VA Tennessee Valley Healthcare System, 1310 24th Ave. South, Nashville, TN 37212

Dave Oslin, M.D.

Philadelphia VA Medical Center, 3900 Woodland Avenue, Philadelphia, PA 19104

Deepak Voora, MD

Durham VA Medical Center, 508 Fulton Street, Durham, NC 27705

MVP Co-Principal Investigators

J. Michael Gaziano, M.D., M.P.H.

VA Boston Healthcare System, 150 S. Huntington Avenue, Boston, MA 02130

Philip S. Tsao, Ph.D.

VA Palo Alto Health Care System, 3801 Miranda Avenue, Palo Alto, CA 94304

MVP Core Operations

Jessica V. Brewer, M.P.H., Director, MVP Cohort Operations

VA Boston Healthcare System, 150 S. Huntington Avenue, Boston, MA 02130

Mary T. Brophy M.D., M.P.H., Director, VA Central Biorepository

VA Boston Healthcare System, 150 S. Huntington Avenue, Boston, MA 02130

Kelly Cho, M.P.H, Ph.D., Director, MVP Phenomics

MVP Core Acknowledgements for Publications_May 2024

VA Boston Healthcare System, 150 S. Huntington Avenue, Boston, MA 02130

Lori Churby, B.S., Director, MVP Regulatory Affairs

VA Palo Alto Health Care System, 3801 Miranda Avenue, Palo Alto, CA 94304

Scott L. DuVall, Ph.D., Director, VA Informatics and Computing Infrastructure (VINCI)

VA Salt Lake City Health Care System, 500 Foothill Drive, Salt Lake City, UT 84148

Saiju Pyarajan Ph.D., Director, Data and Computational Sciences

VA Boston Healthcare System, 150 S. Huntington Avenue, Boston, MA 02130

Robert Ringer, Pharm.D., Director, VA Albuquerque Central Biorepository

New Mexico VA Health Care System, 1501 San Pedro Drive SE, Albuquerque, NM 87108

Luis E. Selva, Ph.D., Director, MVP Biorepository Coordination

VA Boston Healthcare System, 150 S. Huntington Avenue, Boston, MA 02130

Shahpoor (Alex) Shayan, M.S., Director, MVP PRE Informatics

VA Boston Healthcare System, 150 S. Huntington Avenue, Boston, MA 02130

Brady Stephens, M.S., Principal Investigator, MVP Information Center

Canandaigua VA Medical Center, 400 Fort Hill Avenue, Canandaigua, NY 14424

Stacey B. Whitbourne, Ph.D., Director, MVP Cohort Development and Management

VA Boston Healthcare System, 150 S. Huntington Avenue, Boston, MA 02130

MVP Publications and Presentations Committee

Co-Chair: Themistocles L. Assimes, M.D., Ph. D

VA Palo Alto Health Care System, 3801 Miranda Avenue, Palo Alto, CA 94304

Co-Chair: Adriana Hung, M.D.; M.P.H

VA Tennessee Valley Healthcare System, 1310 24th Ave. South, Nashville, TN 37212

Co-Chair: Henry Kranzler, M.D.

Philadelphia VA Medical Center, 3900 Woodland Avenue, Philadelphia, PA 1910
