# Supplementary material for: Polygenic Contribution to Sensorineural Hearing Loss Implicates Novel Risk Loci and Convergence with Congenital Hearing Loss Genes
Source: J Assoc Res Otolaryngol. 2026 Mar 16;27(3):447–63. doi: 10.1007/s10162-026-01044-0 (PMC13237364; doi:10.1007/s10162-026-01044-0)

**Supplementary Data 3**

# Genome-wide association study meta-analysis brings monogenic hearing loss genes into the polygenic realm

Clifford Royce*[1,2], Johnson Jacquelyn[1,3], Mackey Caroline E[1,3], Mikita Elizabeth A[1,3], Ryan Allen F[1,2], Million Veteran Program, Maihofer Adam X[1,3], Nievergelt Caroline M*[1,3]

[1]Veterans Affairs San Diego Healthcare System, Research Service, San Diego, CA, USA
[2]University of California San Diego, Department of Otolaryngology – Head and Neck Surgery, La Jolla, CA, USA
[3]University of California San Diego, Department of Psychiatry, La Jolla, CA, US

*Corresponding authors: Nievergelt (cnievergelt@health.ucsd.edu) and Clifford (reclifford@health.ucsd.edu)

Regional association plots of each significant locus from GWAS. Loci are indexed by the values provided in other manuscript tables. Every circle represents a genetic variant tested in GWAS. Variants are colored according to their estimated LD with the labeled index SNP (color bar on side of plot indicates color coding of r2 values). The LD r2 values were taken from 1000 Genomes Project Phase 3 (1KGP3) subpopulations. For a given GWAS, the subpopulation selected for LD was based on correspondence between 1KGP3 and GWAS: 1KGP AFR samples are used for the African ancestry (AFA) GWAS. 1KGP AMR samples are used for the Indigenous American (IAA) GWAS. 1KGP EUR samples are used for European ancestry (EUA) GWAS and for the meta-analyses. The x-axis represents the genetic position (in base pairs, build hg19). The lefthand y-axis represents –log10 p- value from GWAS. Local estimates of recombination rate are indicated in light blue (legend on righthand y-axis). Gene names, strands, and boundaries are shown in the box below the regional plot. a) Meta- analysis of MVP multi-ancestry and UKBB. b) MVP multi-ancestry GWAS. c) UKB GWAS. d) MVP IAA GWAS. e) MVP AFA GWAS. f) MVP EUA GWAS.

# locus001 | rs112725535

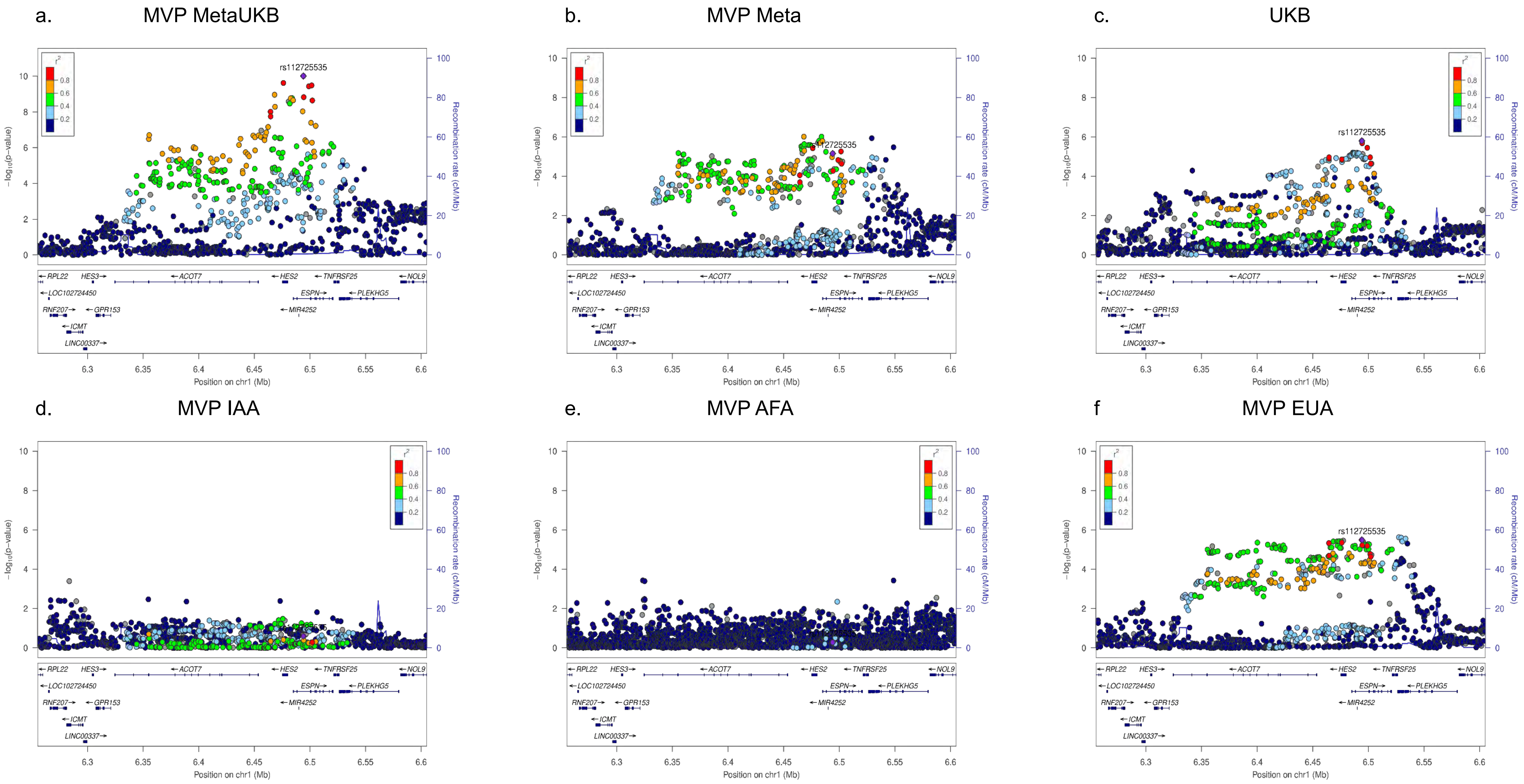

# locus002 | rs2863455

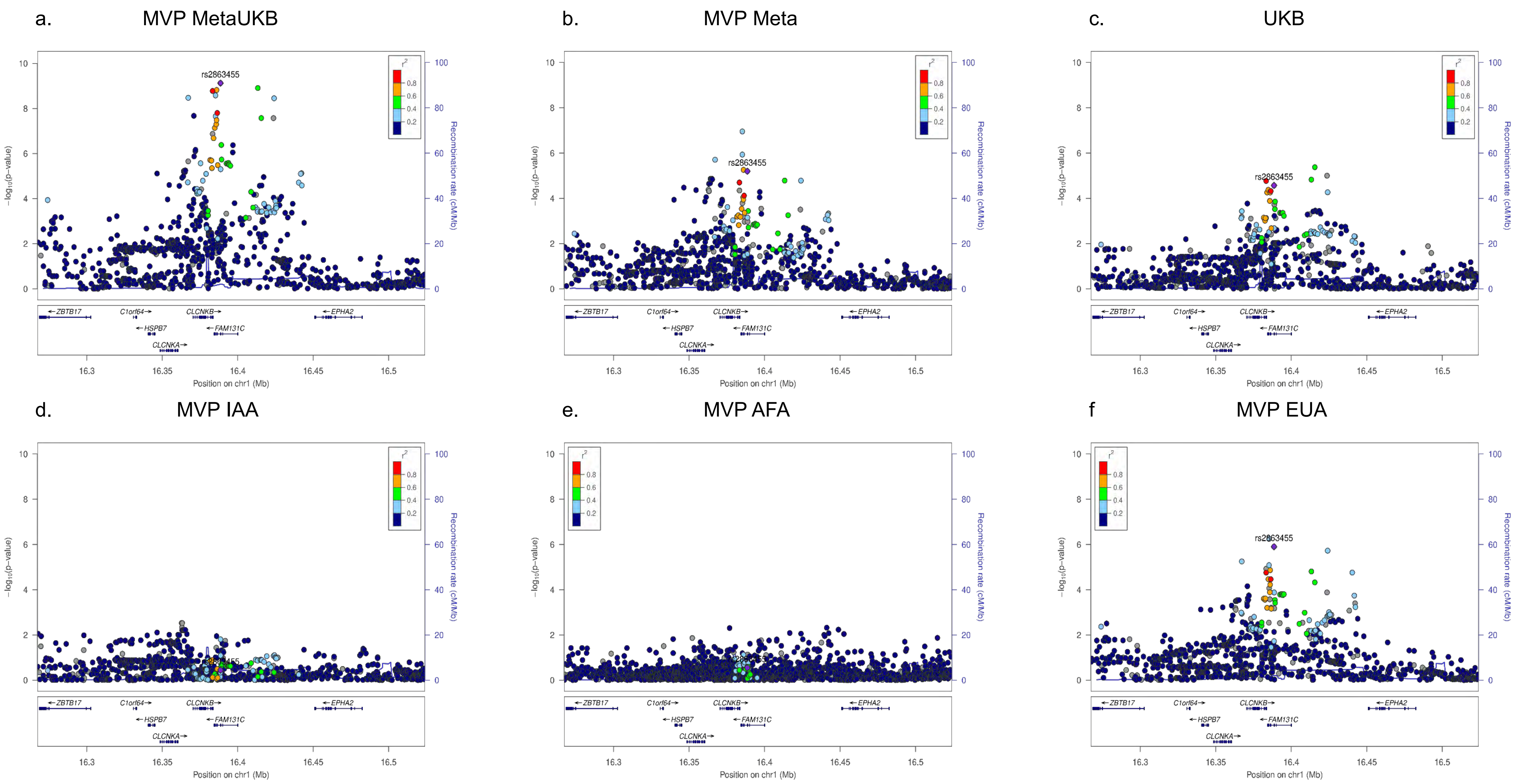

# locus003 | rs2281179

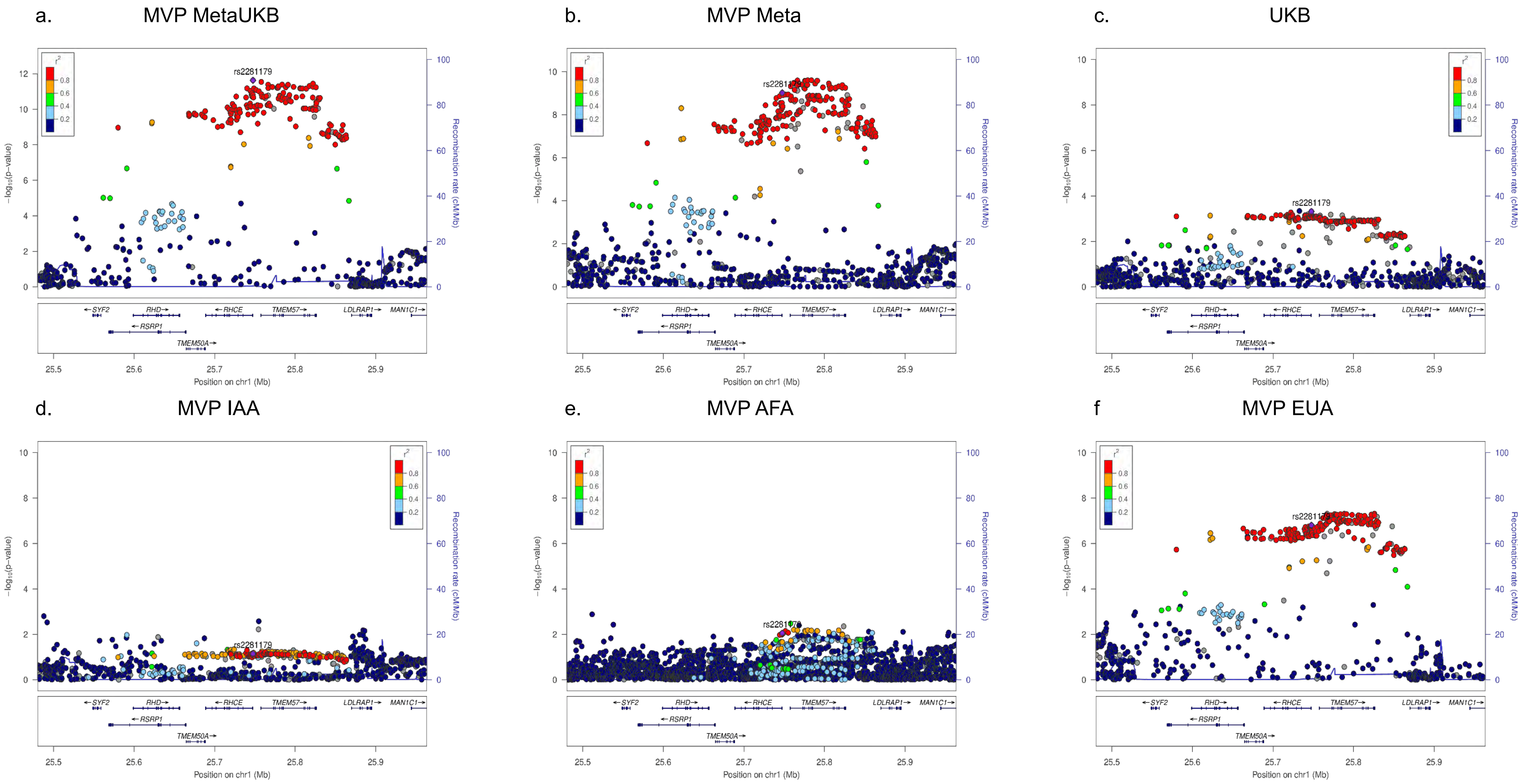

# locus003 | rs34997029

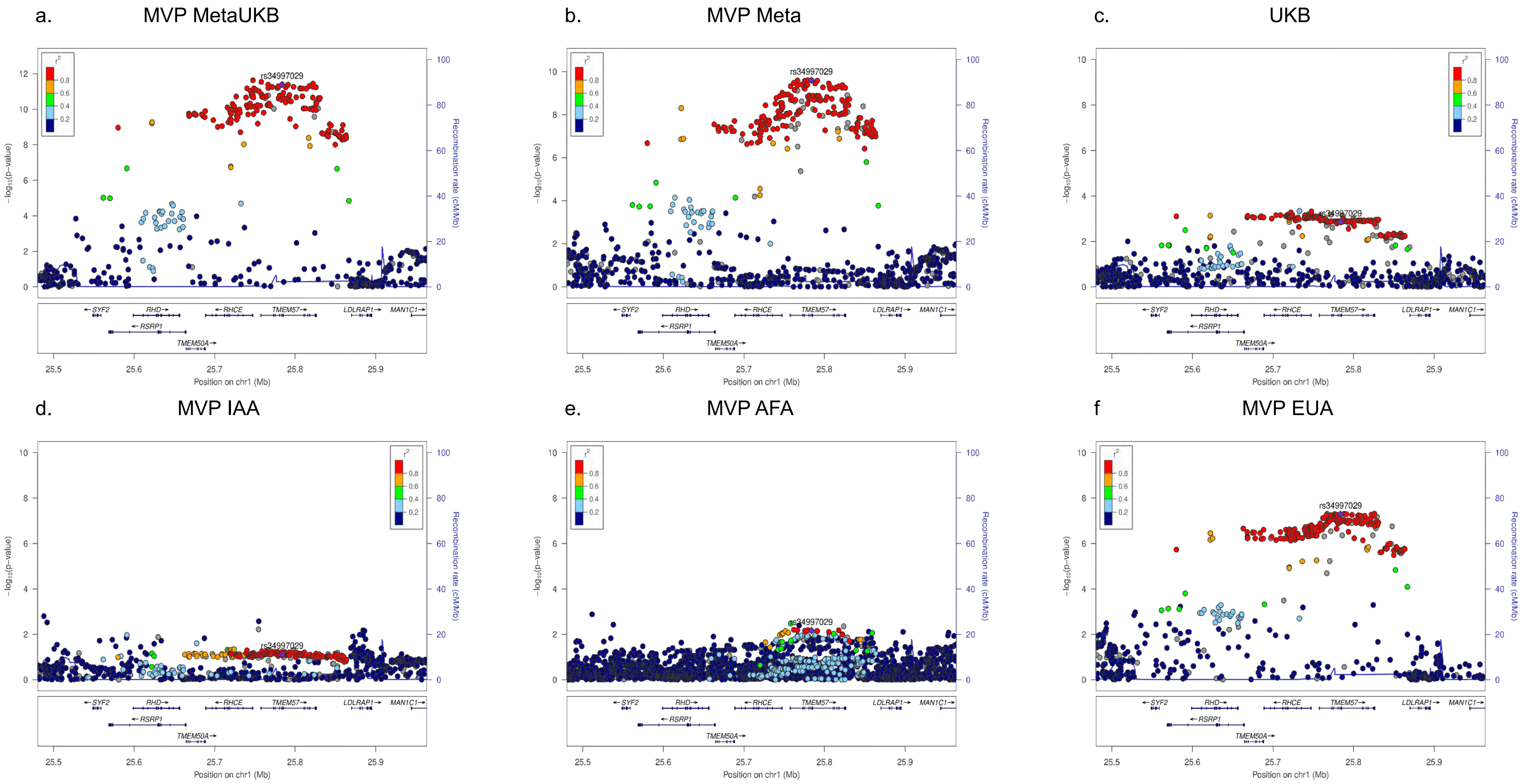

# locus003 | rs67125653

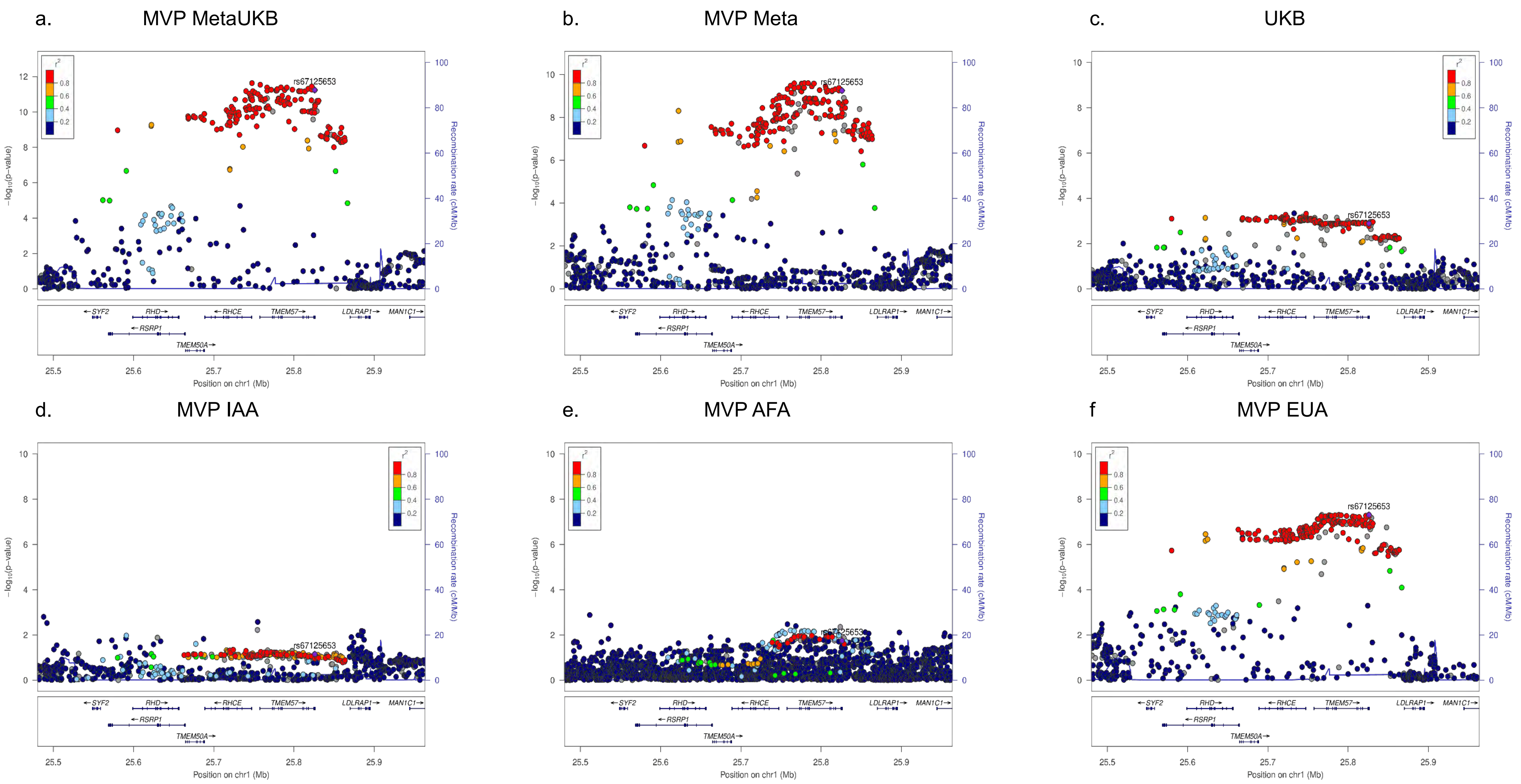

# locus004 | rs201377643

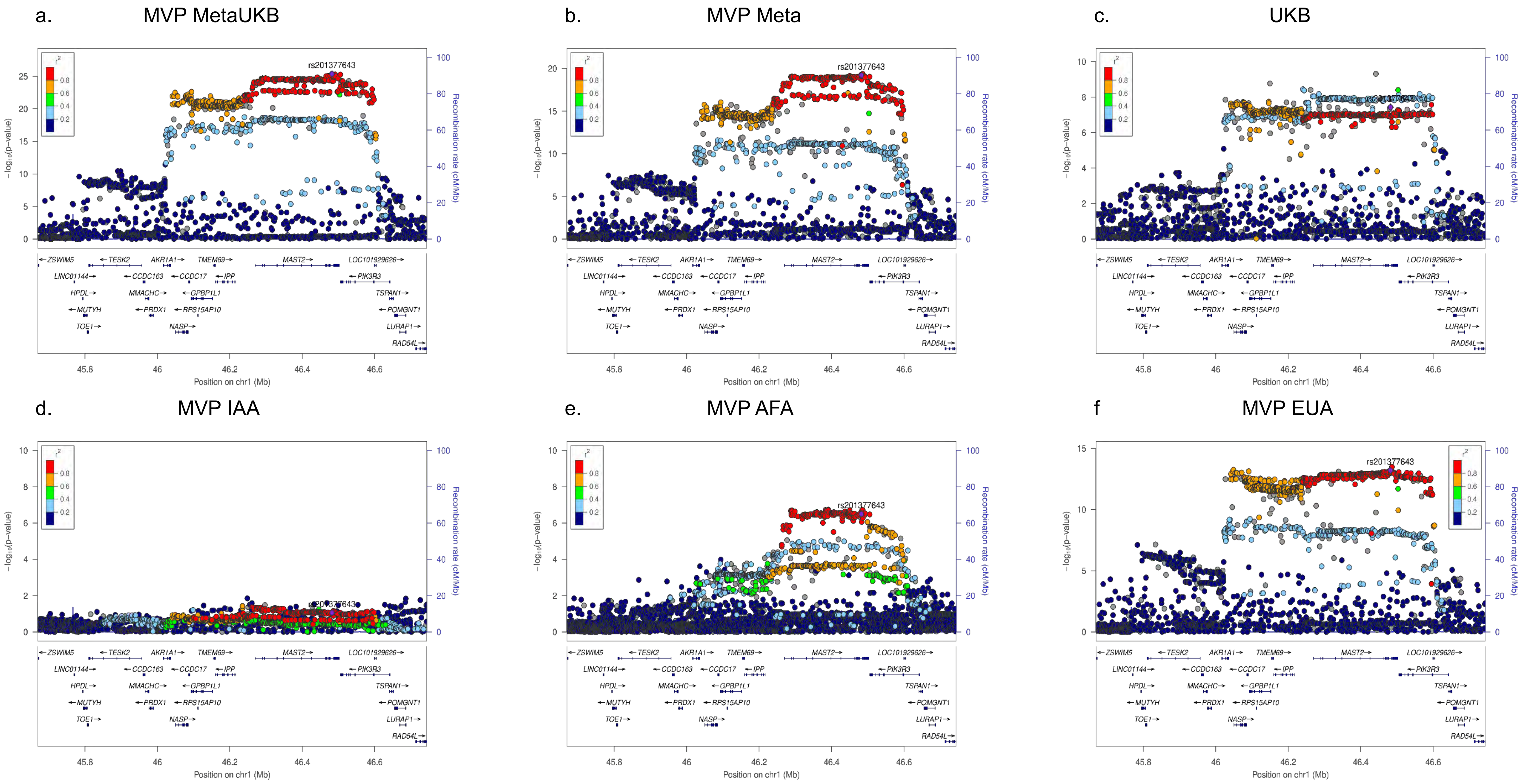

# locus004 | rs946527

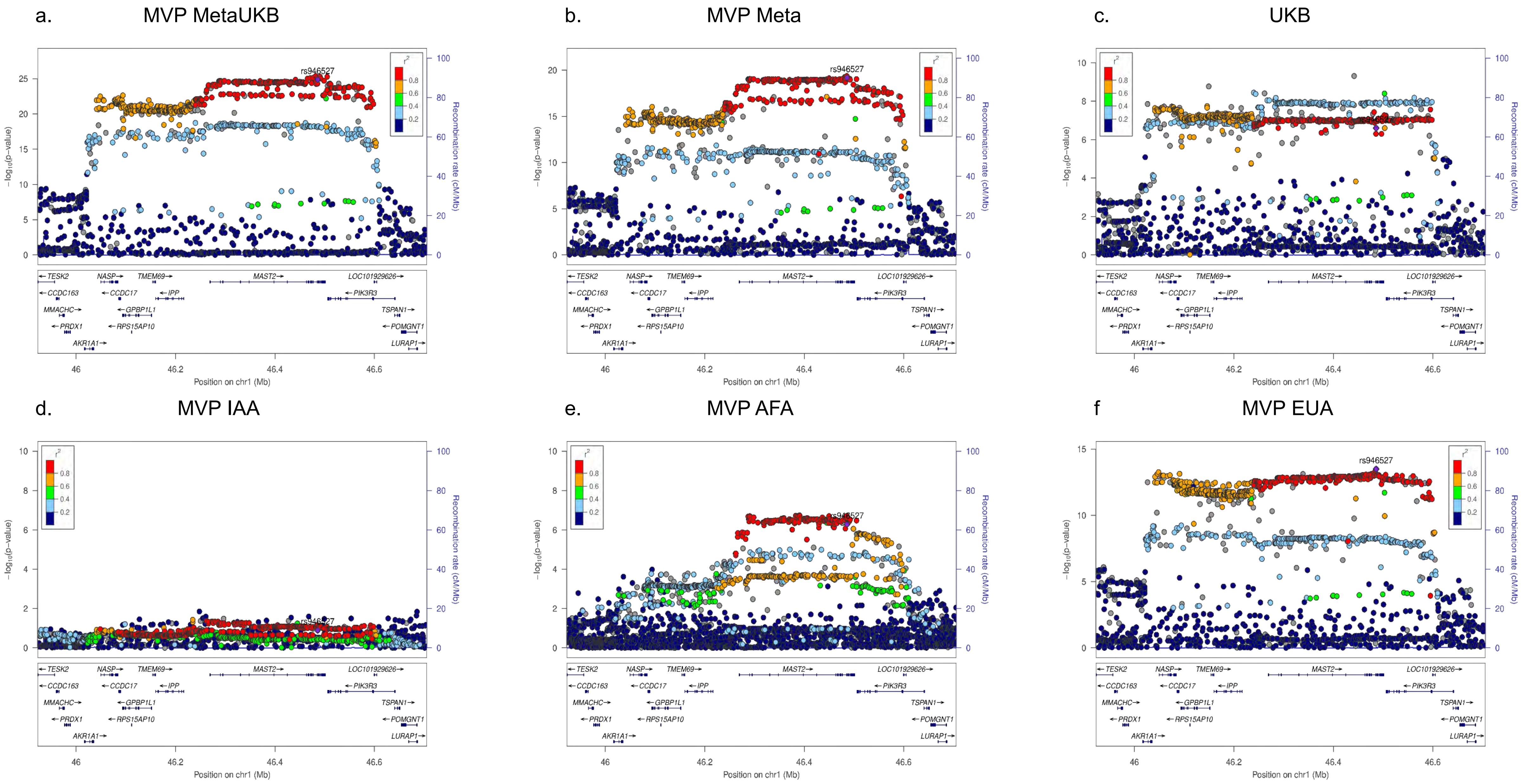

# locus004 | rs946528

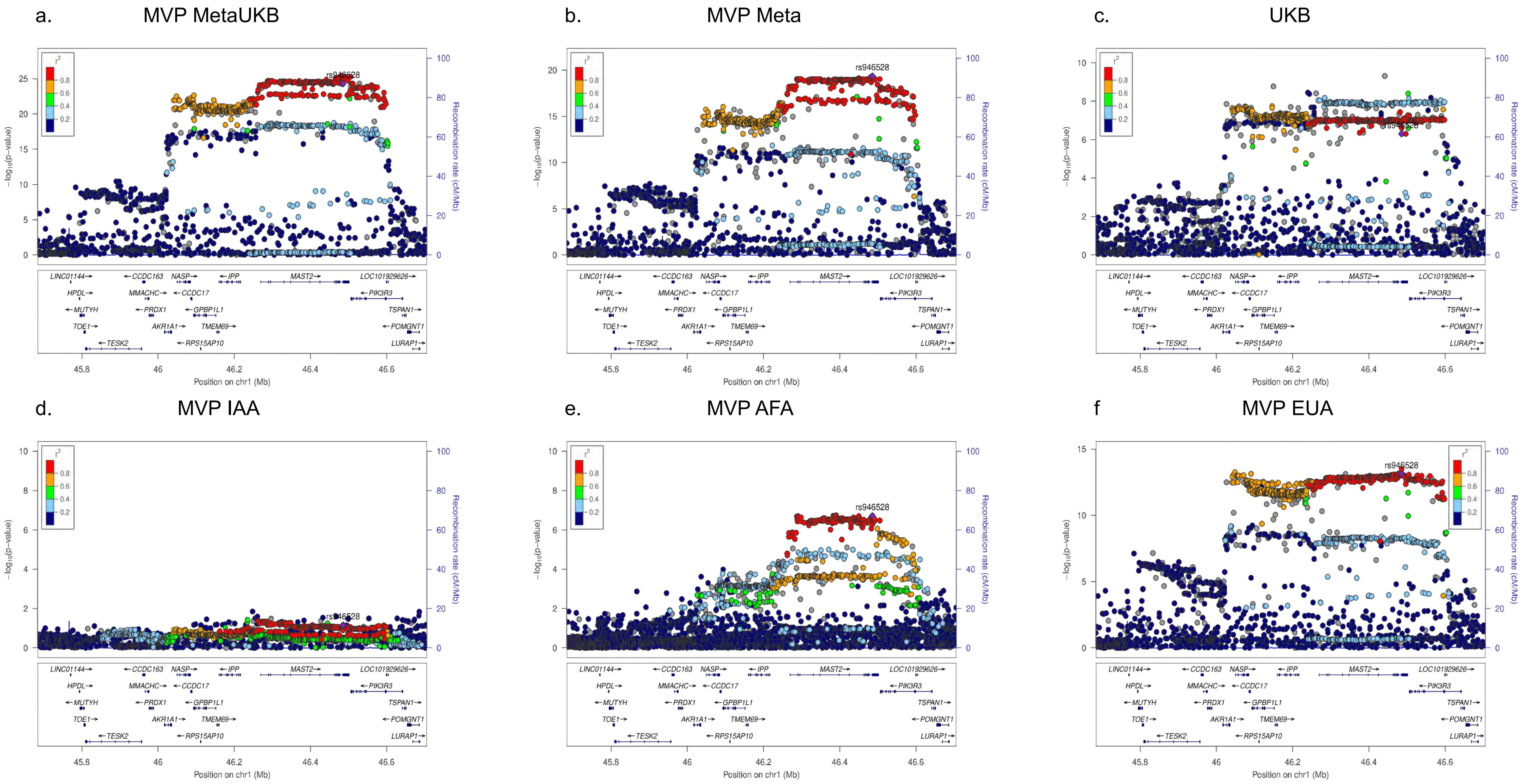

# locus005 | rs2180201

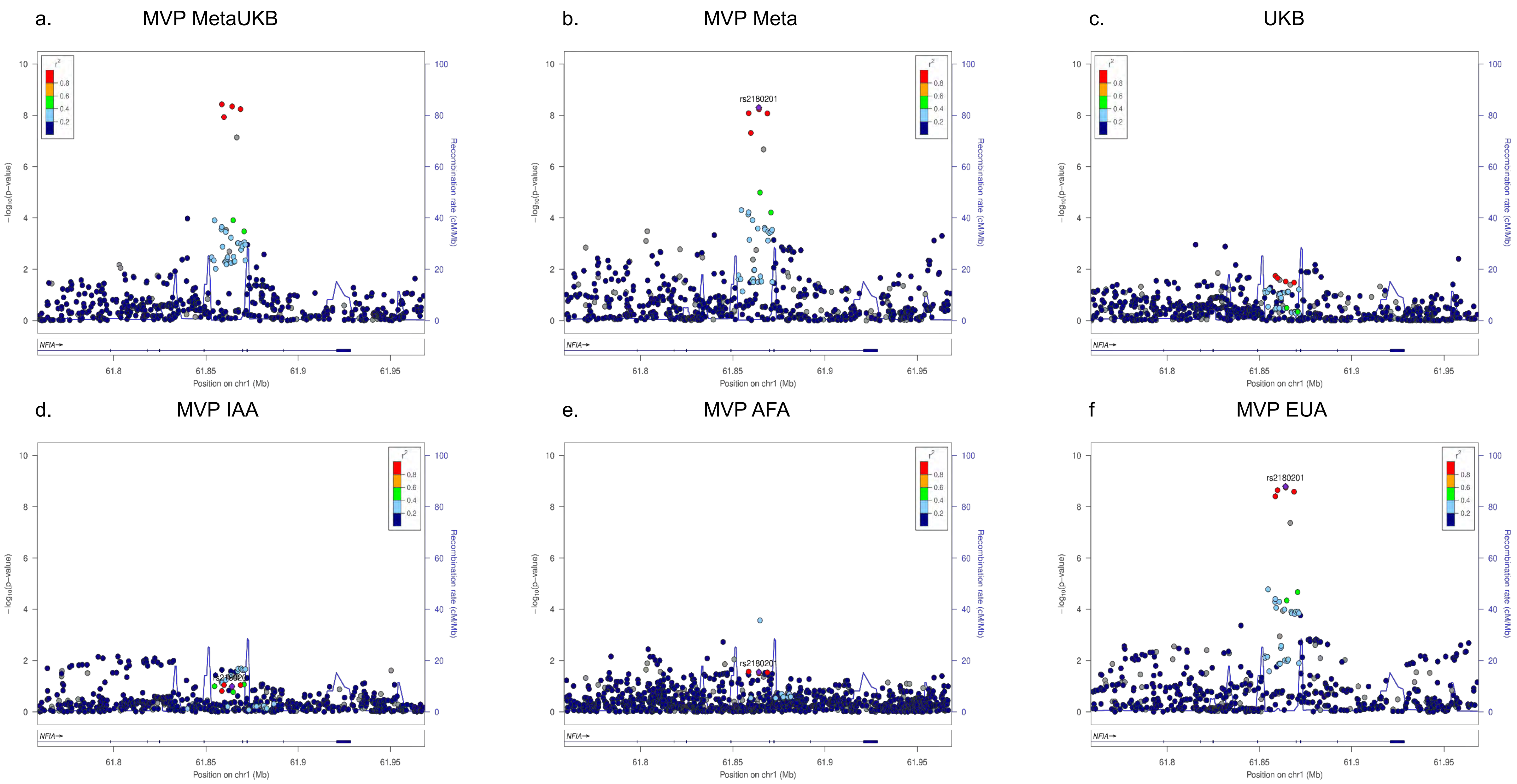

# locus005 | rs2499507

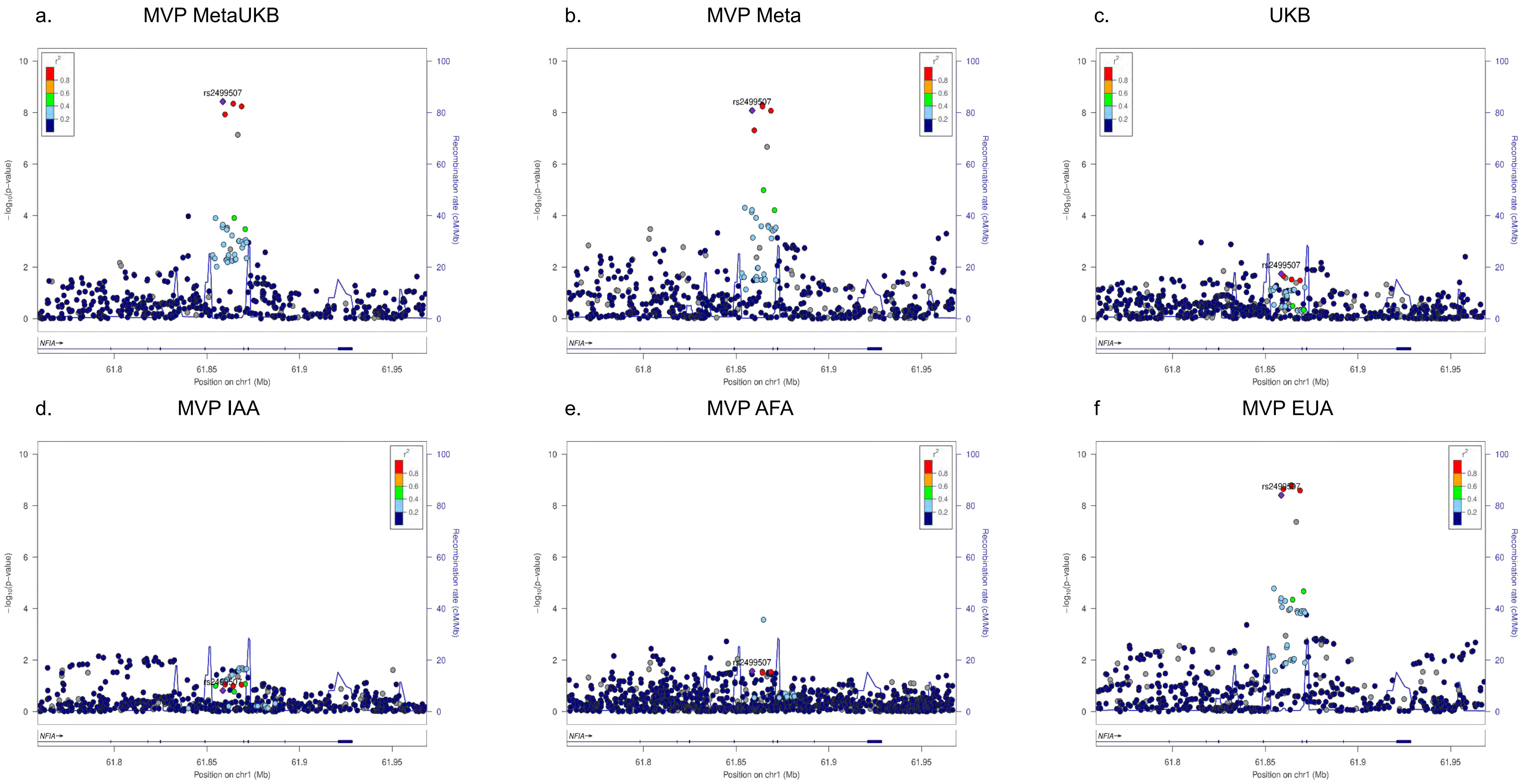

# locus006 | rs61789075

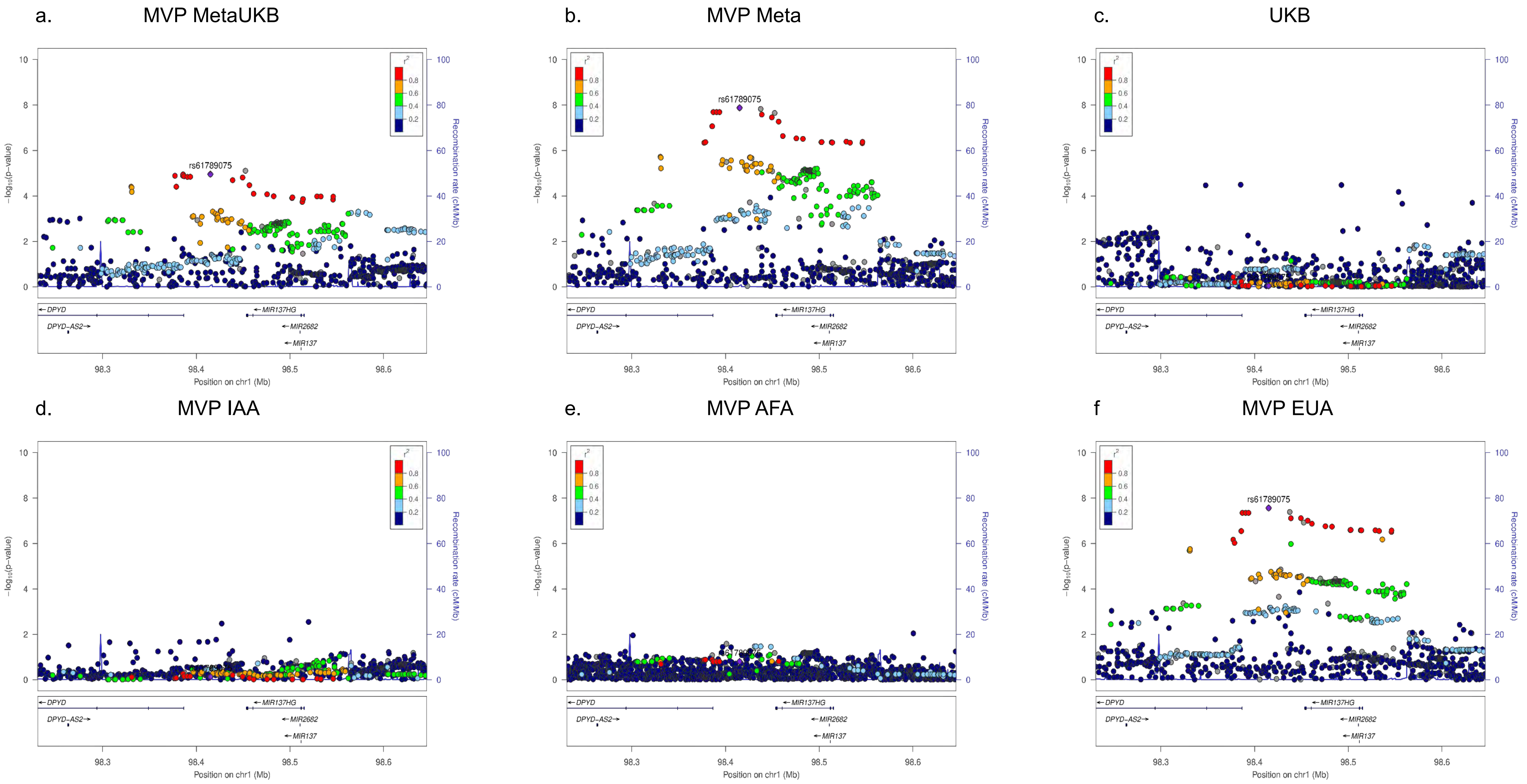

# locus007 | rs12740738

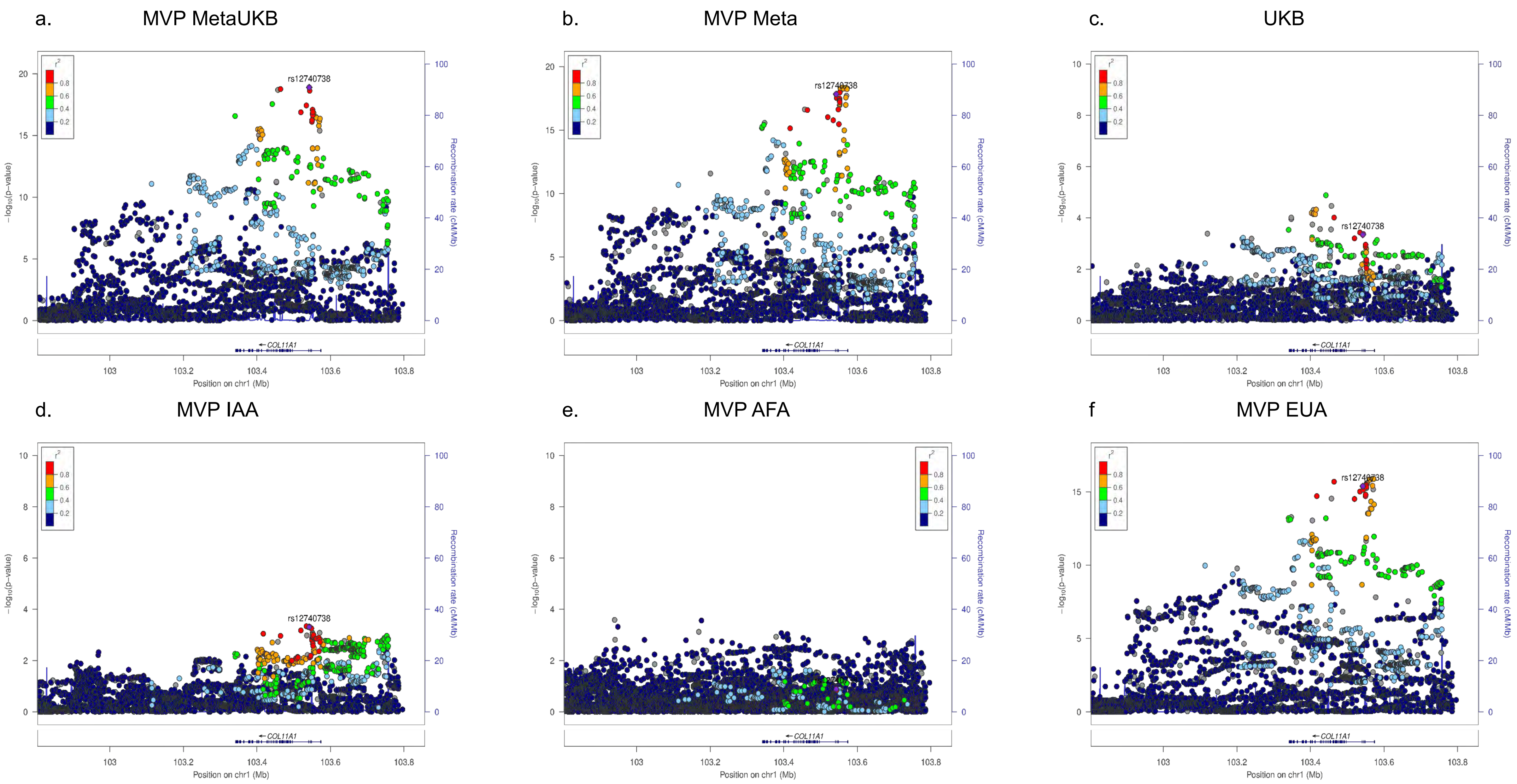

# locus007 | rs2376280

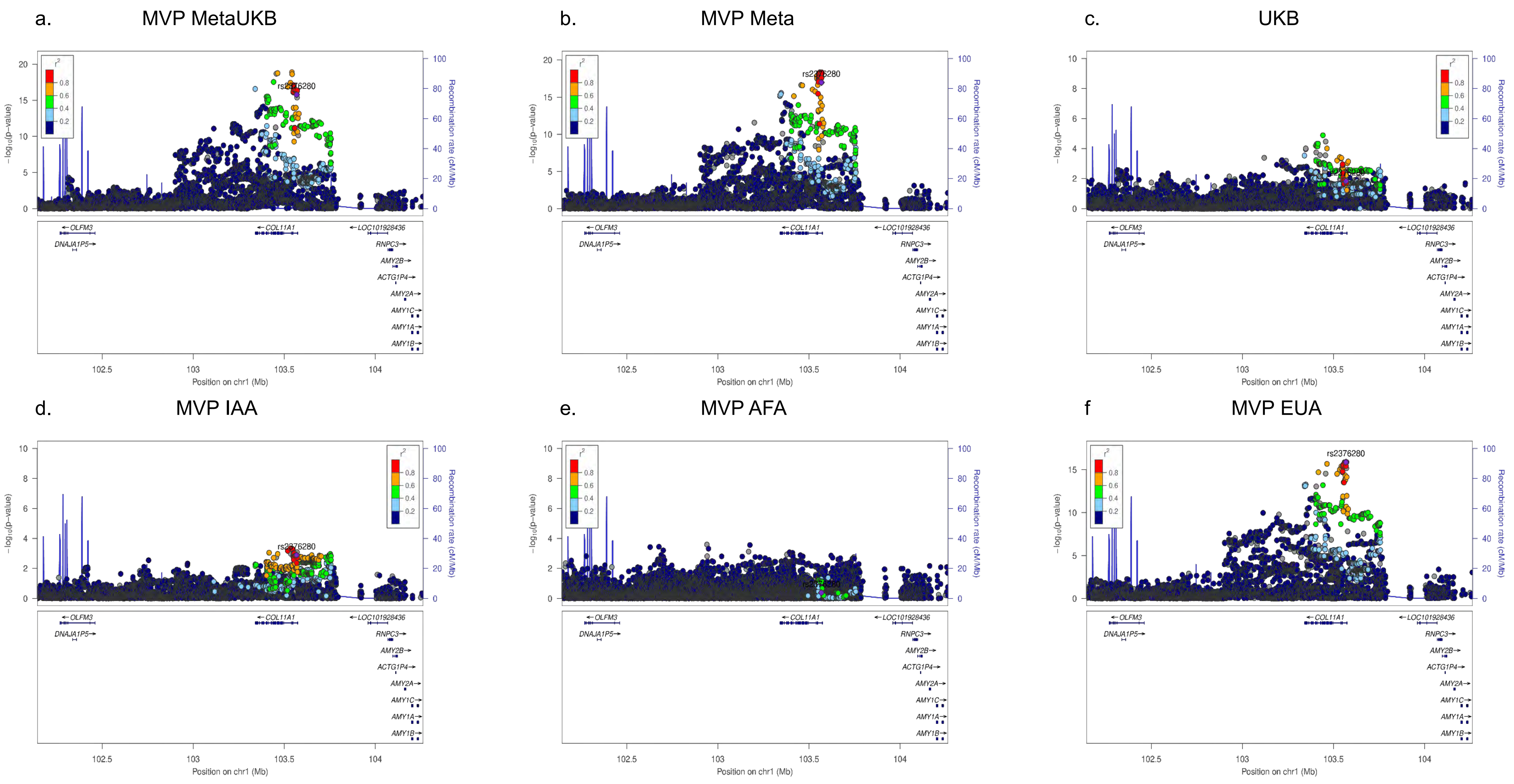

# locus007 | rs9970114

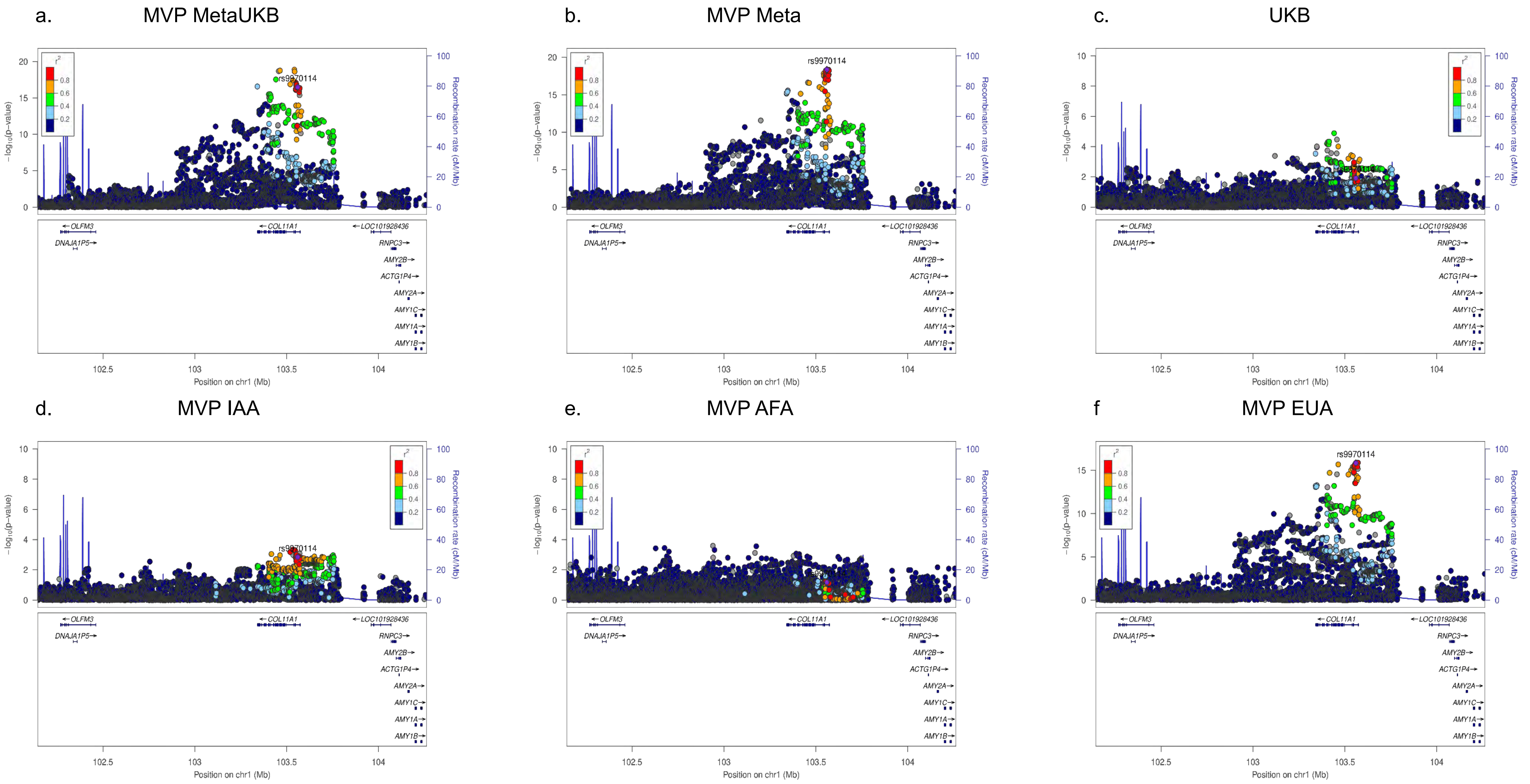

# locus008 | rs4657403

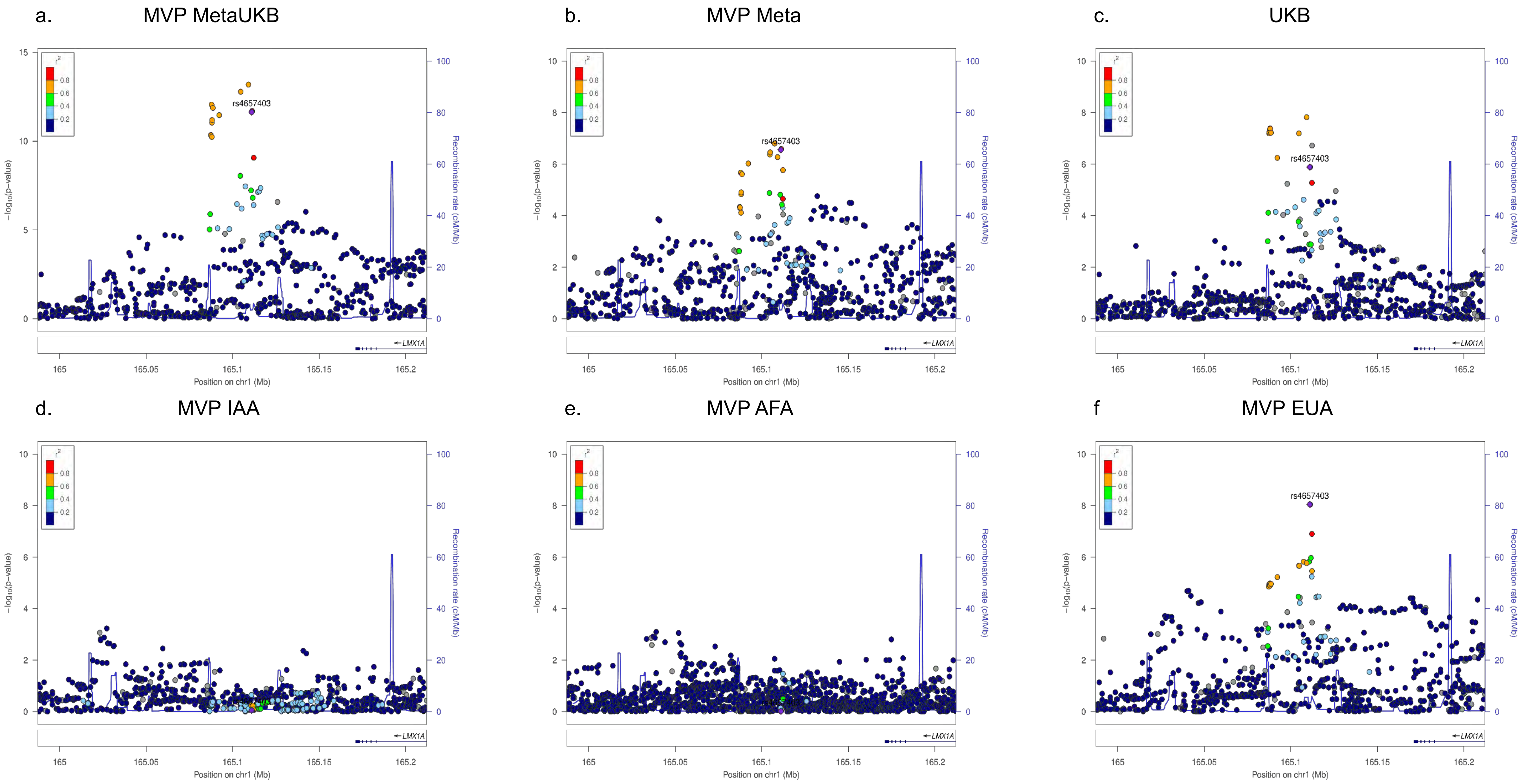

# locus008 | rs7525101

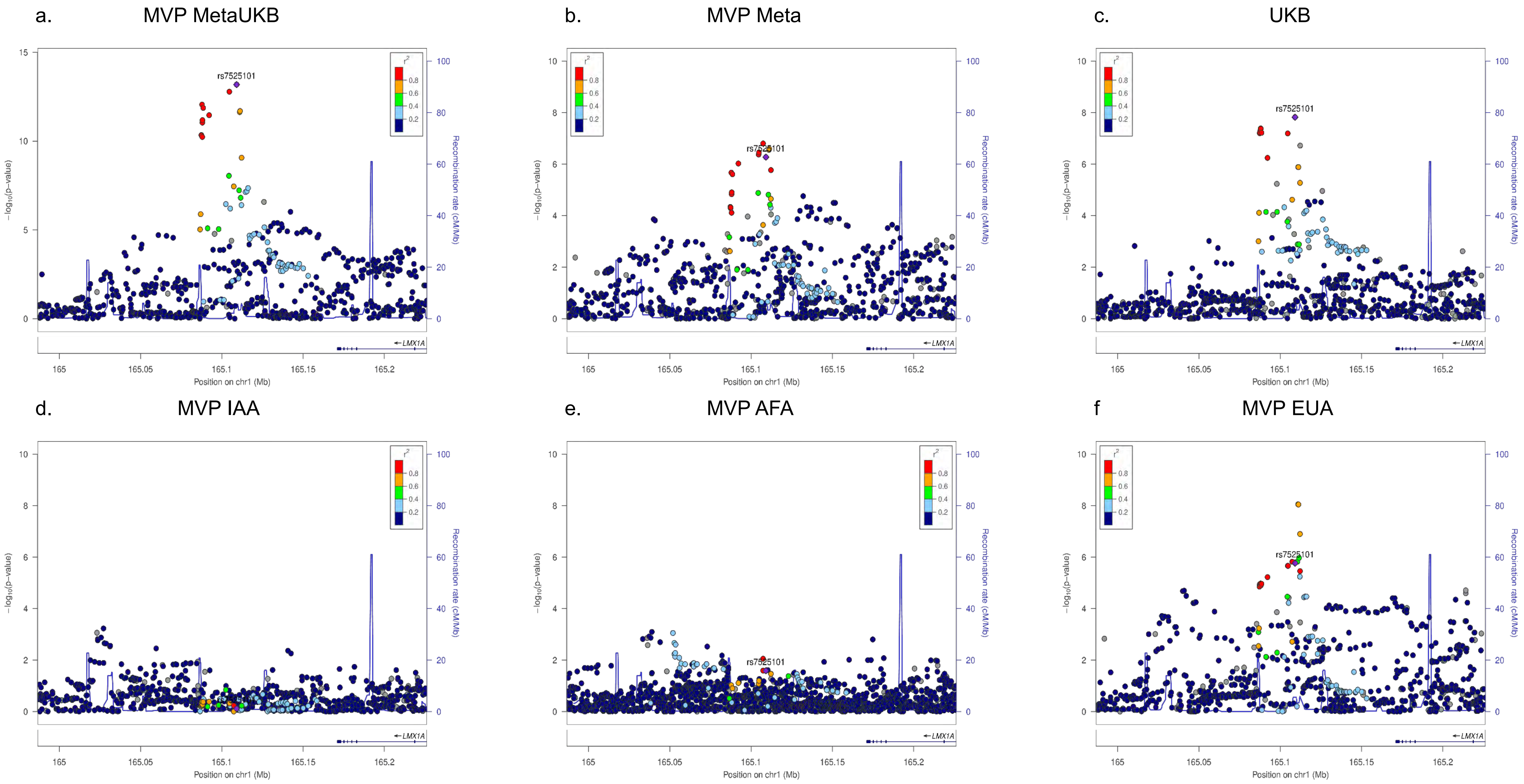

# locus009 | rs7532898

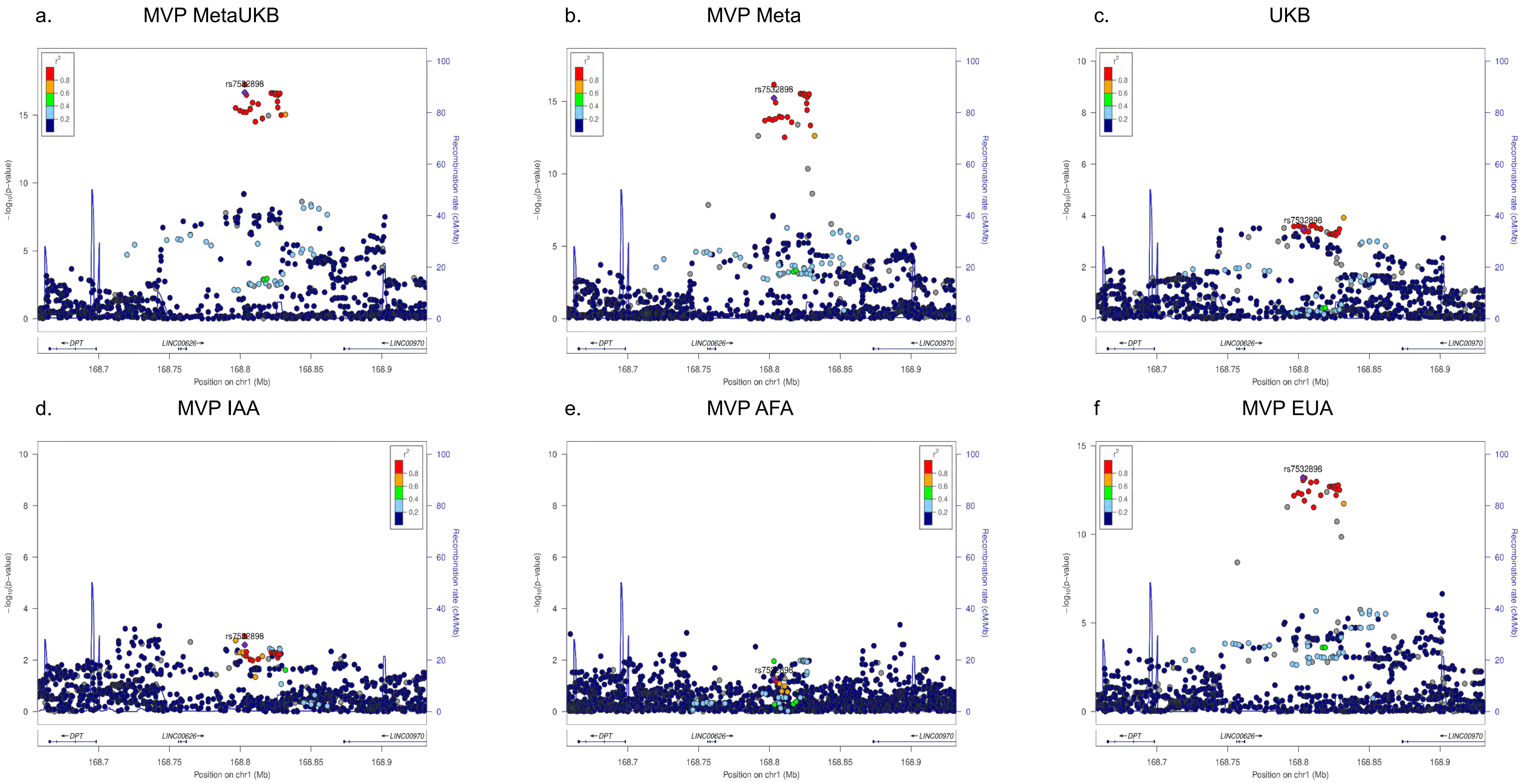

locus009 | rs7555365

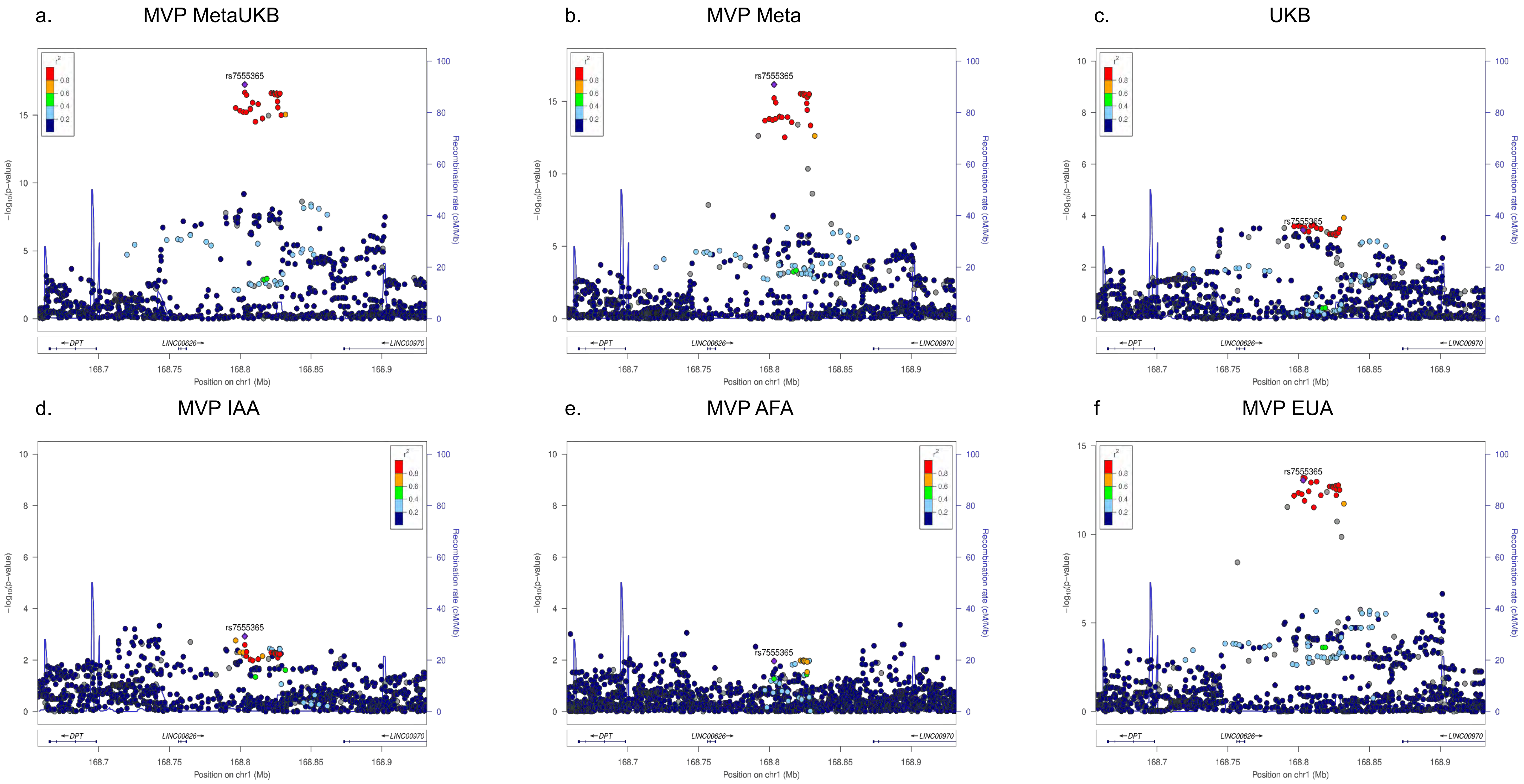

# locus010 | rs10919329

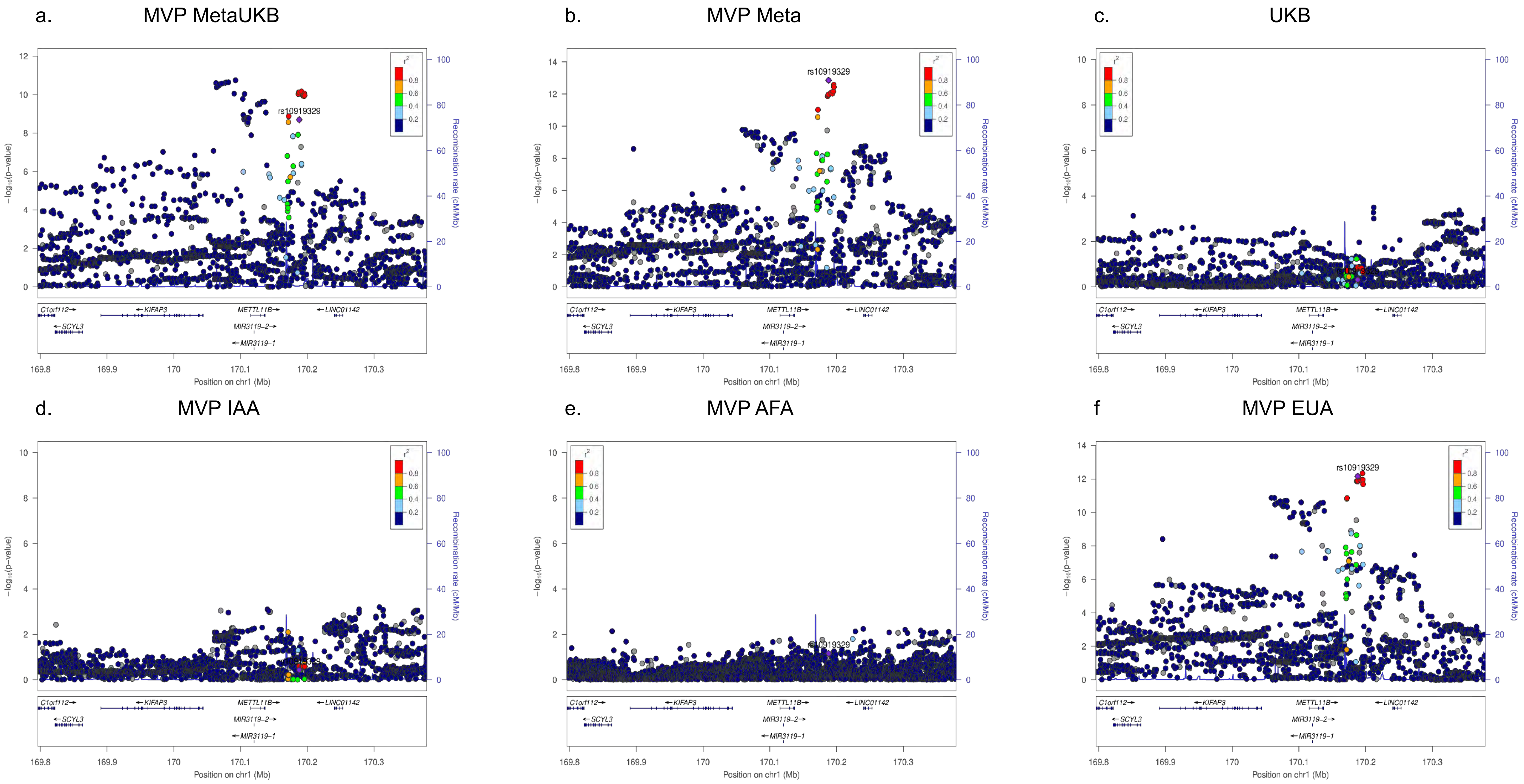

# locus010 | rs4399218

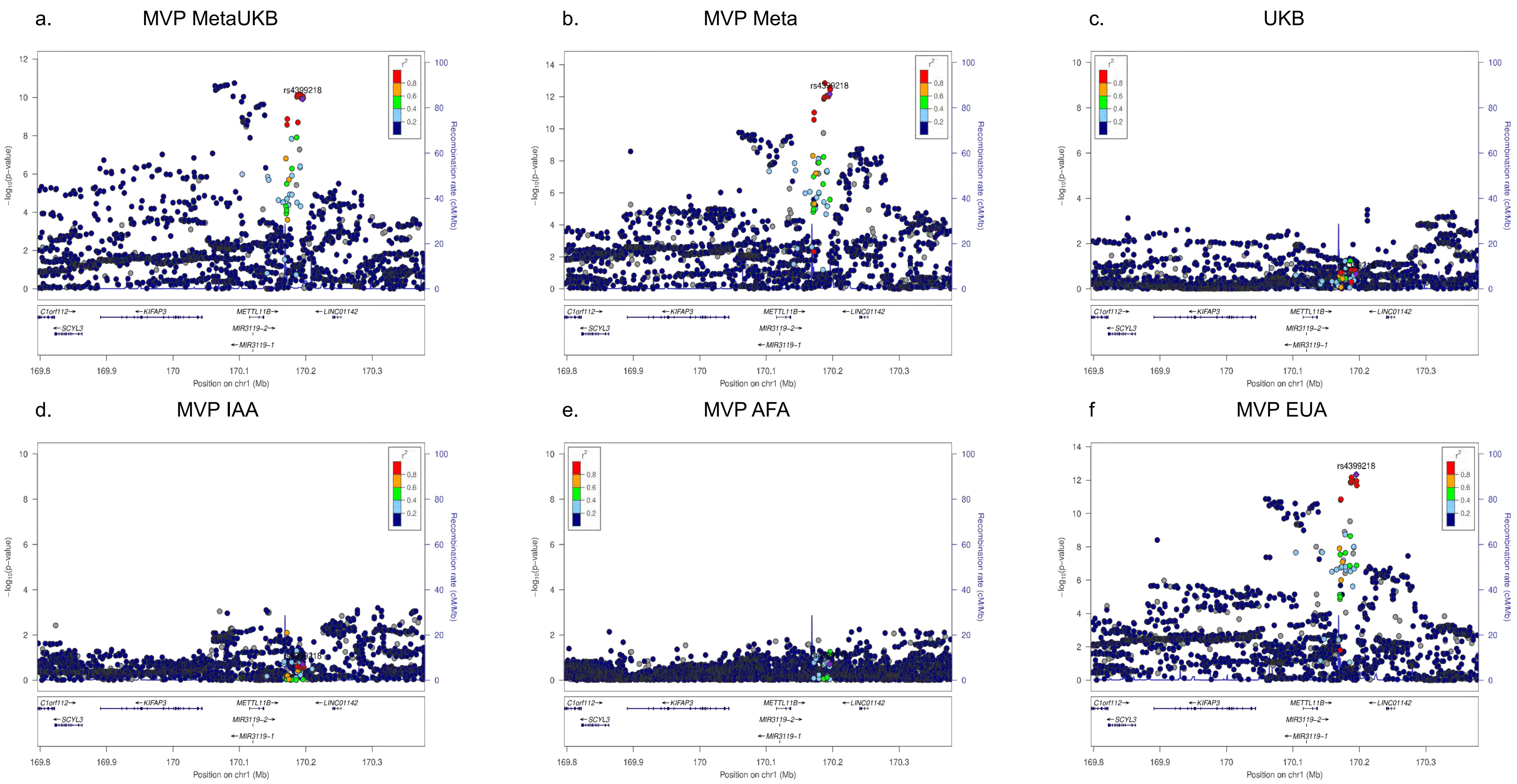

# locus010 | rs501925

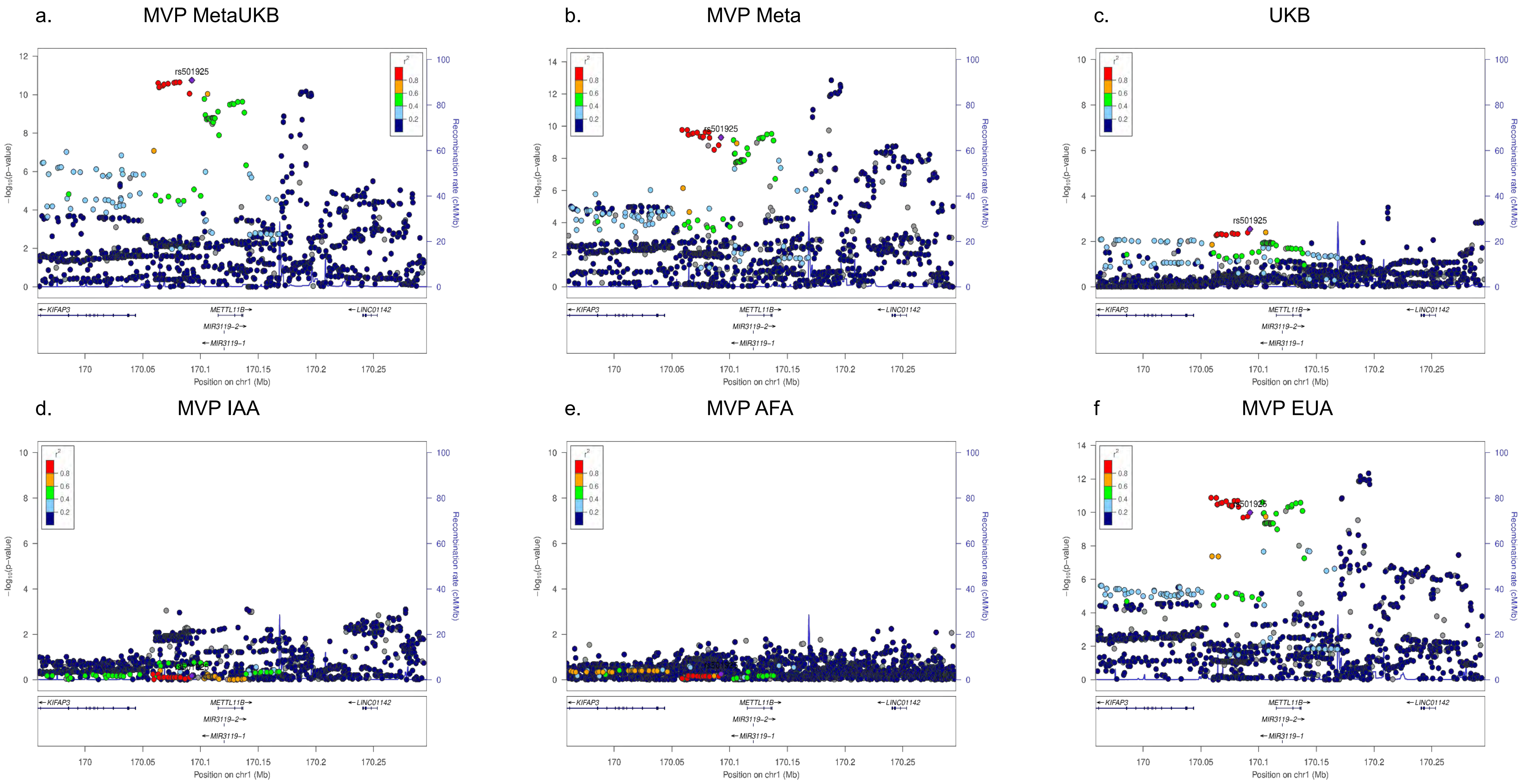

# locus011 | rs823116

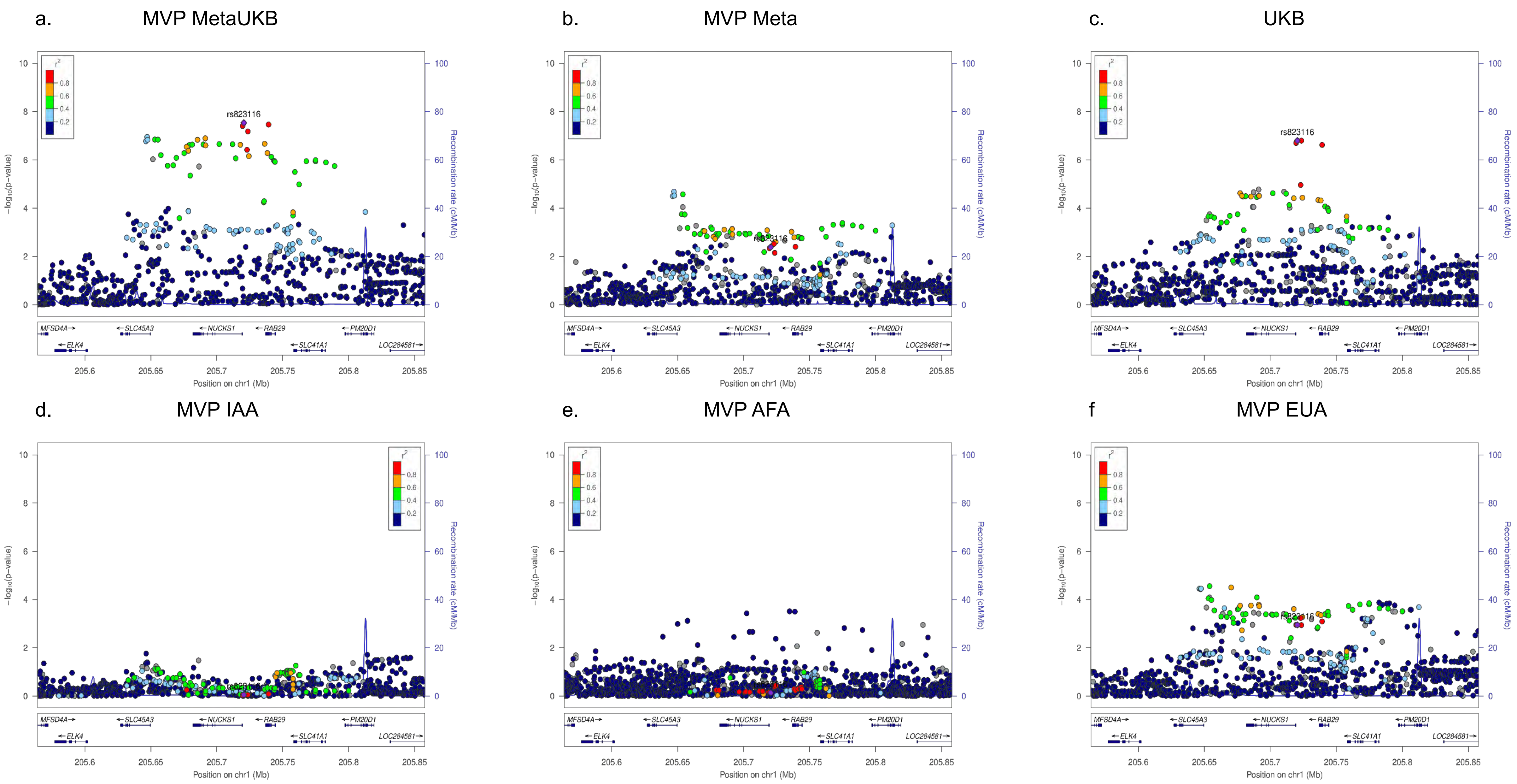

# locus012 | rs10927035

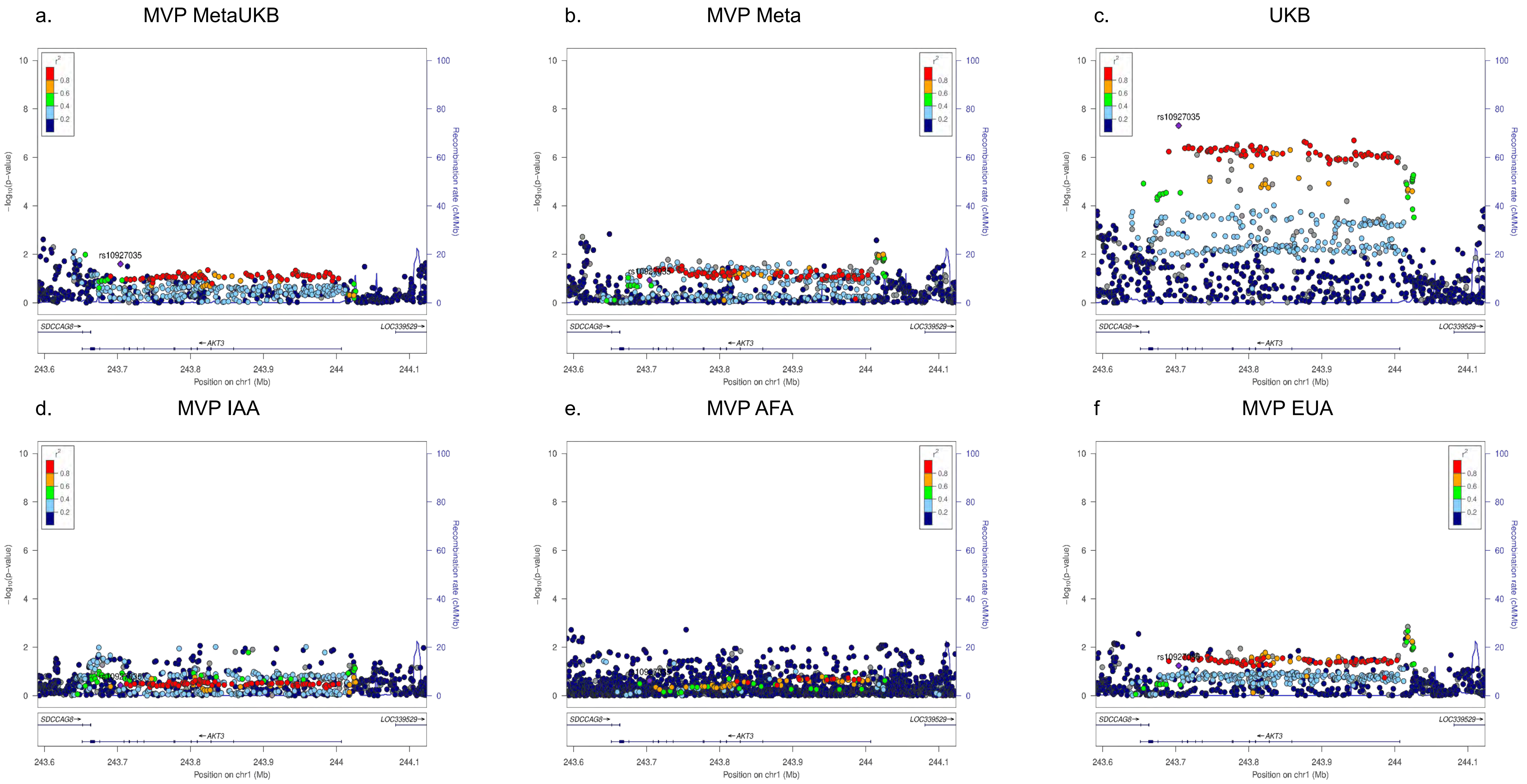

# locus013 | rs67278917

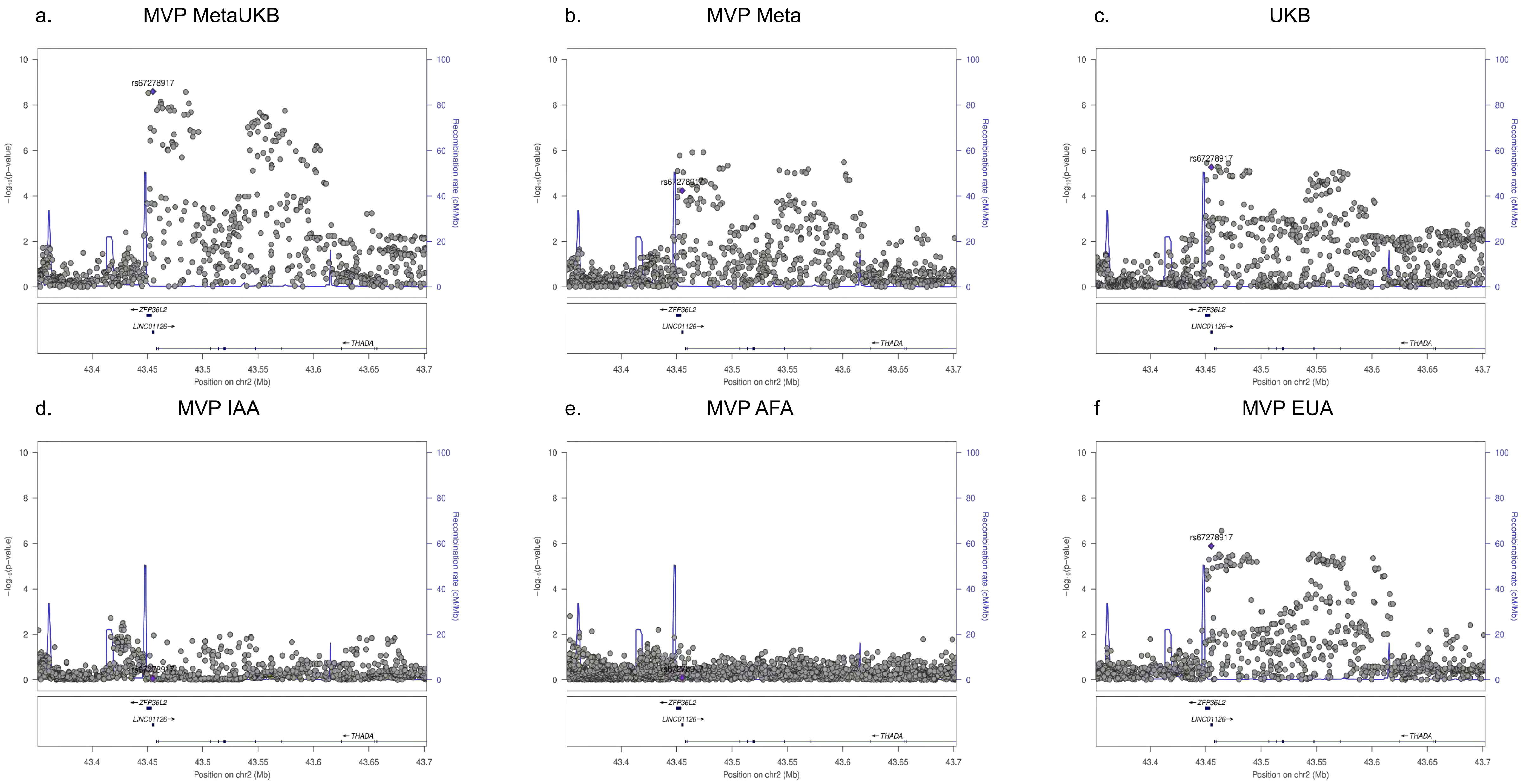

# locus014 | rs2941580

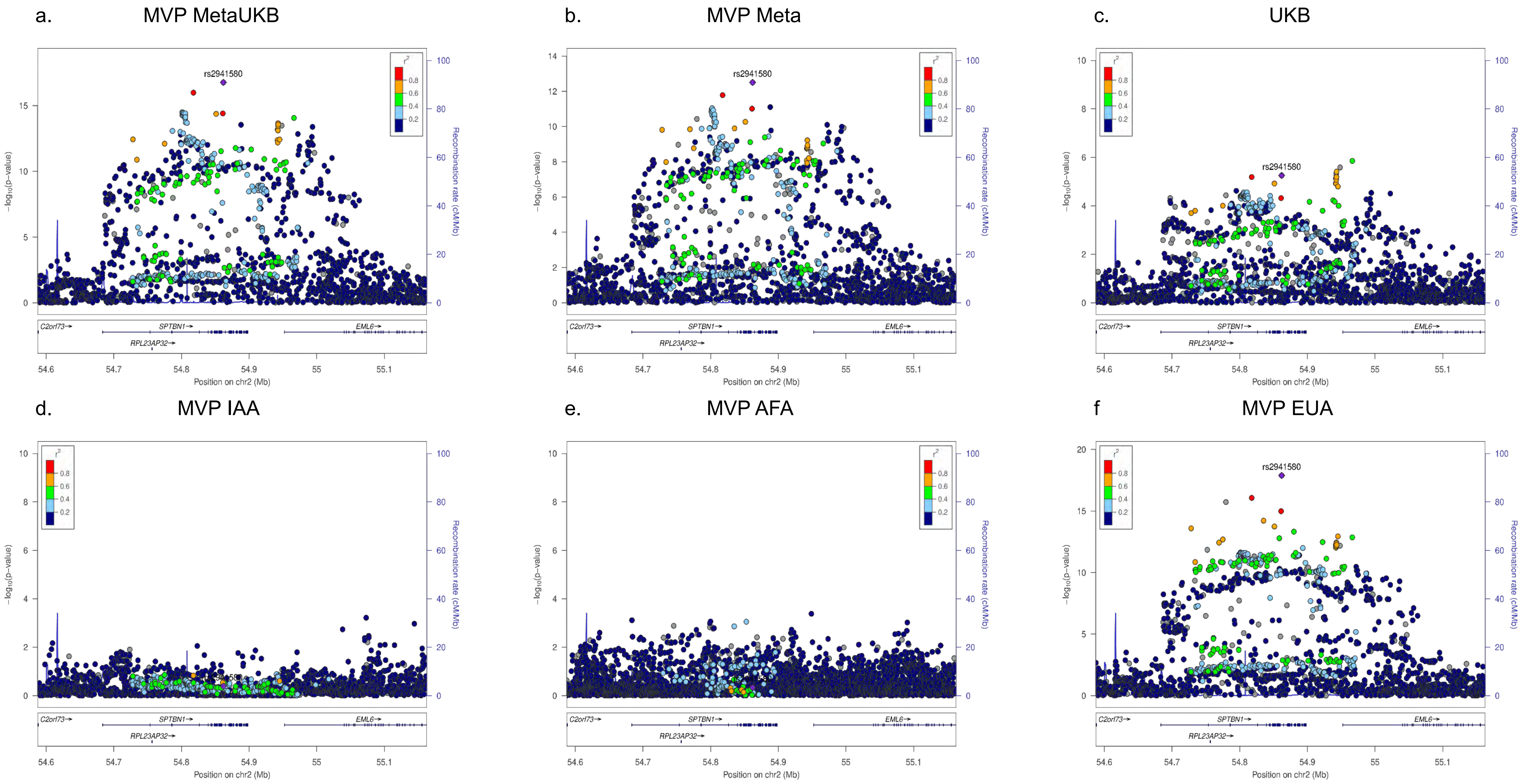

# locus015 | rs2341098

a. MVP MetaUKB

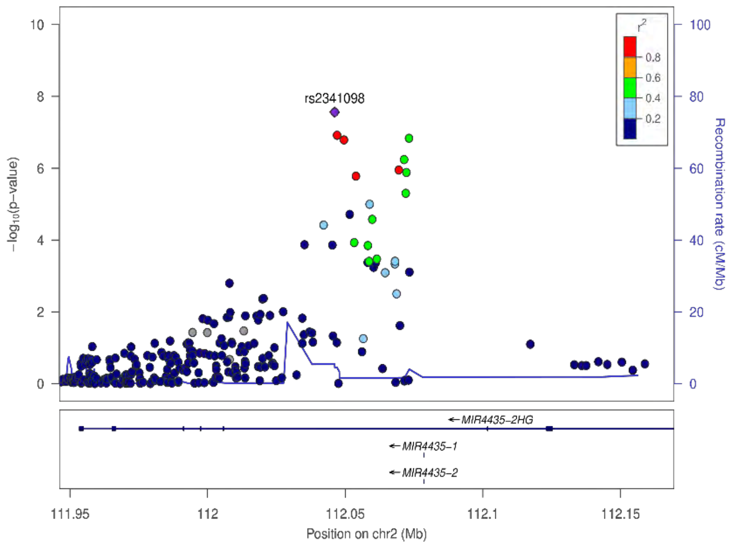


b. MVP Meta

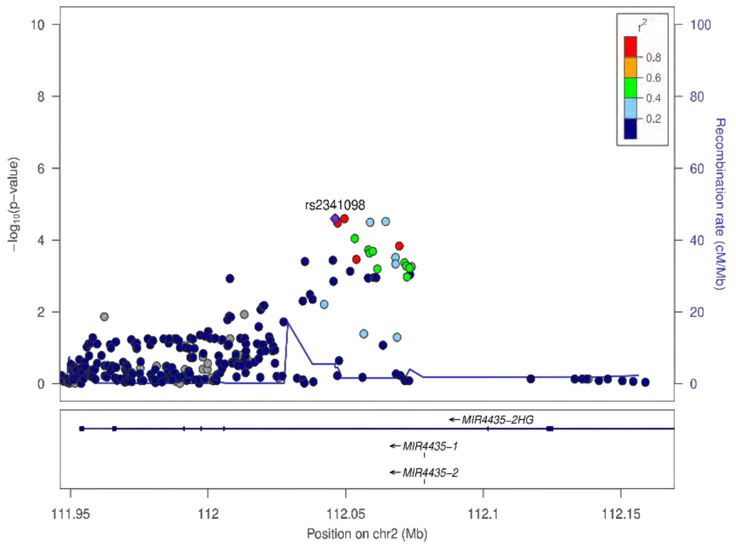


c. UKB

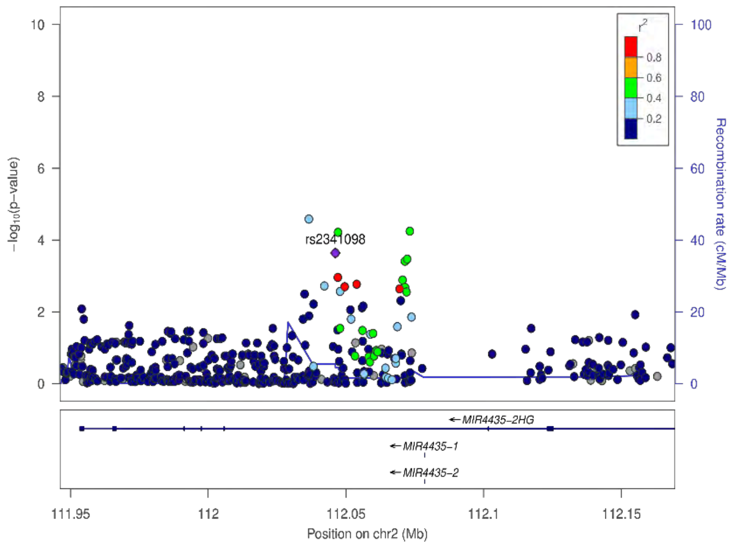


d. MVP IAA

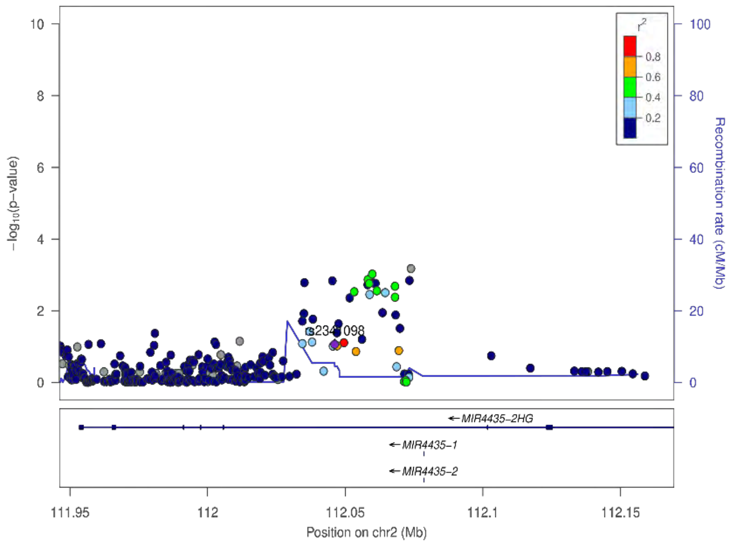


e. MVP AFA

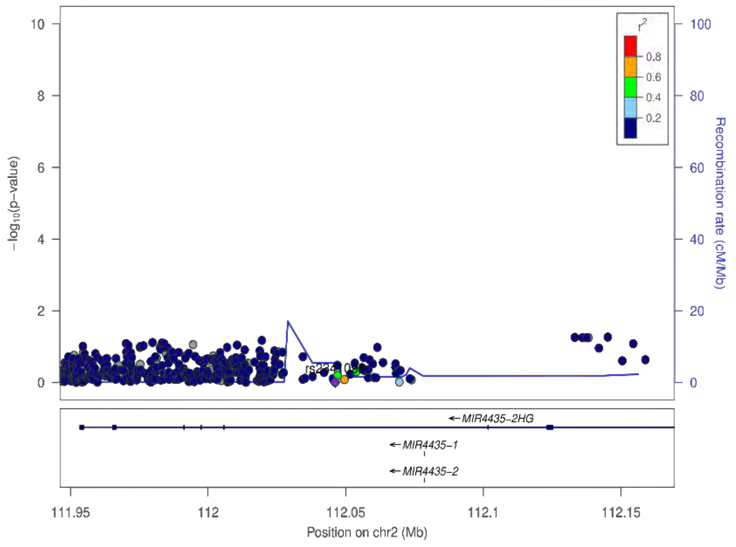


f MVP EUA

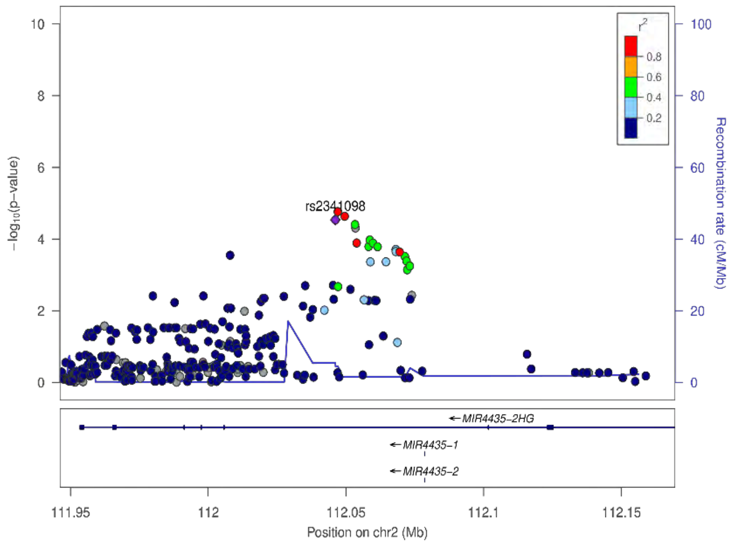

# locus016 | rs2216374

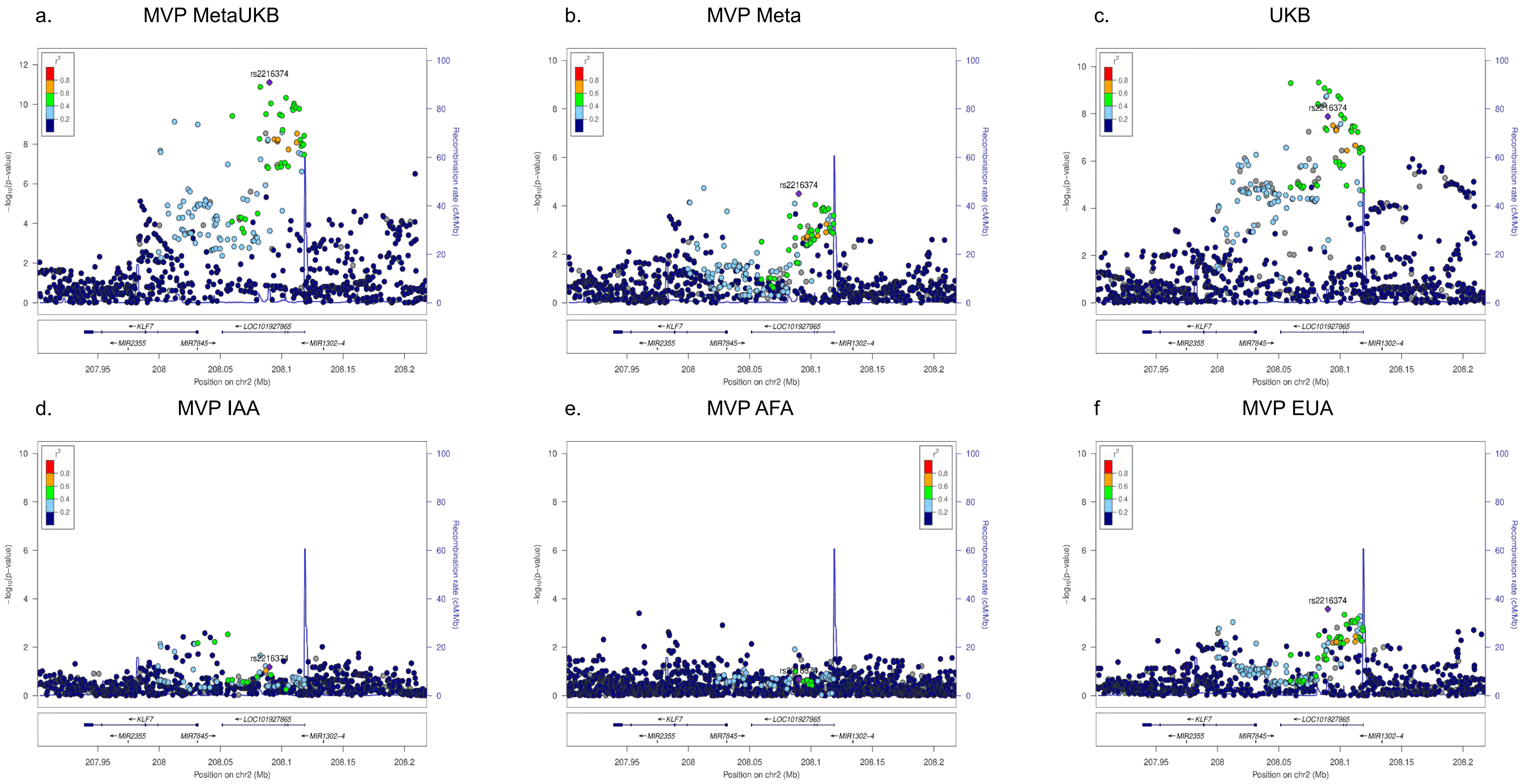

# locus016 | rs62188635

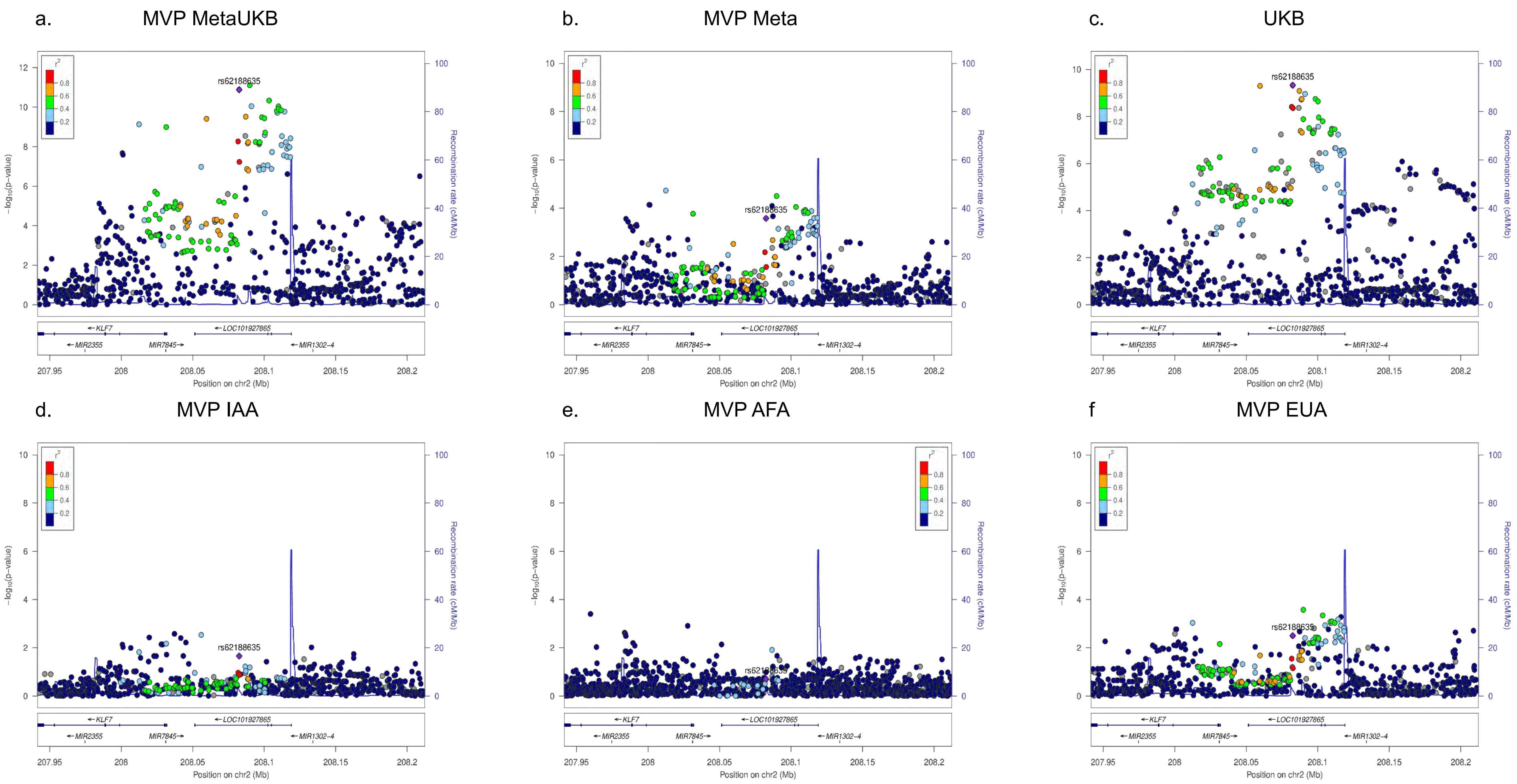

# locus017 | rs511273

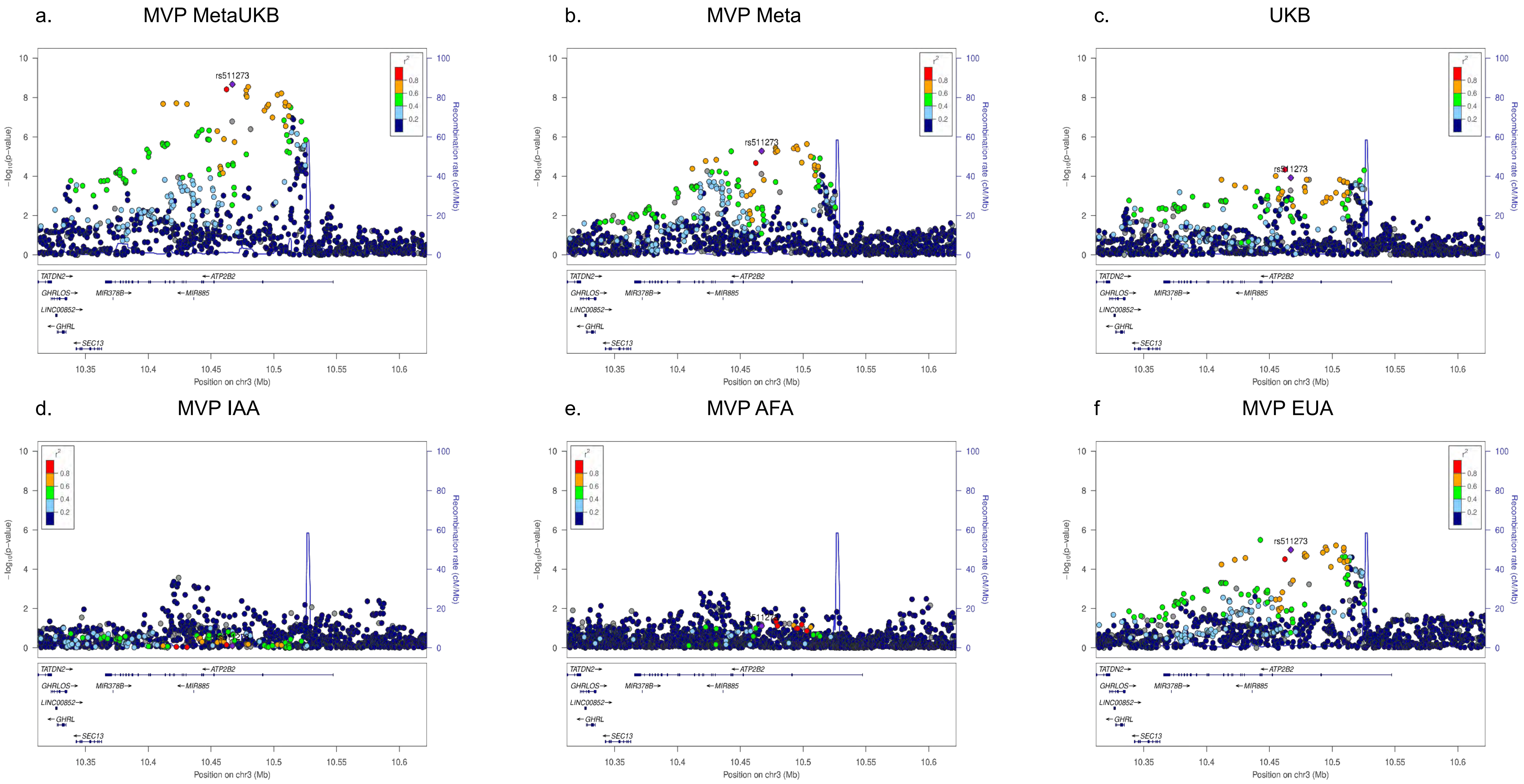

# locus018 | rs877306

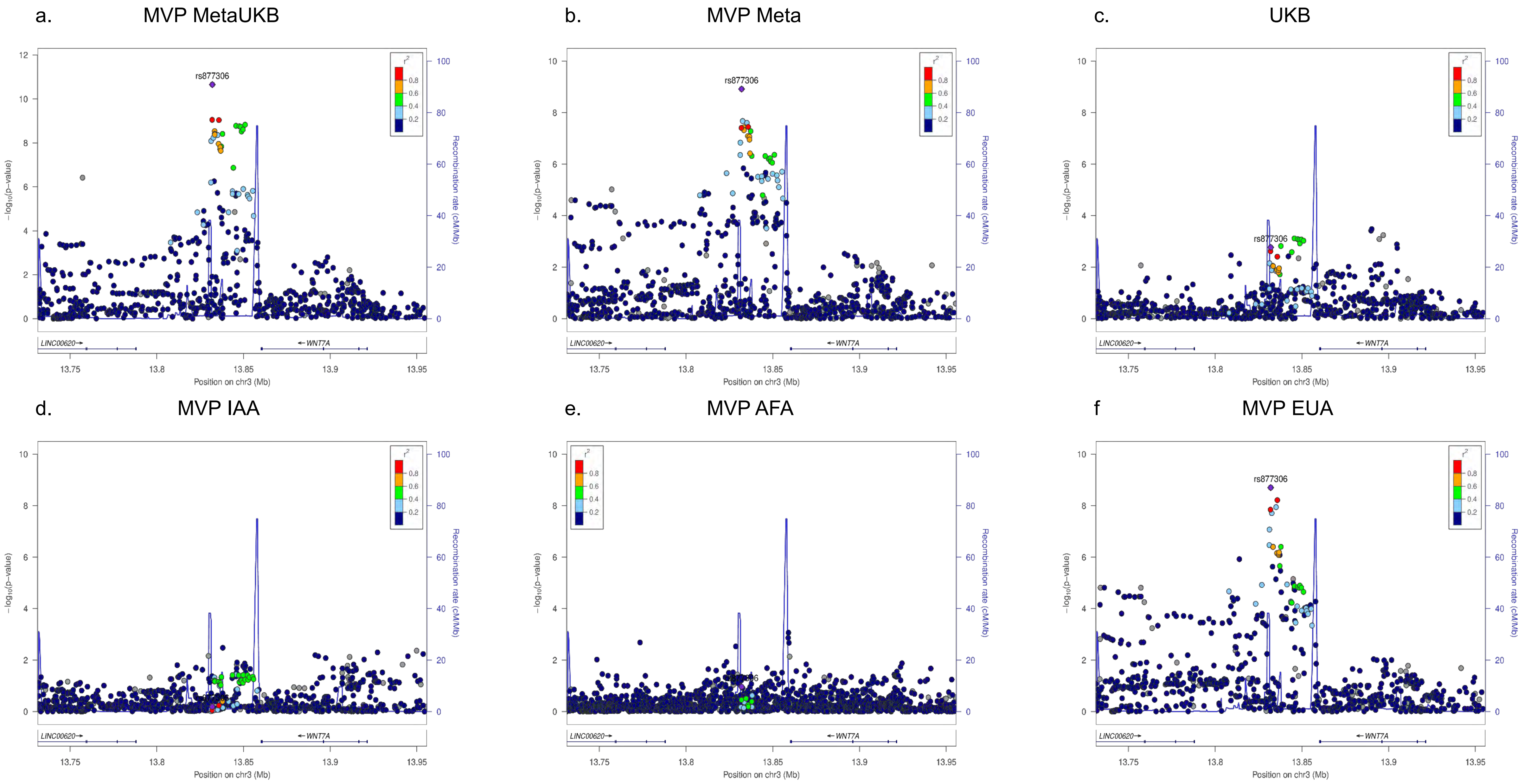

# locus019 | rs6779690

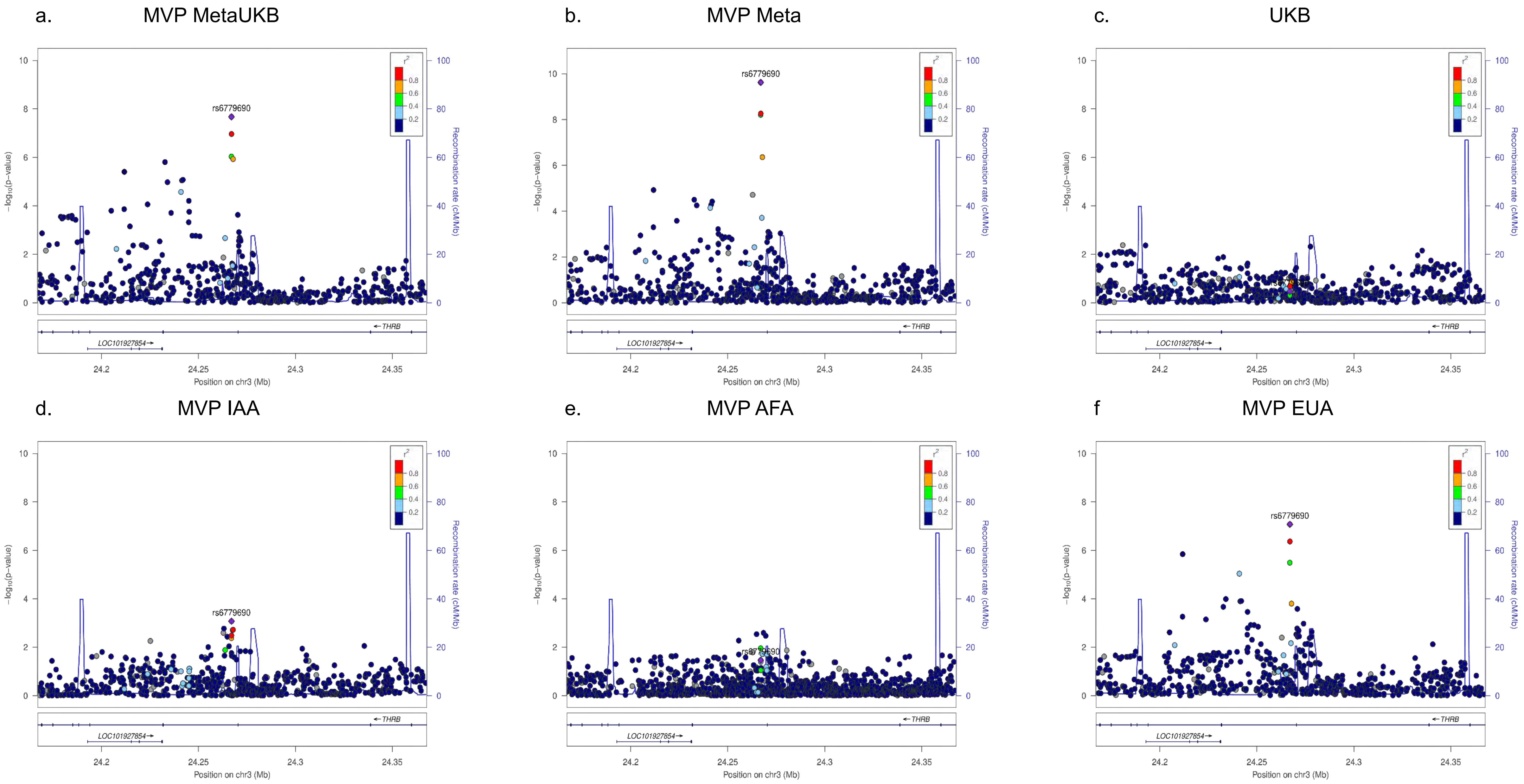

# locus020 | rs75175086

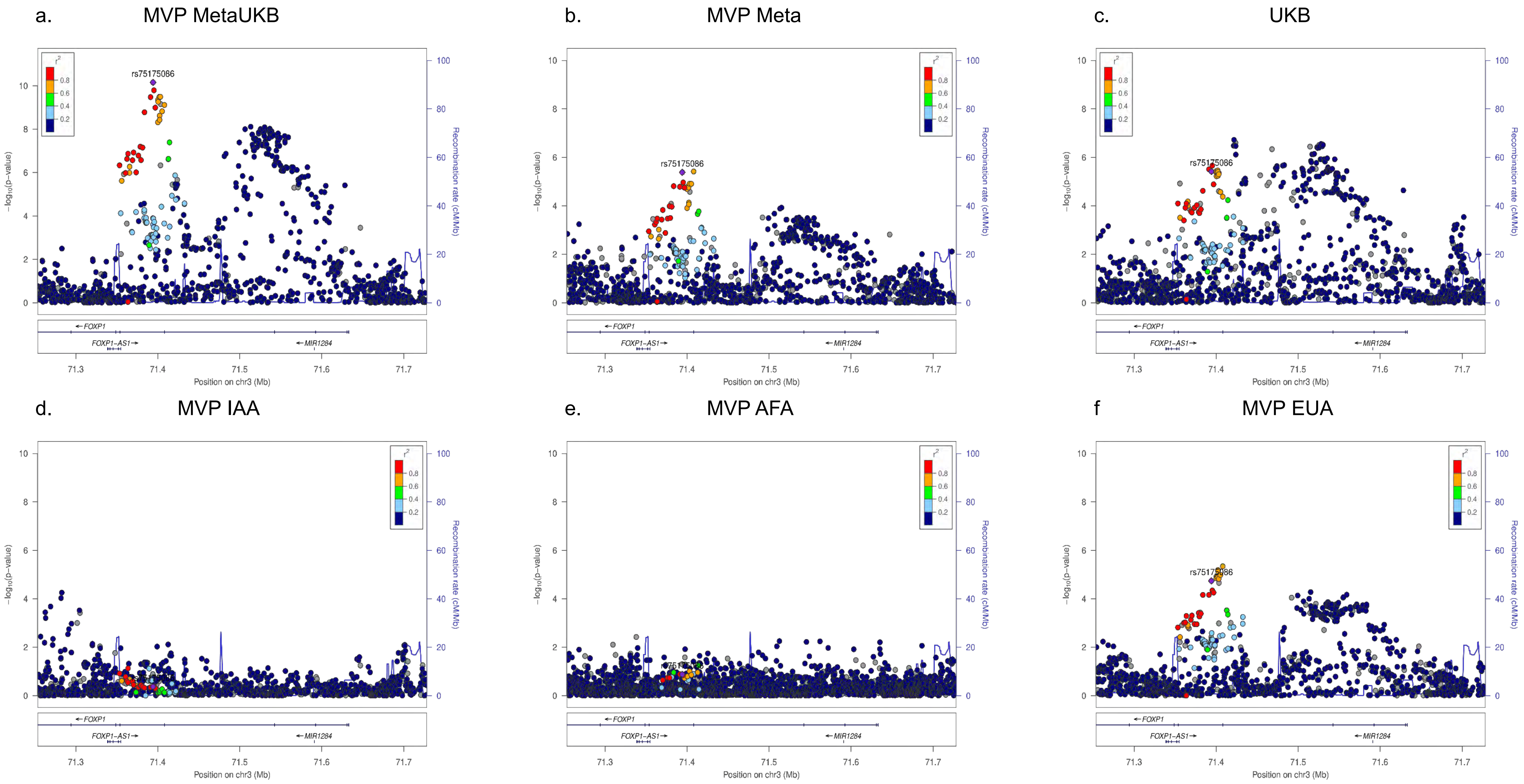

# locus021 | rs9853536

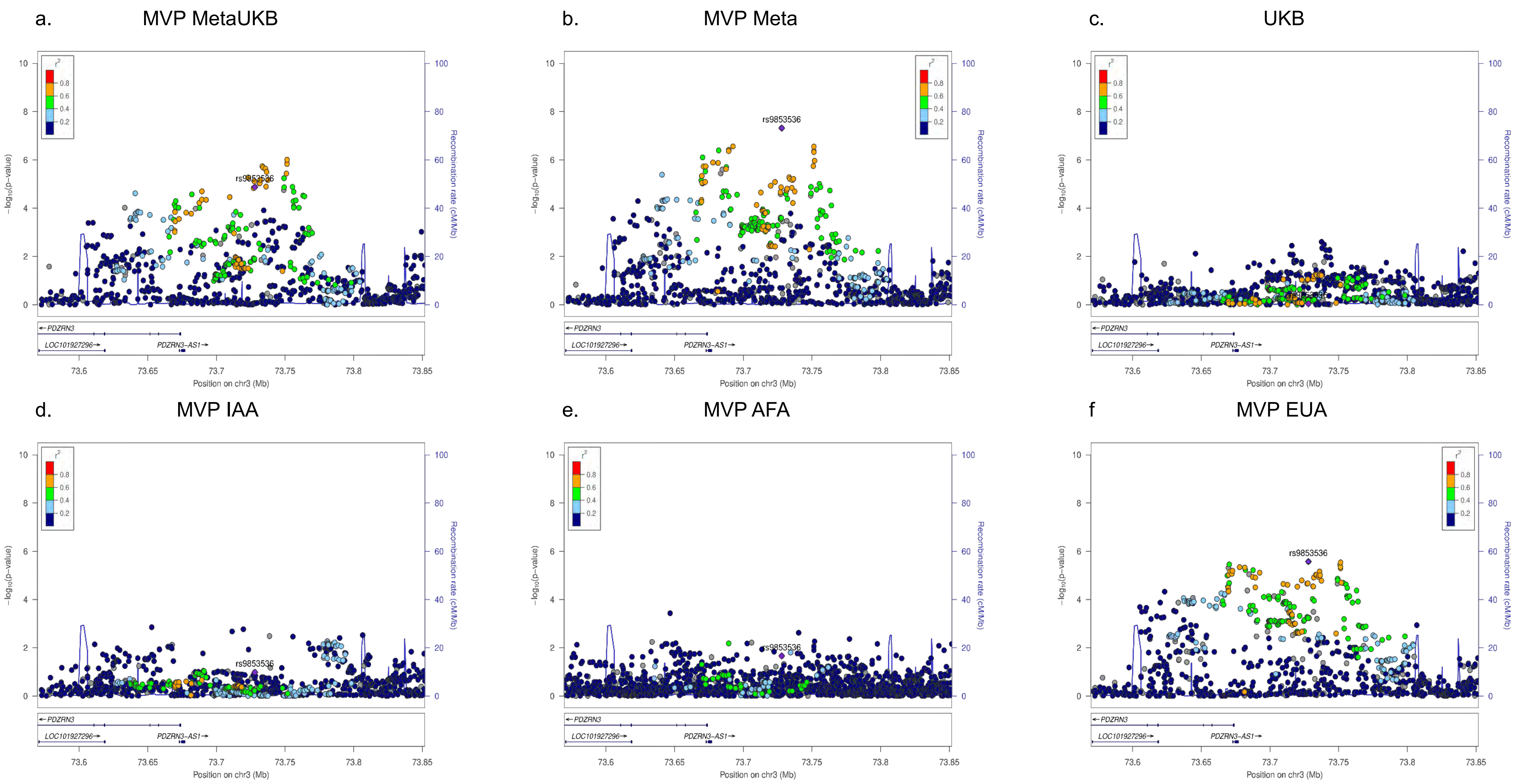

locus022 | rs13093972

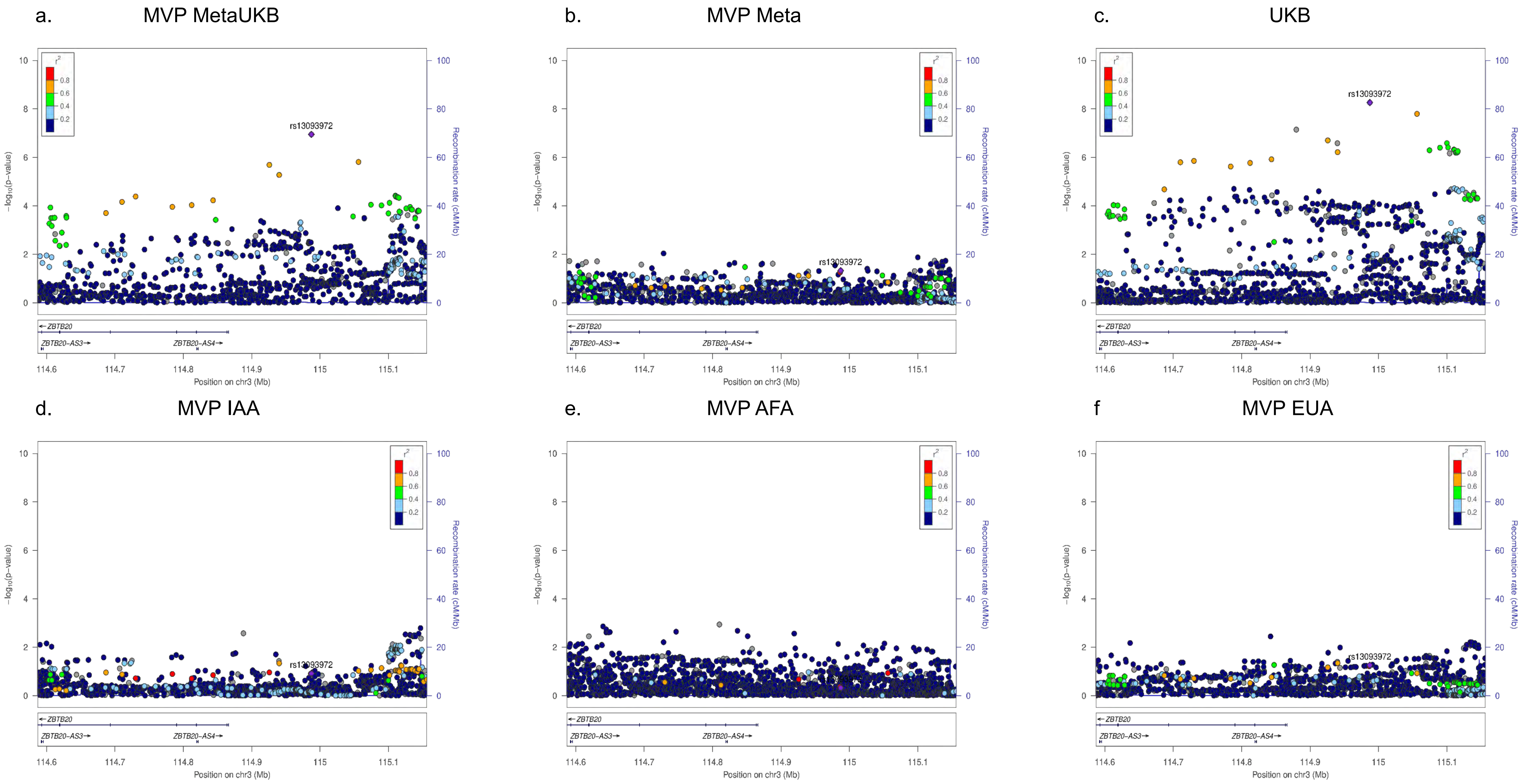

# locus023 | rs3915060

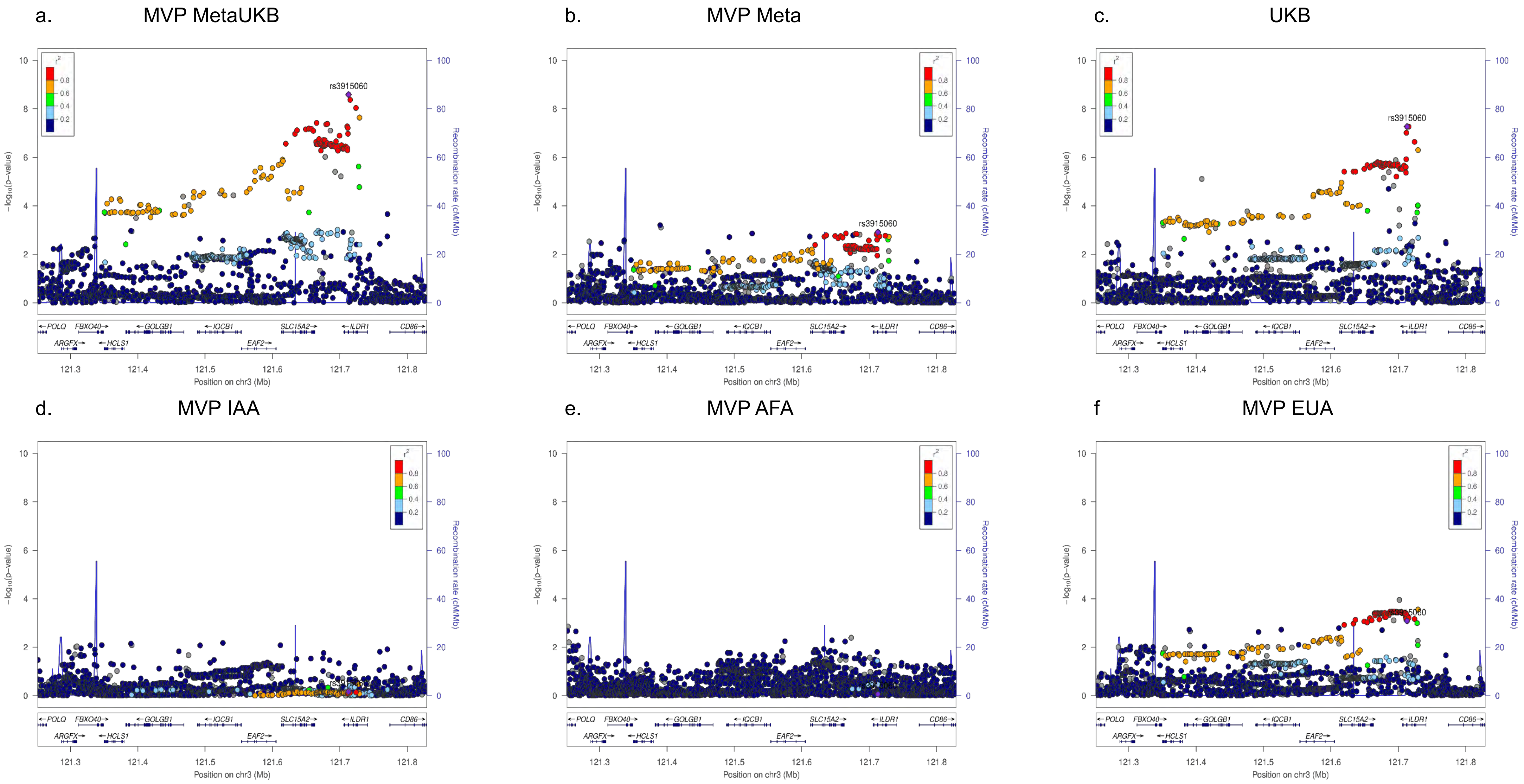

# locus024 | rs200745338

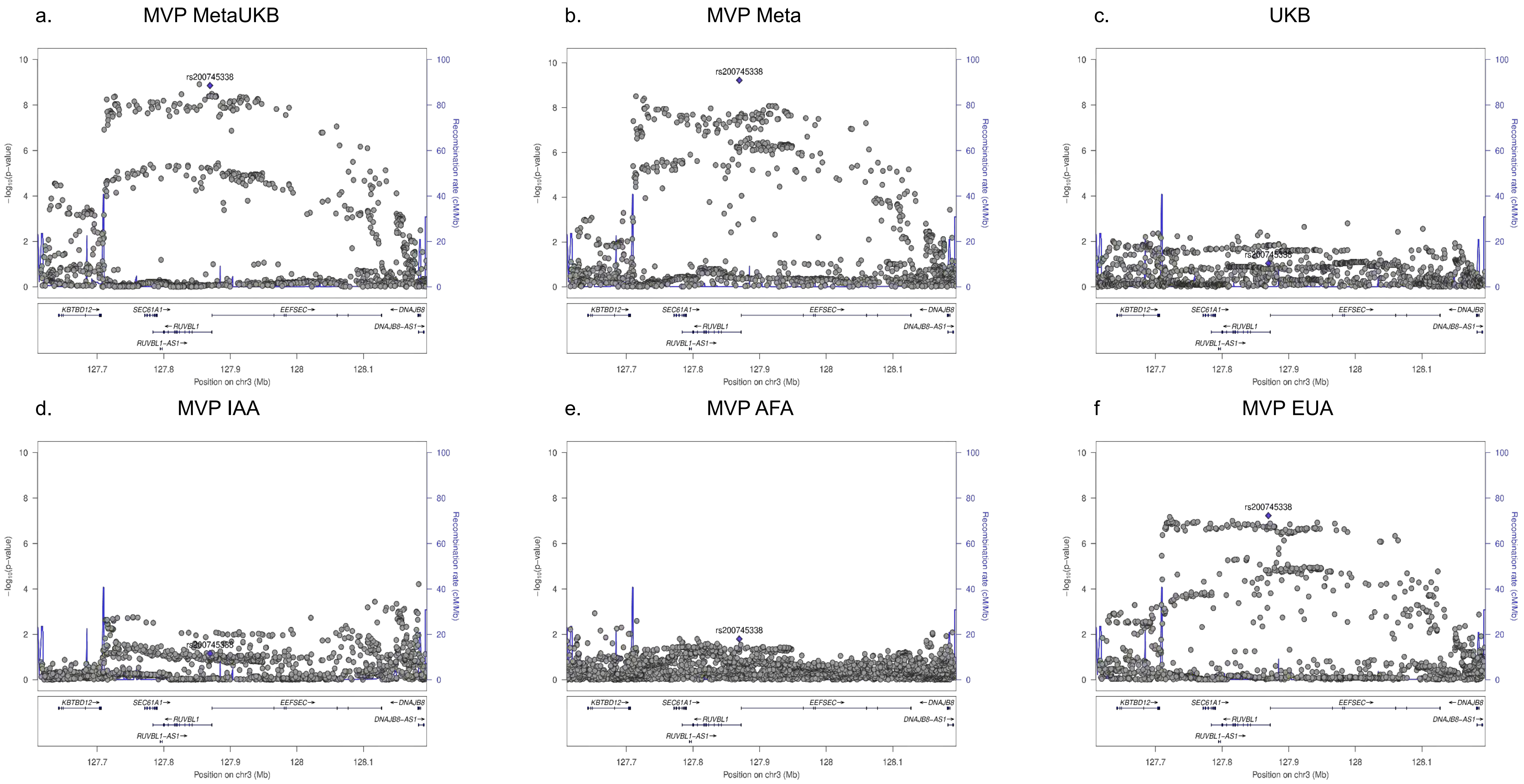

# locus024 | rs4857868

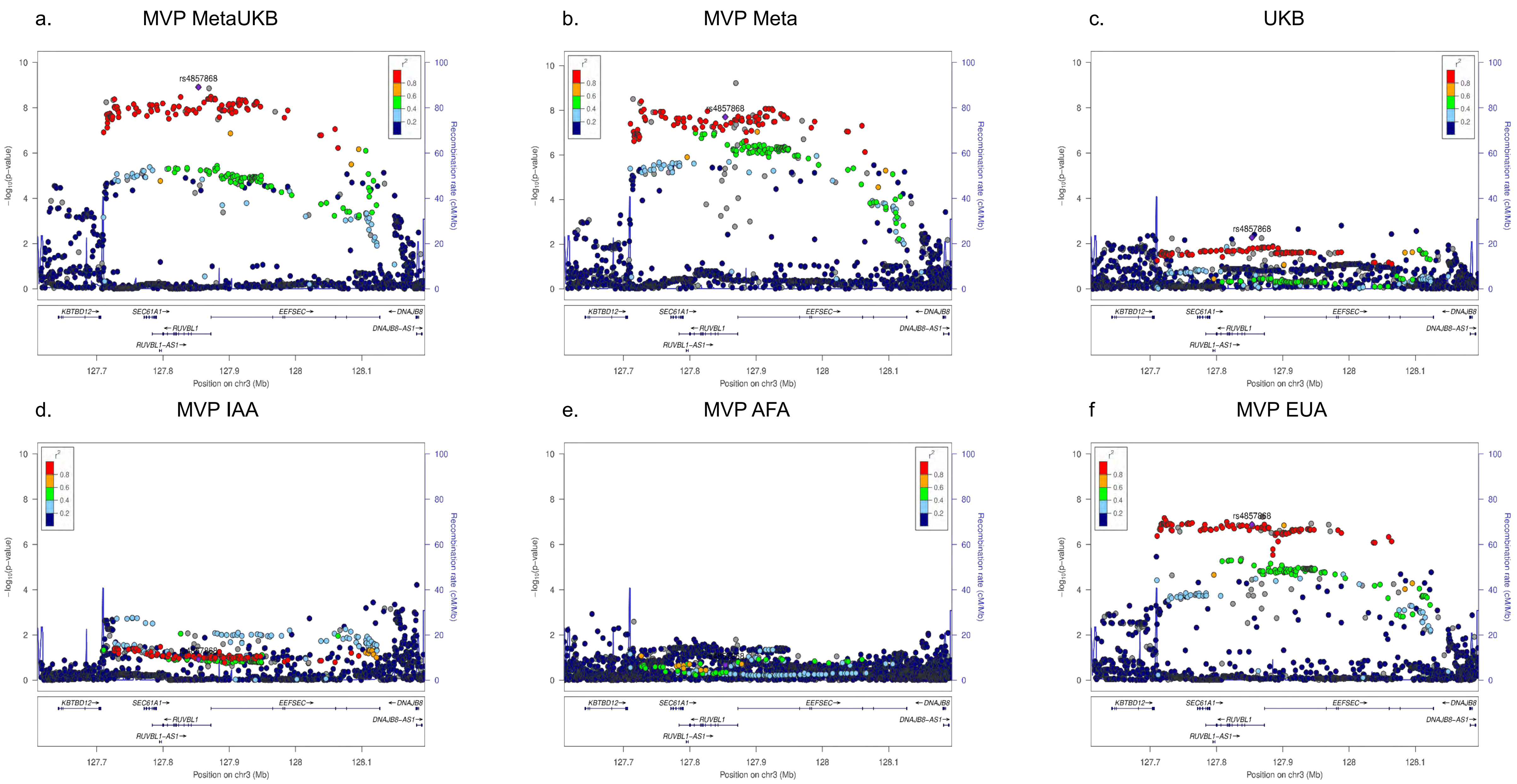

# locus025 | rs9856073

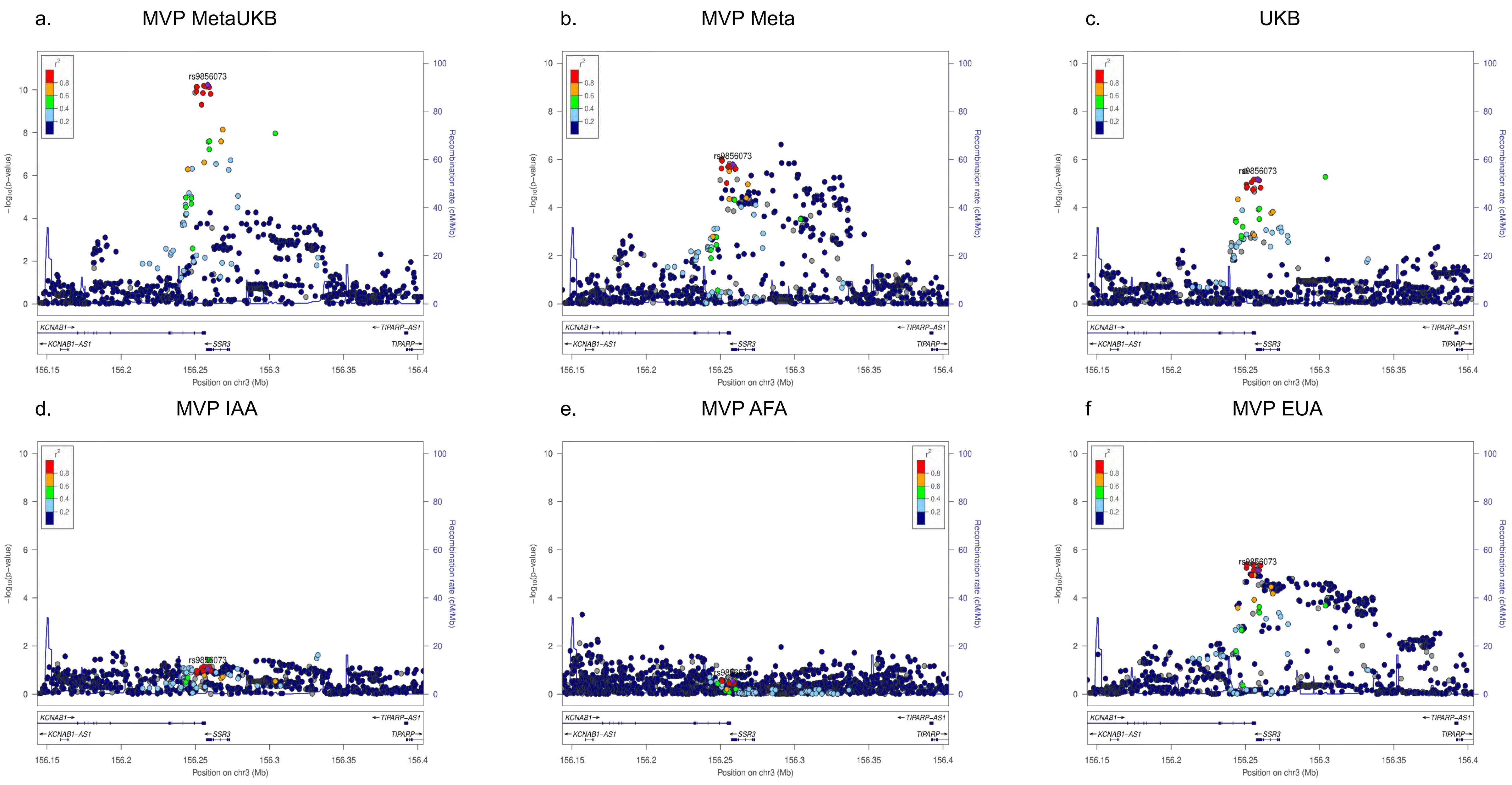

# locus026 | rs4859116

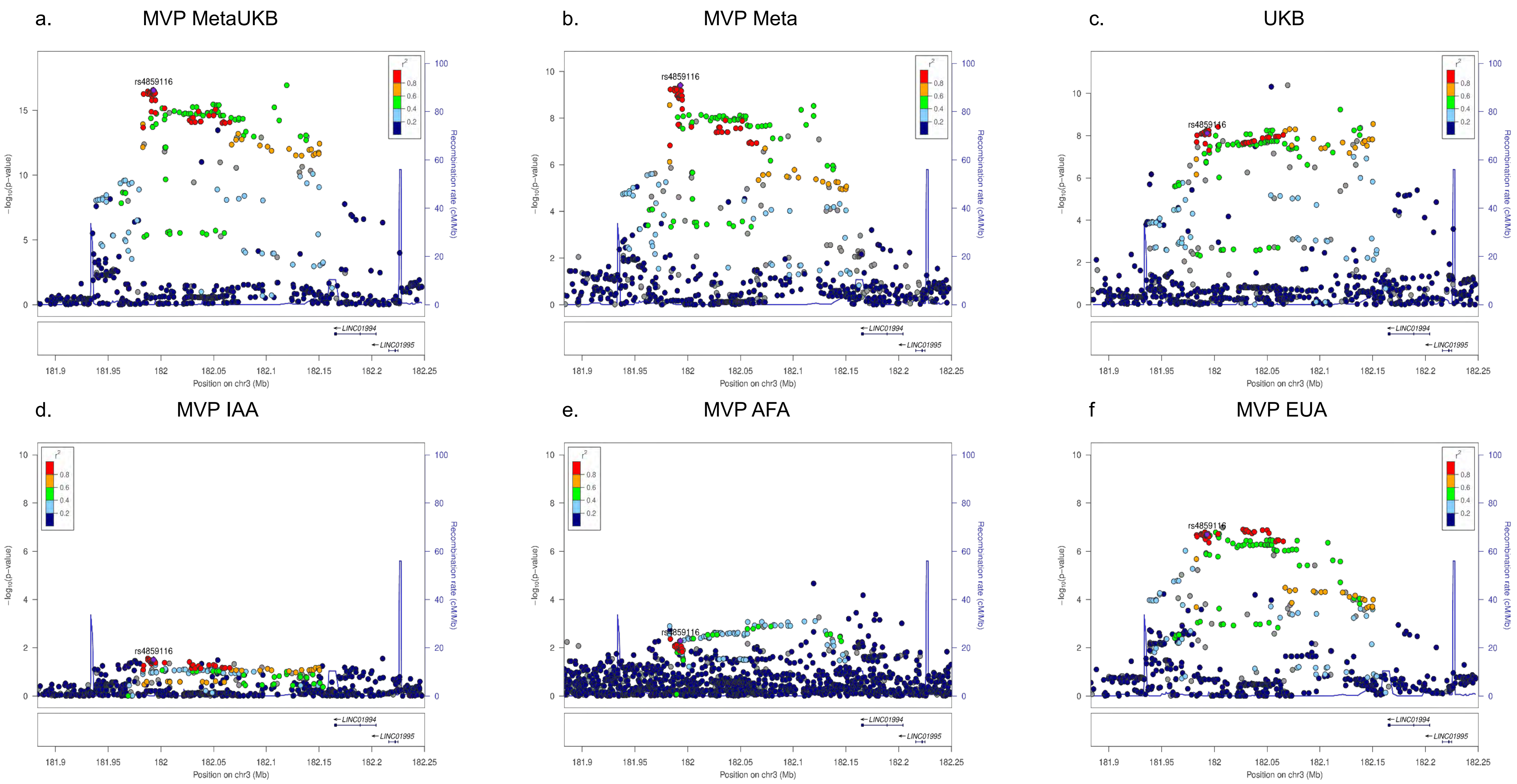

# locus026 | rs7649191

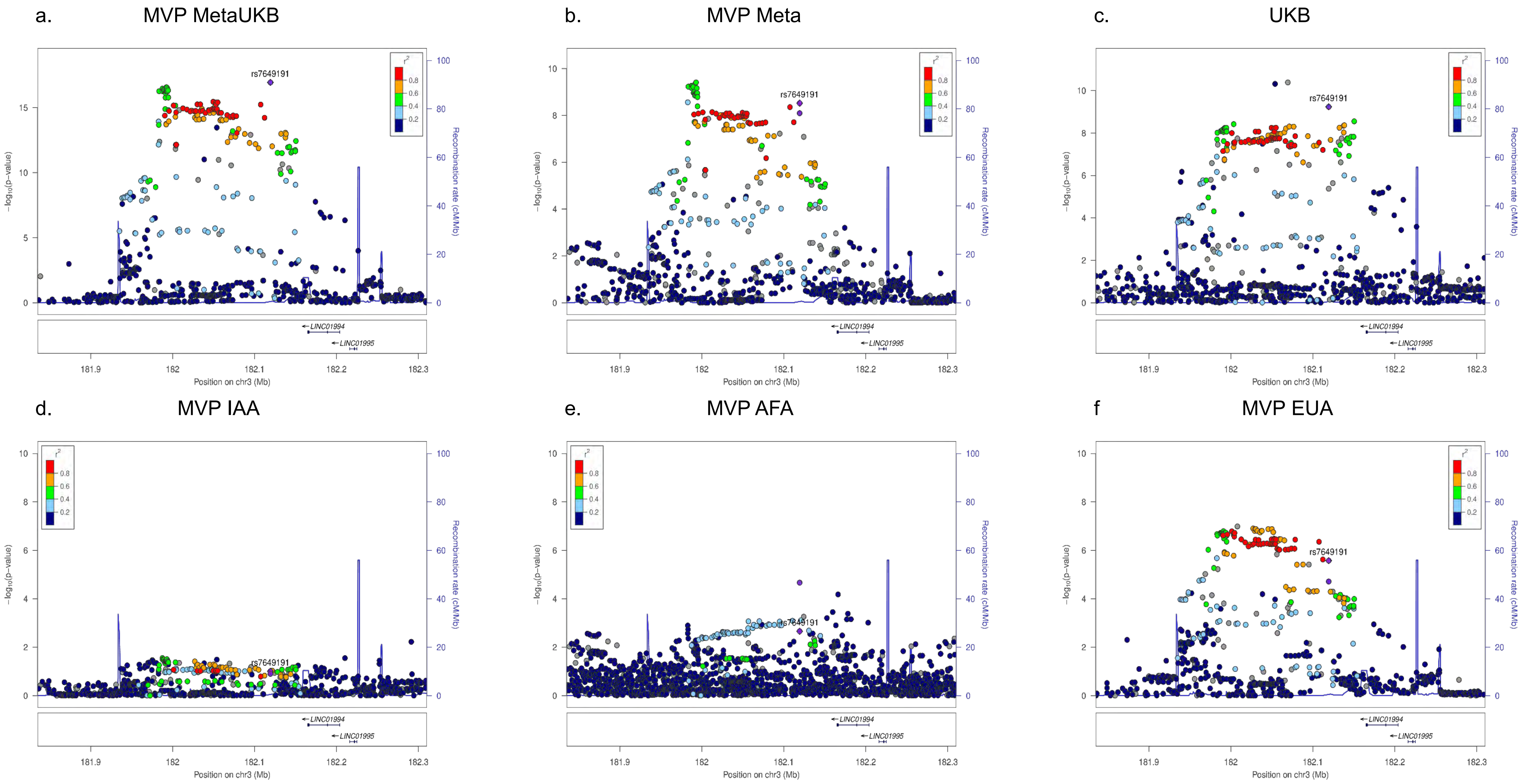

locus027 | rs569238340

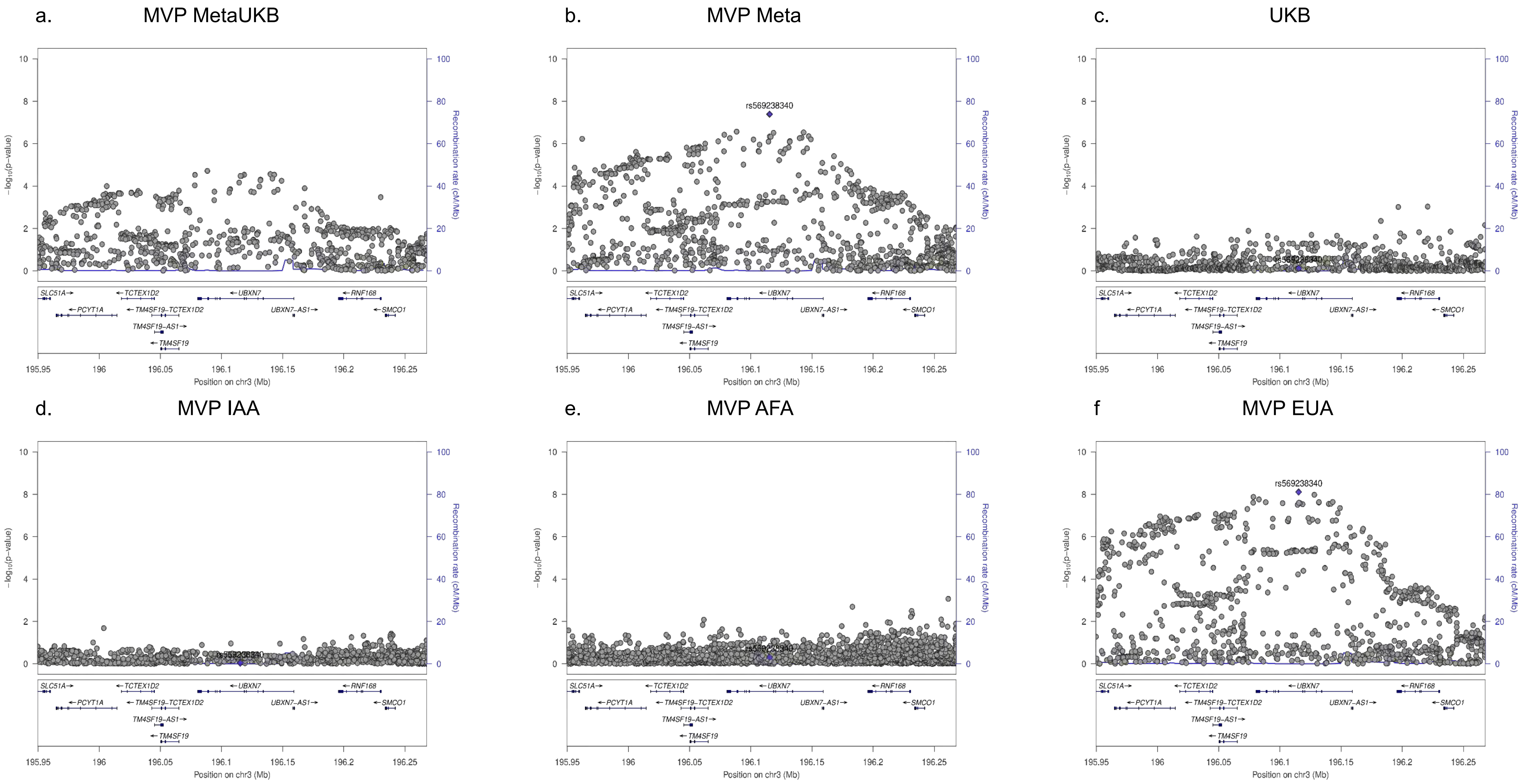

# locus028 | rs3129327

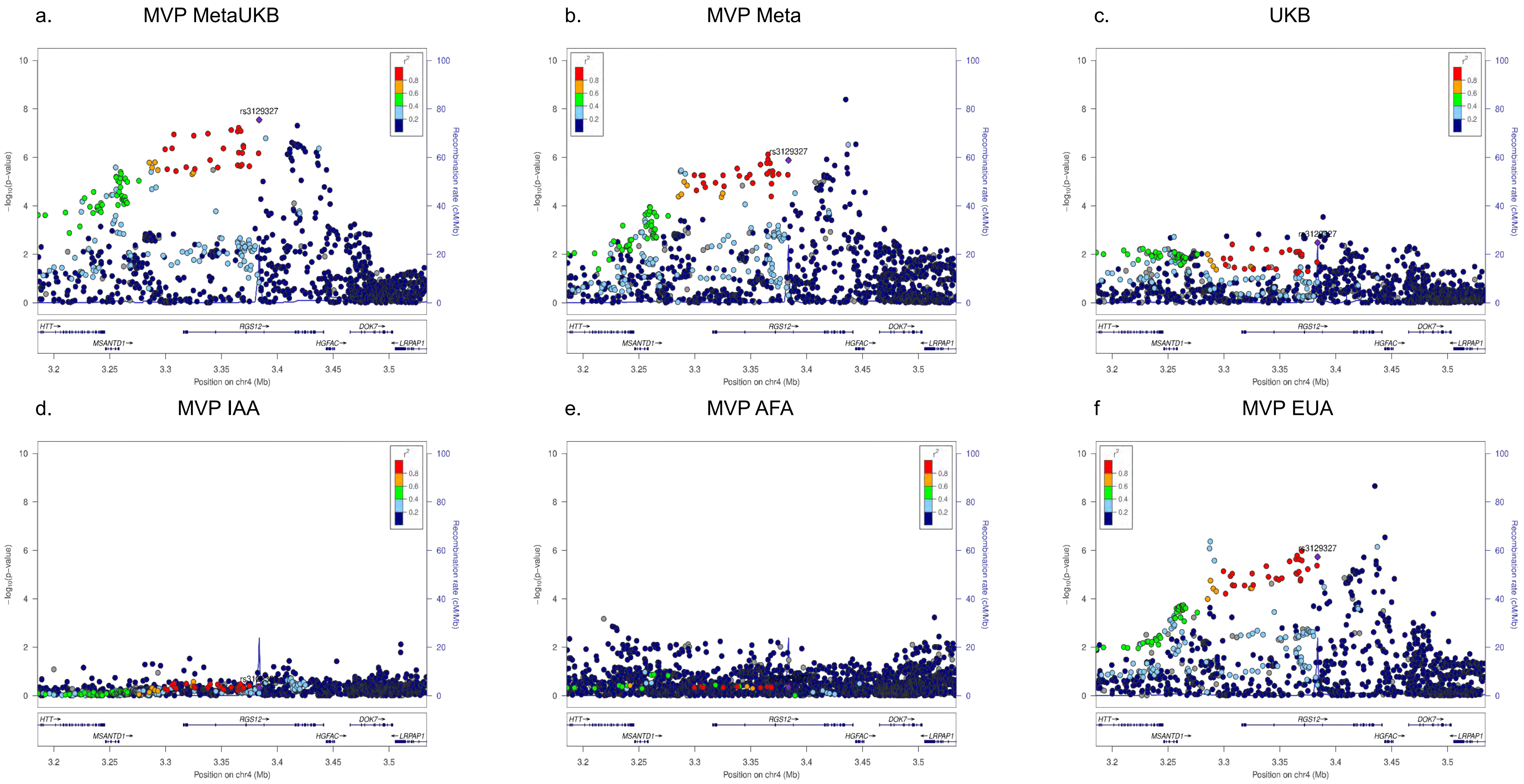

locus029 | rs6818397

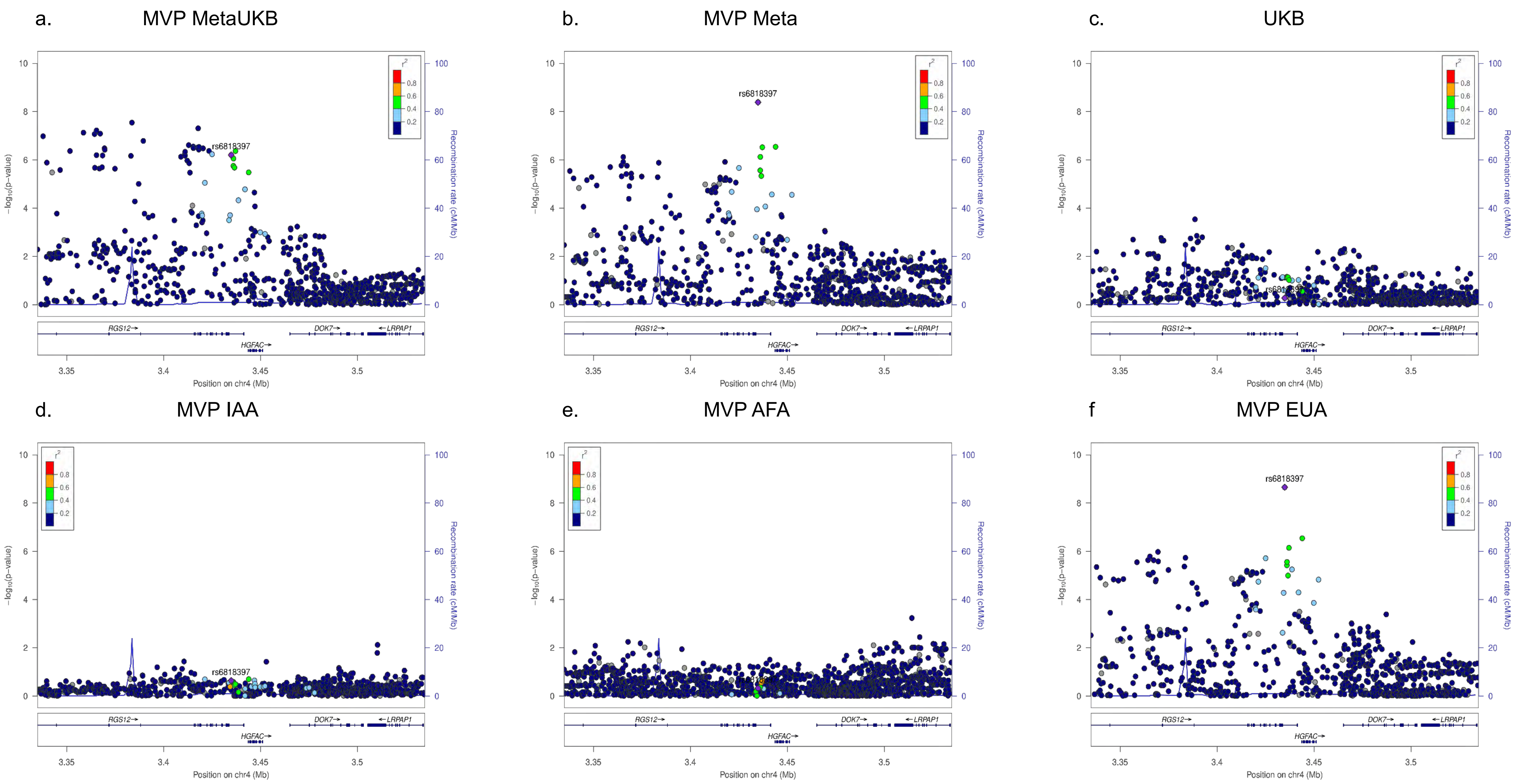

# locus030 | rs28369282

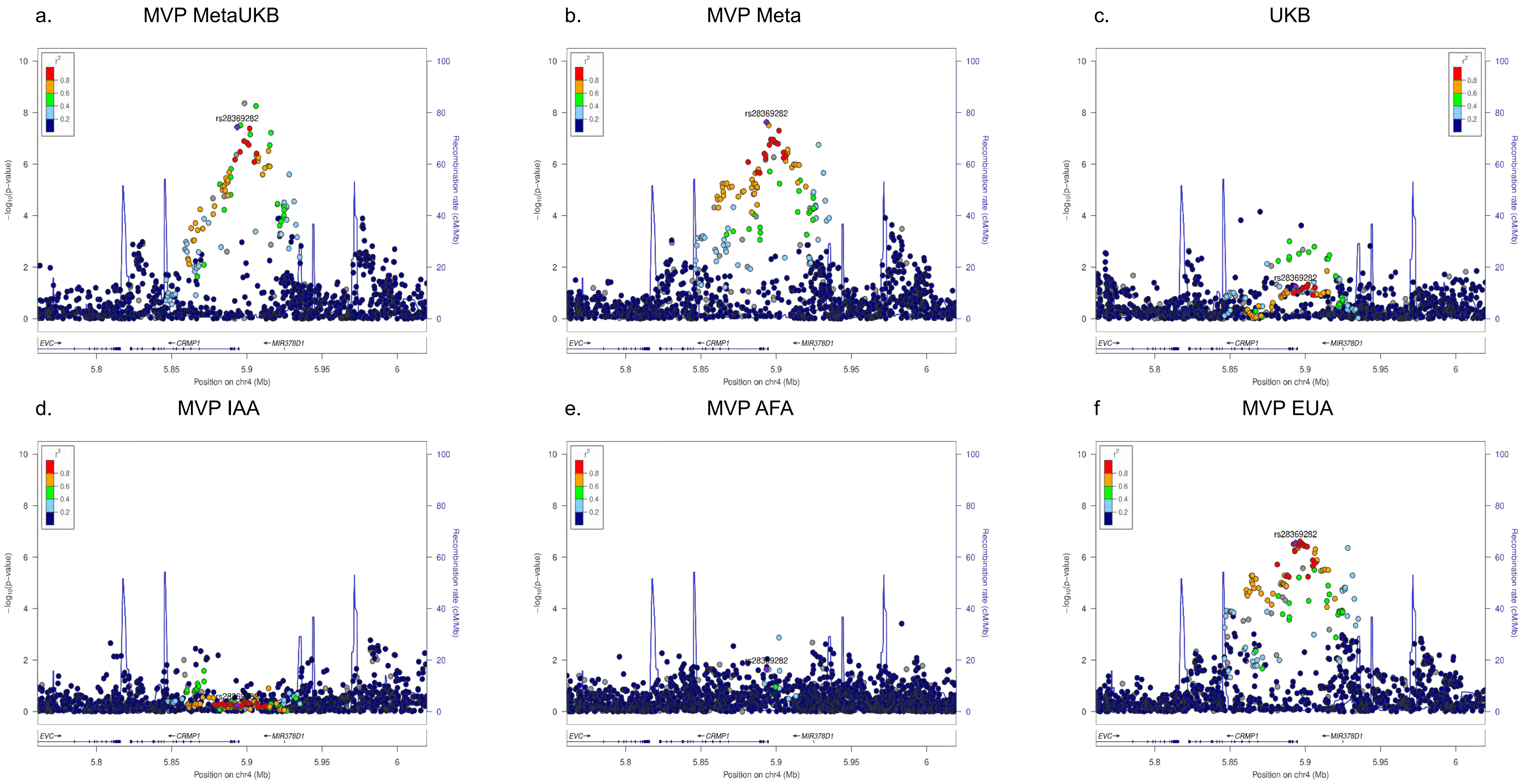

# locus030 | rs6446406

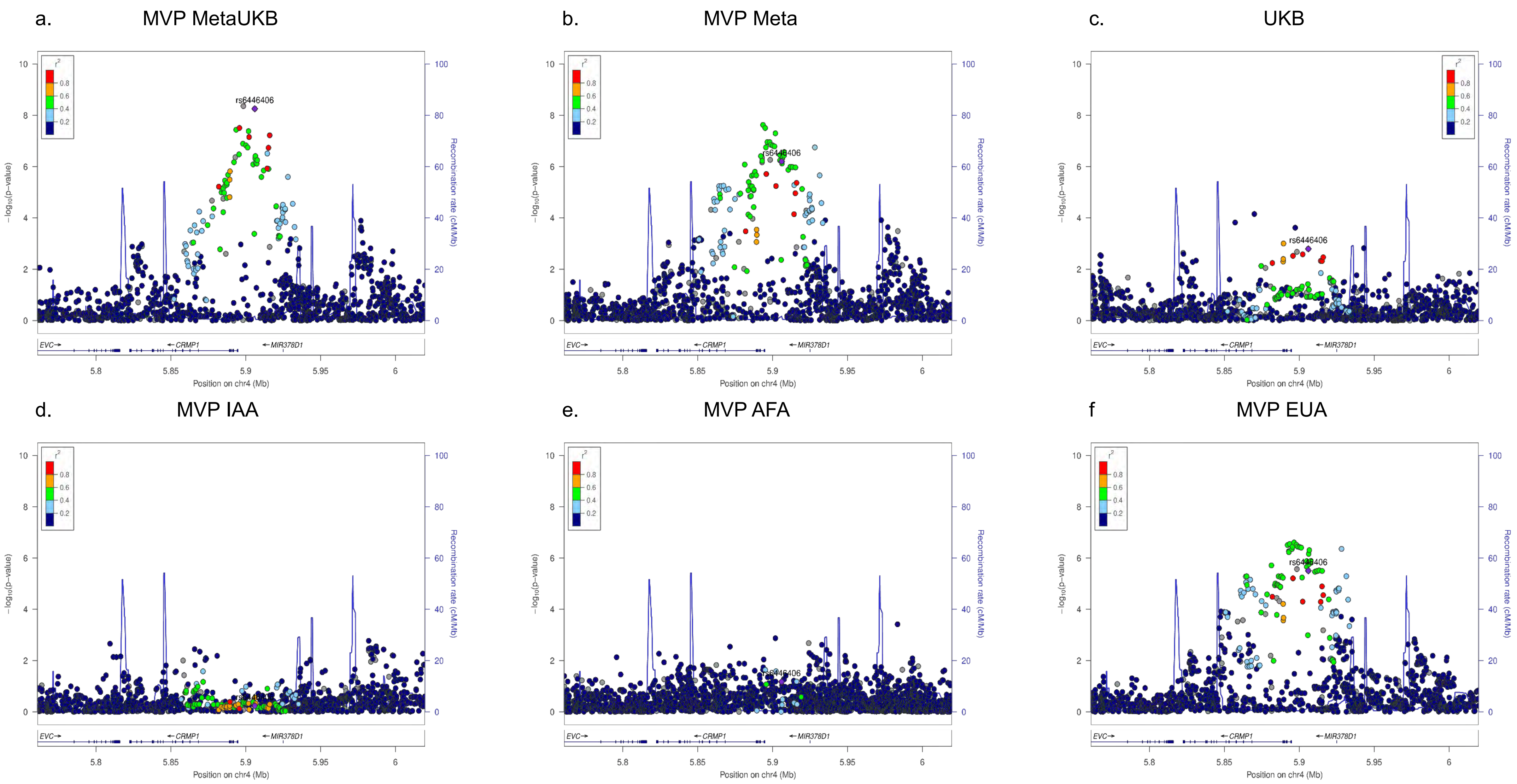

# locus031 | rs10489074

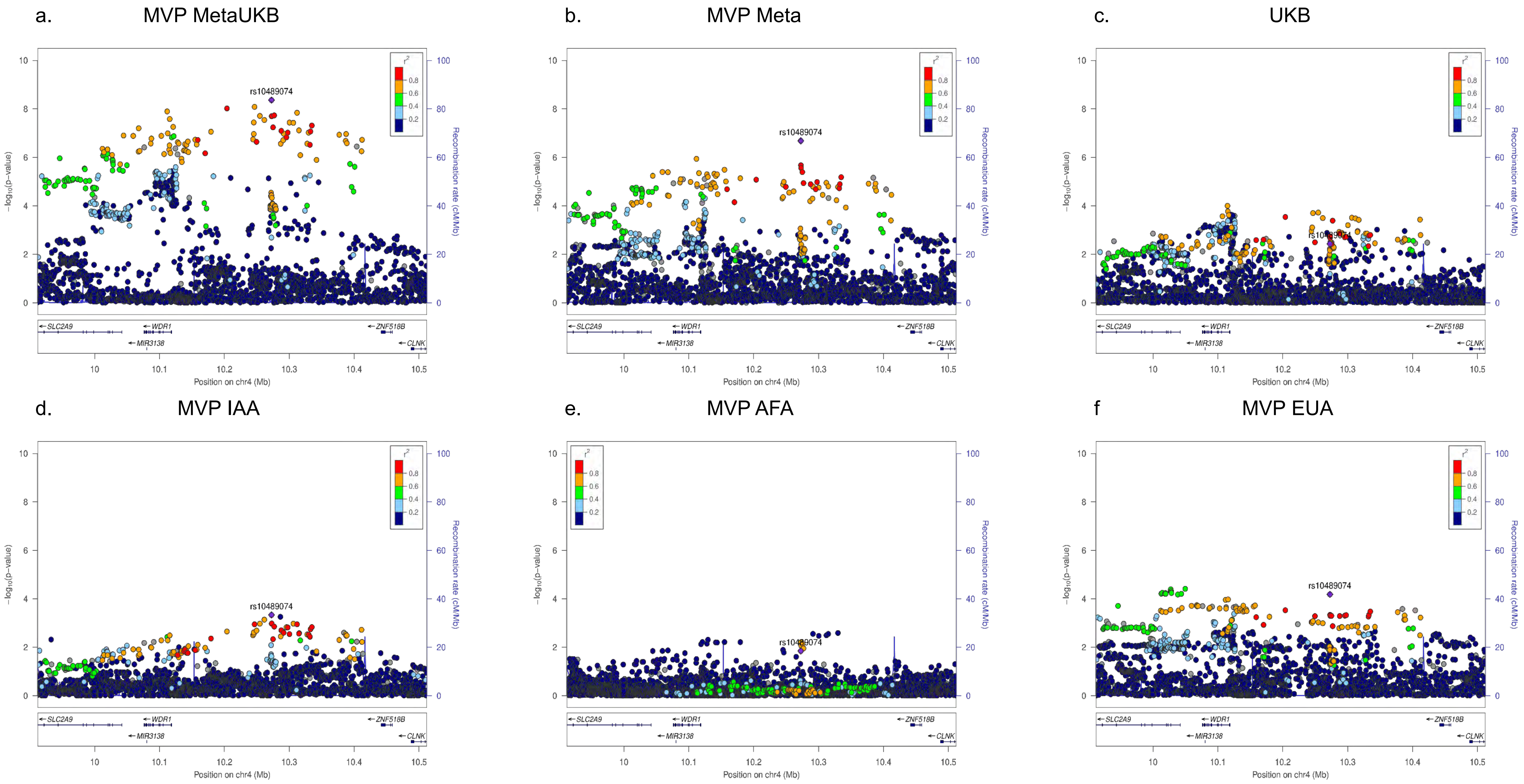

# locus032 | rs13147559

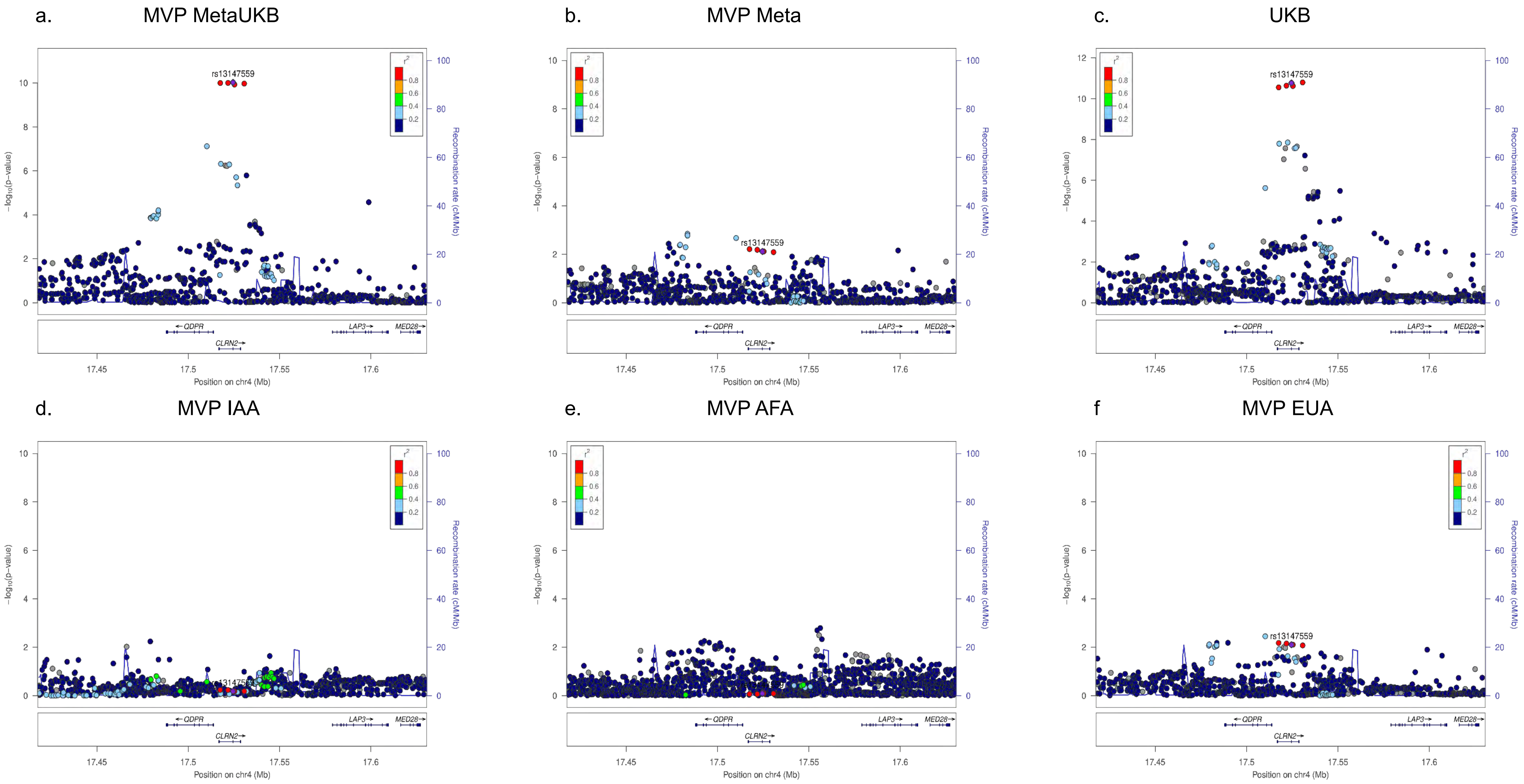

# locus032 | rs35414371

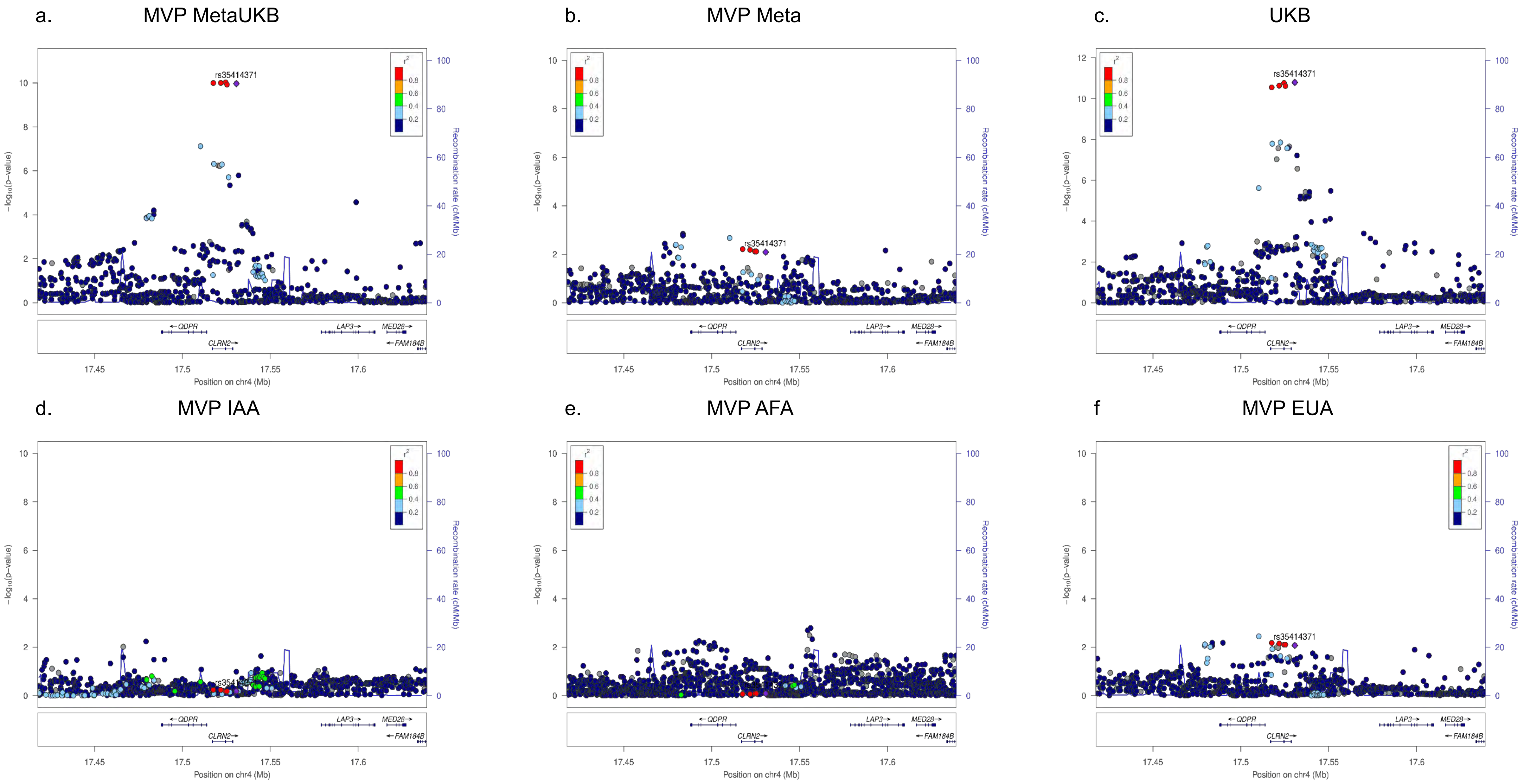

# locus033 | rs13152711

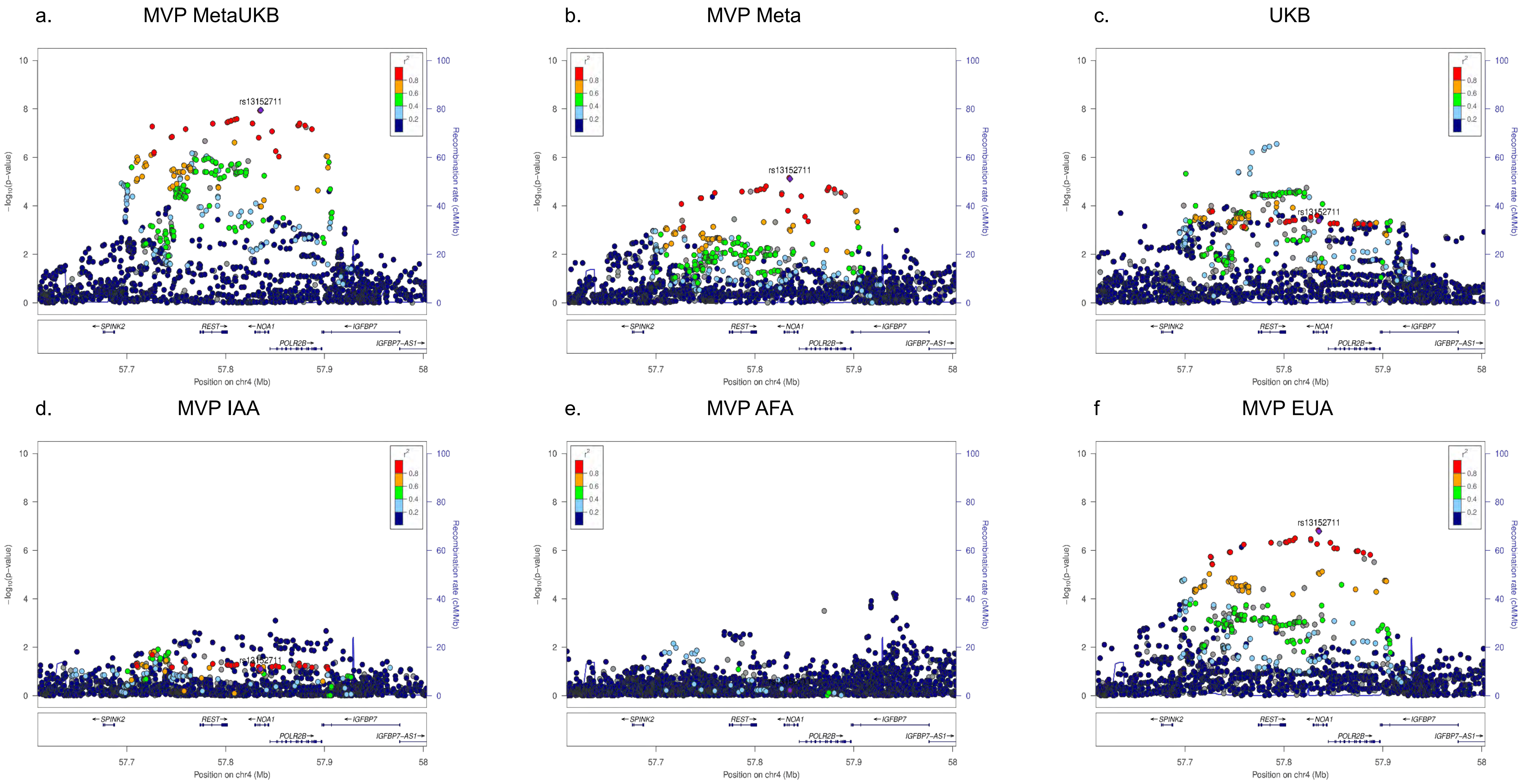

# locus034 | rs10475169

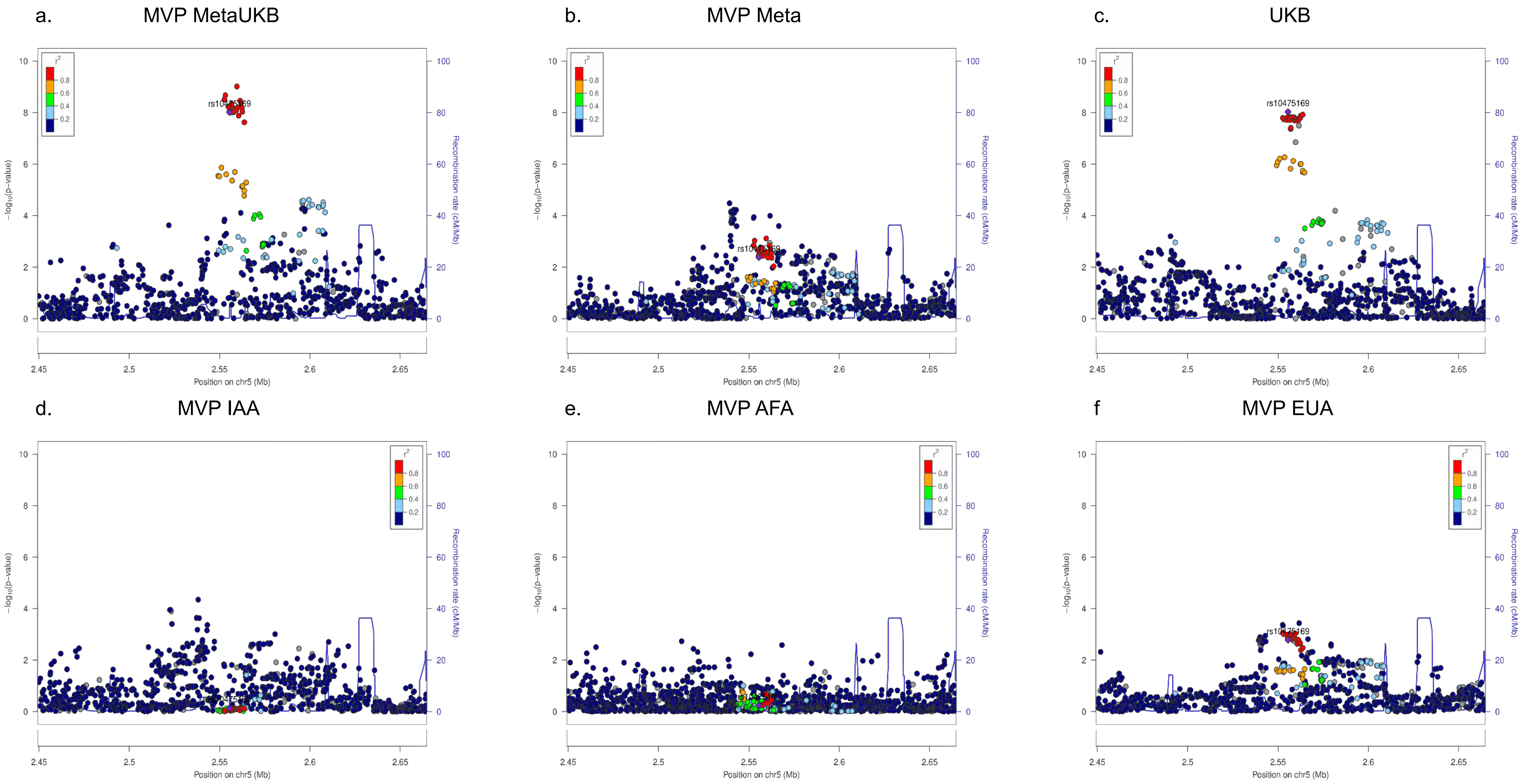

# locus034 | rs7712395

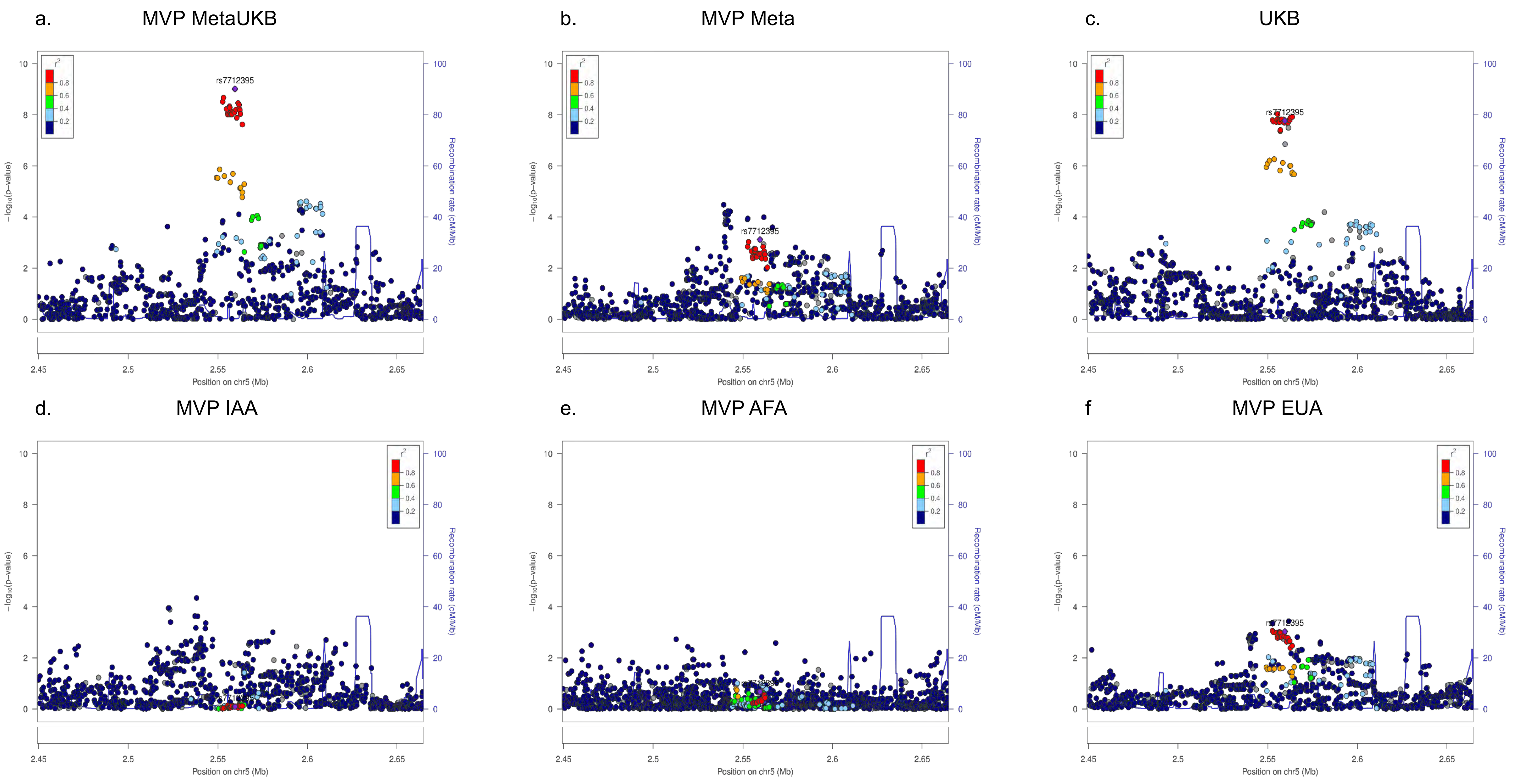

# locus035 | rs10941003

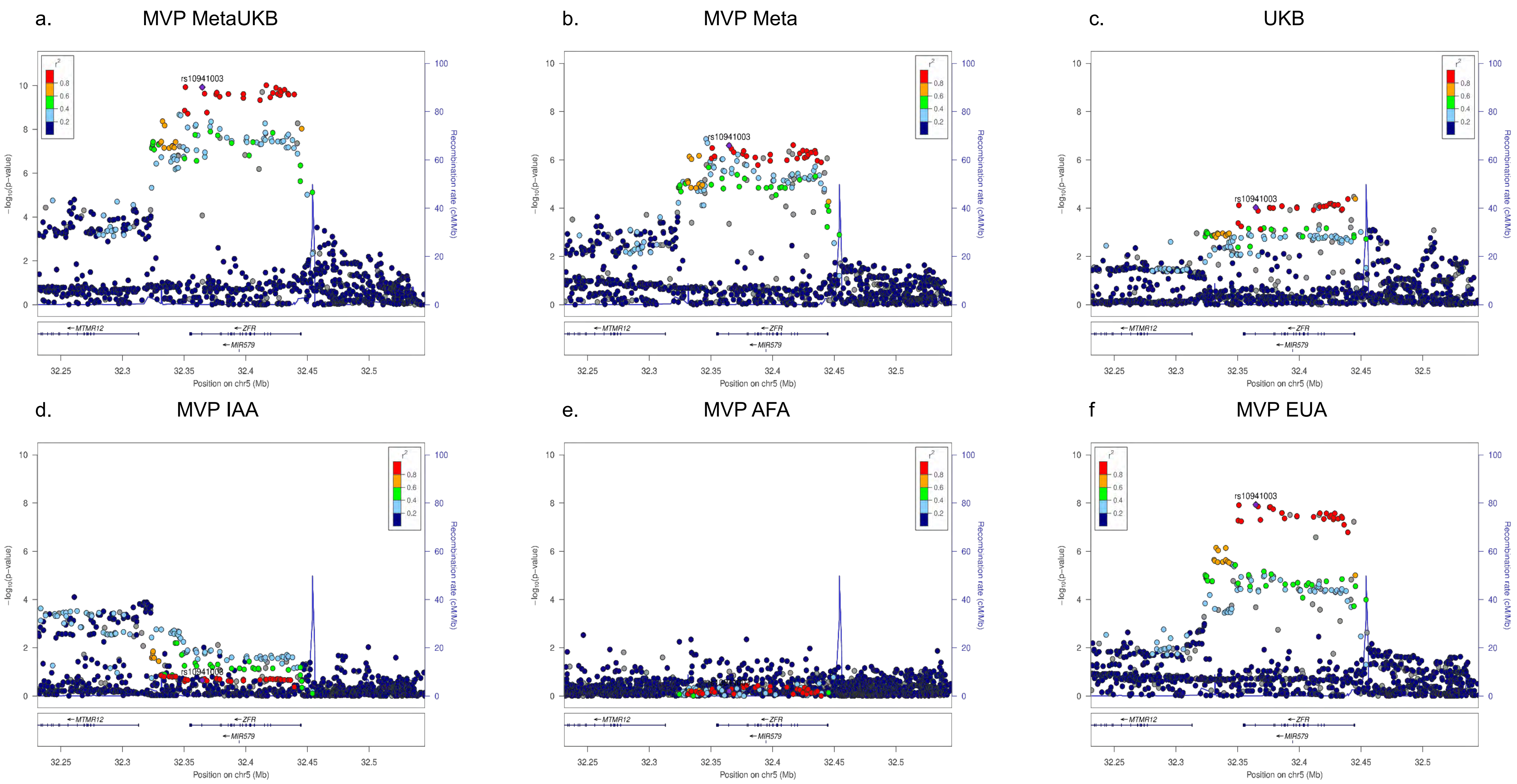

# locus035 | rs2963985

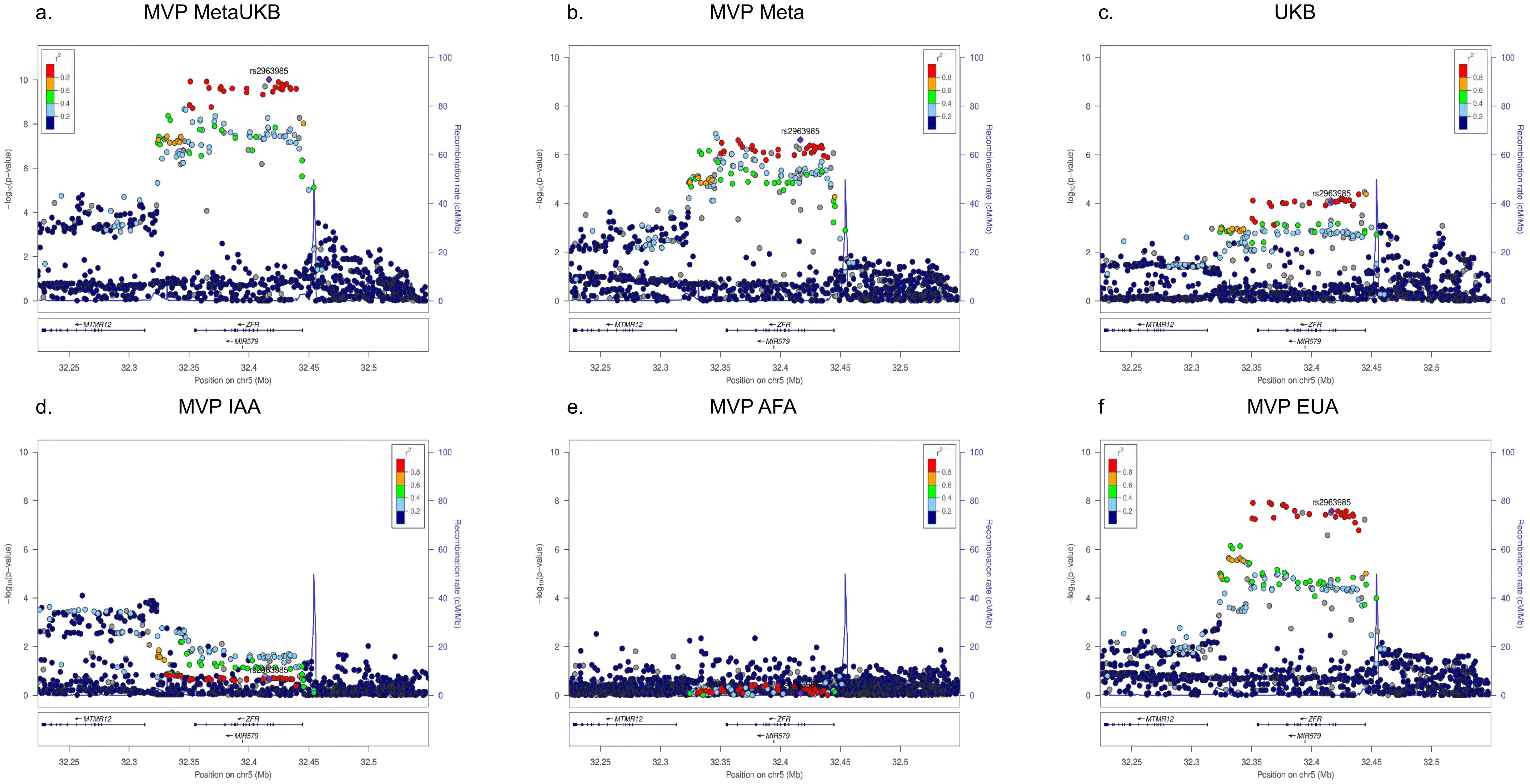

# locus036 | rs1427907

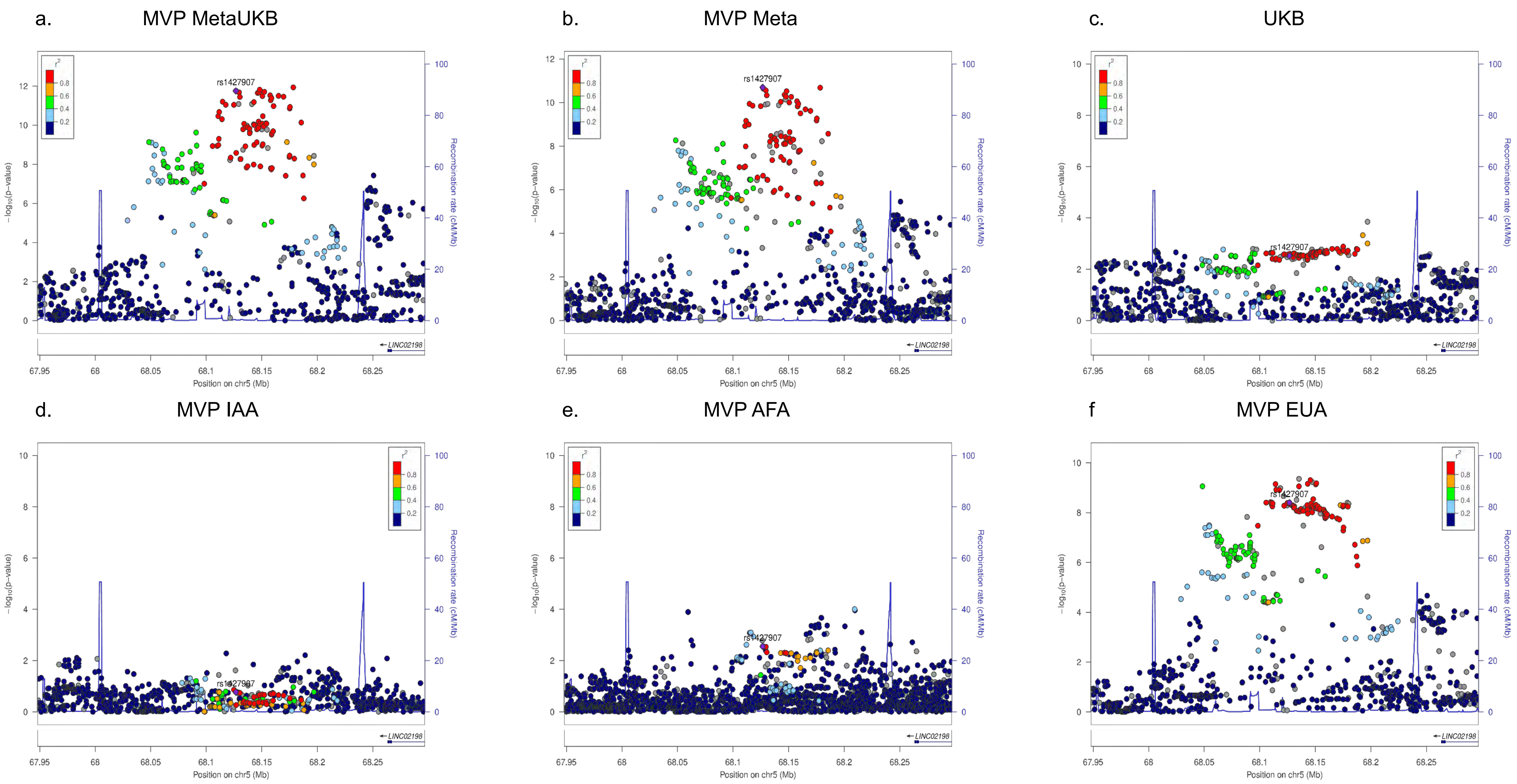

# locus036 | rs35028055

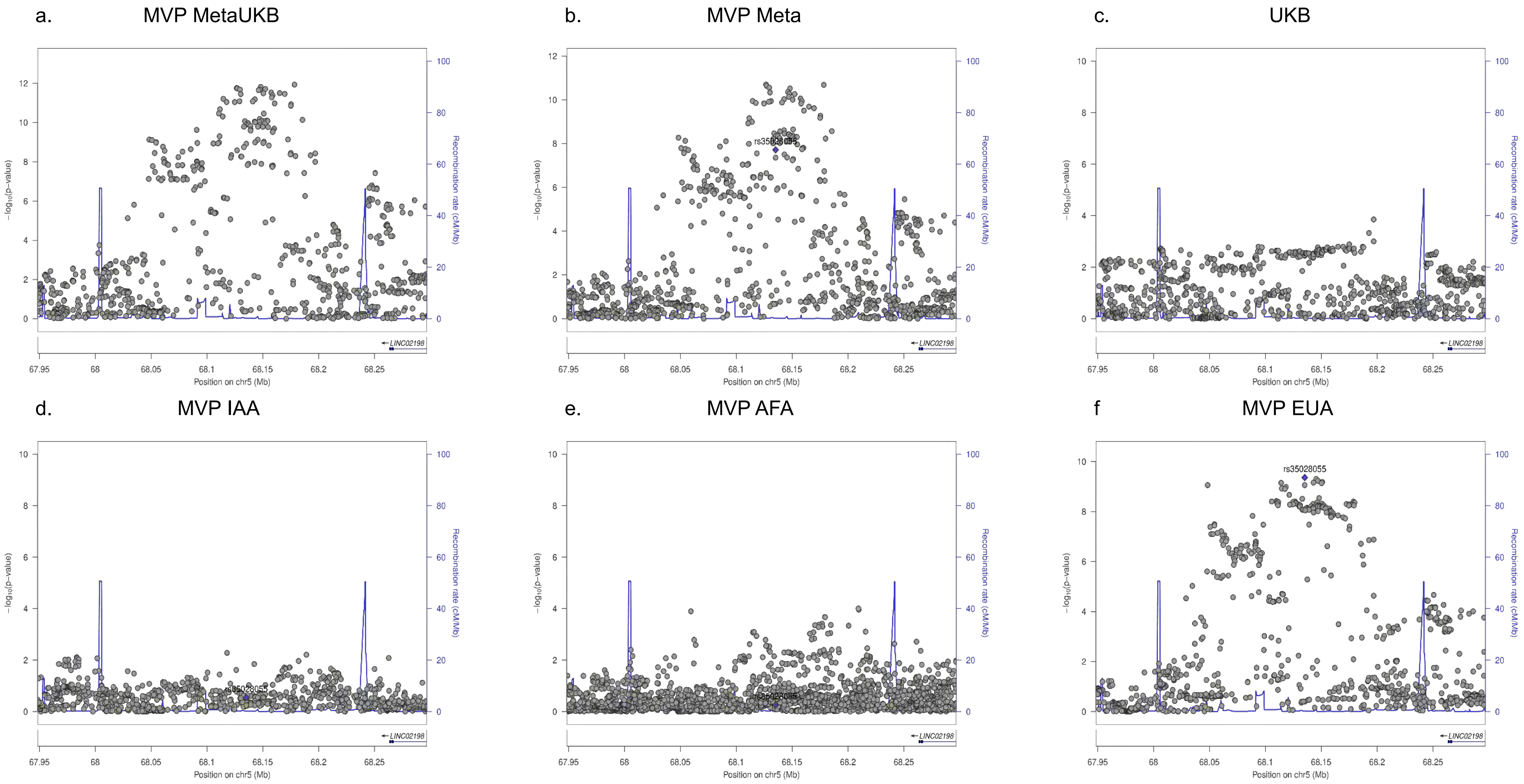

## locus036 | rs4246760

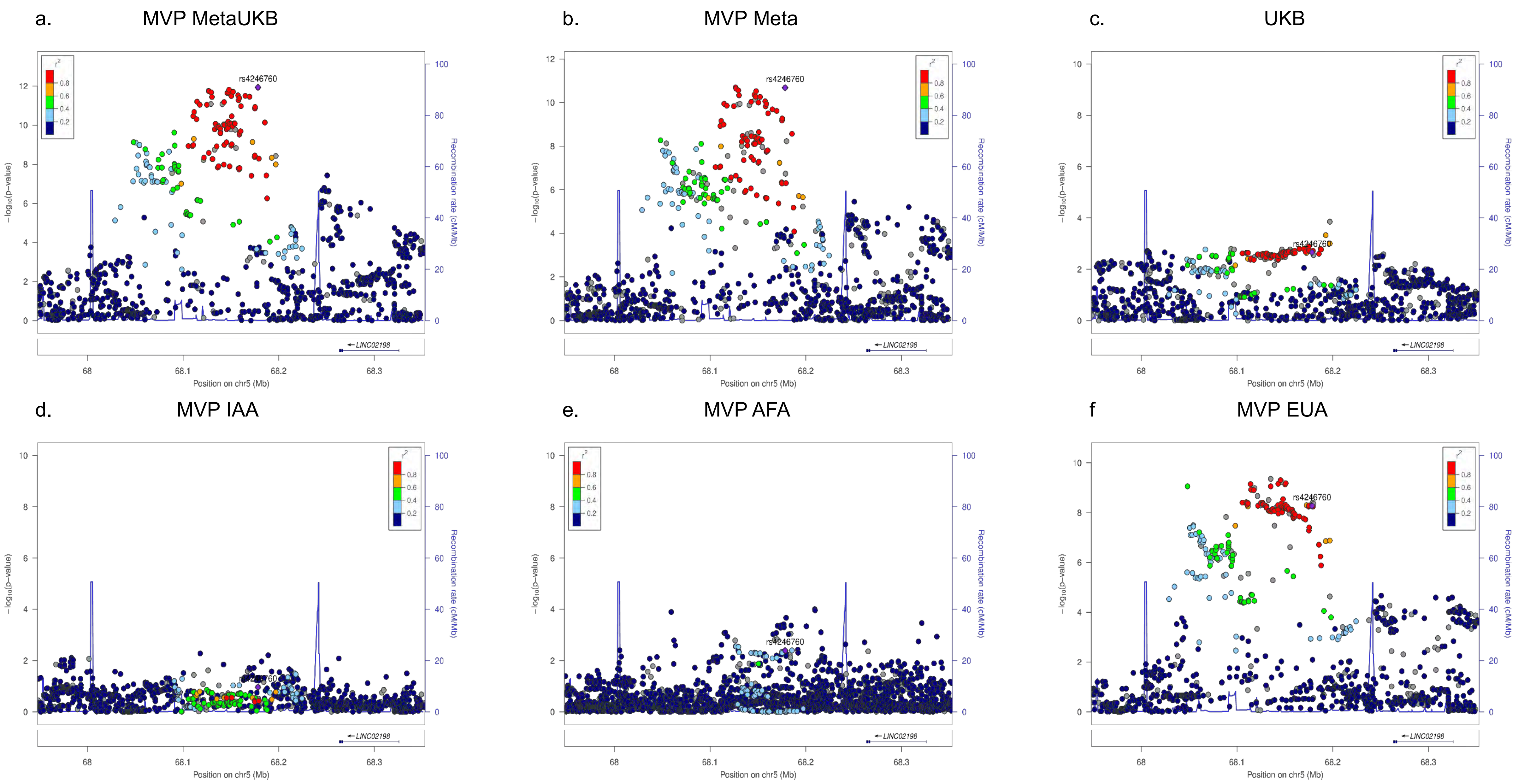

# locus037 | rs6453022

a. MVP MetaUKB

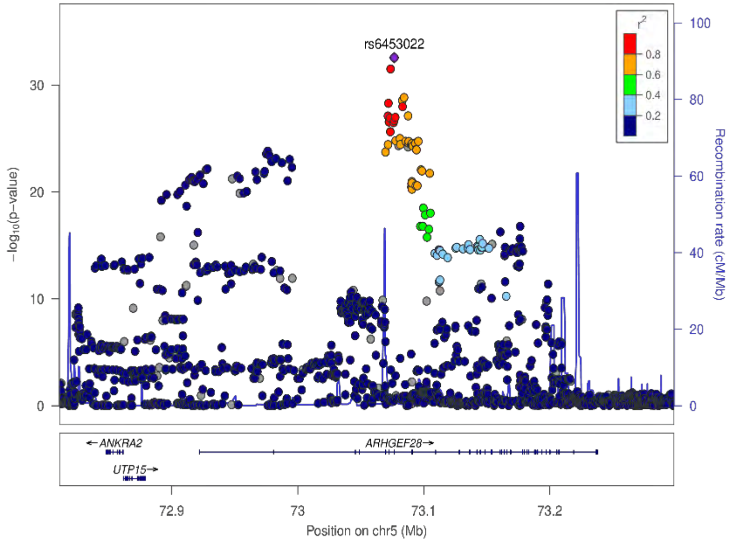


b. MVP Meta

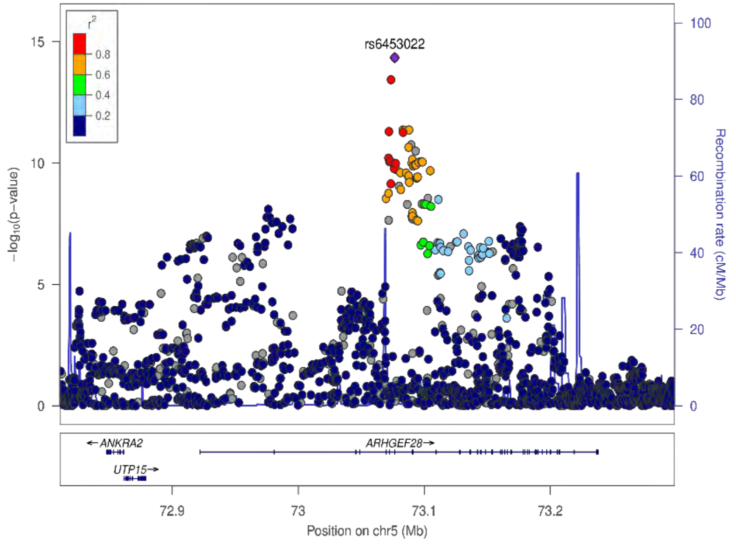


c. UKB

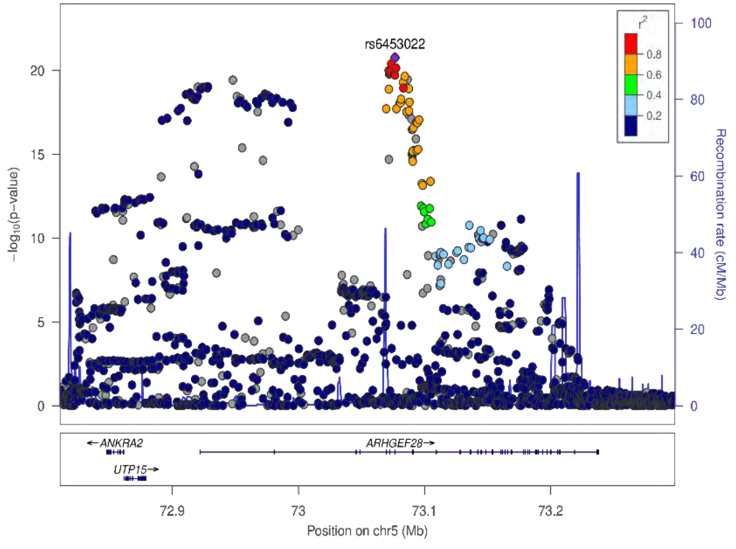


d. MVP IAA

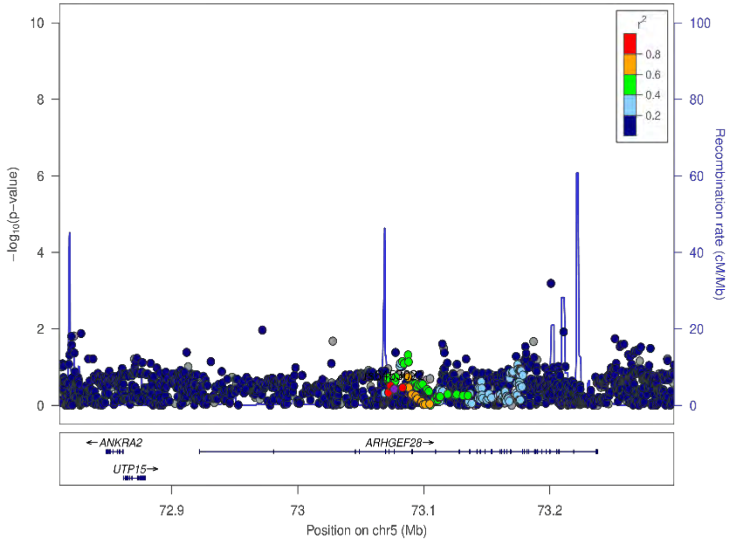


e. MVP AFA

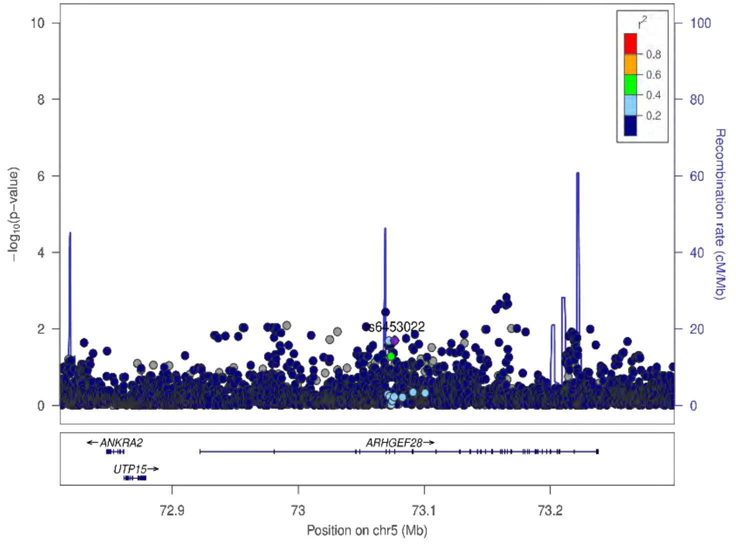


f MVP EUA

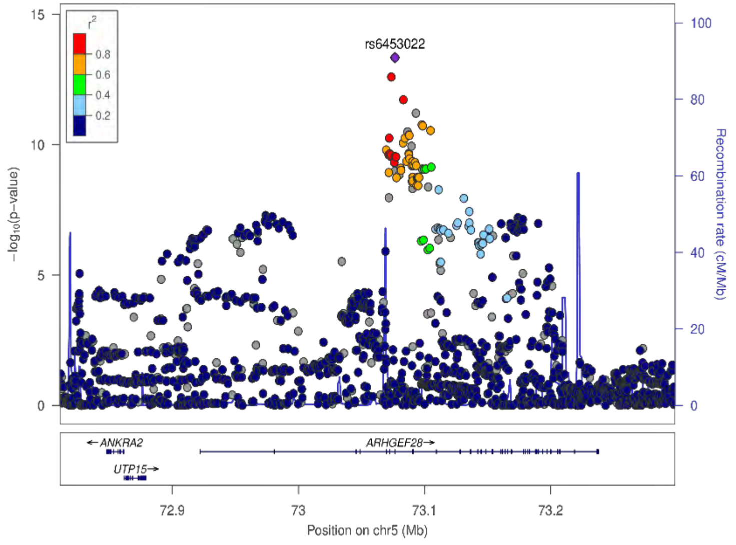

# locus038 | rs2386237

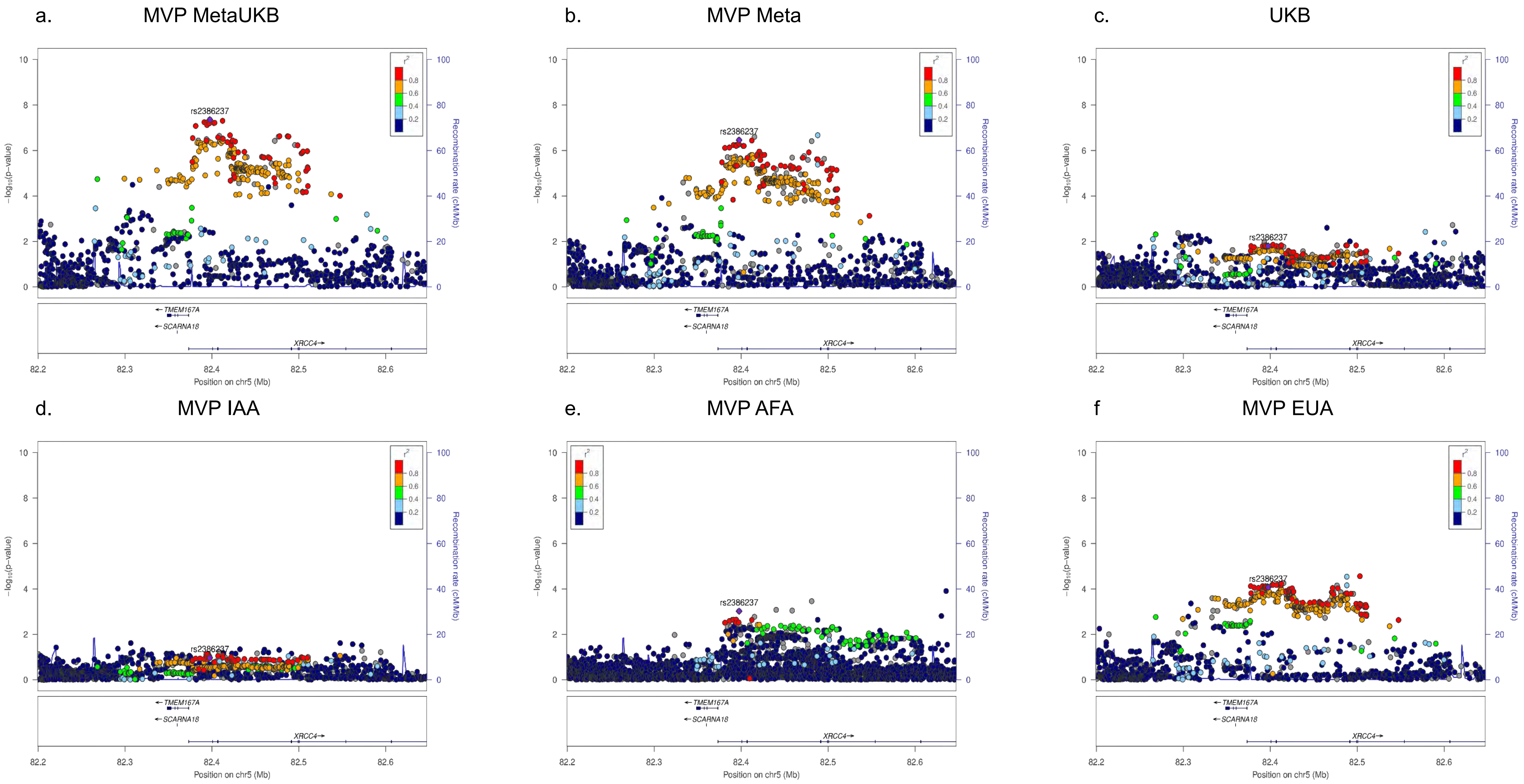

# locus039 | rs10514301

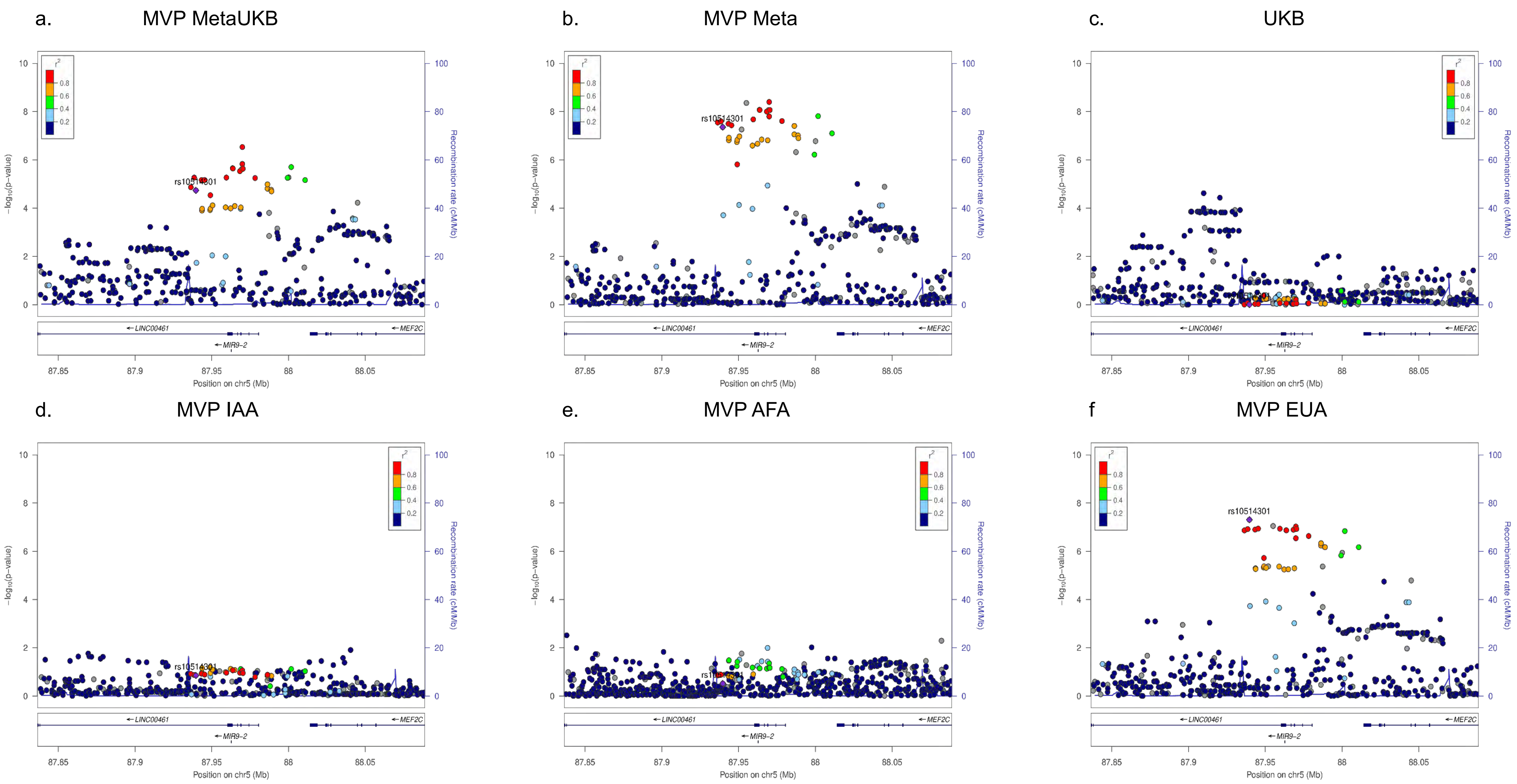

# locus039 | rs1846974

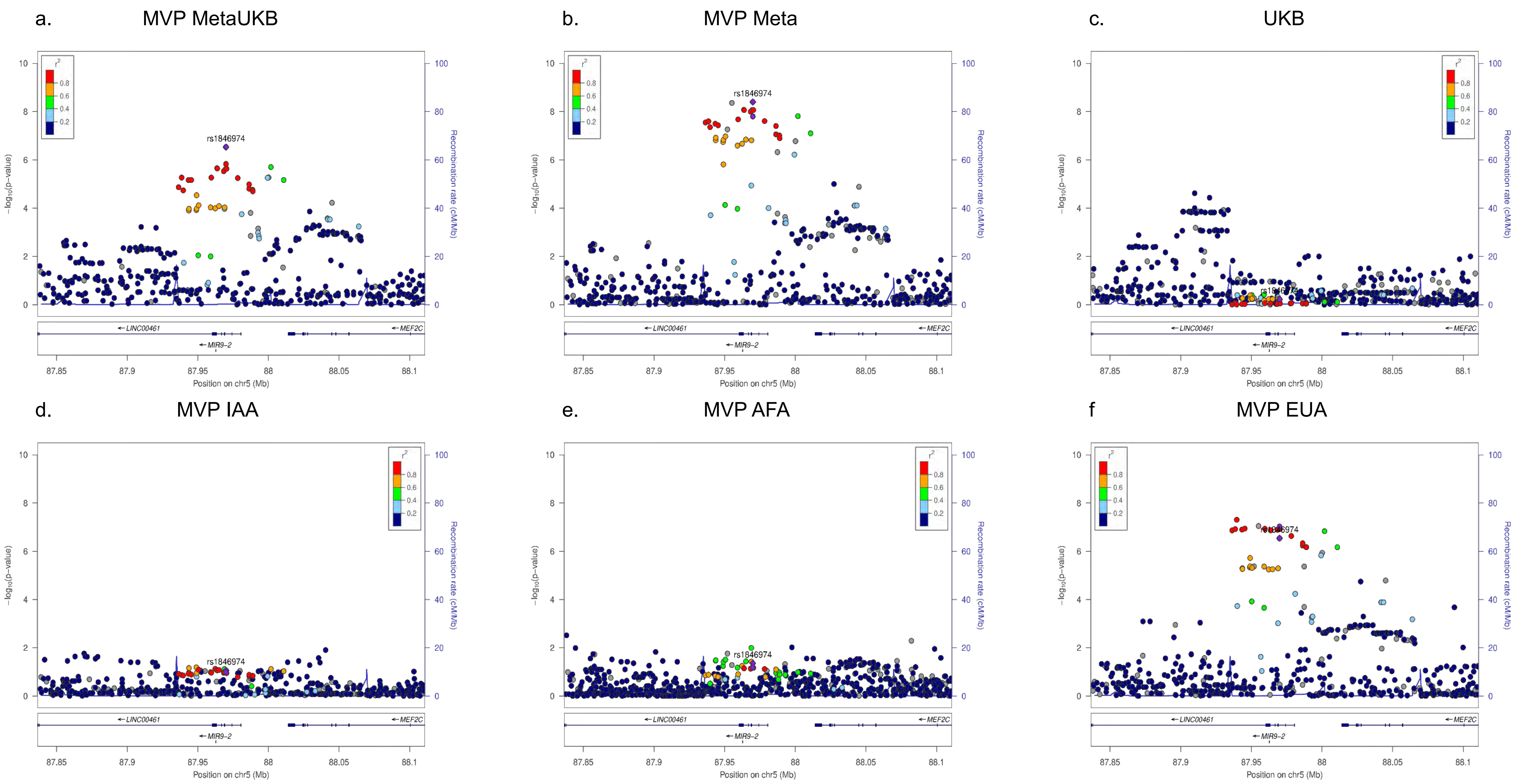

# locus040 | rs167570

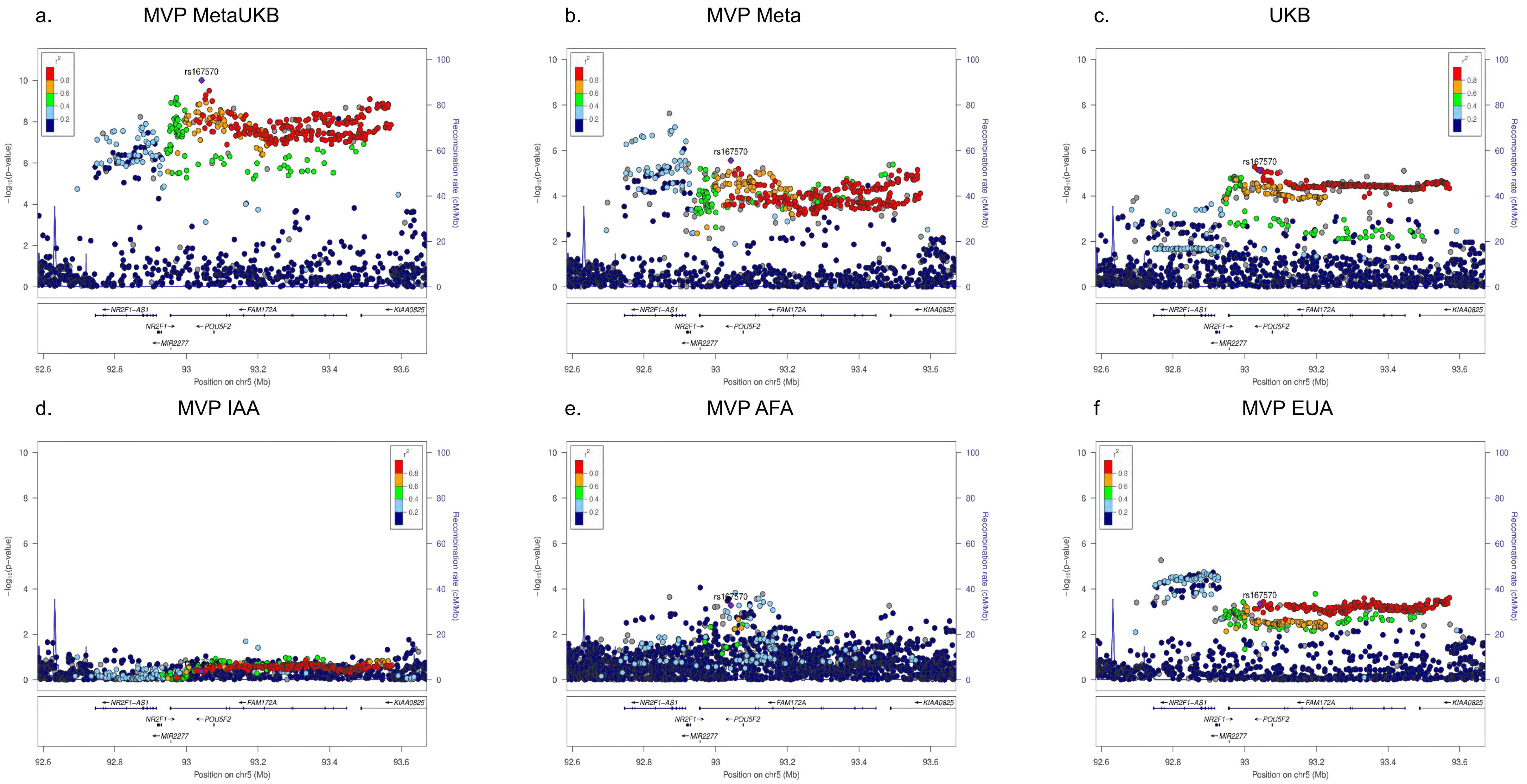

# locus040 | rs566192265

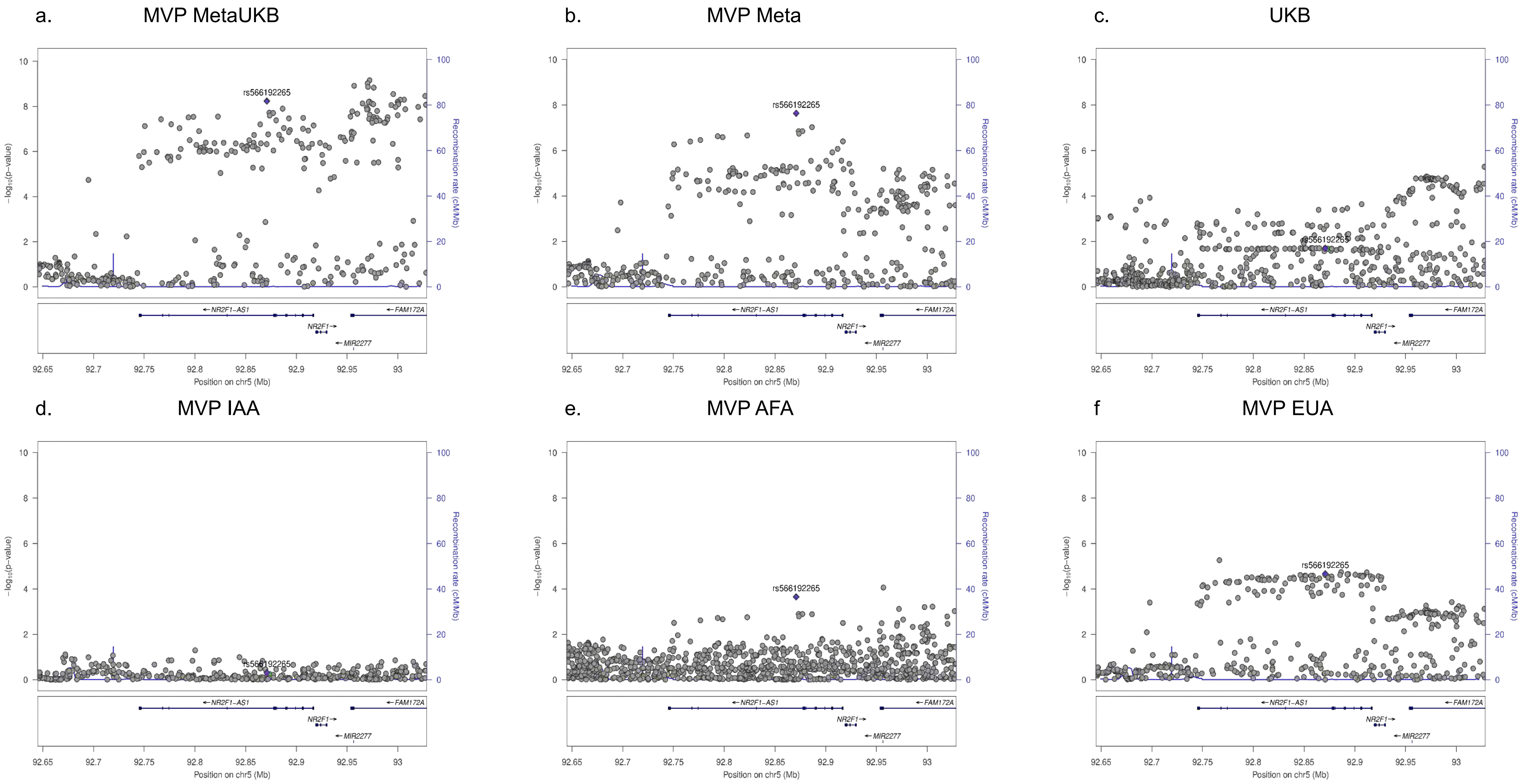

# locus041 | rs34442808

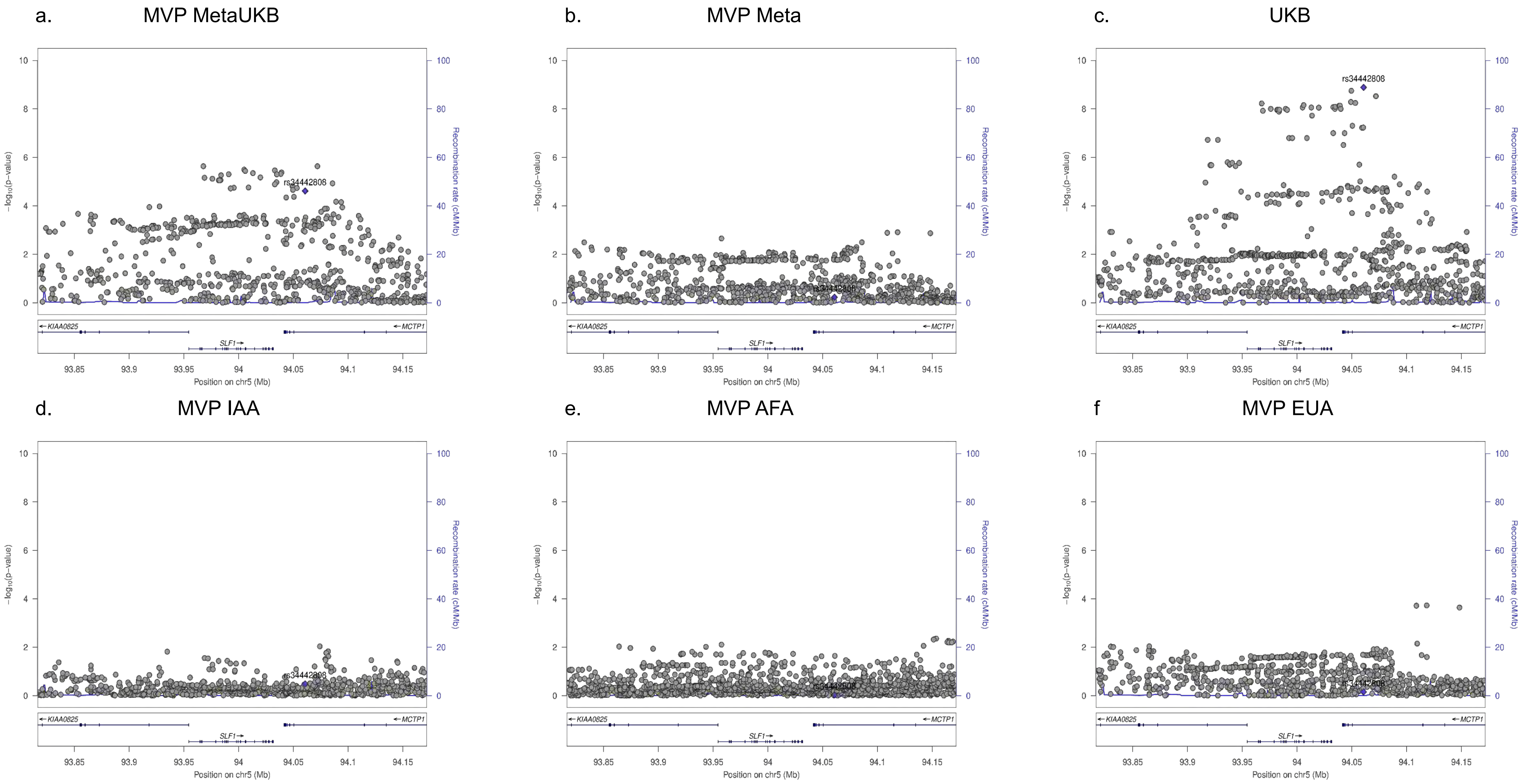

# locus042 | rs9688110

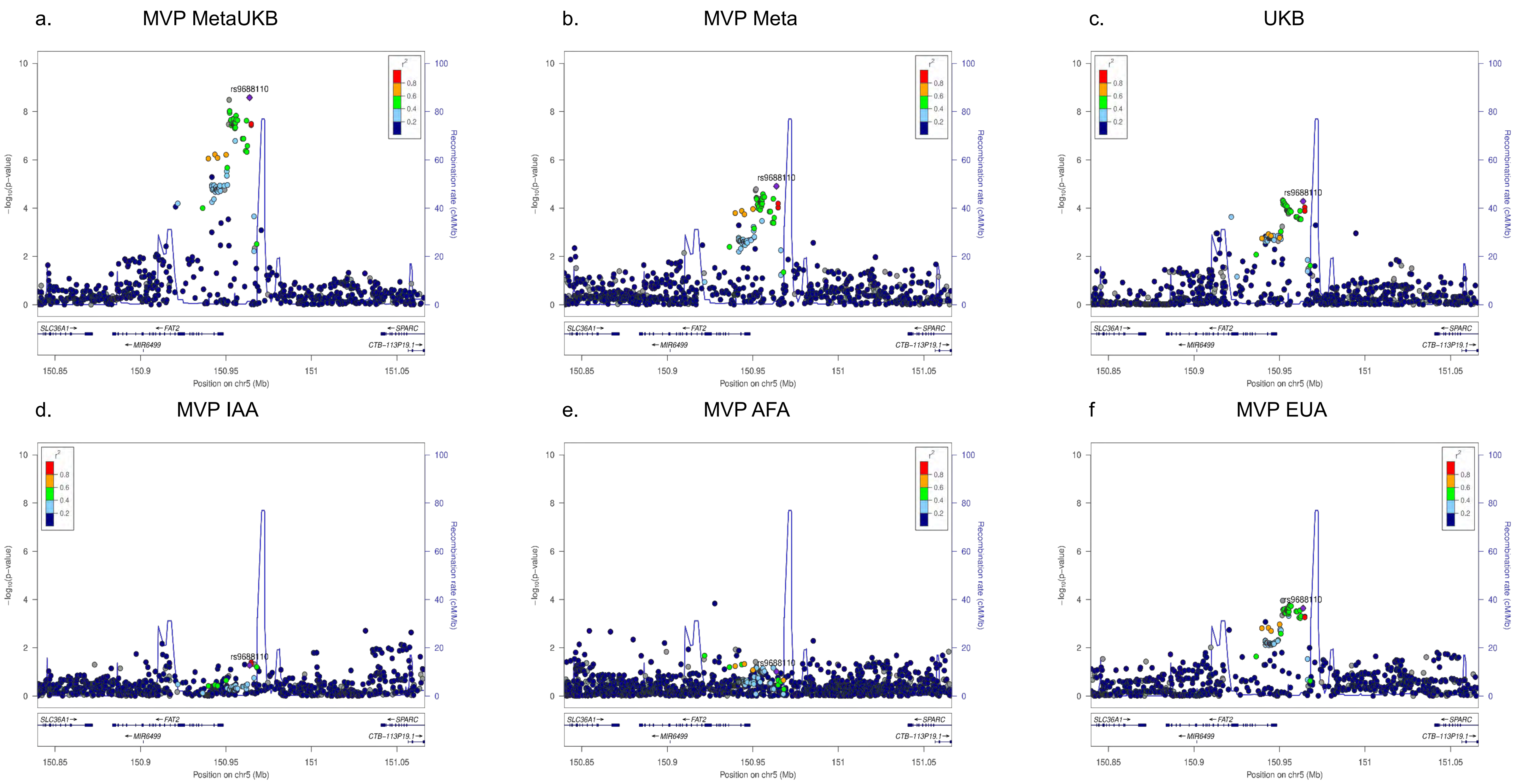

# locus043 | rs3074558

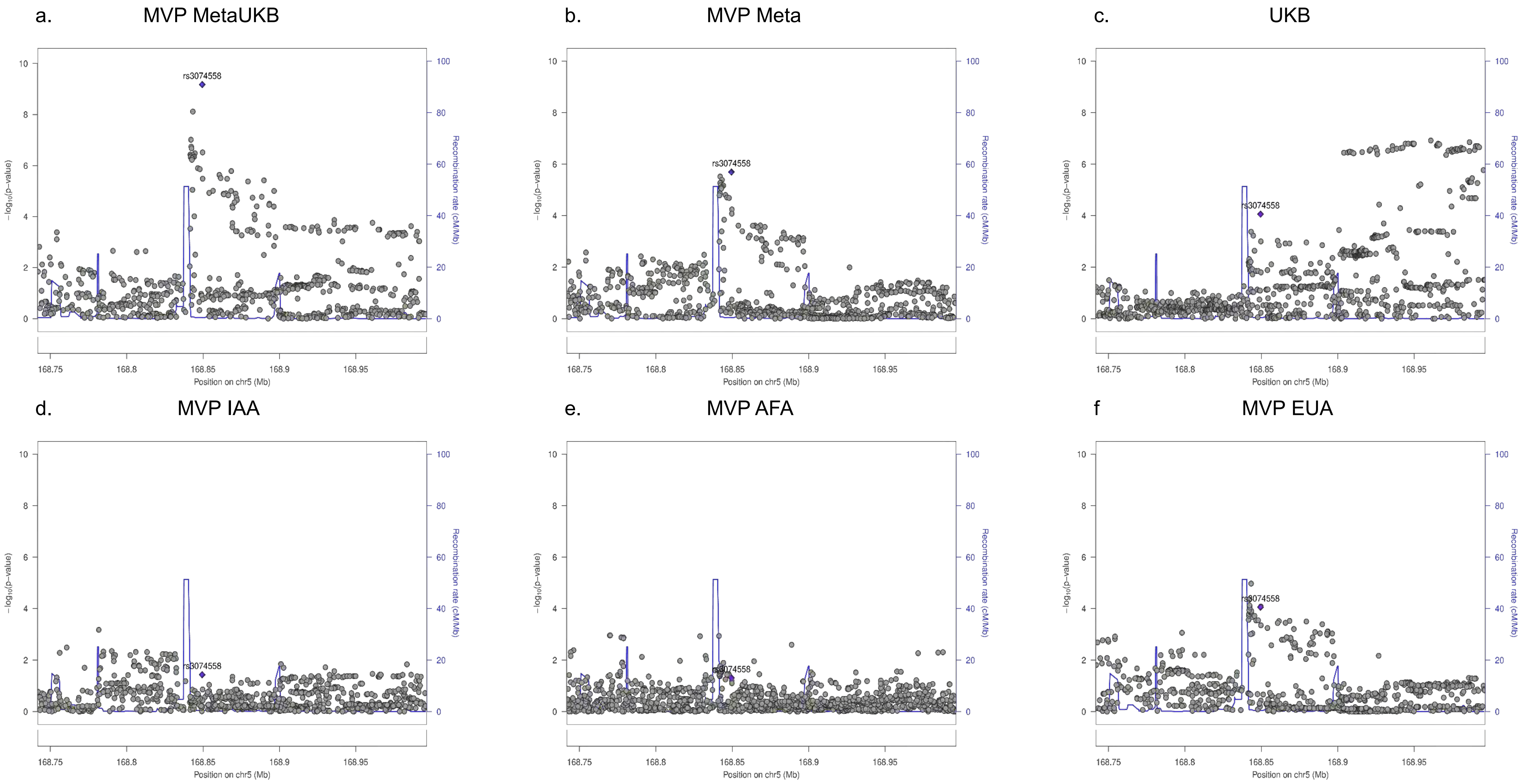

# locus044 | rs11243143

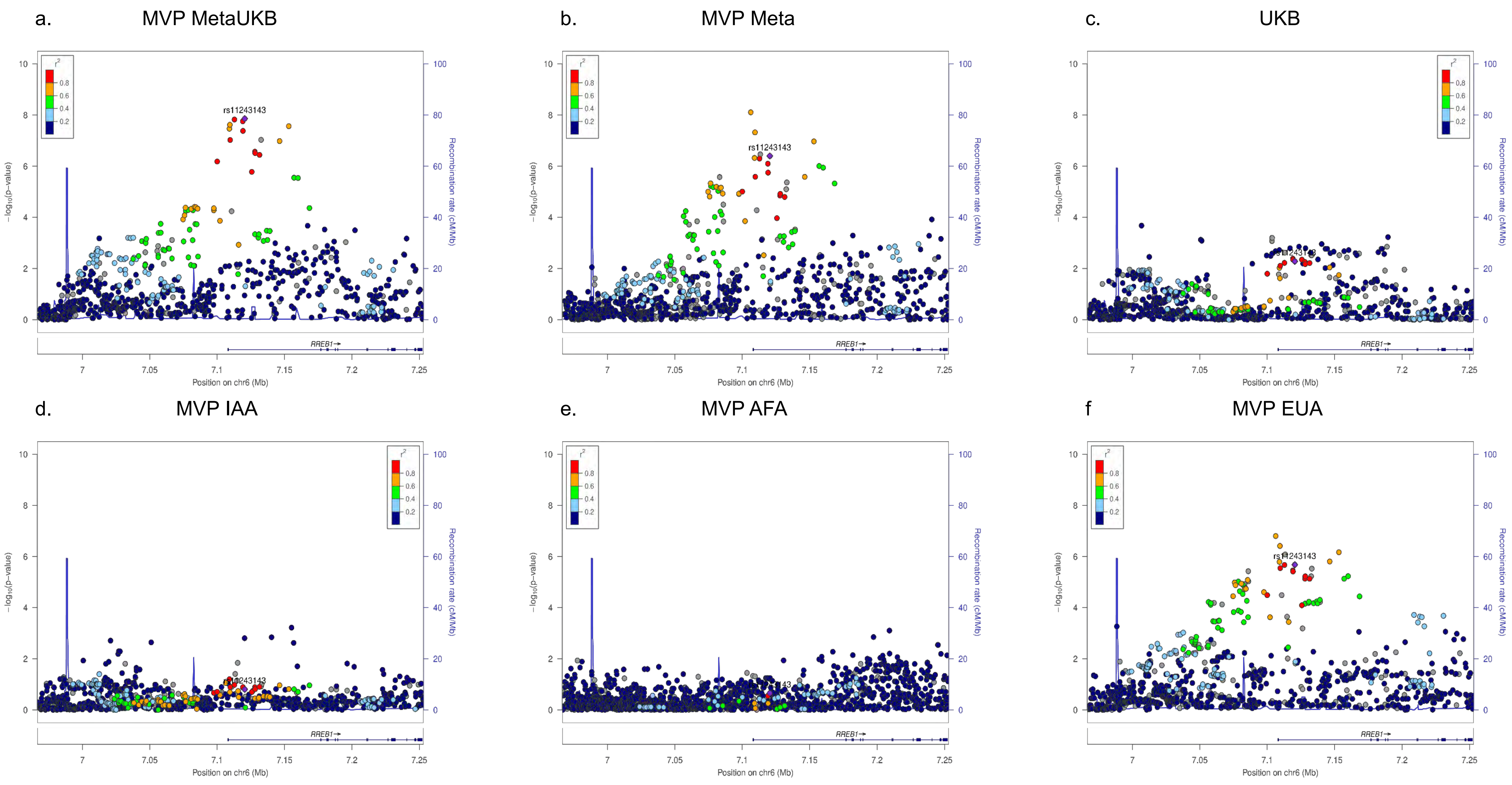

# locus044 | rs2842895

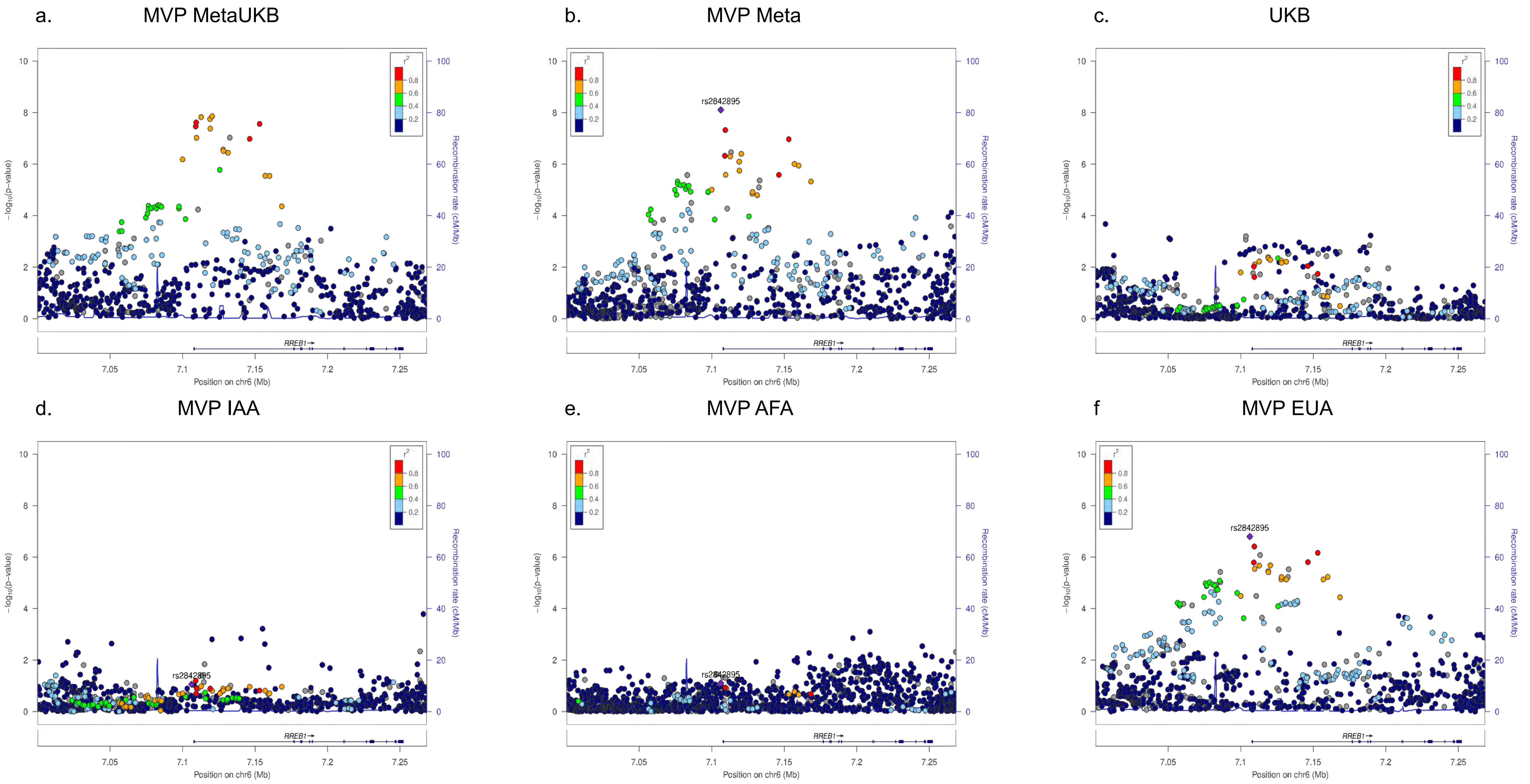

# locus045 | rs1928176

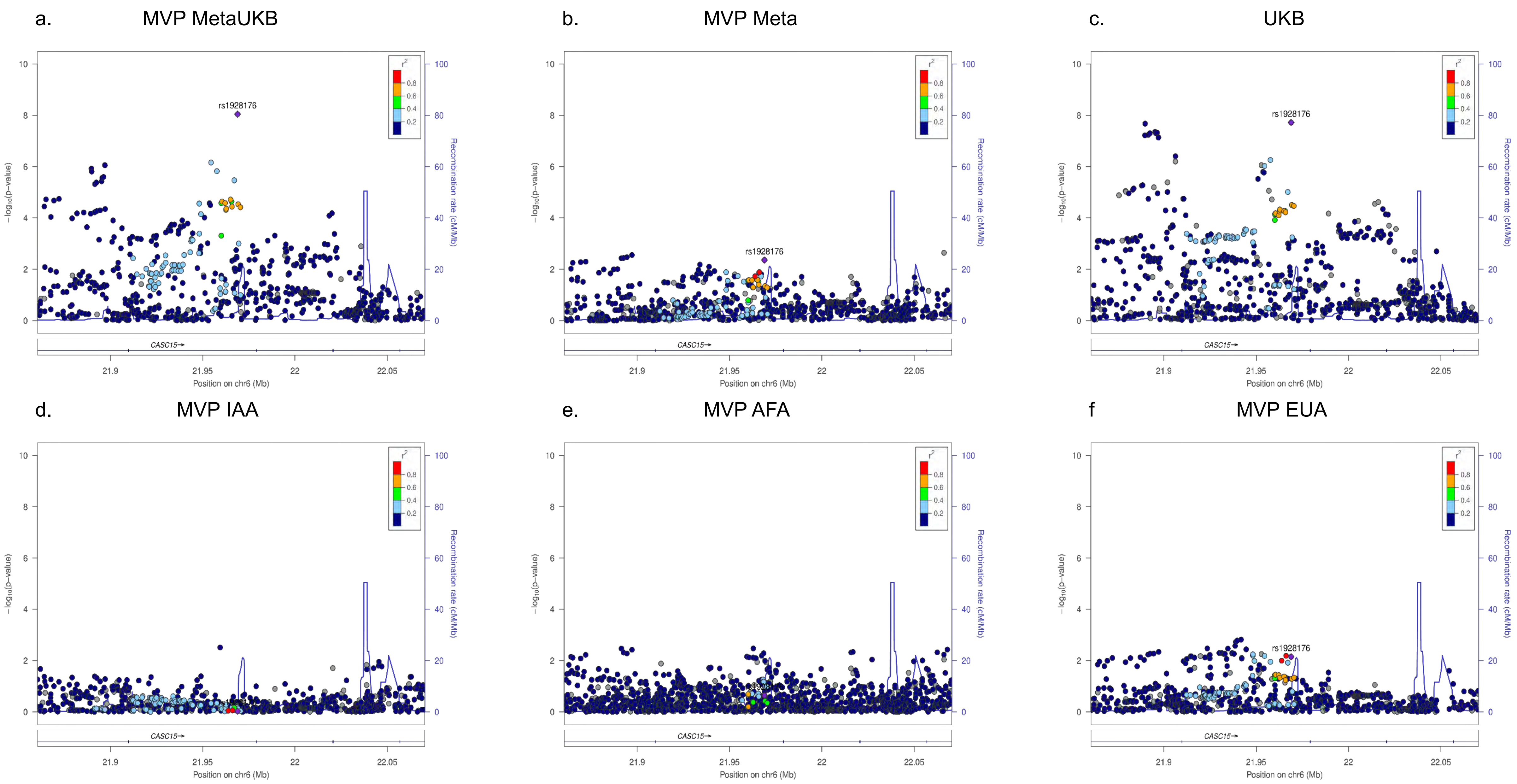

# locus046 | rs2765220

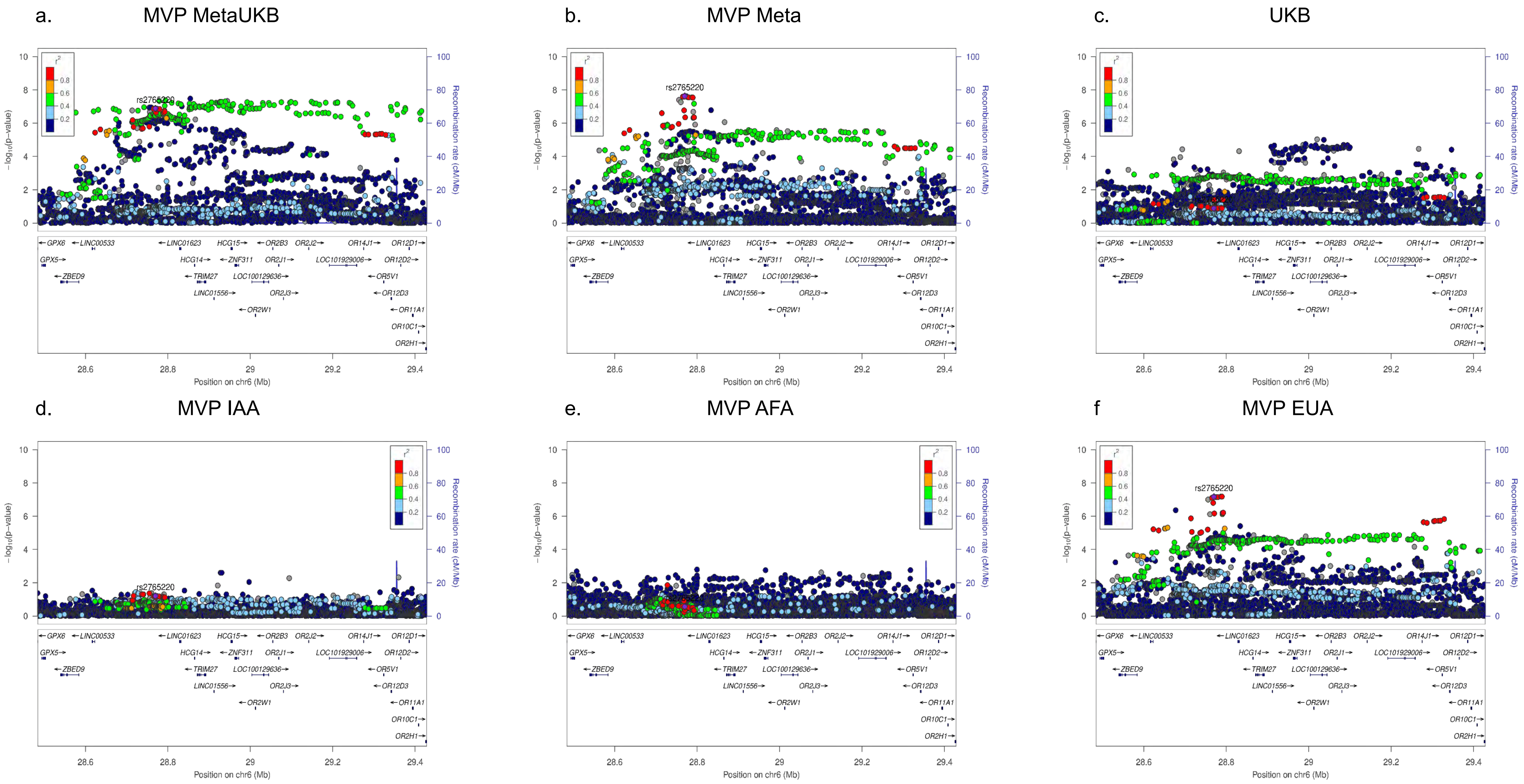

# locus046 | rs66868086

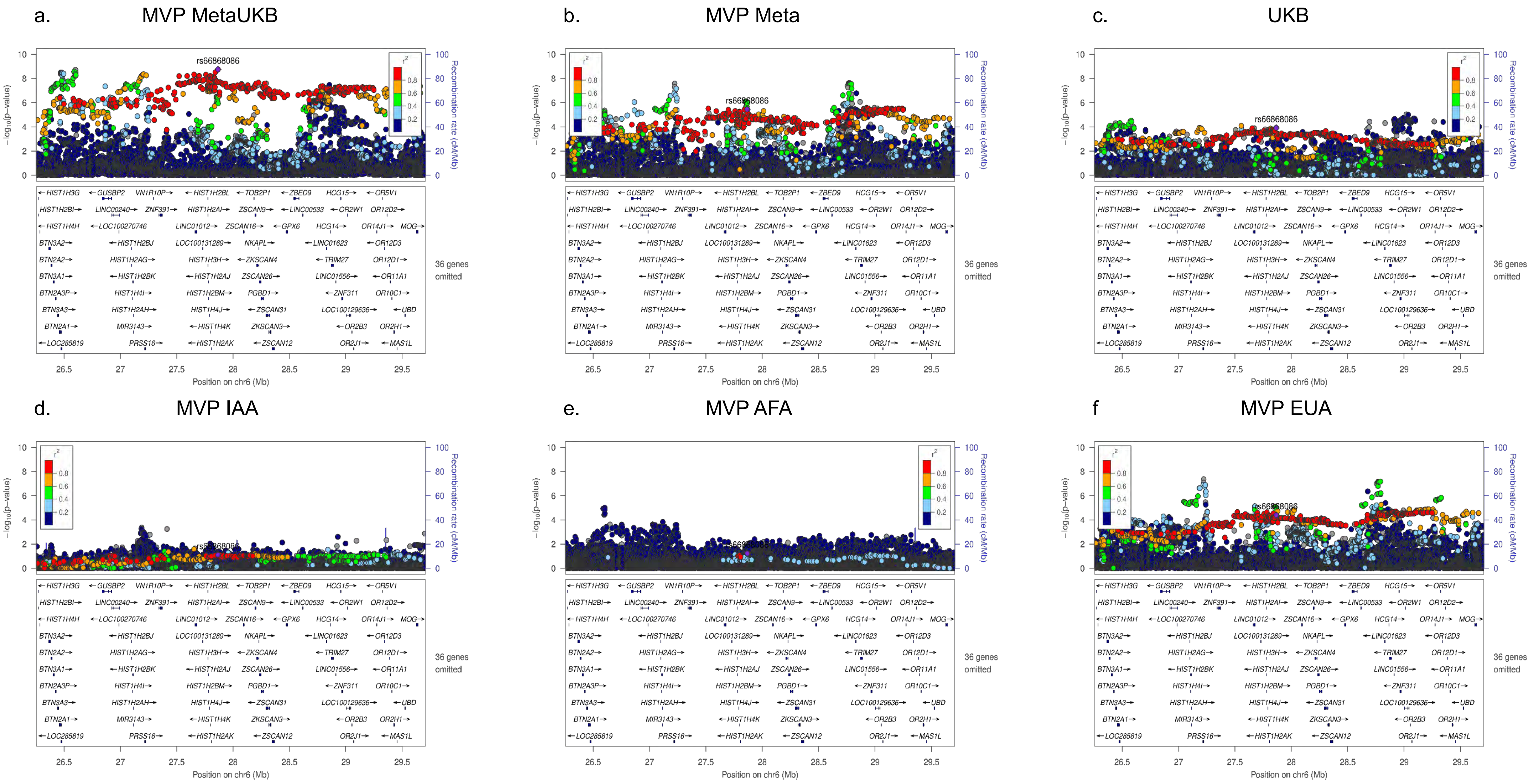

# locus047 | rs1574430

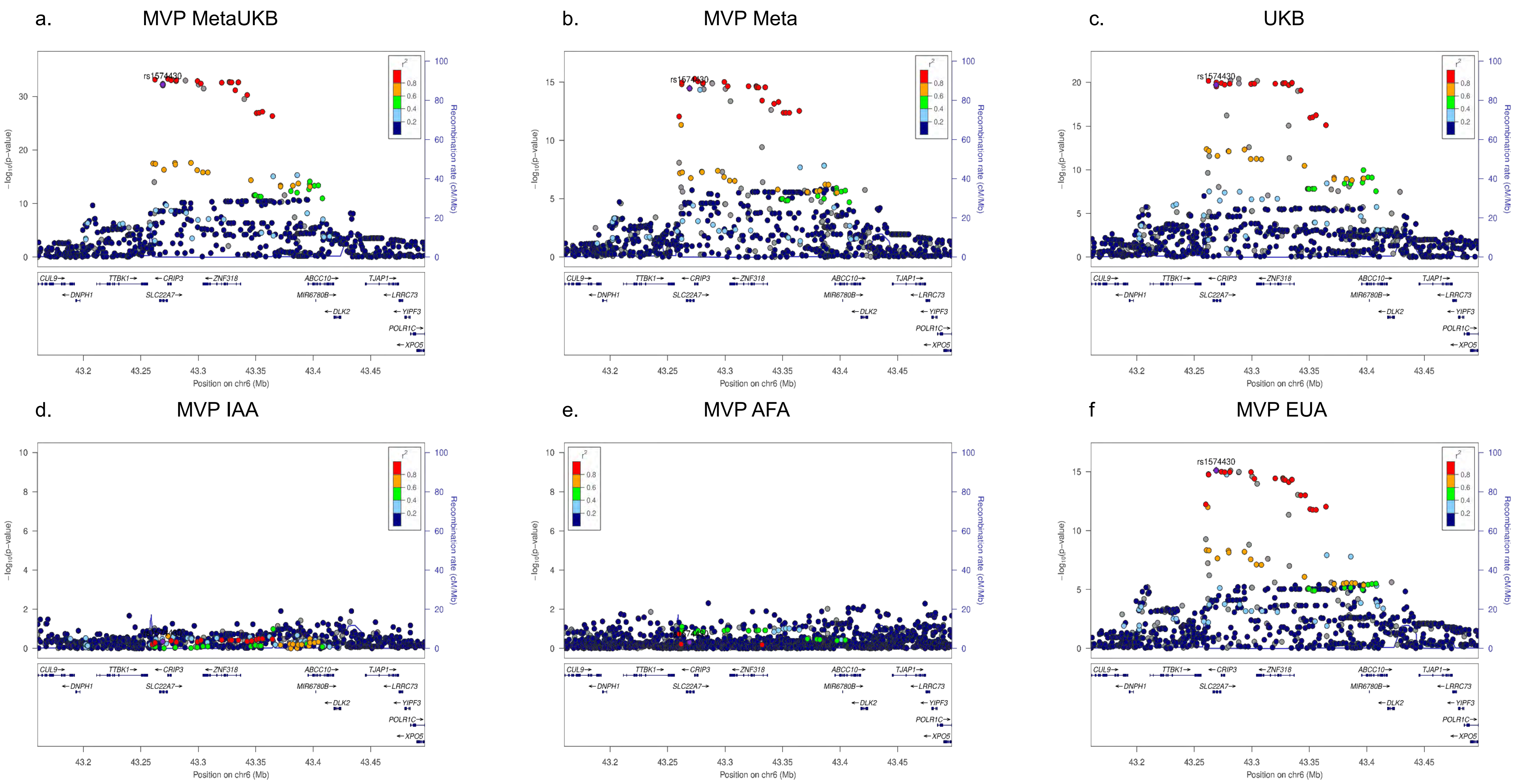

# locus047 | rs2242416

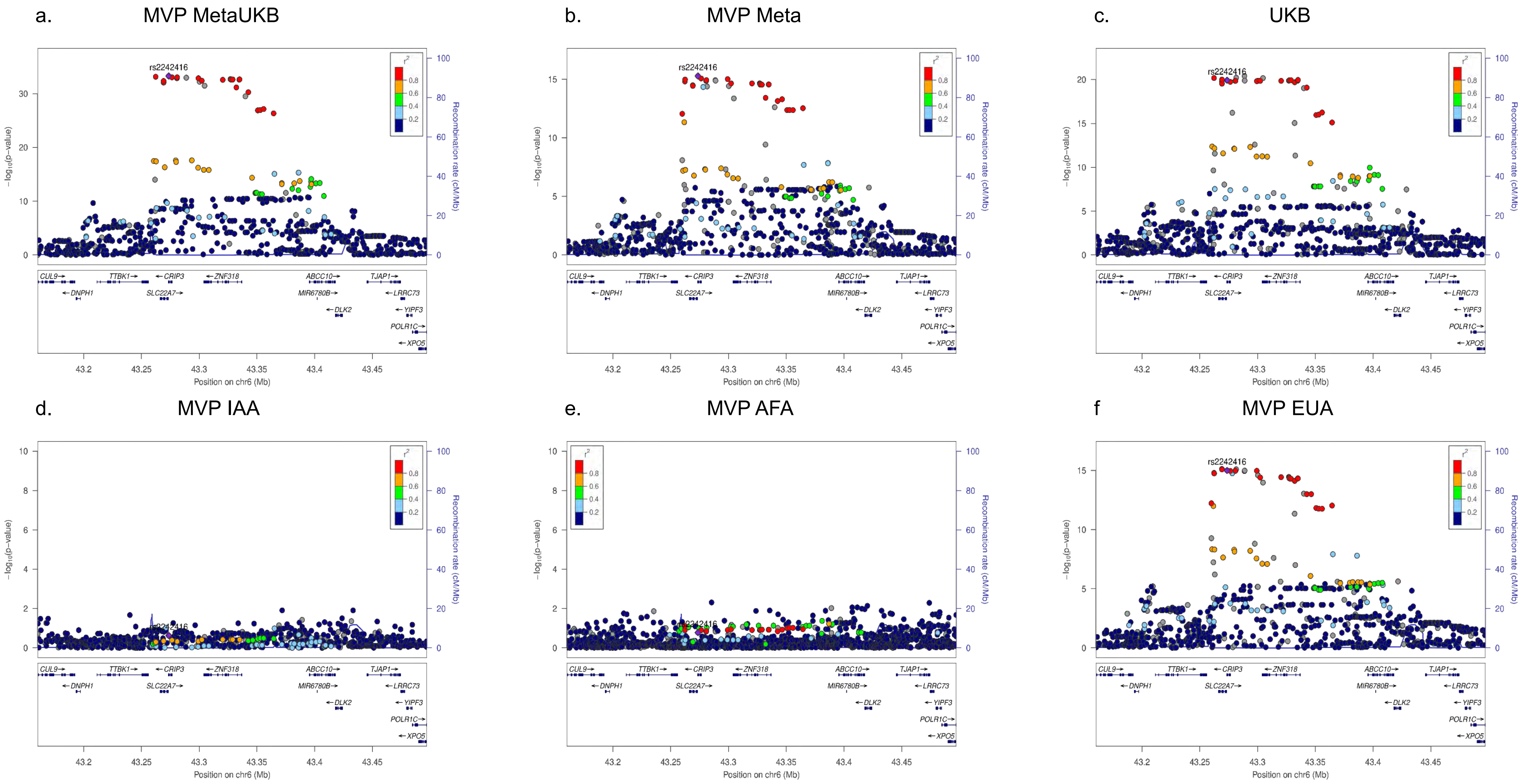

# locus047 | rs553448379

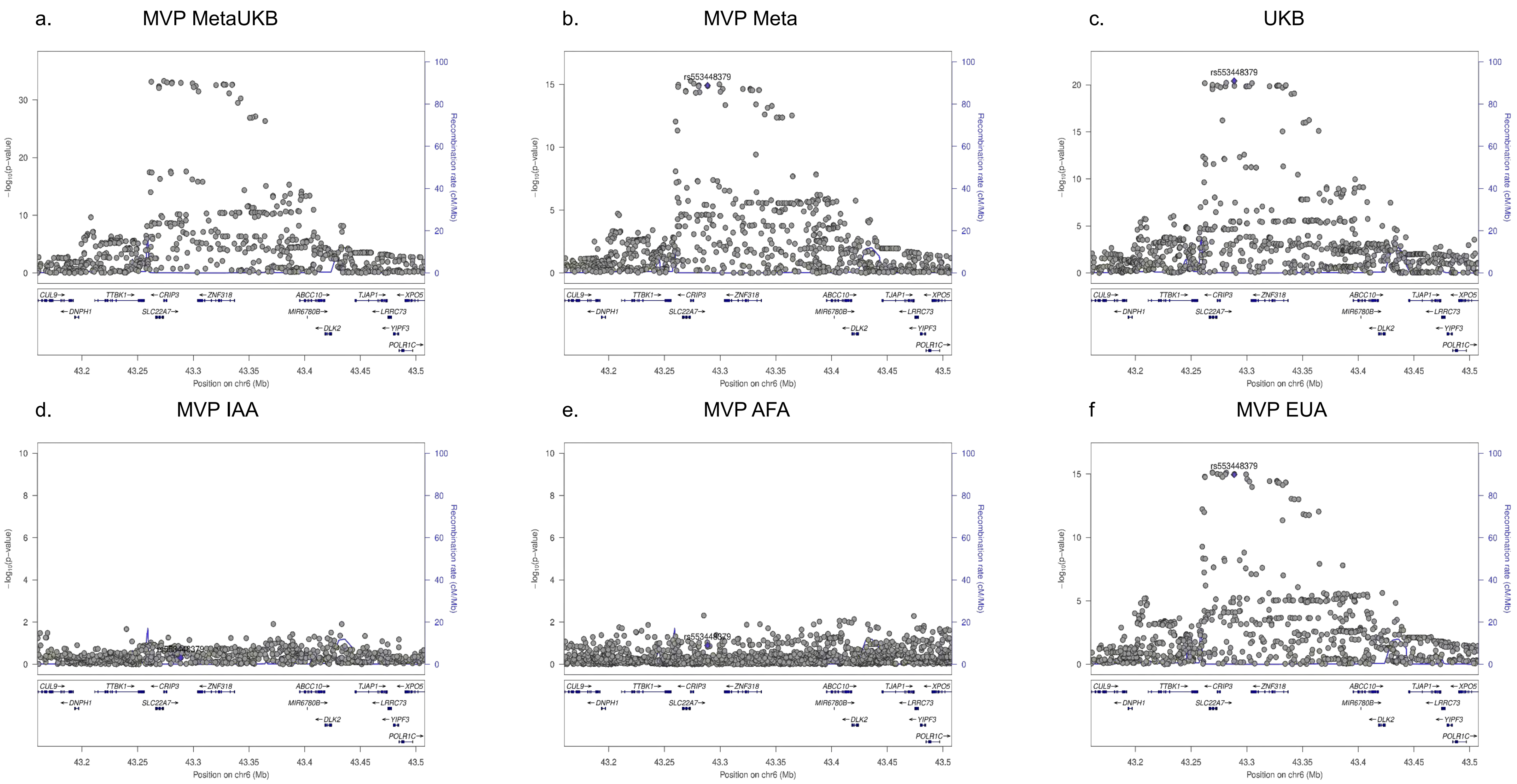

# locus048 | rs3777621

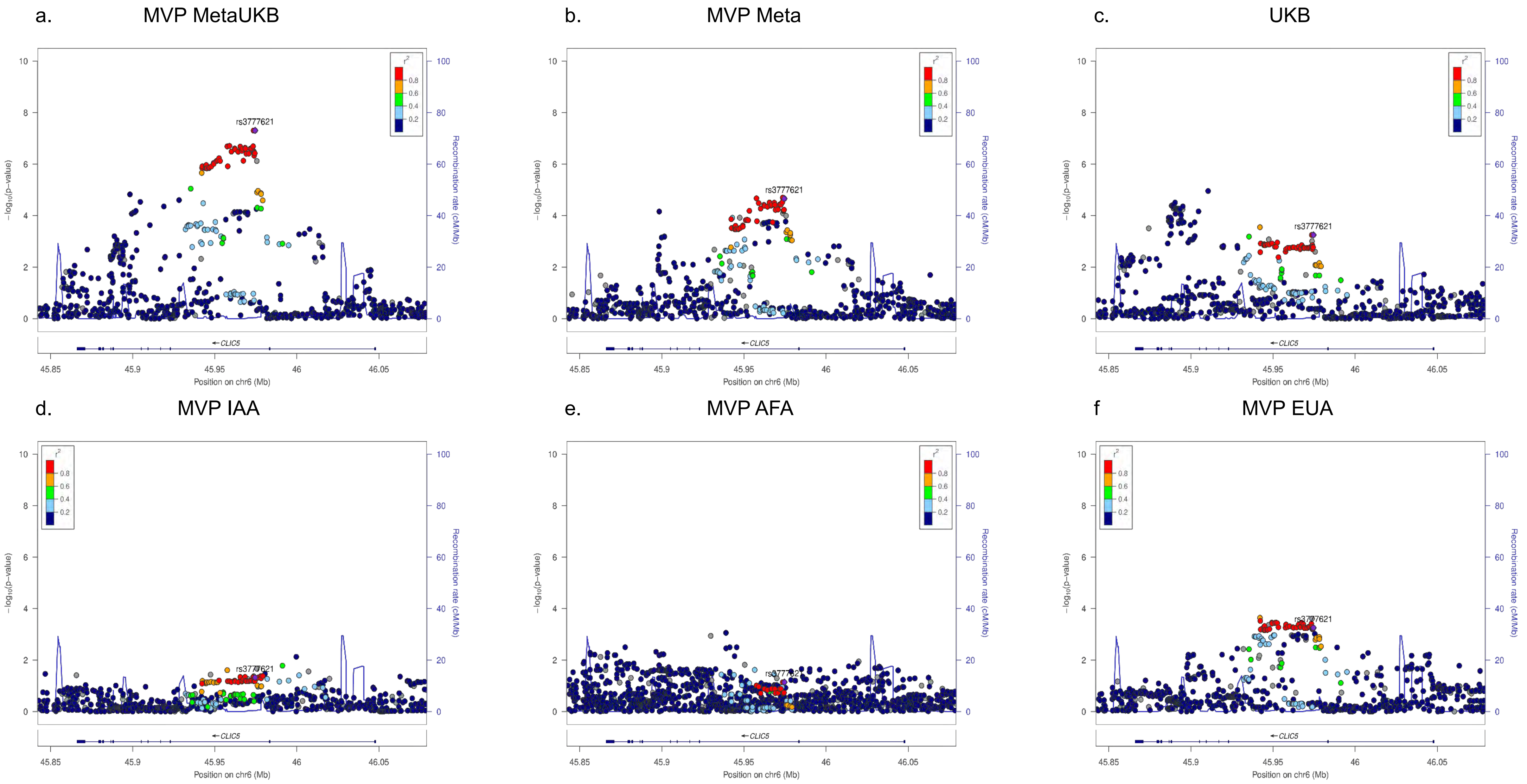

# locus049 | rs2038297

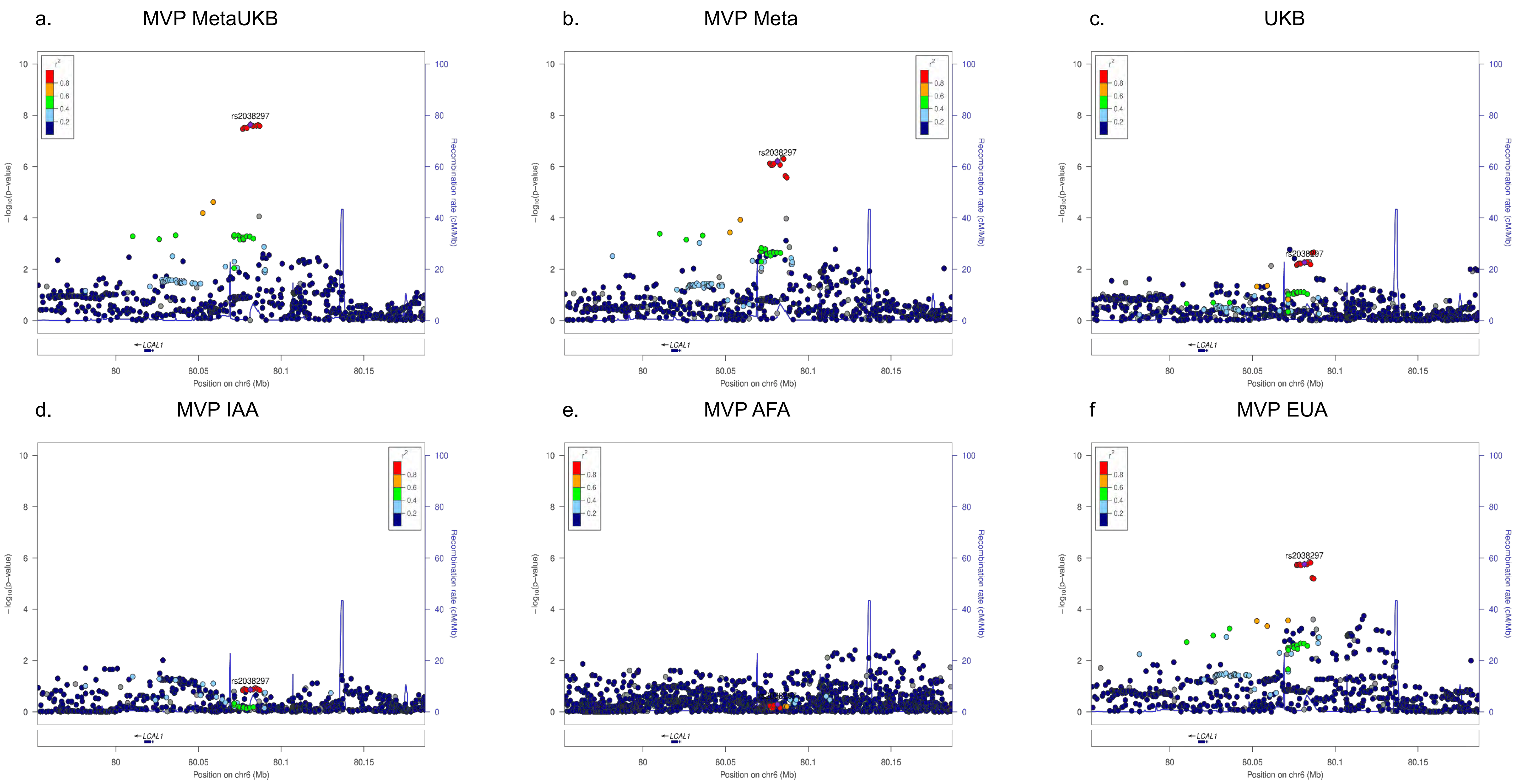

# locus050 | rs217287

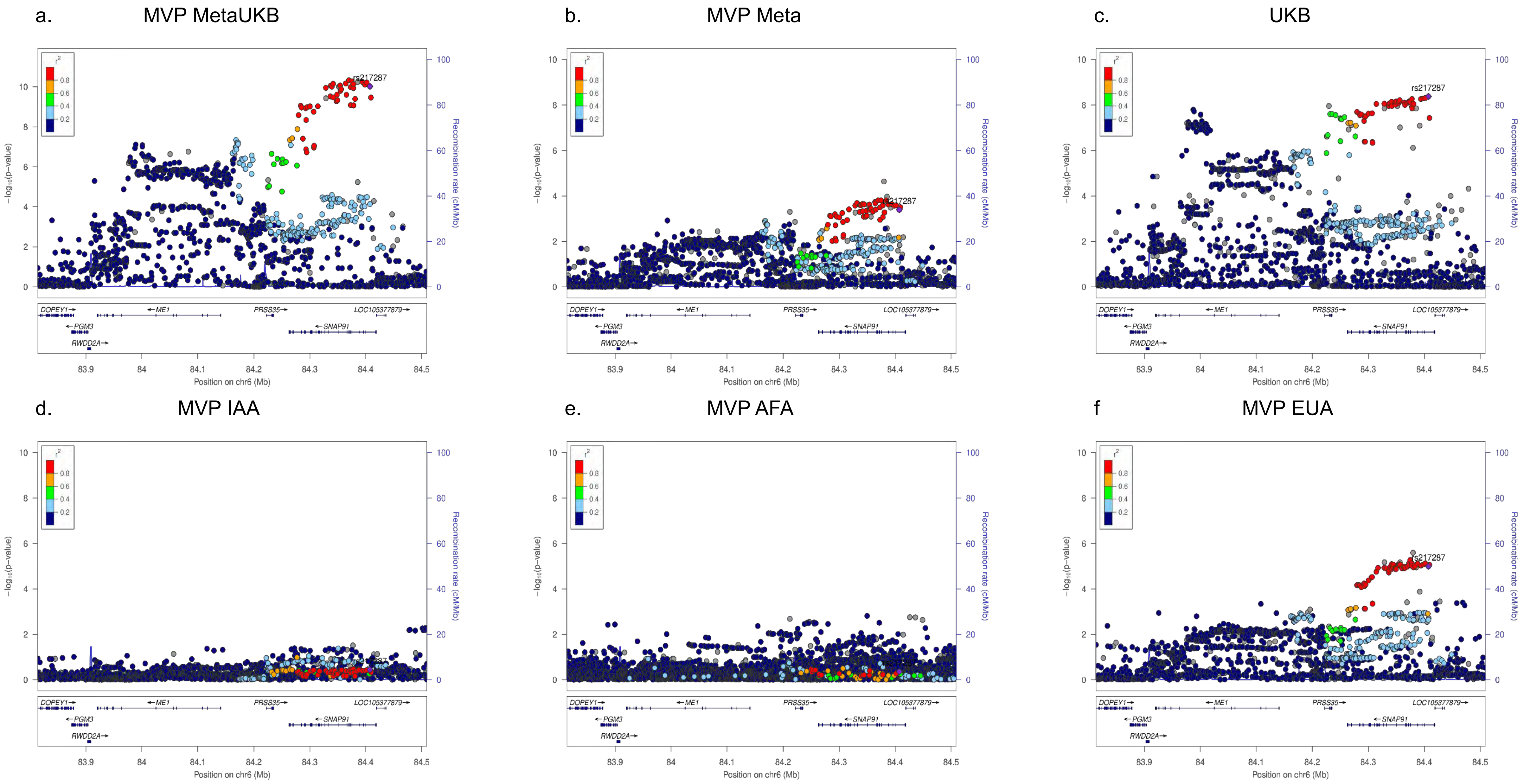

# locus050 | rs217308

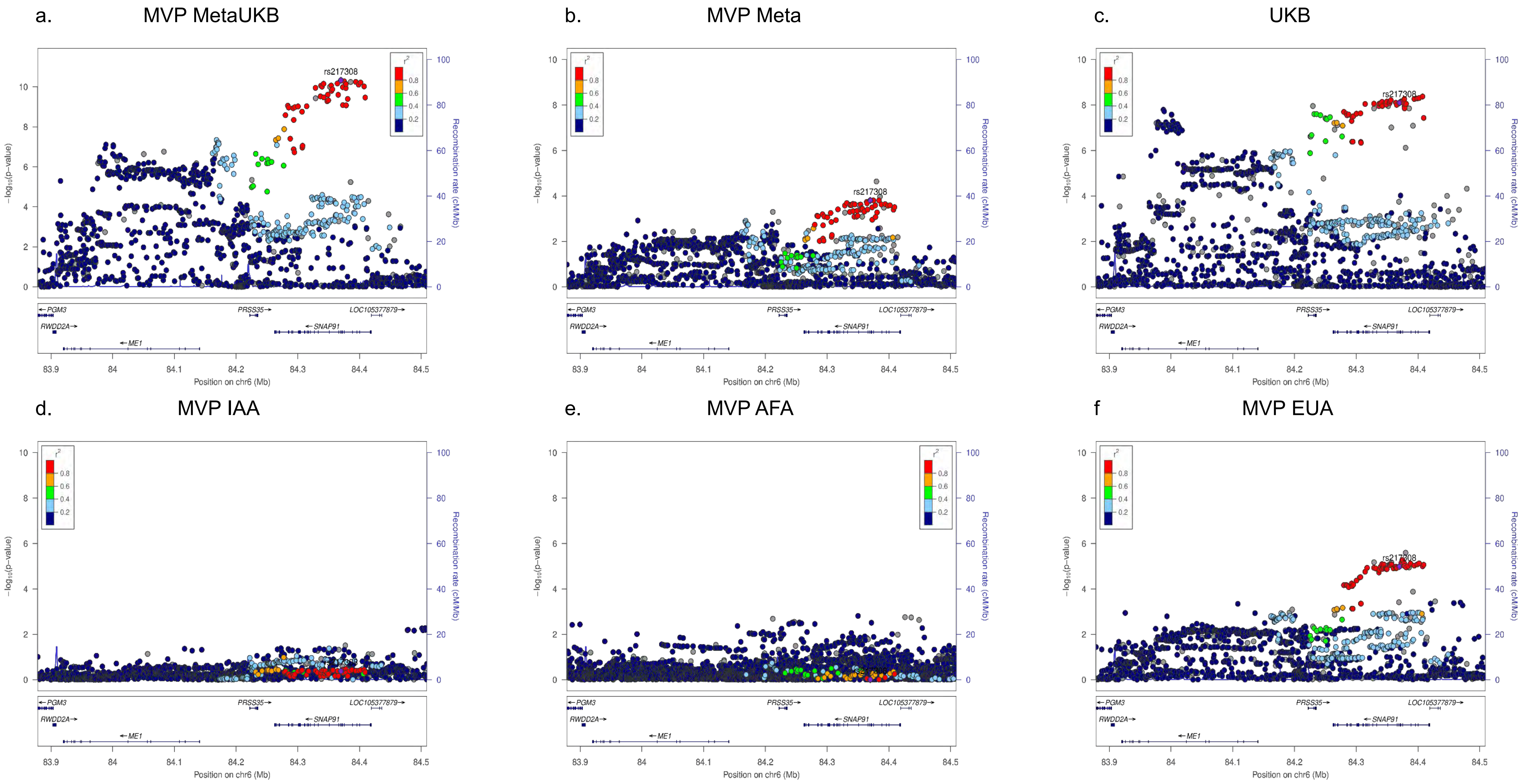

# locus051 | rs12213197

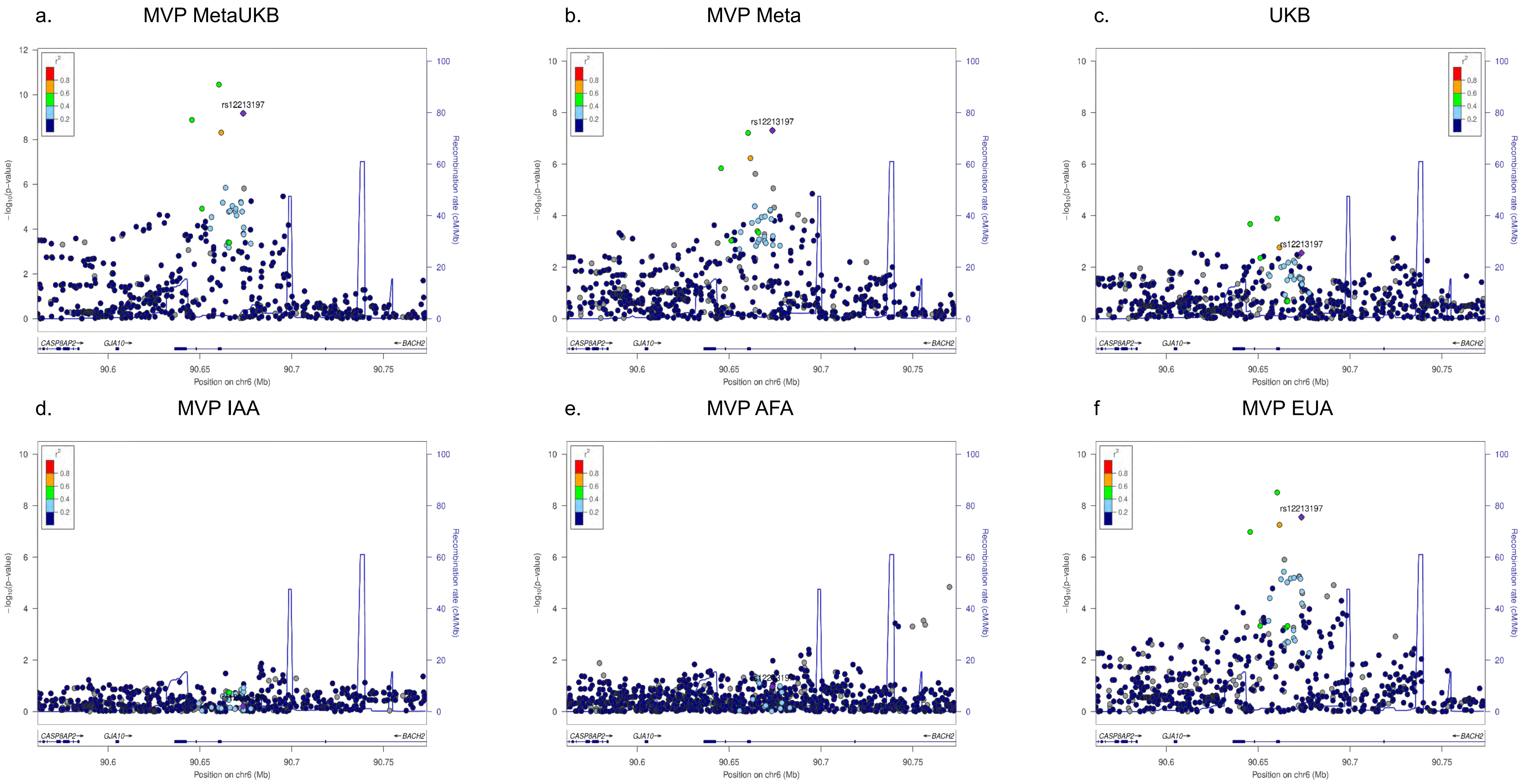

# locus051 | rs9451298

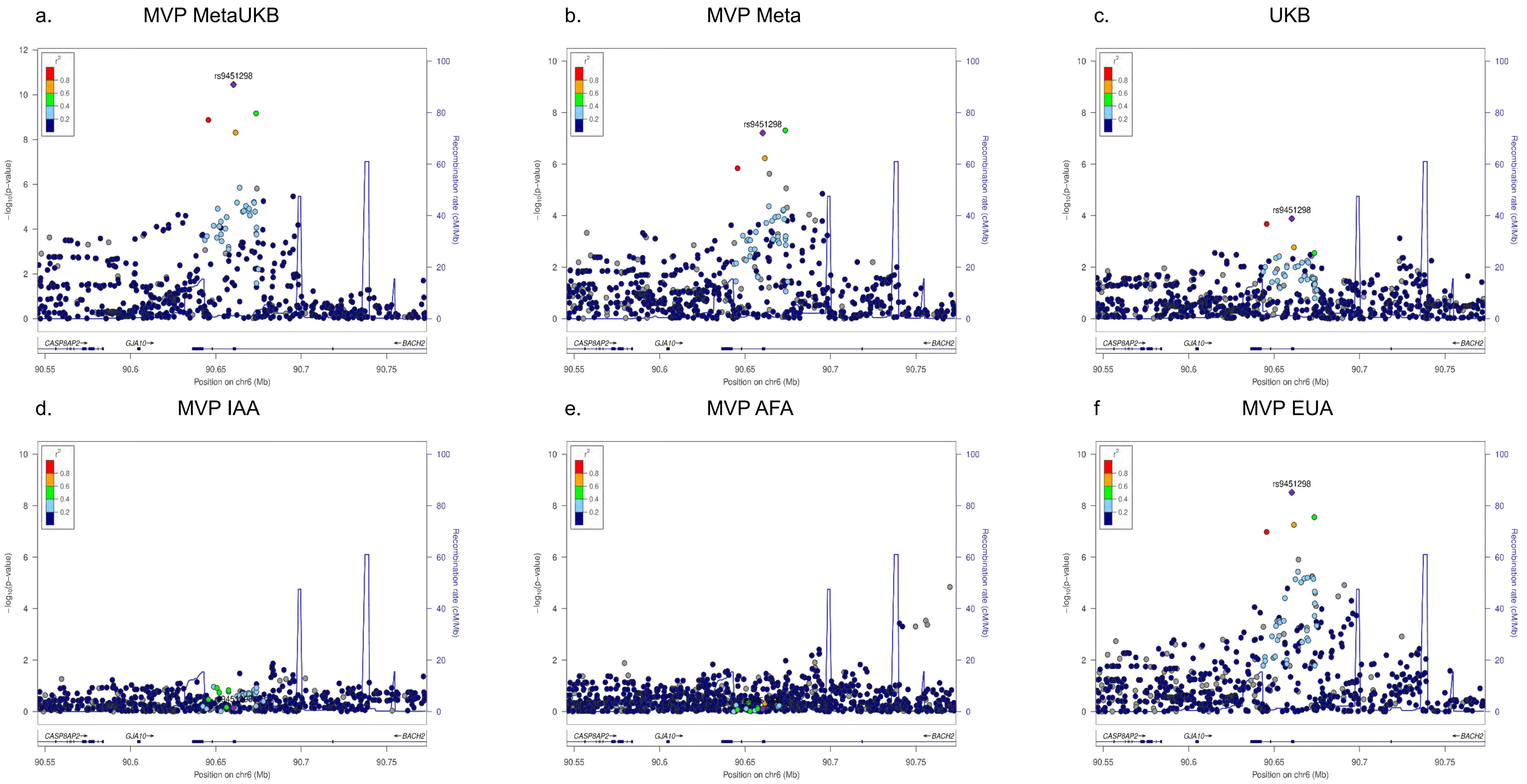

# locus052 | rs1022514

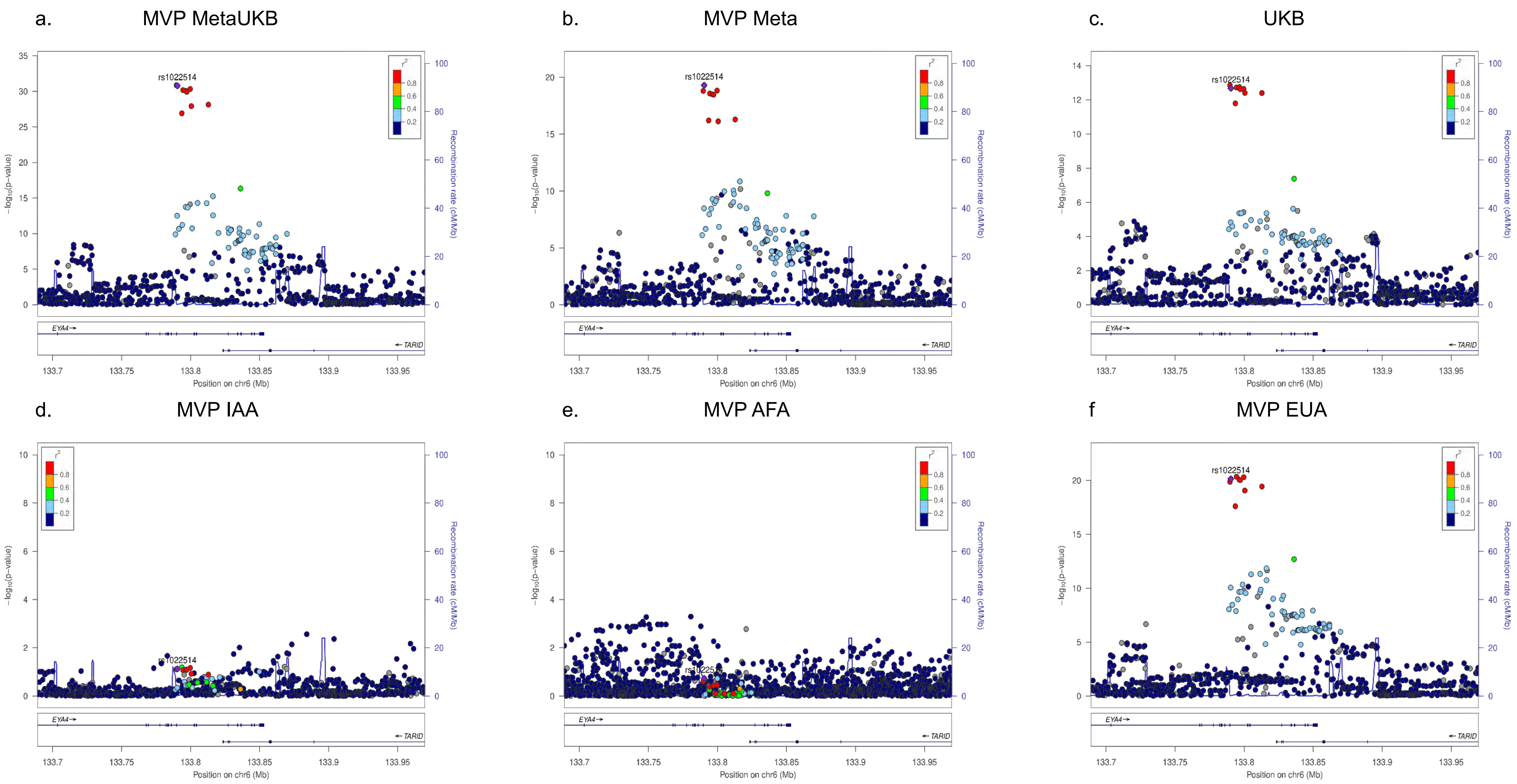

# locus052 | rs9321402

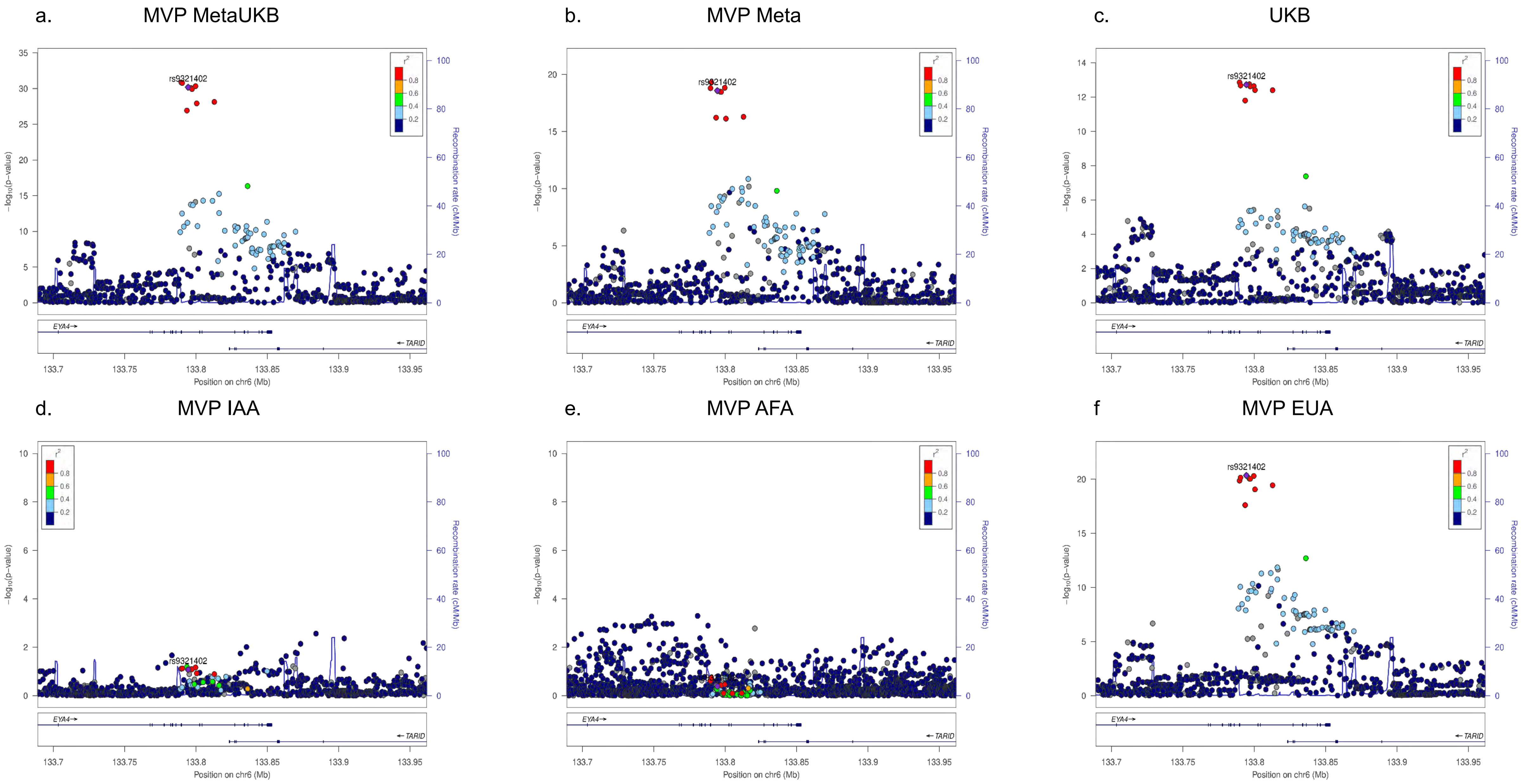

# locus052 | rs9493627

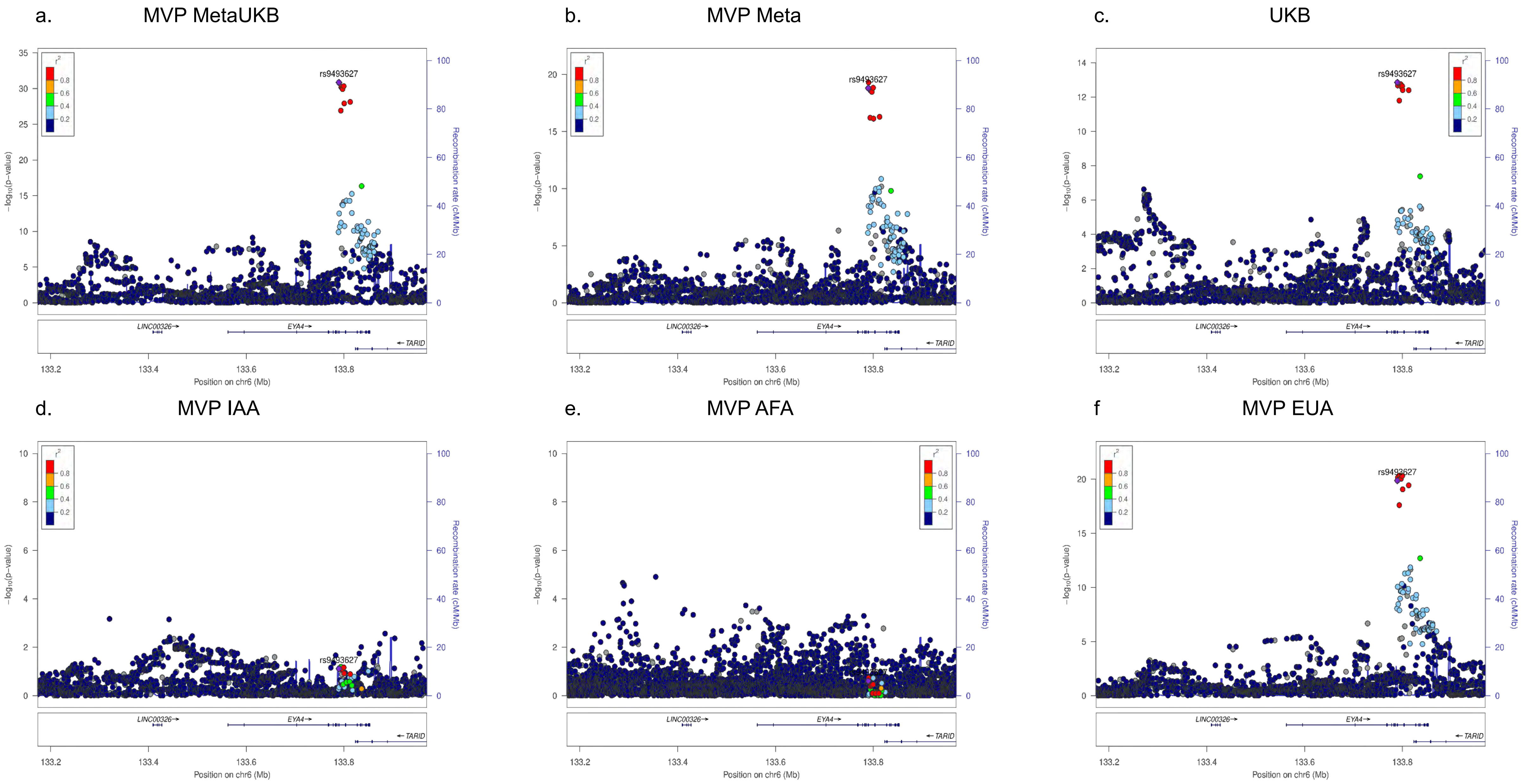

# locus053 | rs9390488

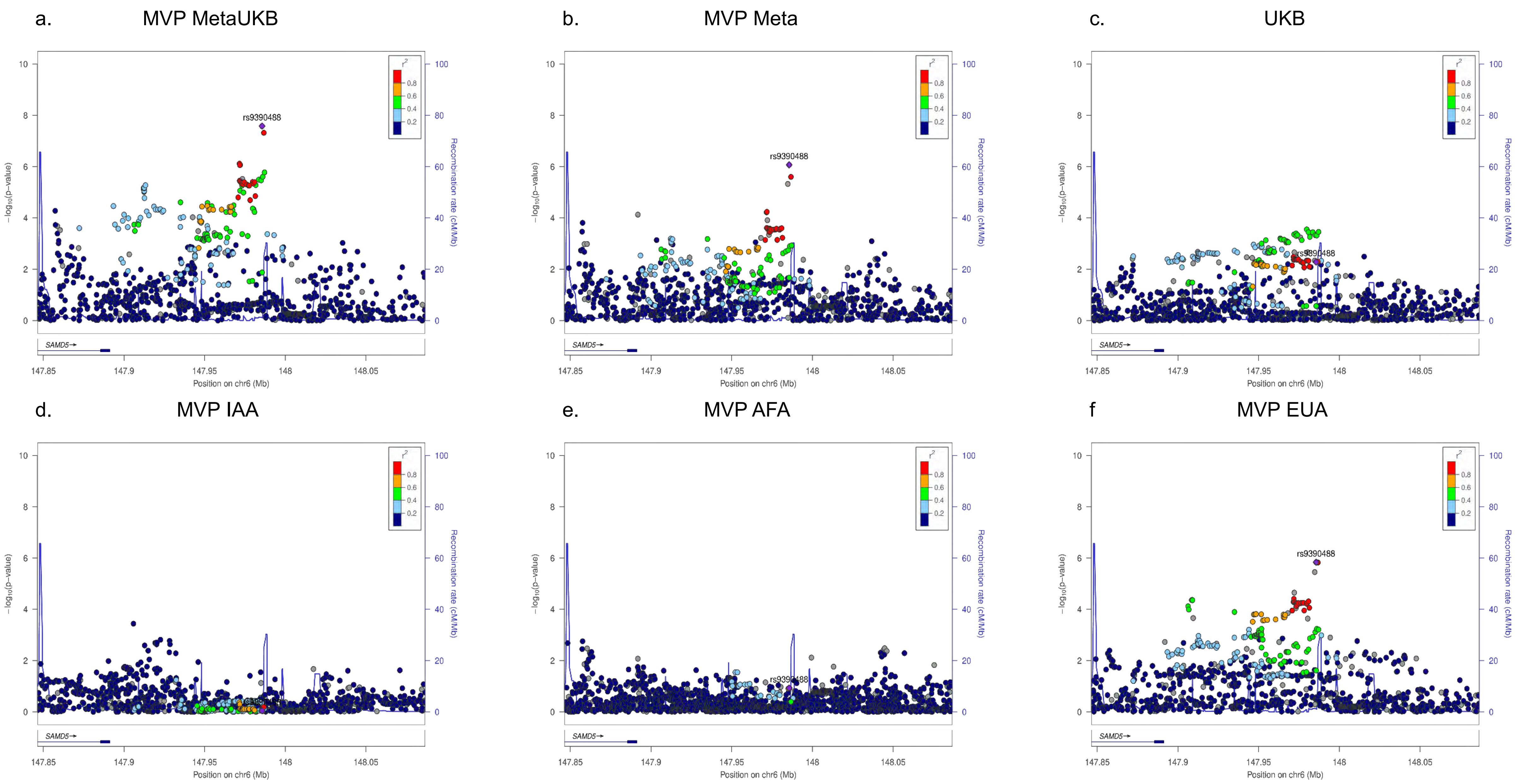

# locus054 | rs2236401

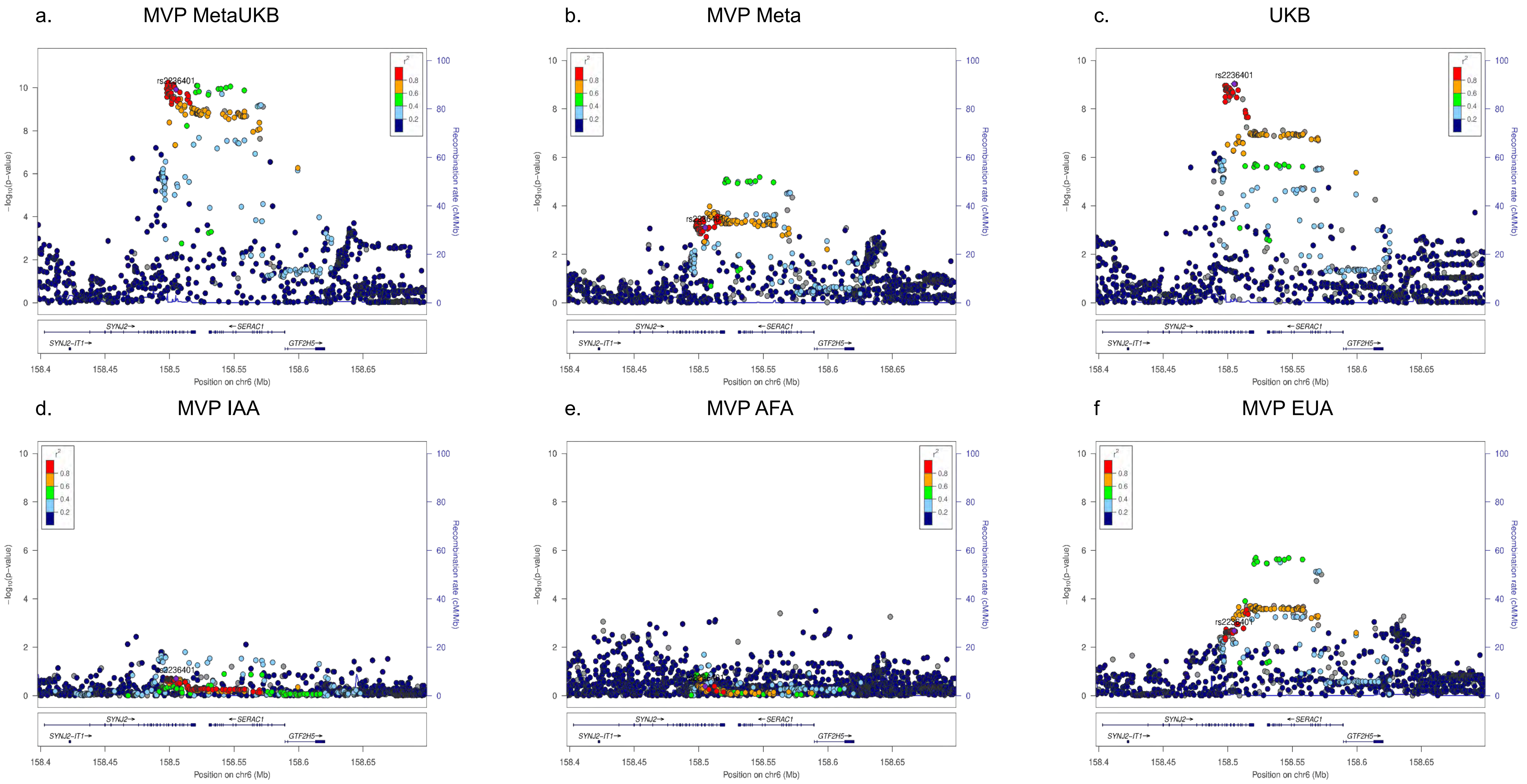

# locus054 | rs3818457

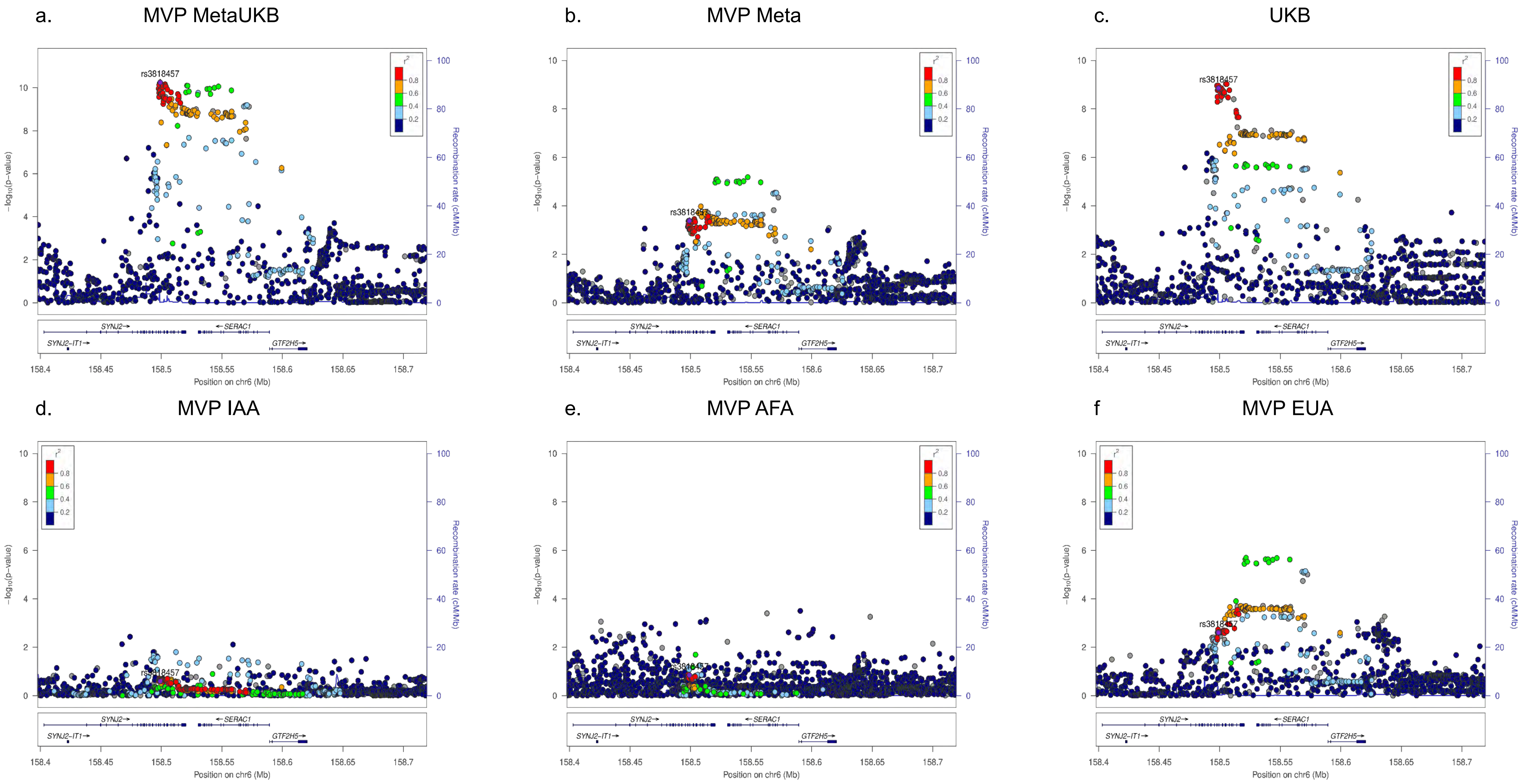

# locus055 | rs1737323

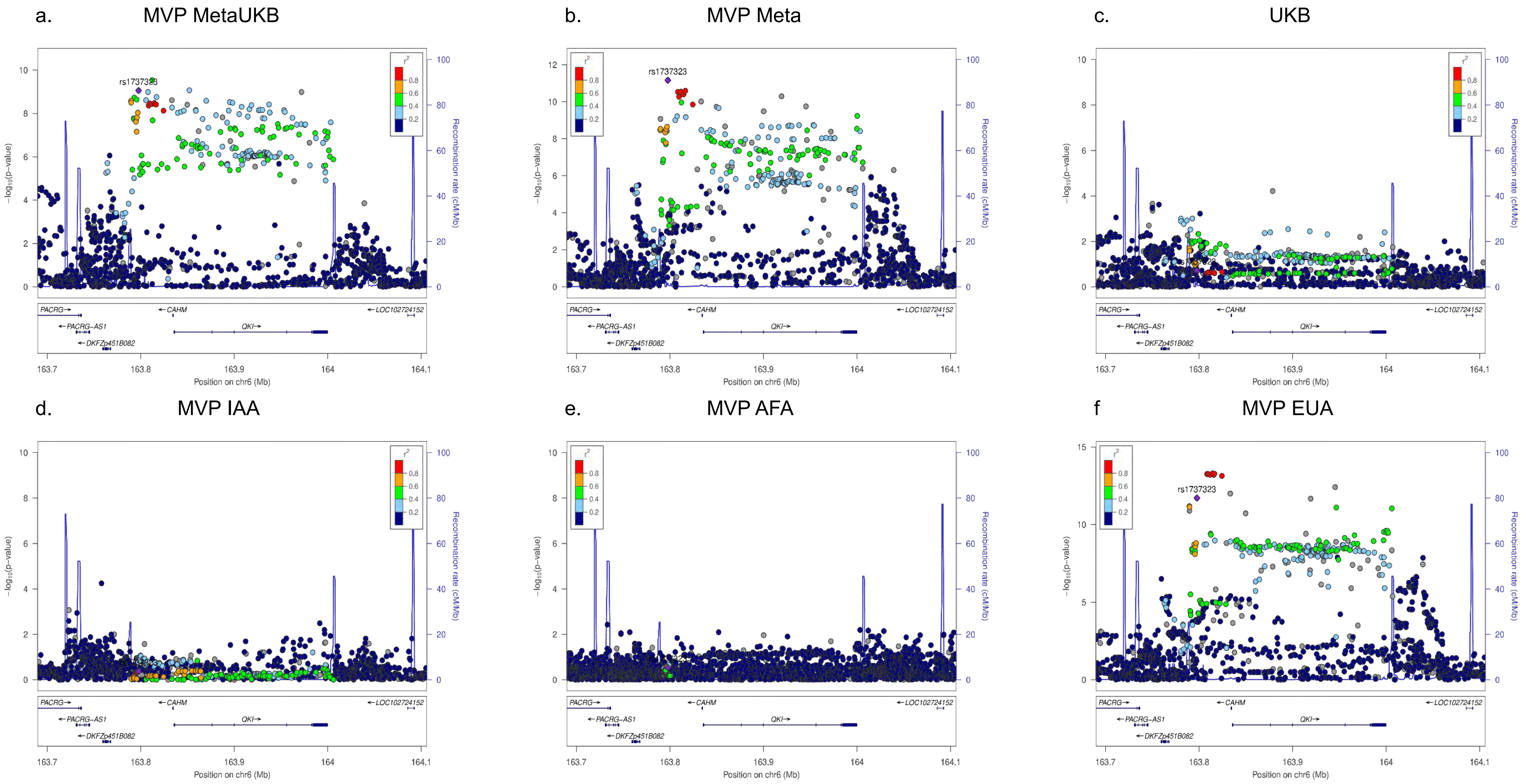

# locus055 | rs1737332

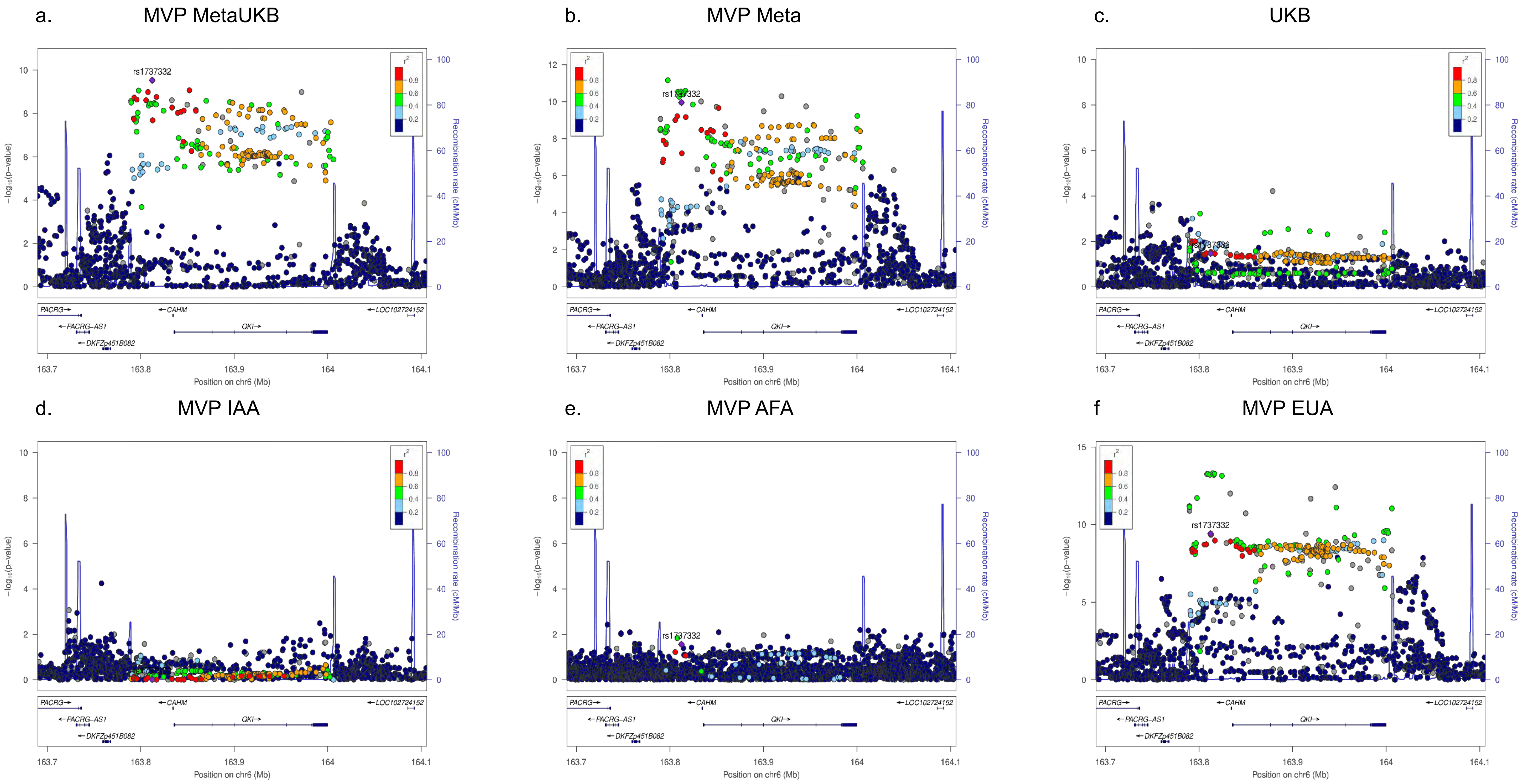

# locus055 | rs4709715

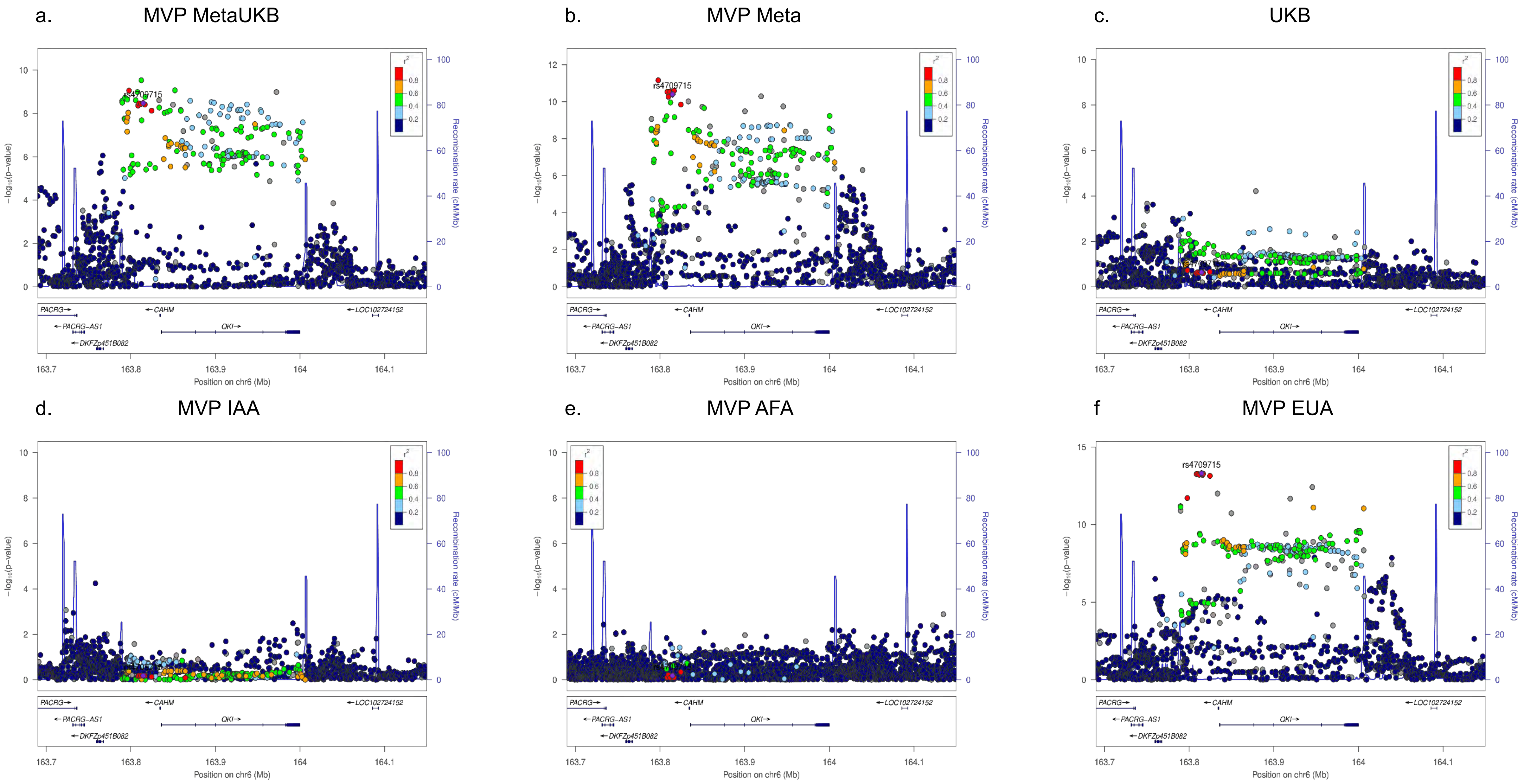

# locus056 | rs10232942

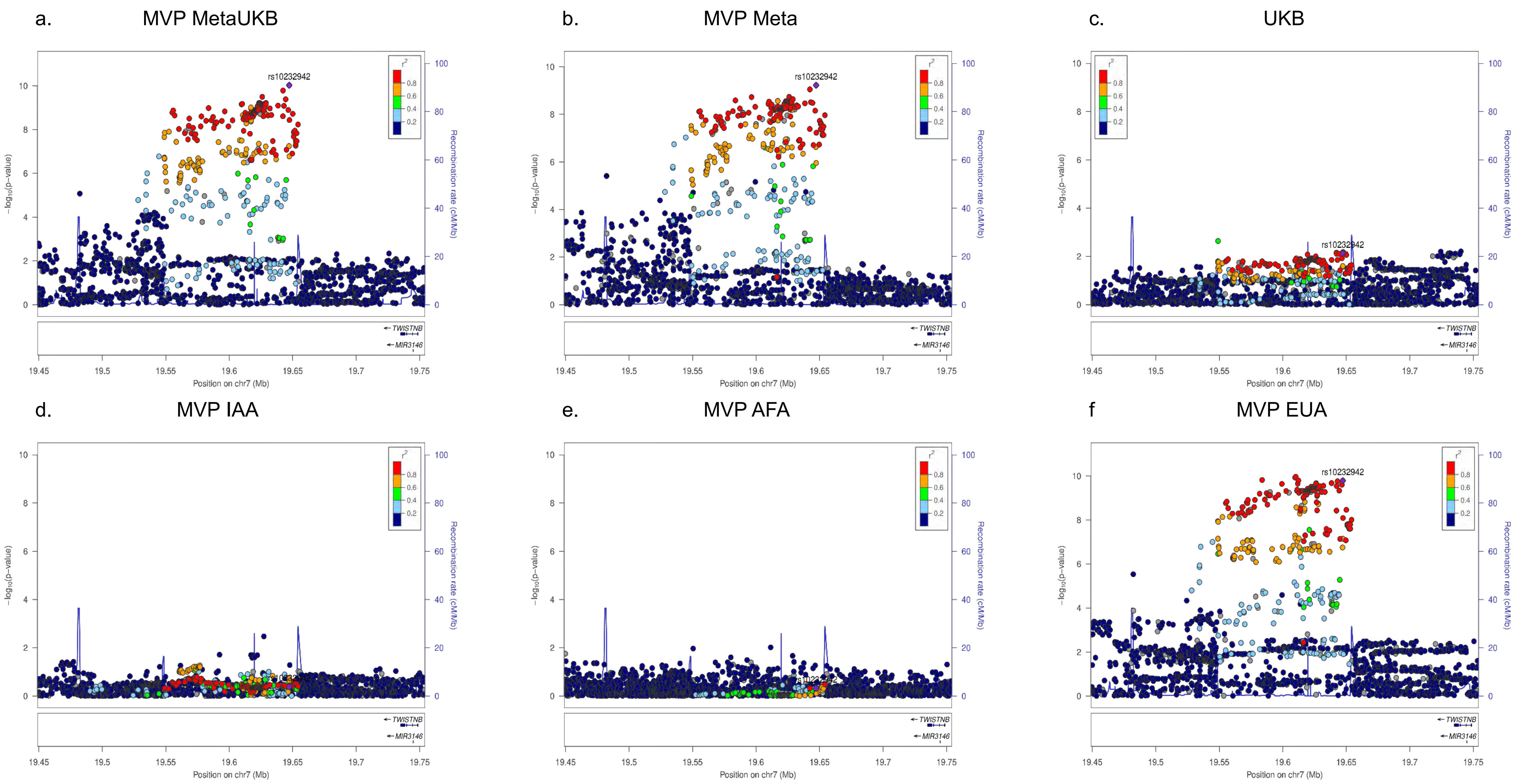

# locus056 | rs10241336

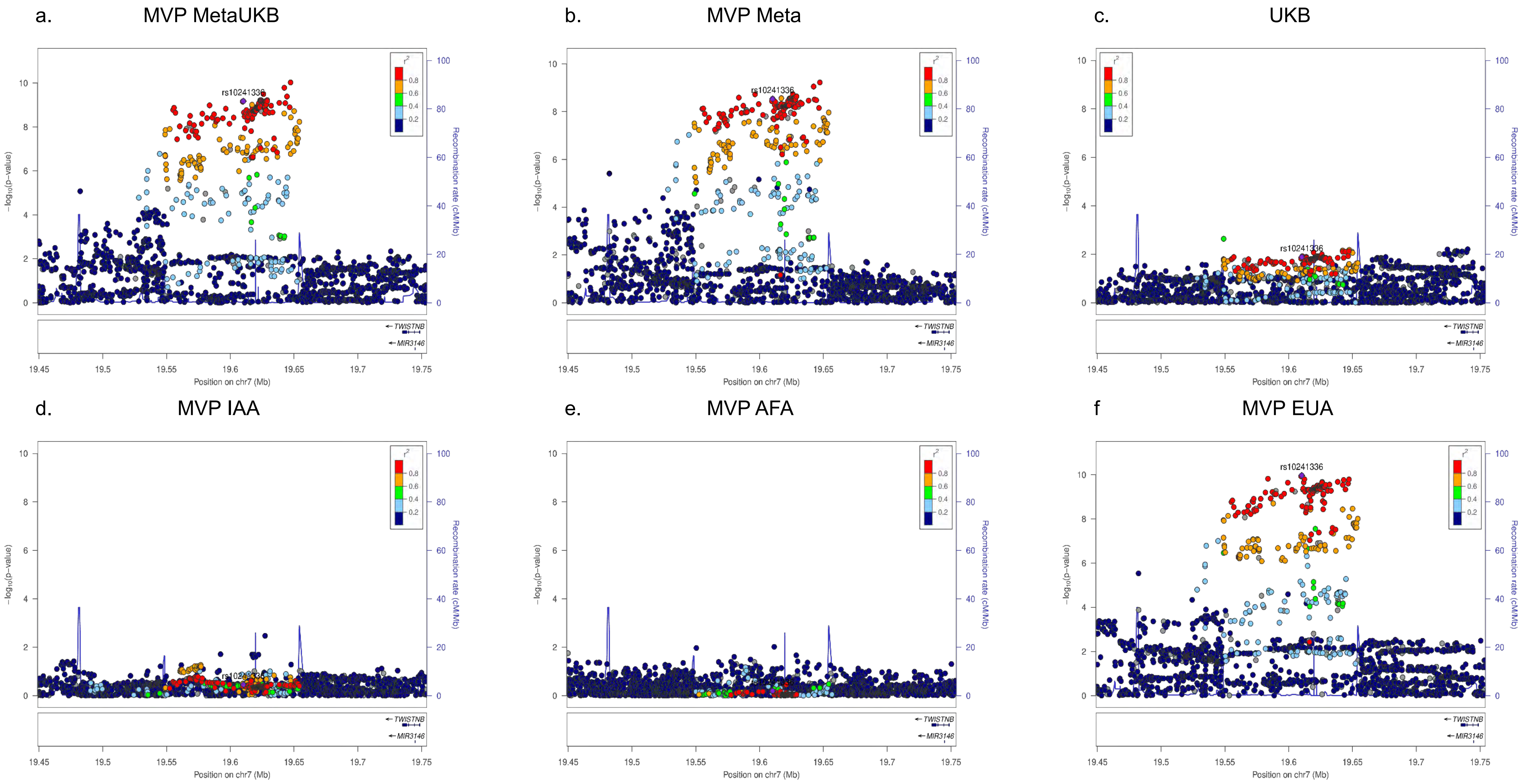

# locus057 | rs1962390

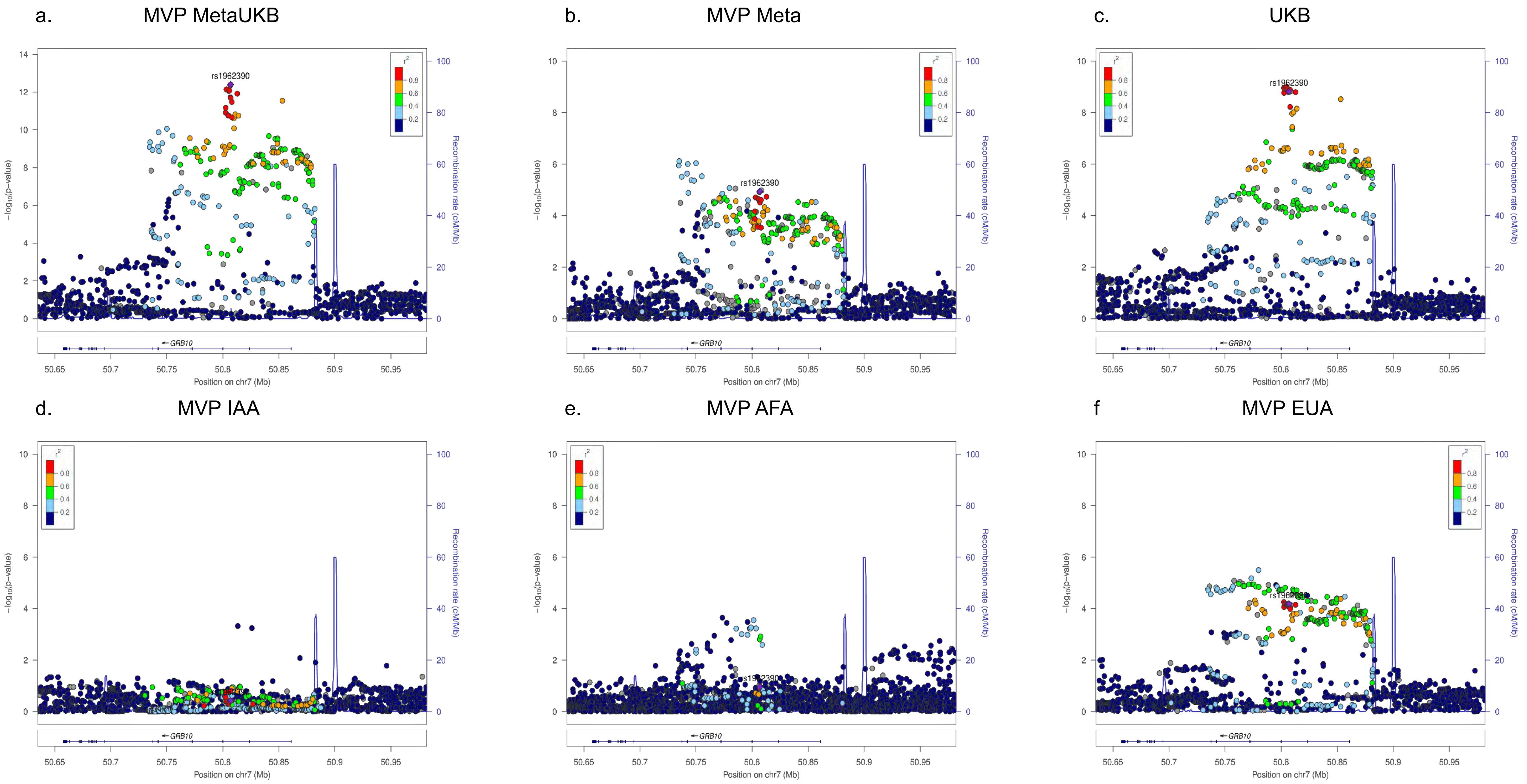

# locus057 | rs4947828

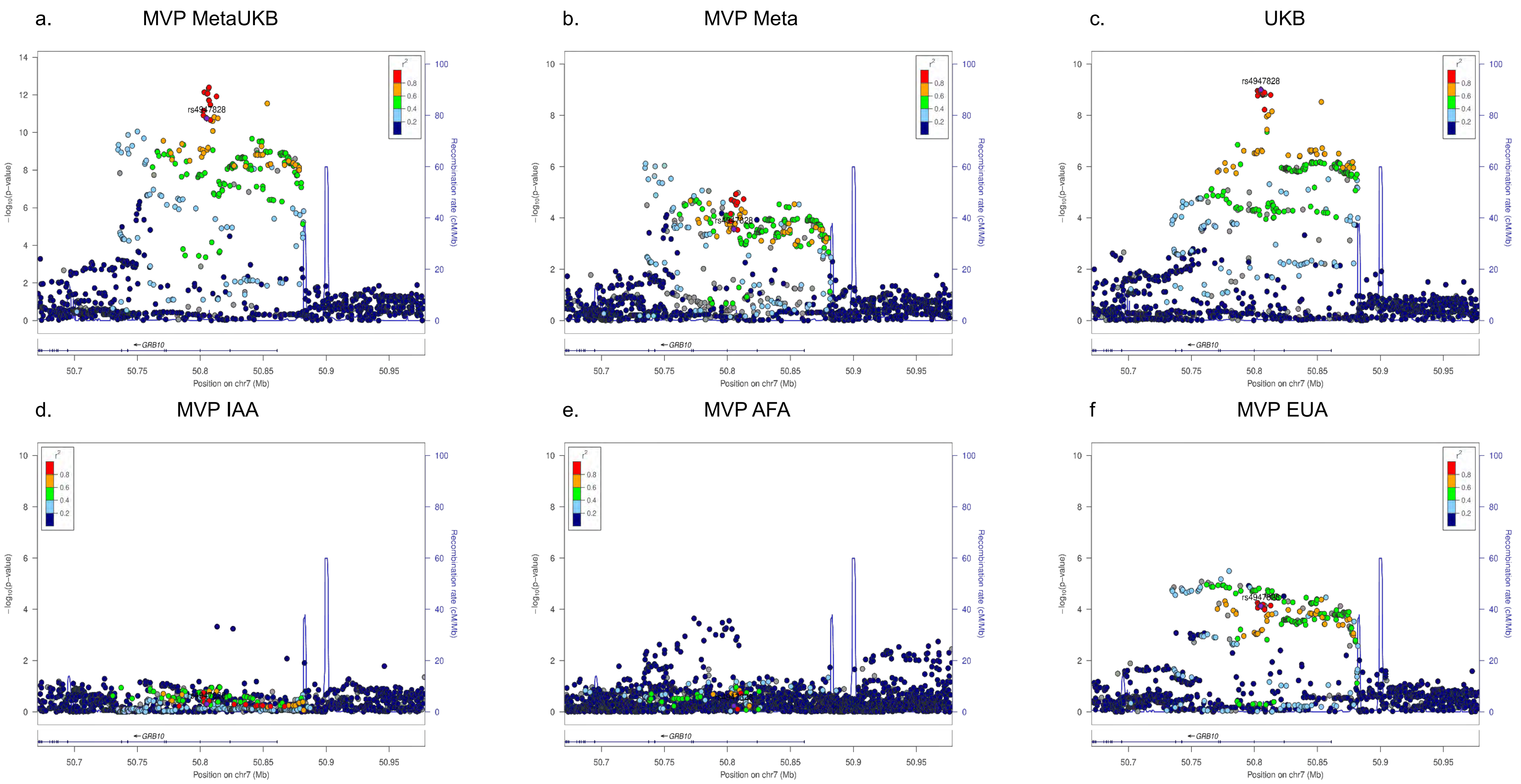

# locus058 | rs274625

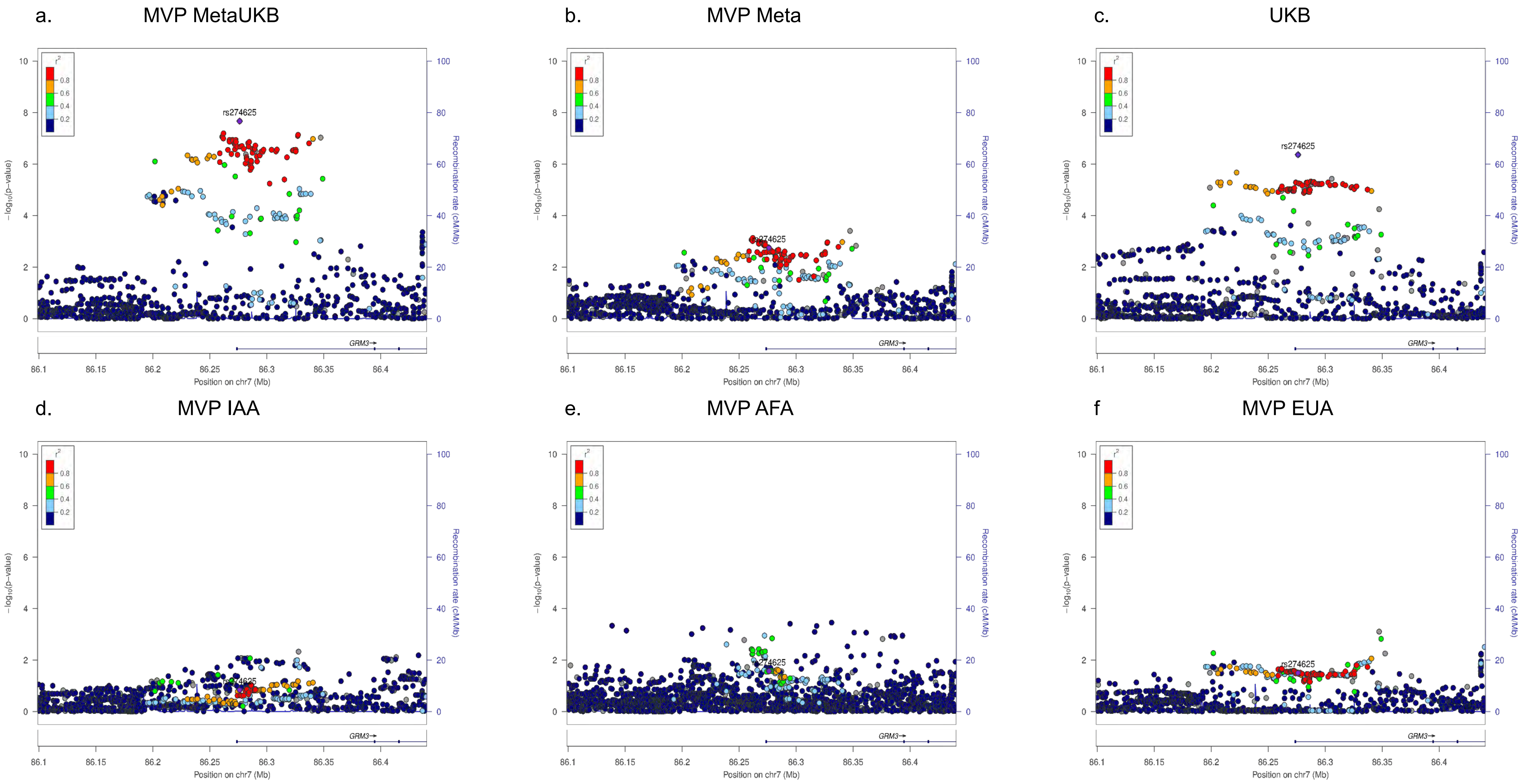

# locus059 | rs7780560

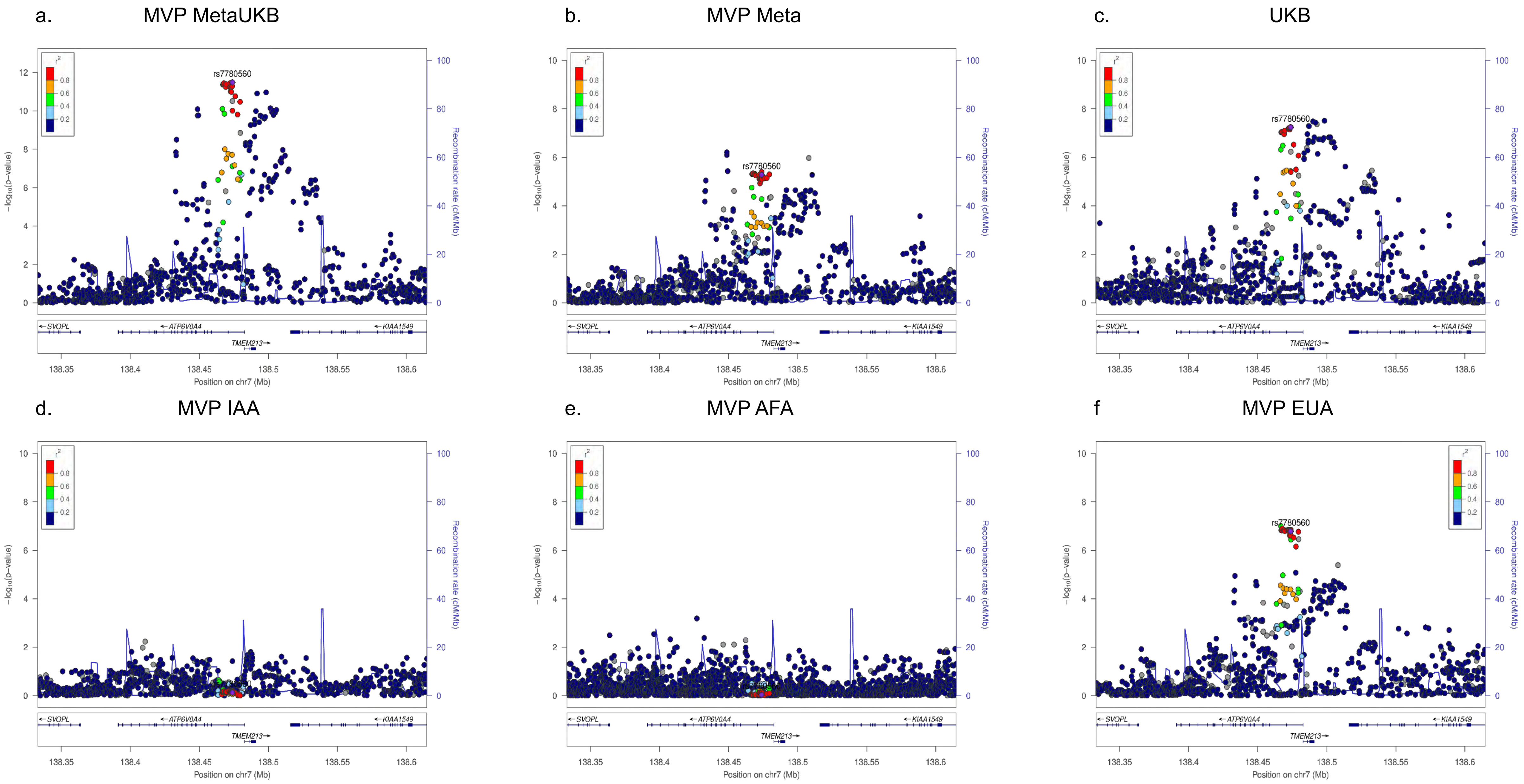

# locus059 | rs9691831

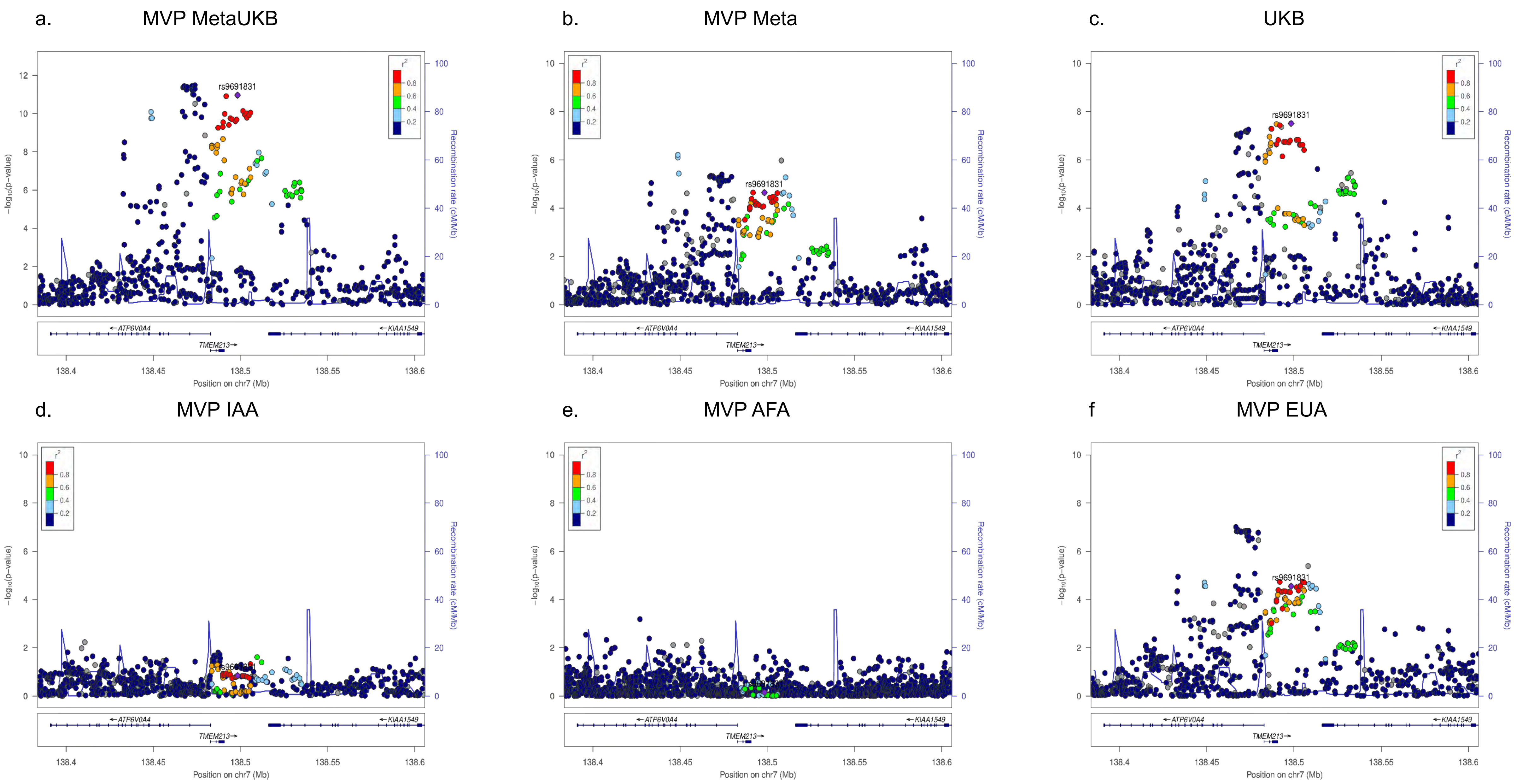

# locus060 | rs3890736

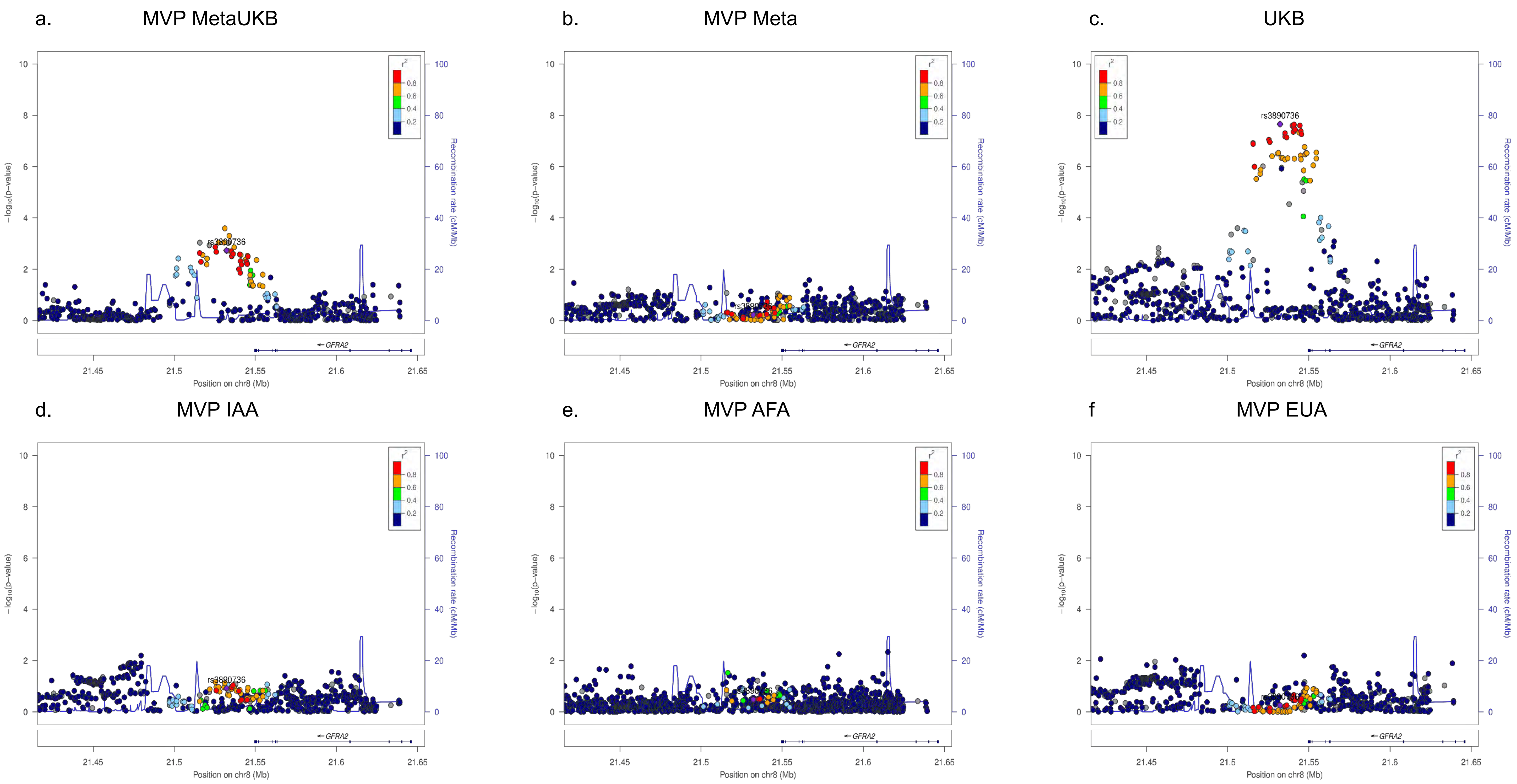

# locus061 | rs113973451

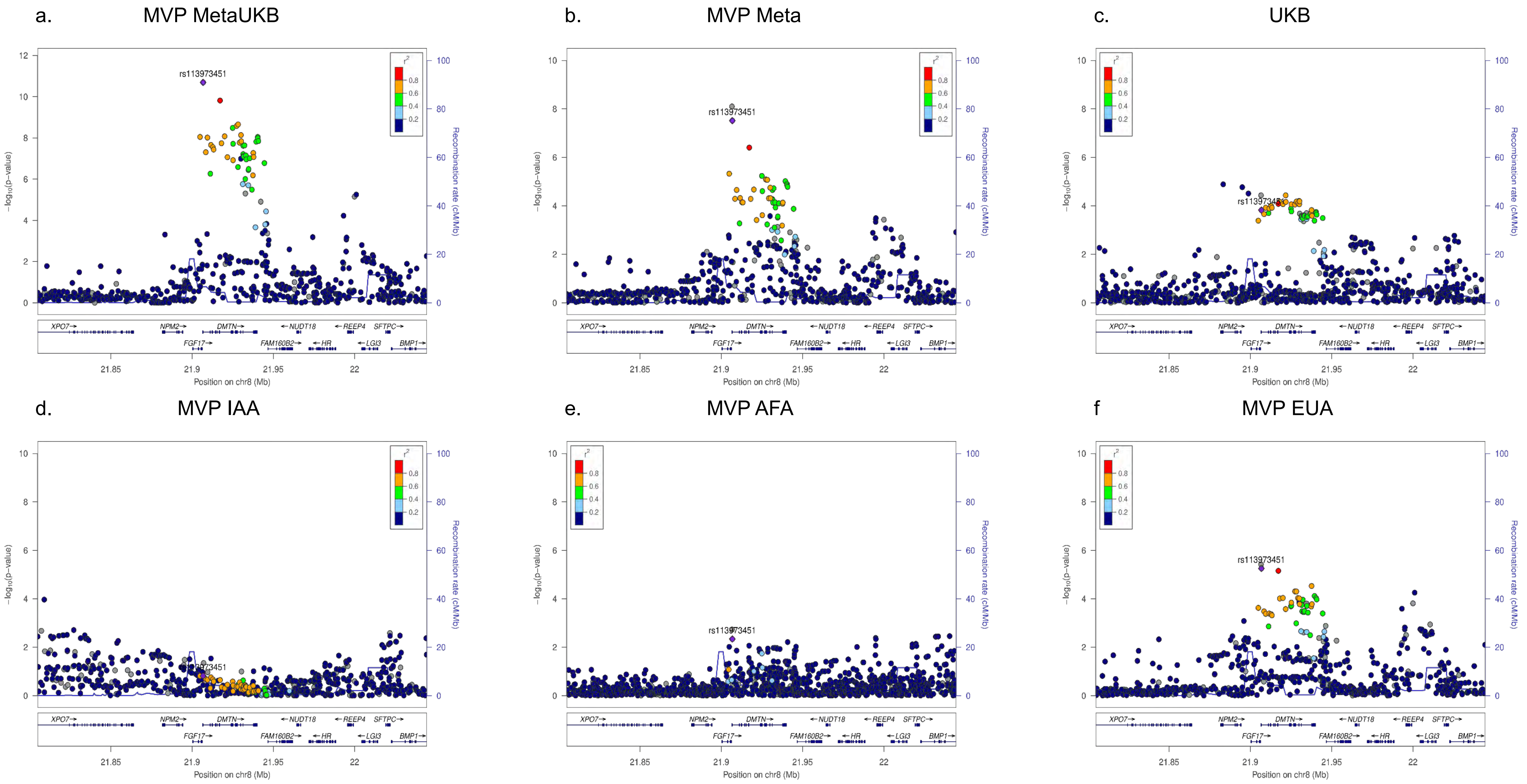

# locus061 | rs200363538

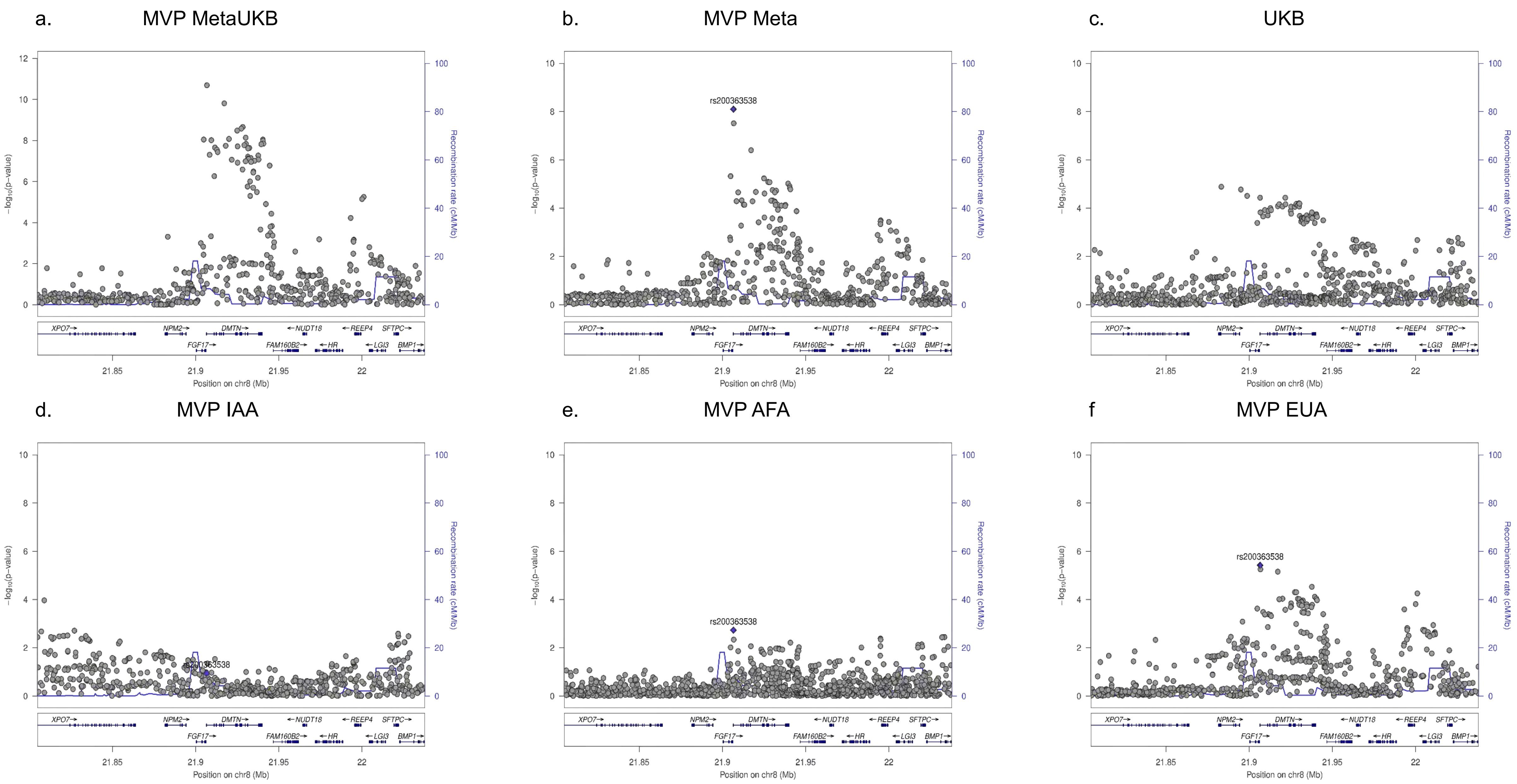

# locus062 | rs17627903

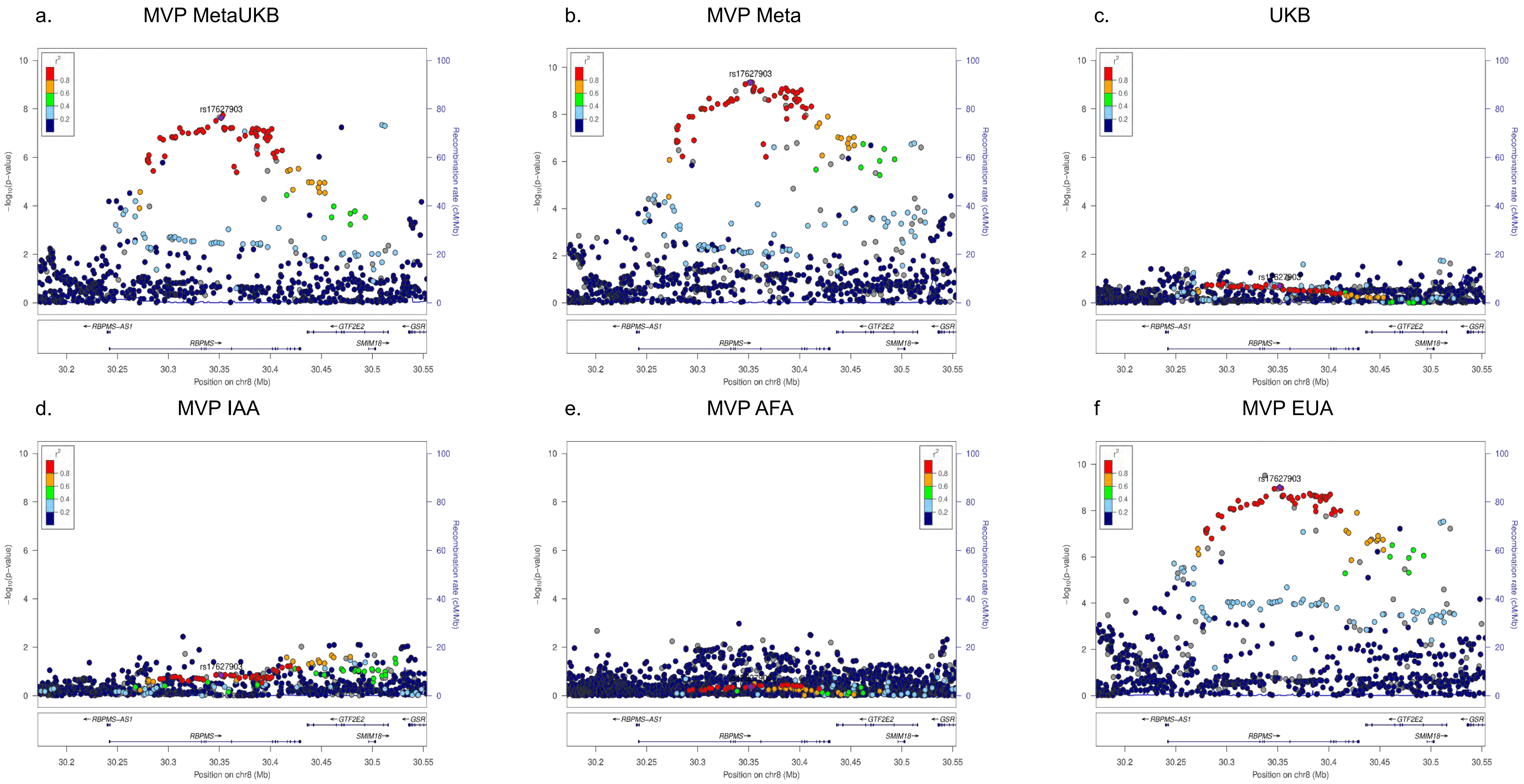

# locus062 | rs3824107

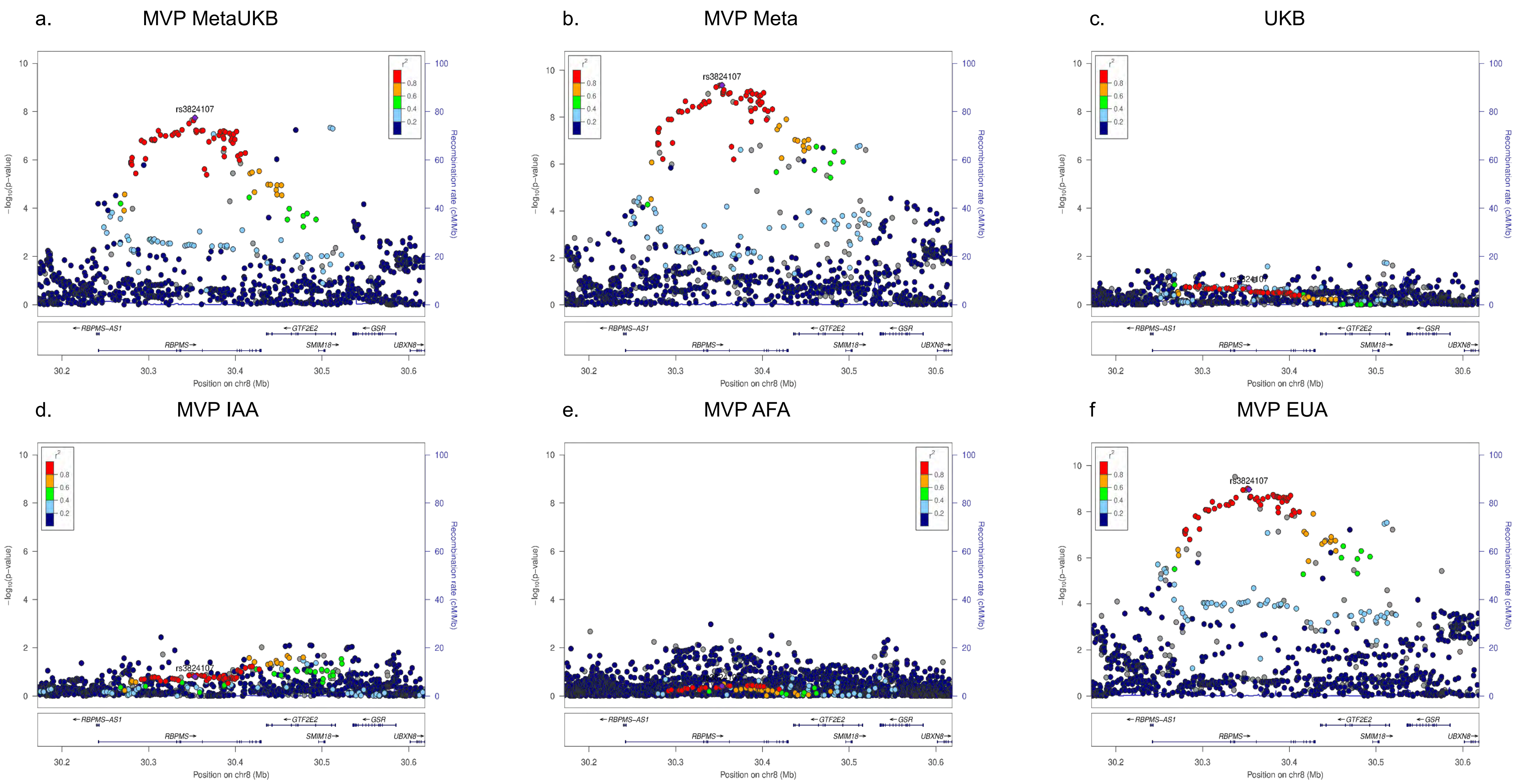

# locus062 | rs5890523

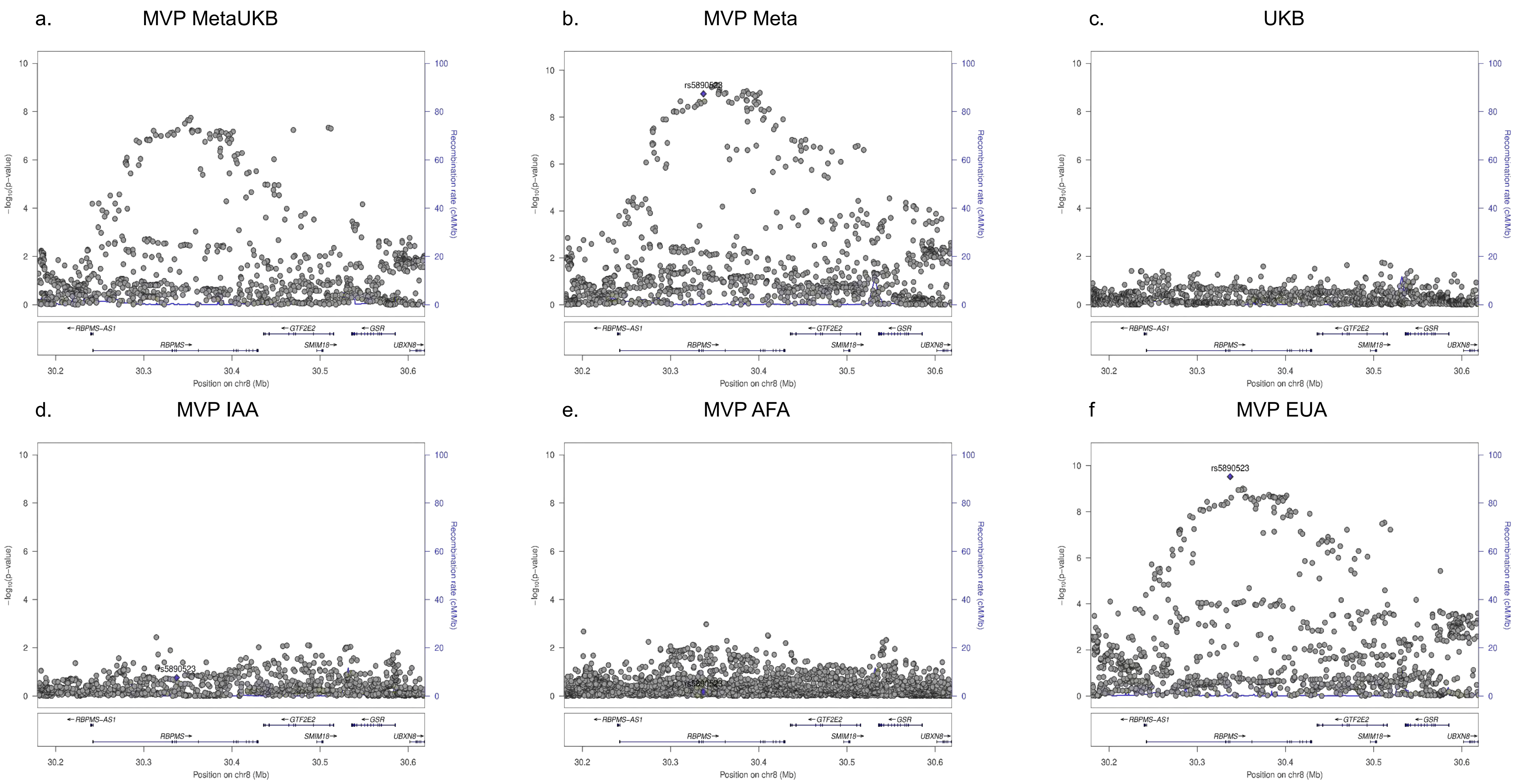

# locus063 | rs17760069

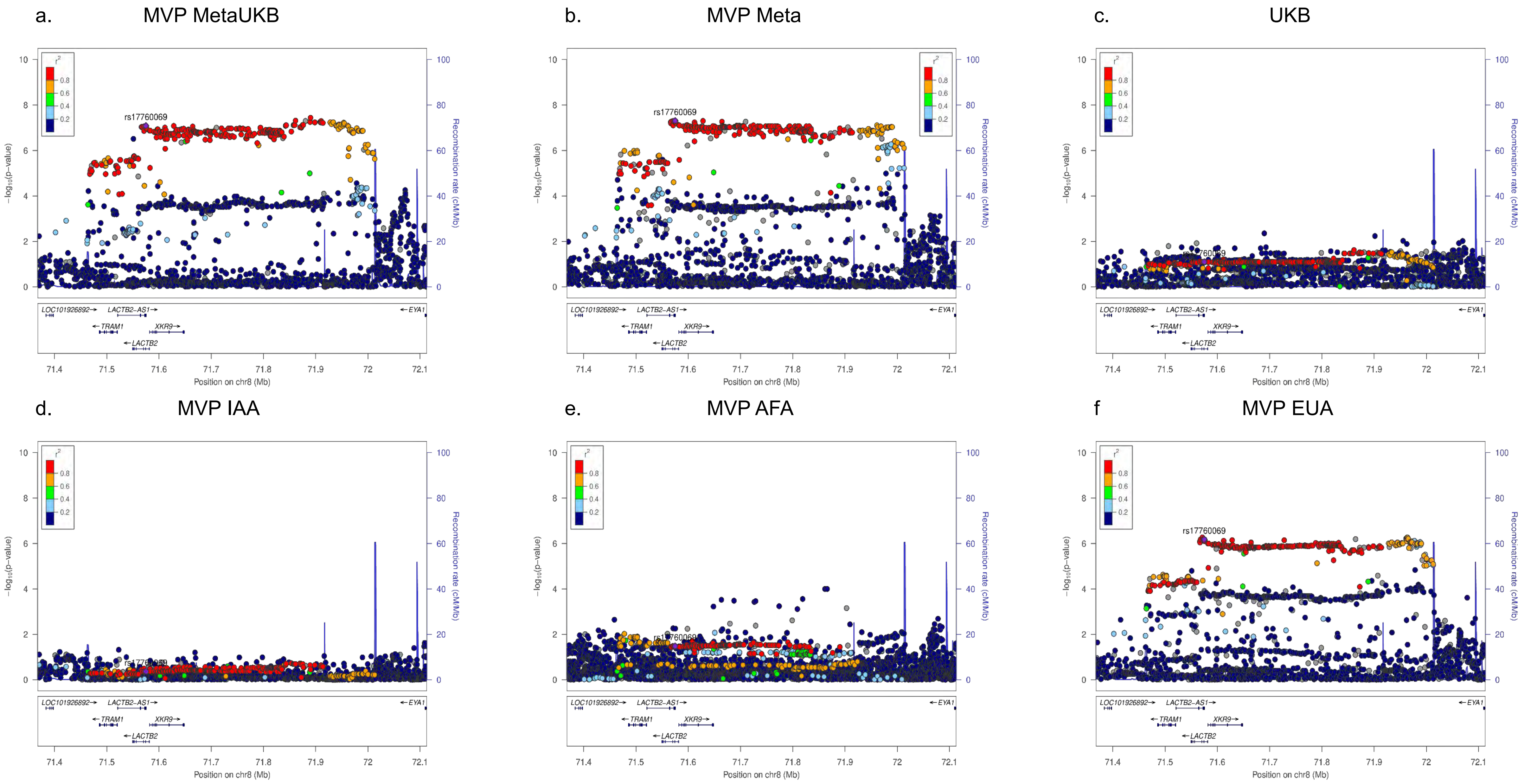

# locus063 | rs62506938

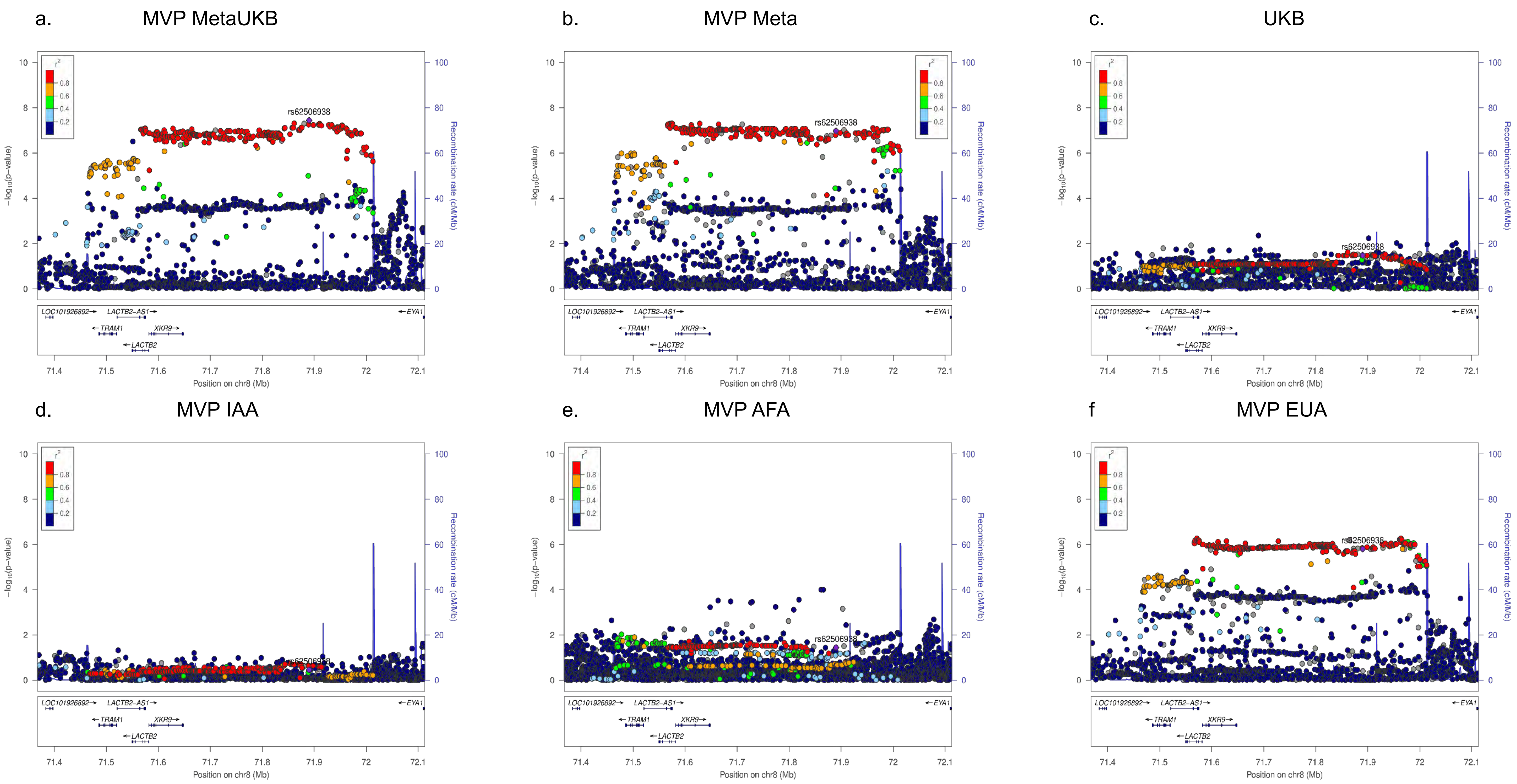

locus064 | rs7819550

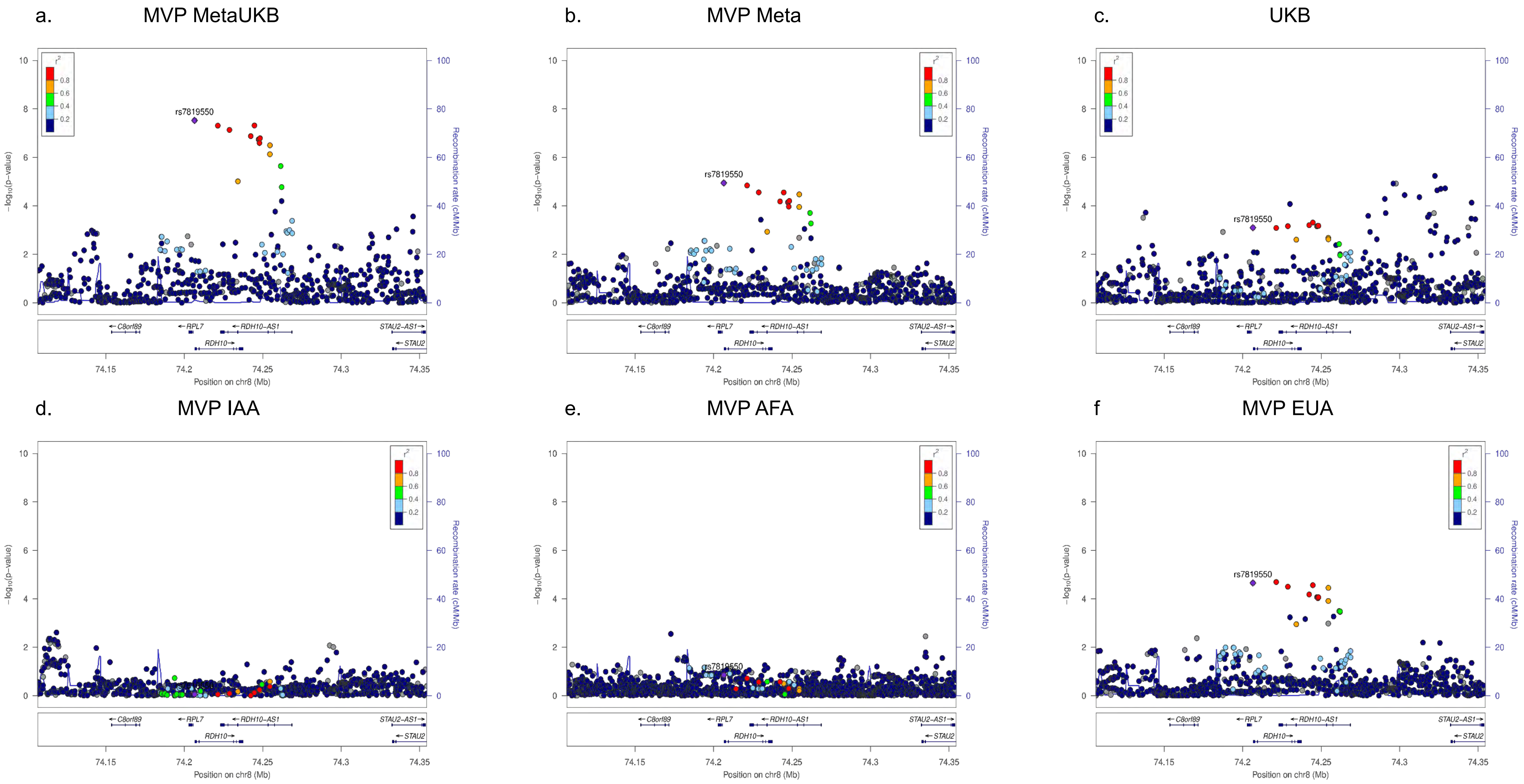

# locus065 | rs76837345

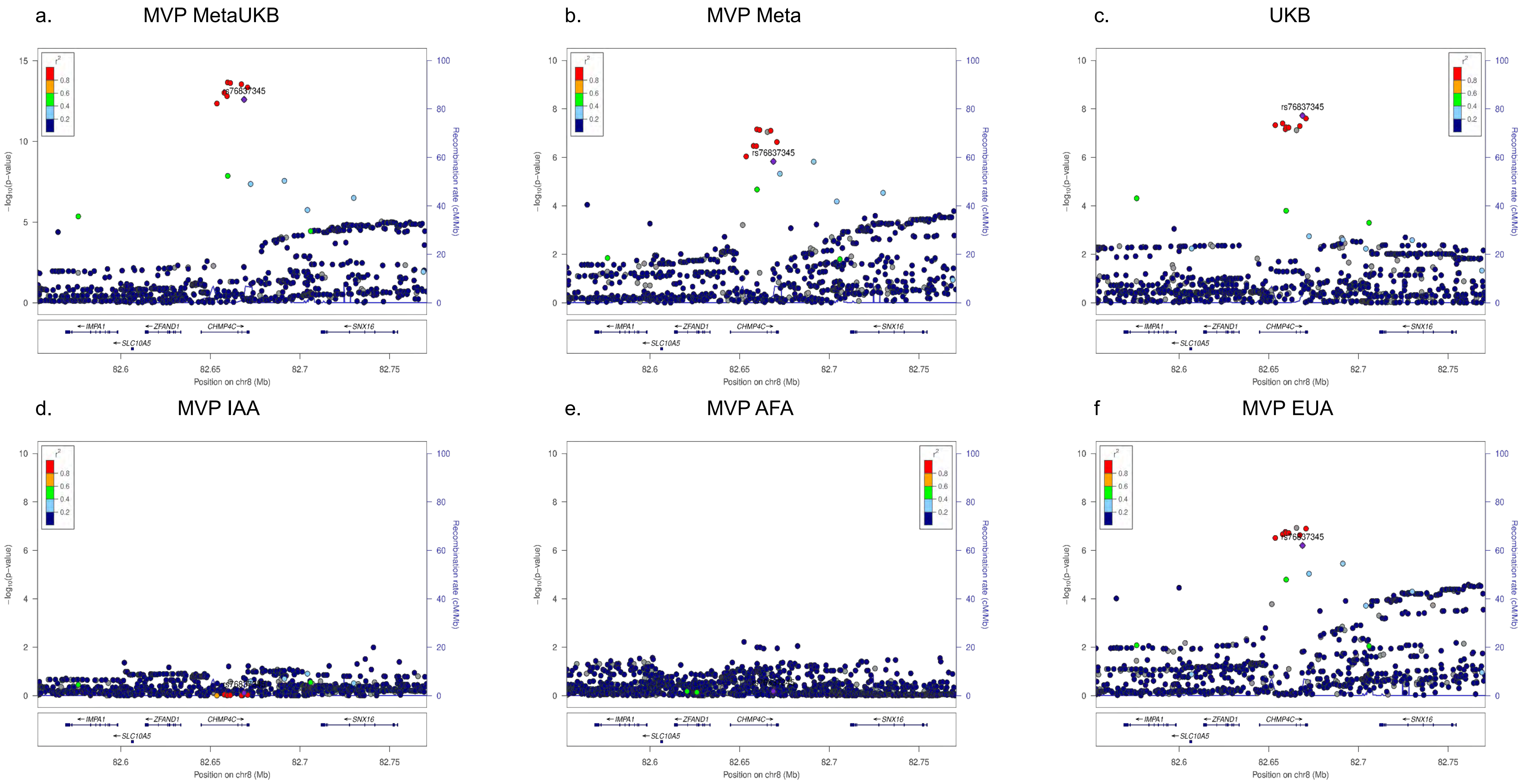

# locus065 | rs78724141

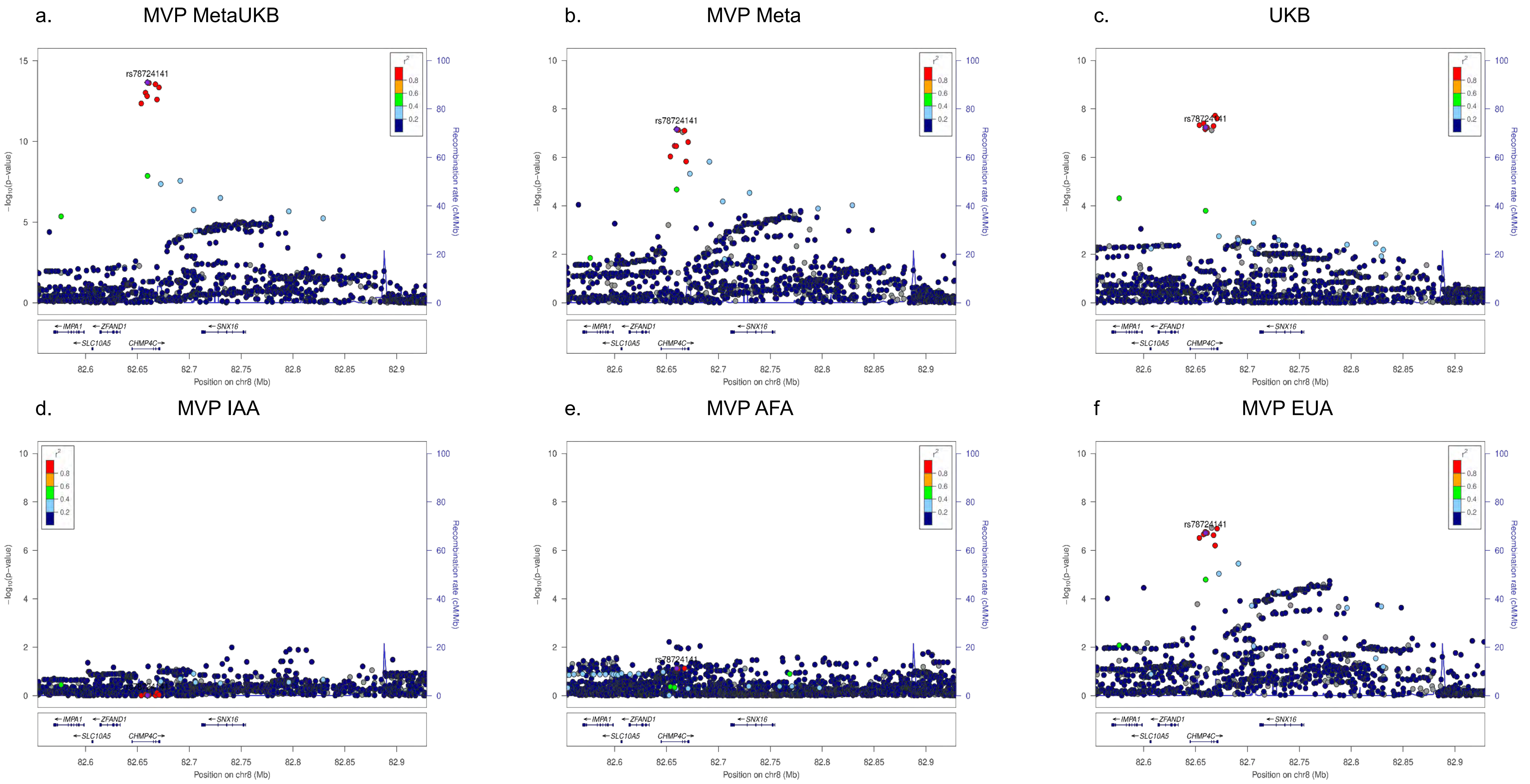

# locus066 | rs11784152

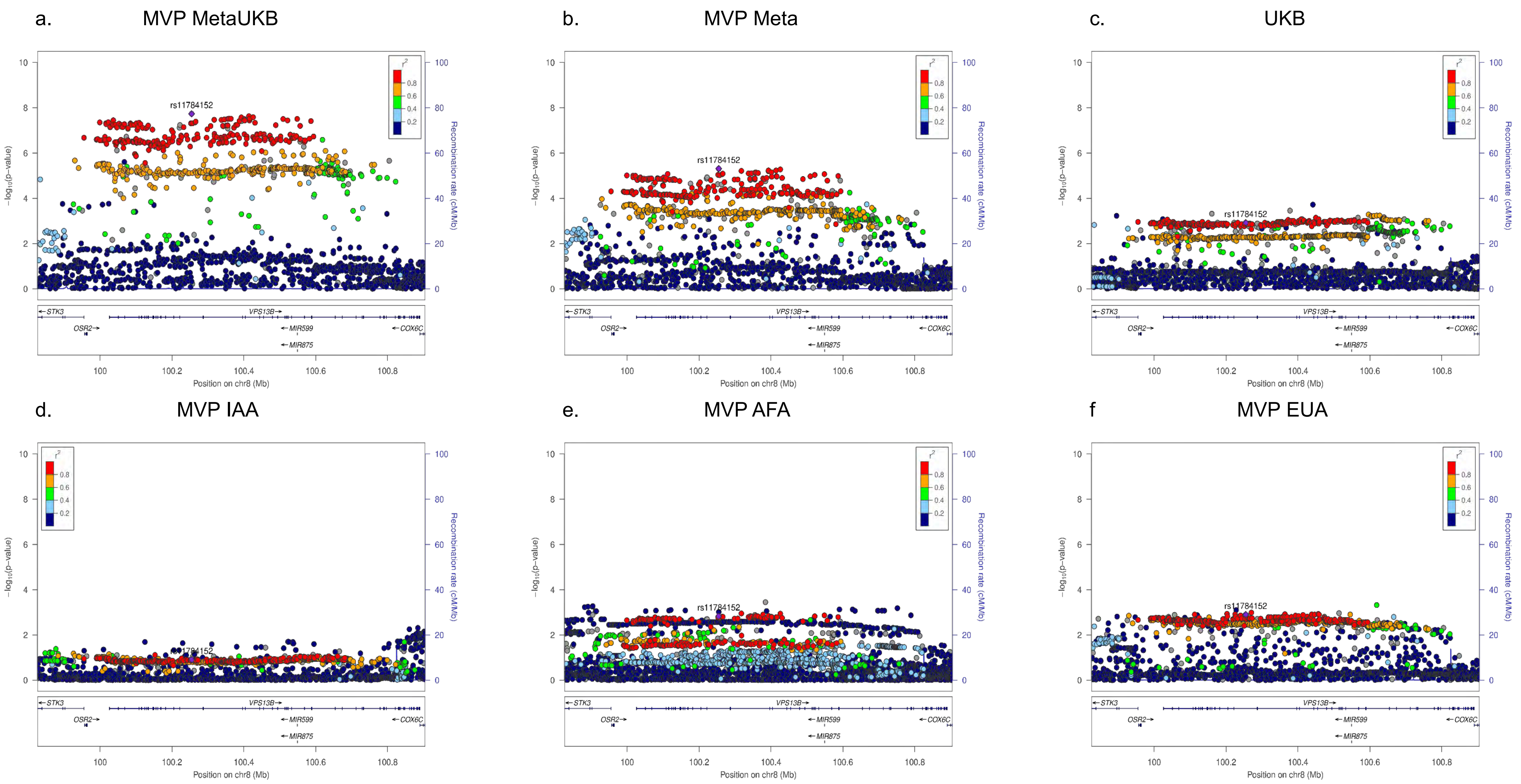

# locus067 | rs12156228

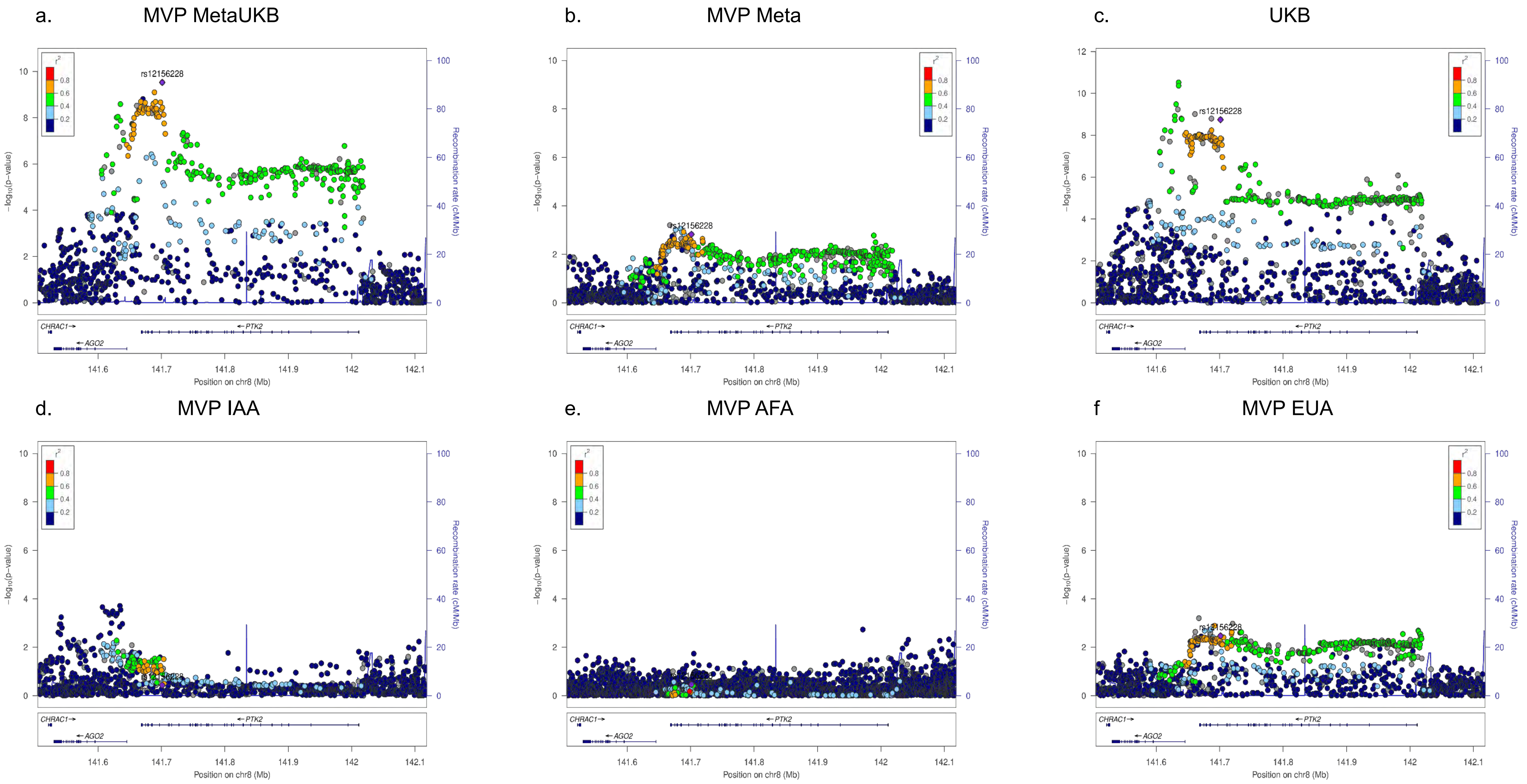

# locus067 | rs1962104

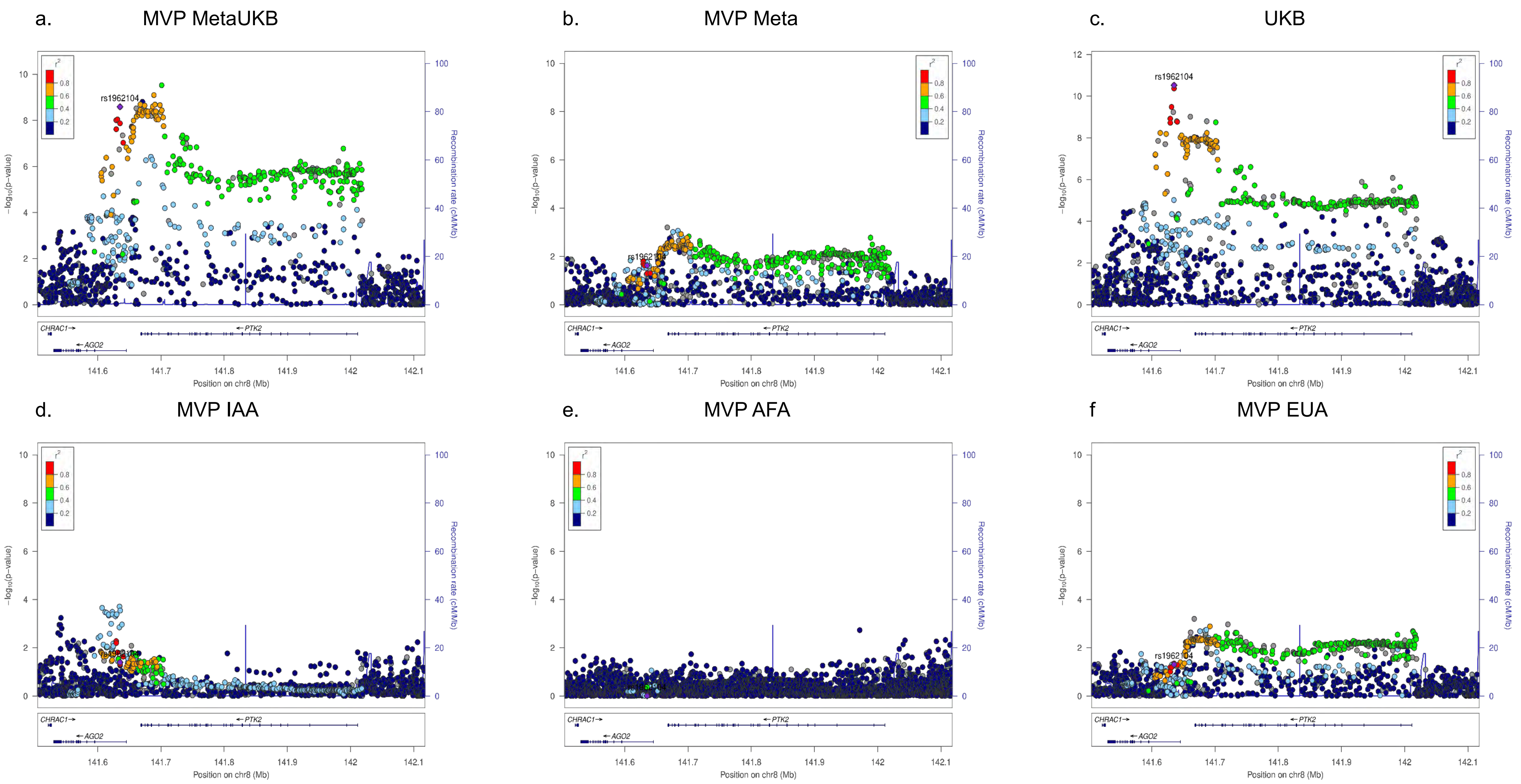

locus068 |  rs1409880

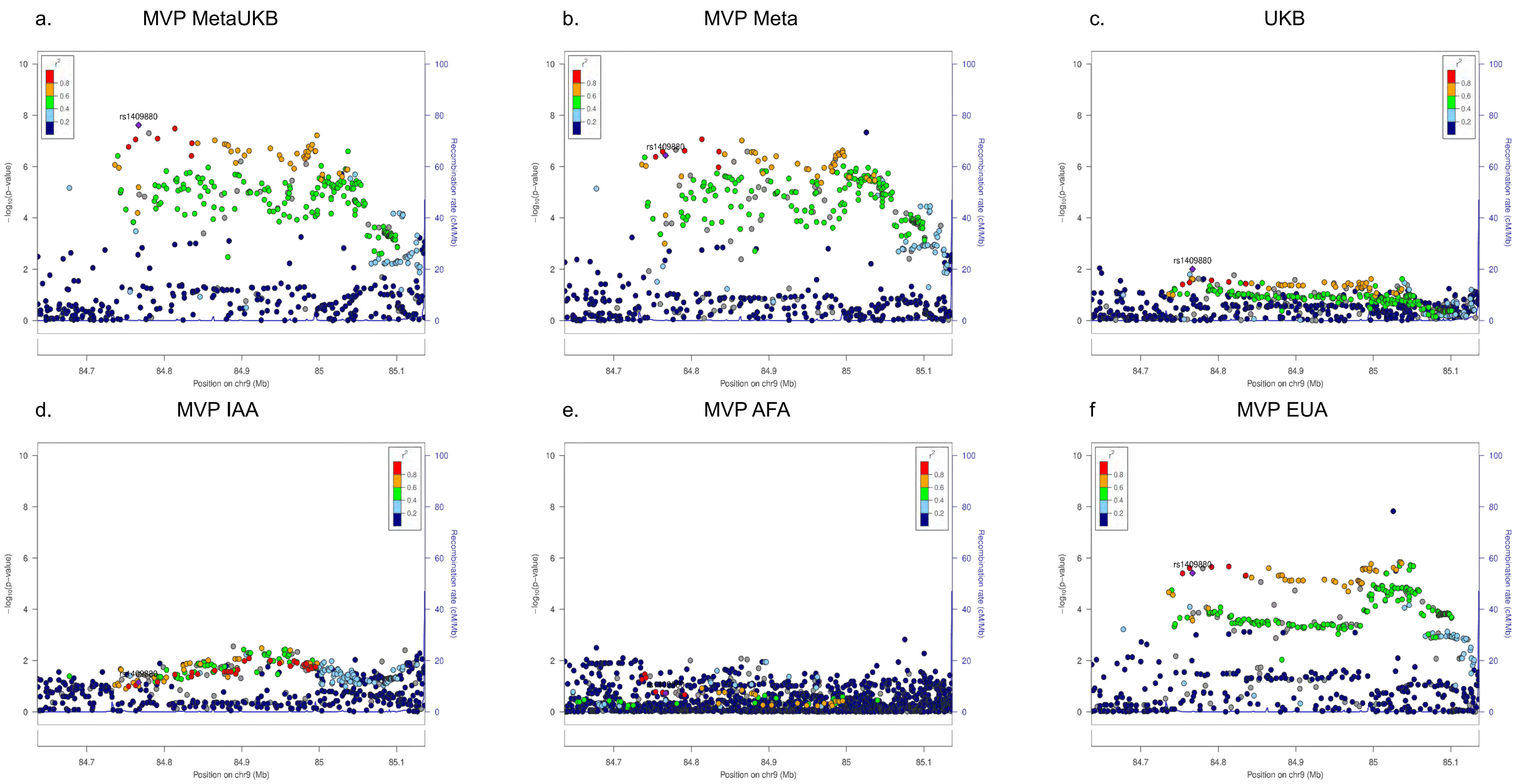

# locus068 | rs72743537

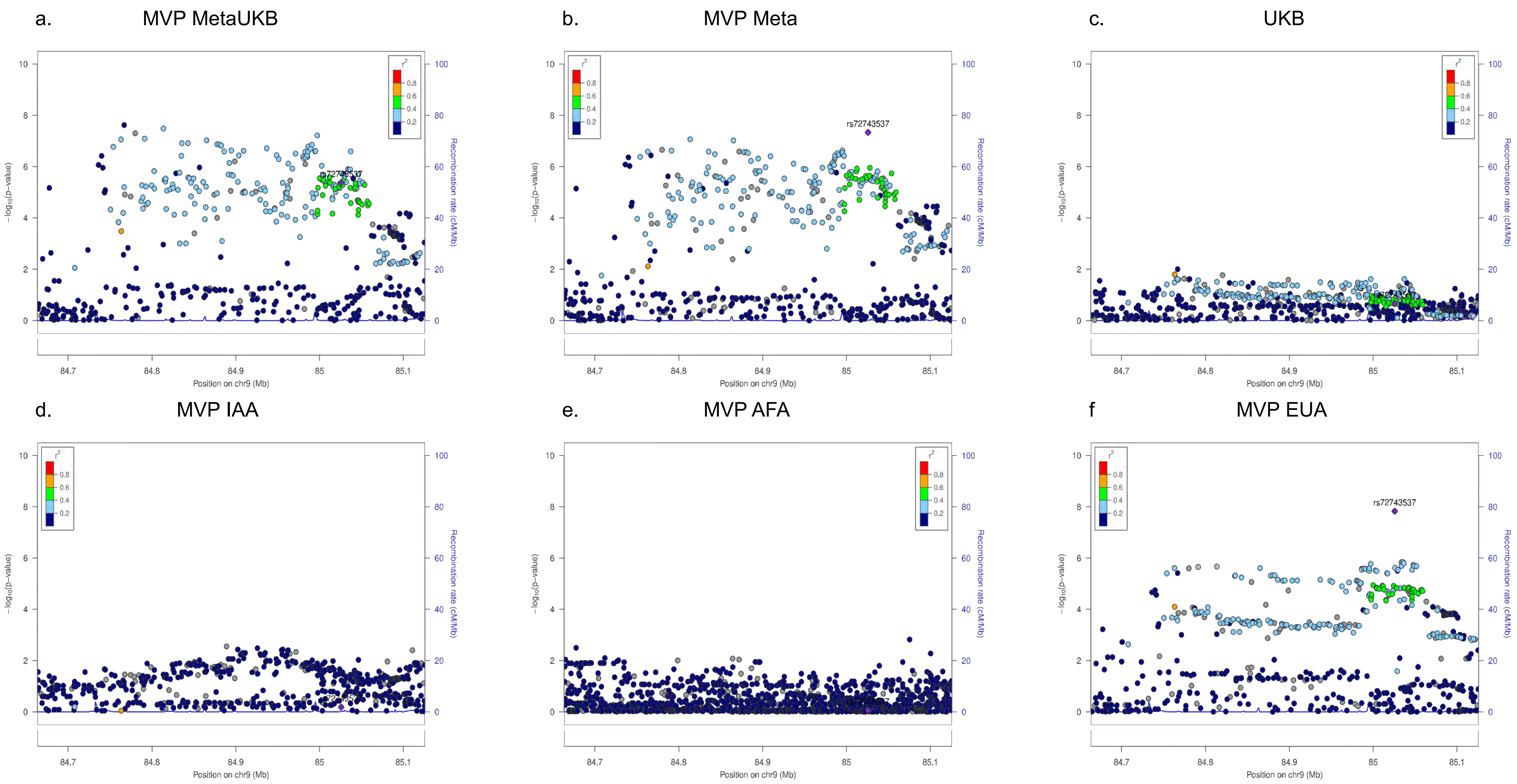

# locus069 | rs10821202

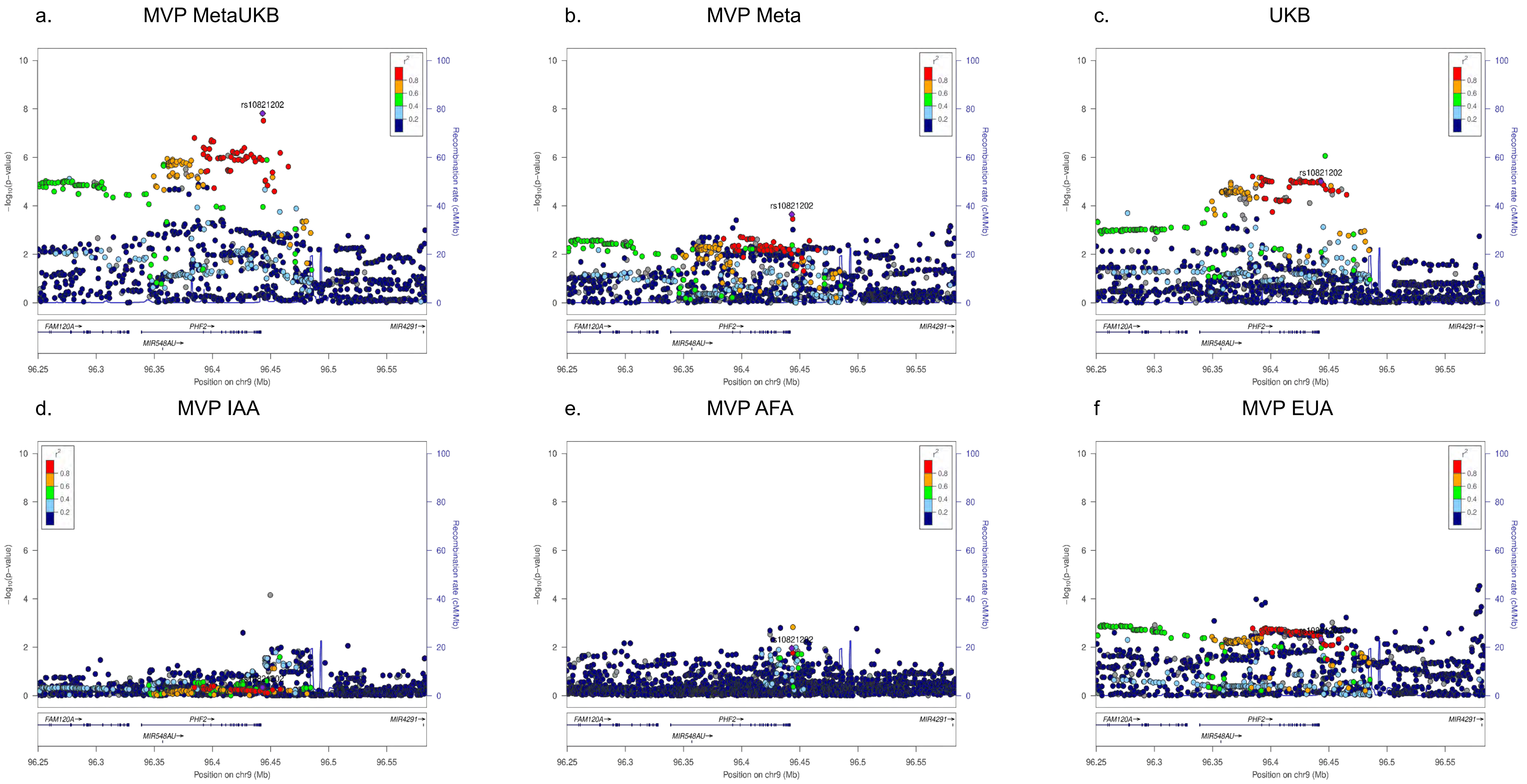

# locus070 | rs12355391

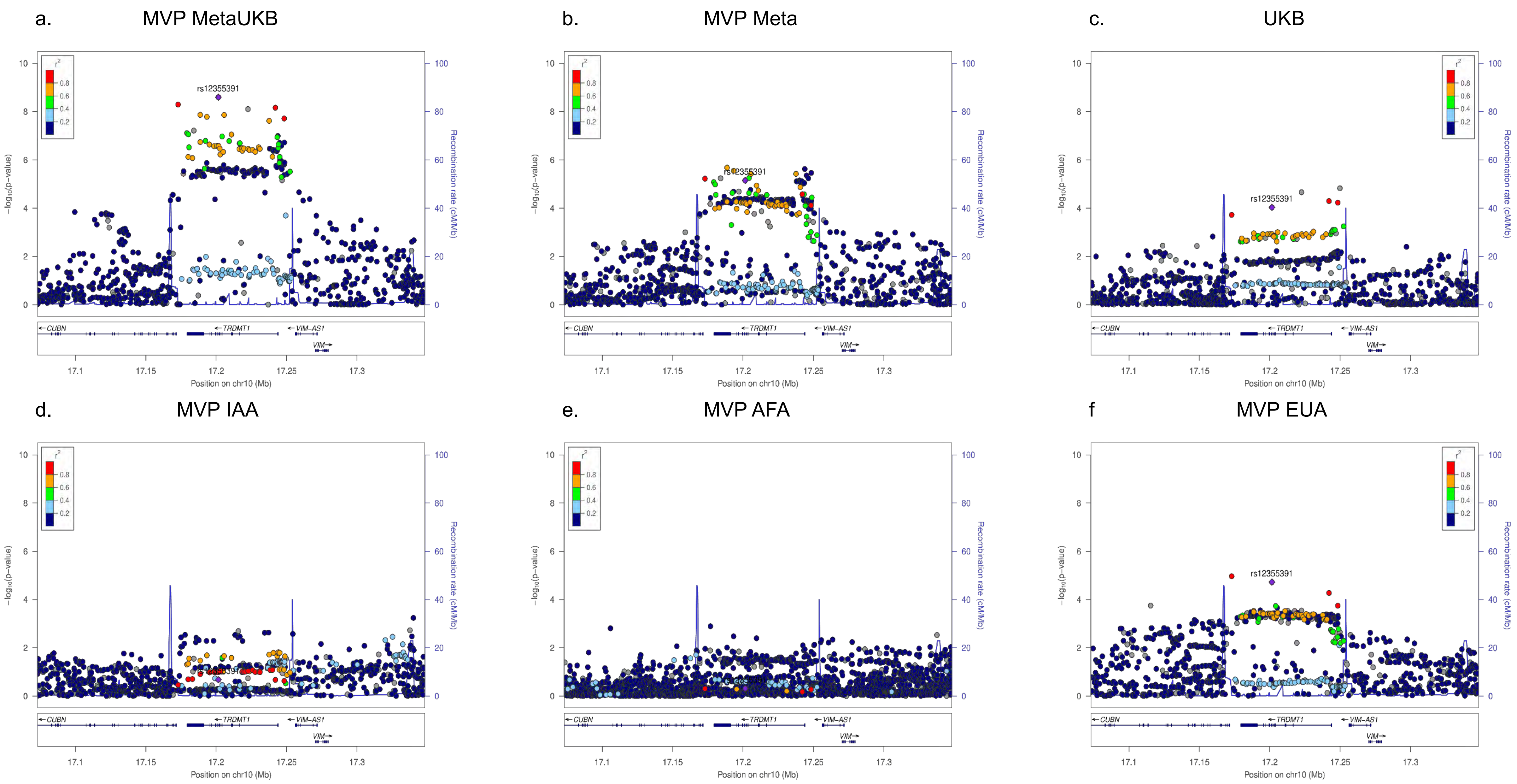

# locus071 | rs2393729

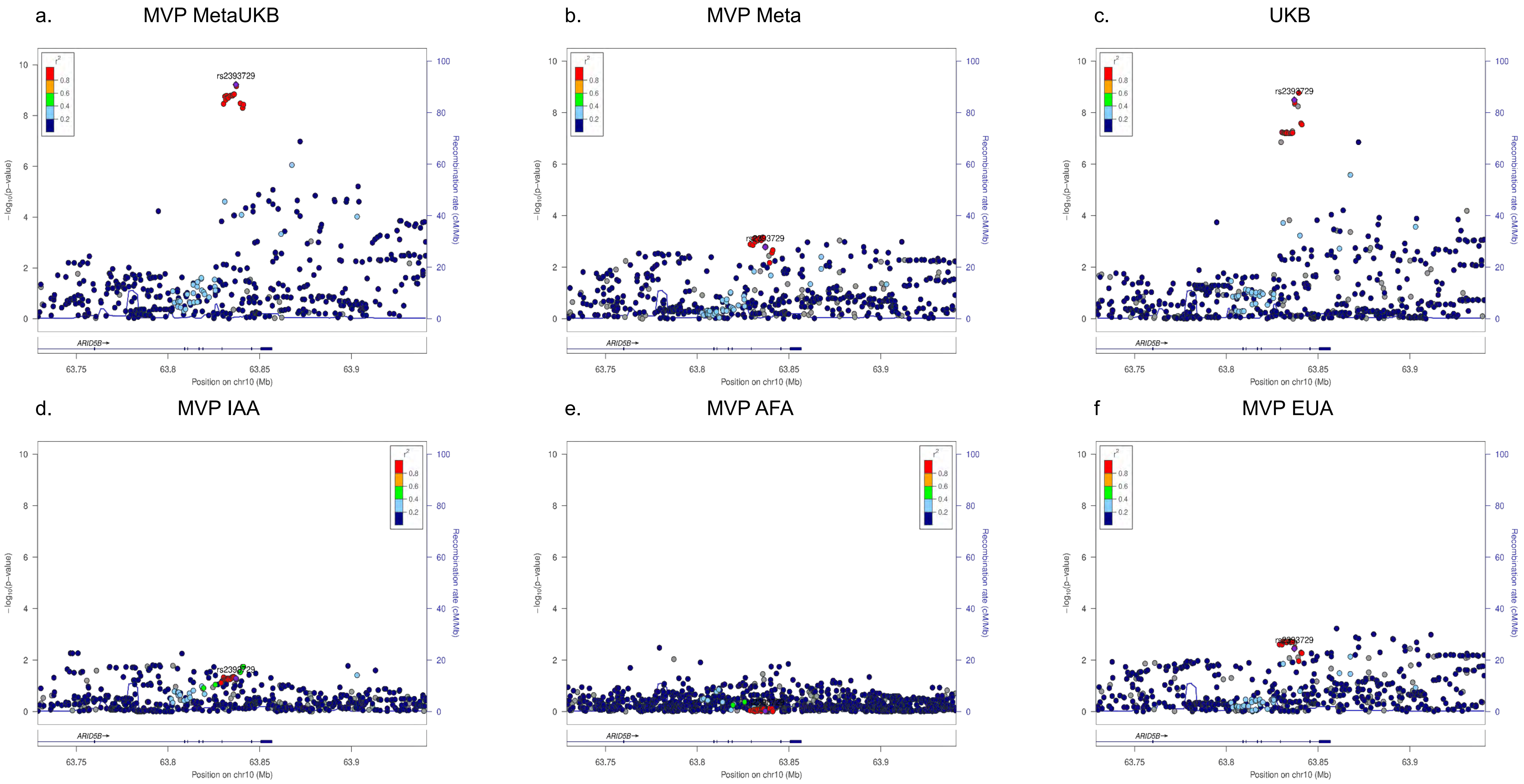

# locus071 | rs4948502

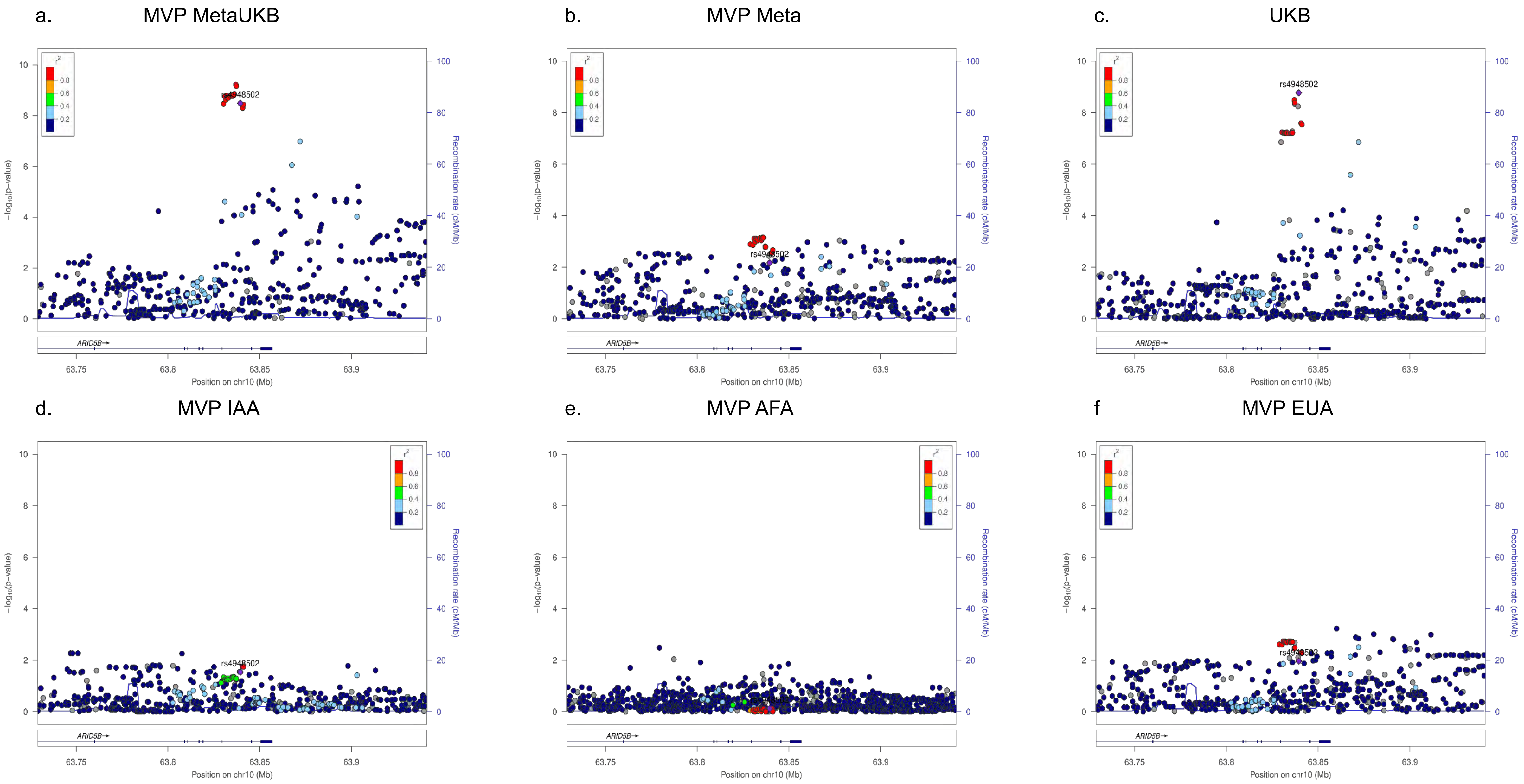

# locus072 | rs143282422

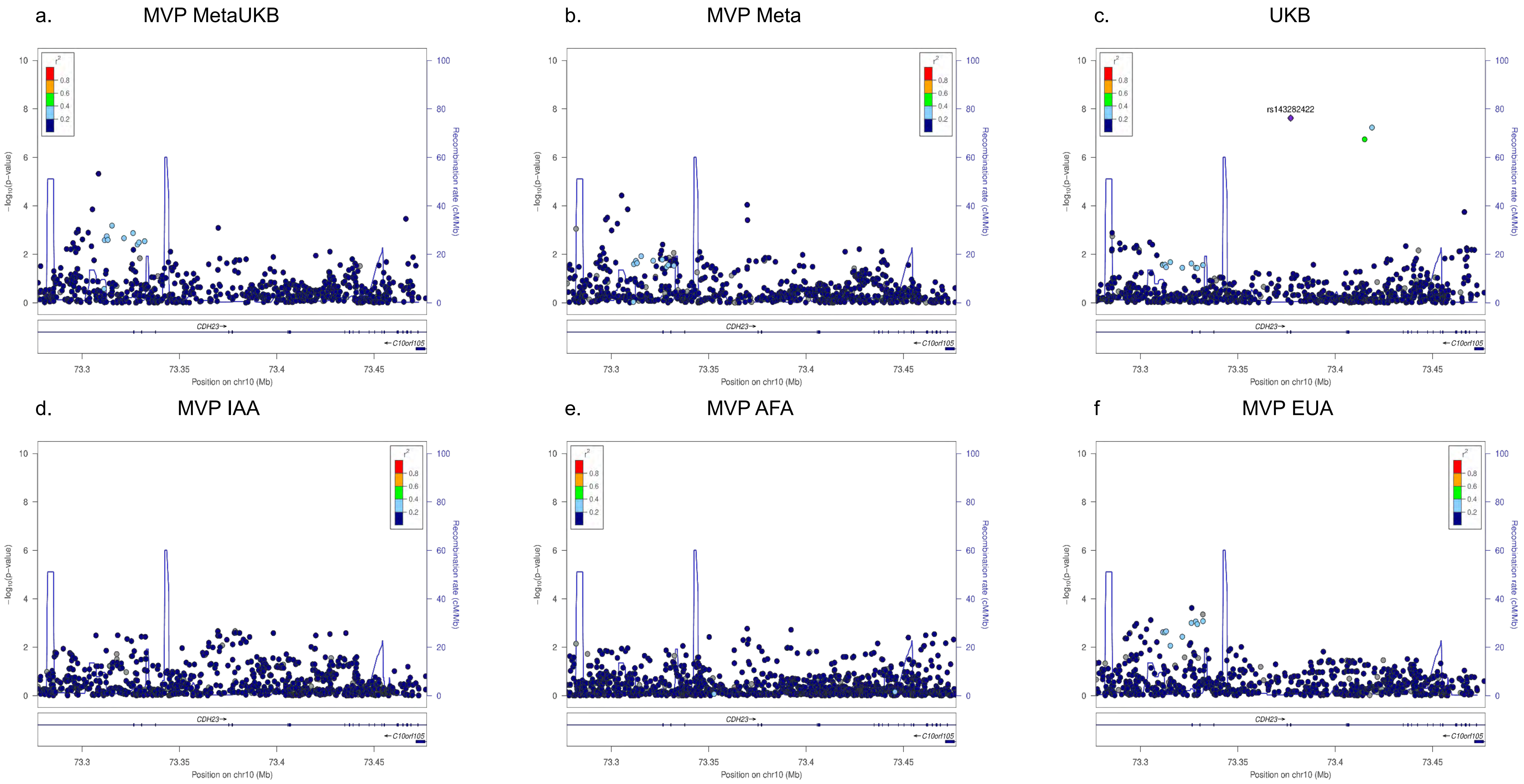

# locus073 | rs2270550

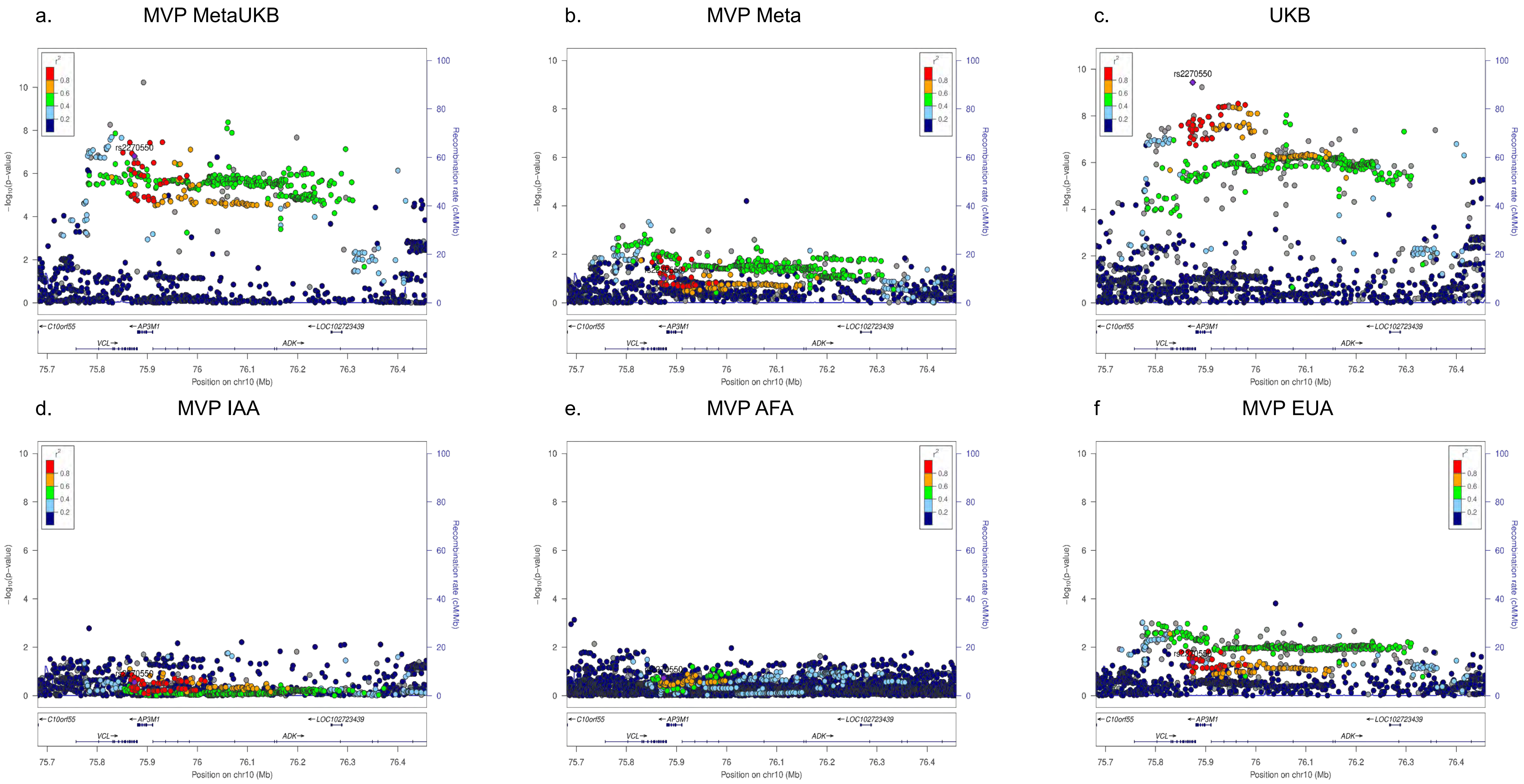

# locus073 | rs377150672

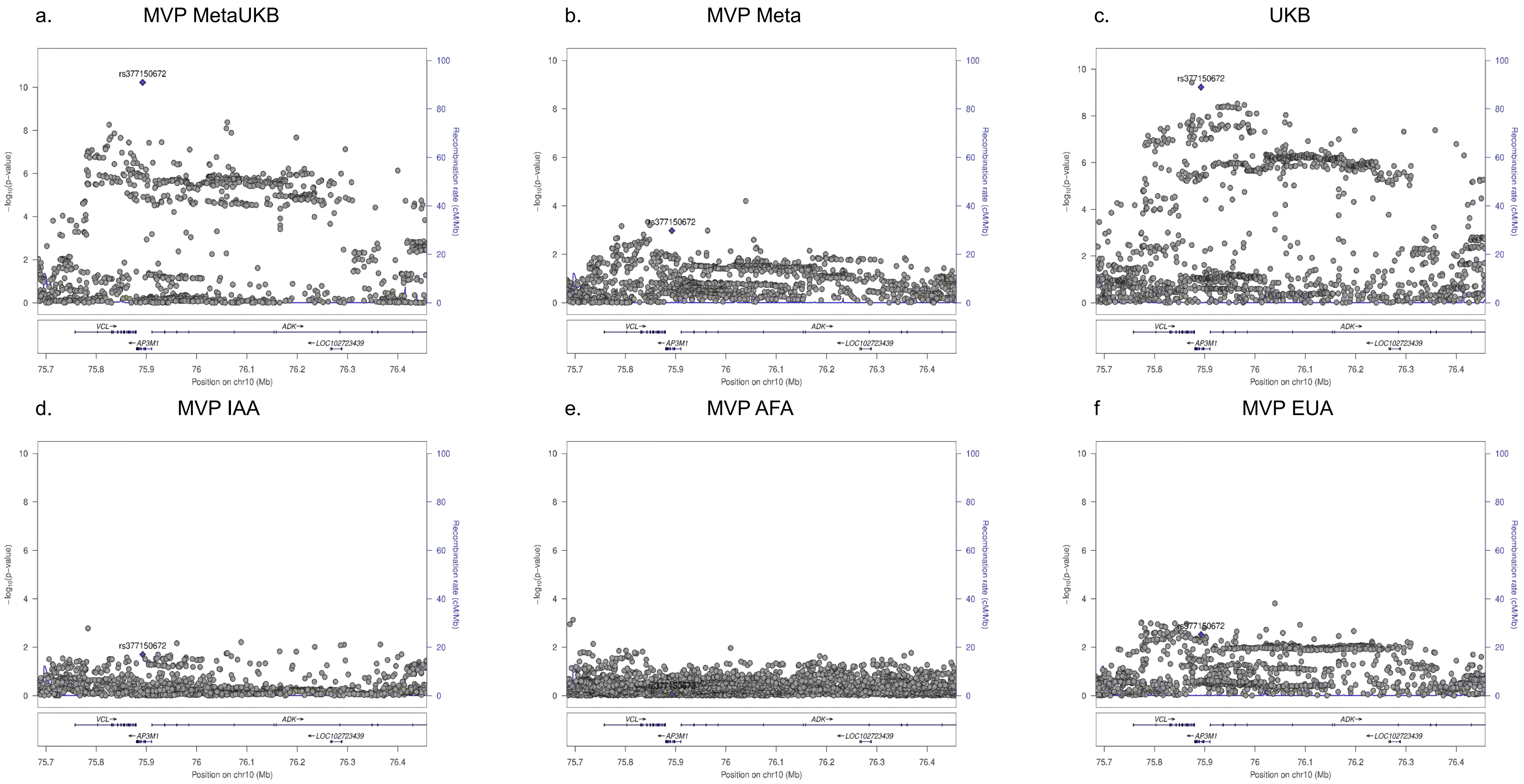

# locus074 | rs11596052

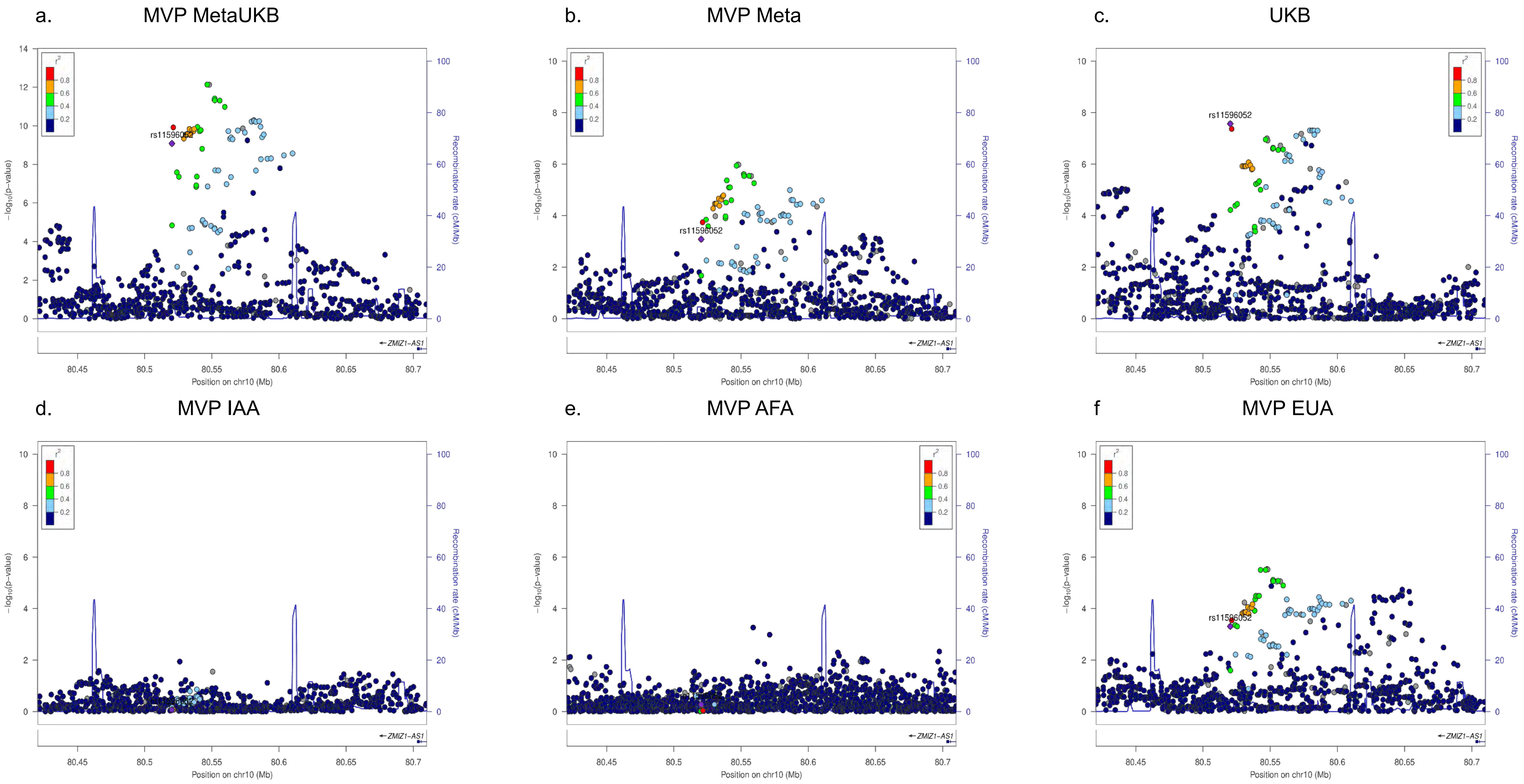

# locus074 | rs149979867

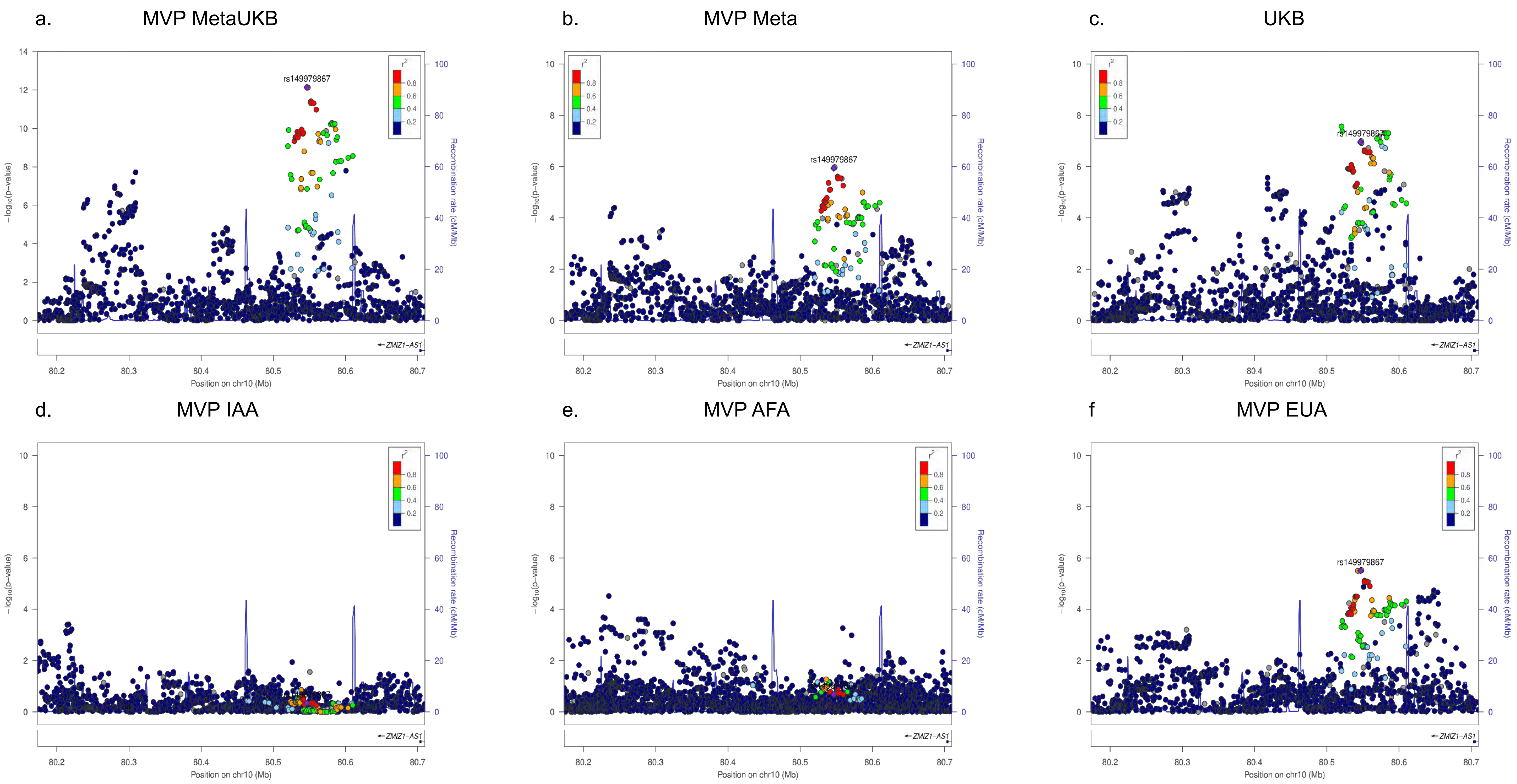

# locus075 | rs71483314

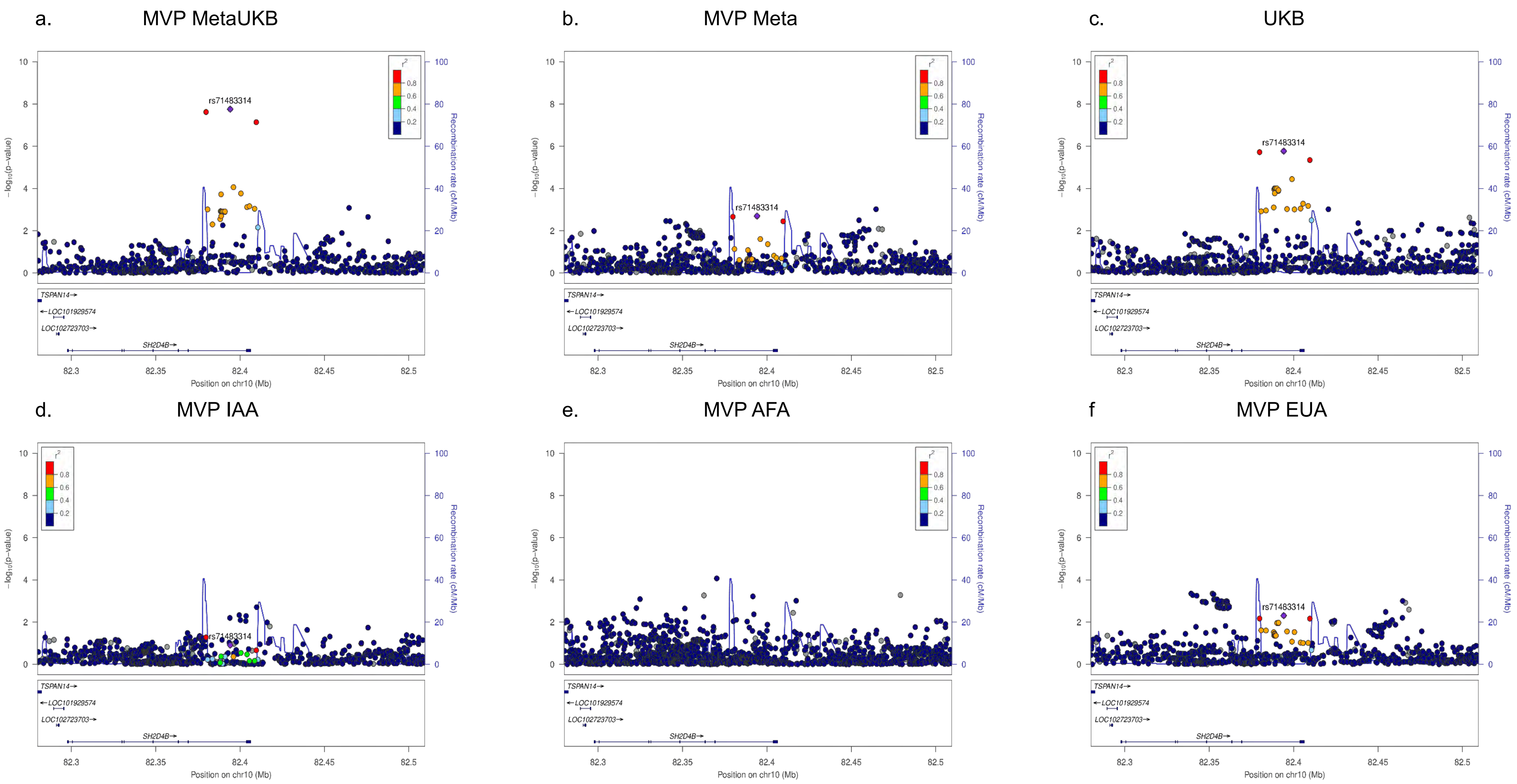

# locus076 | rs12771031

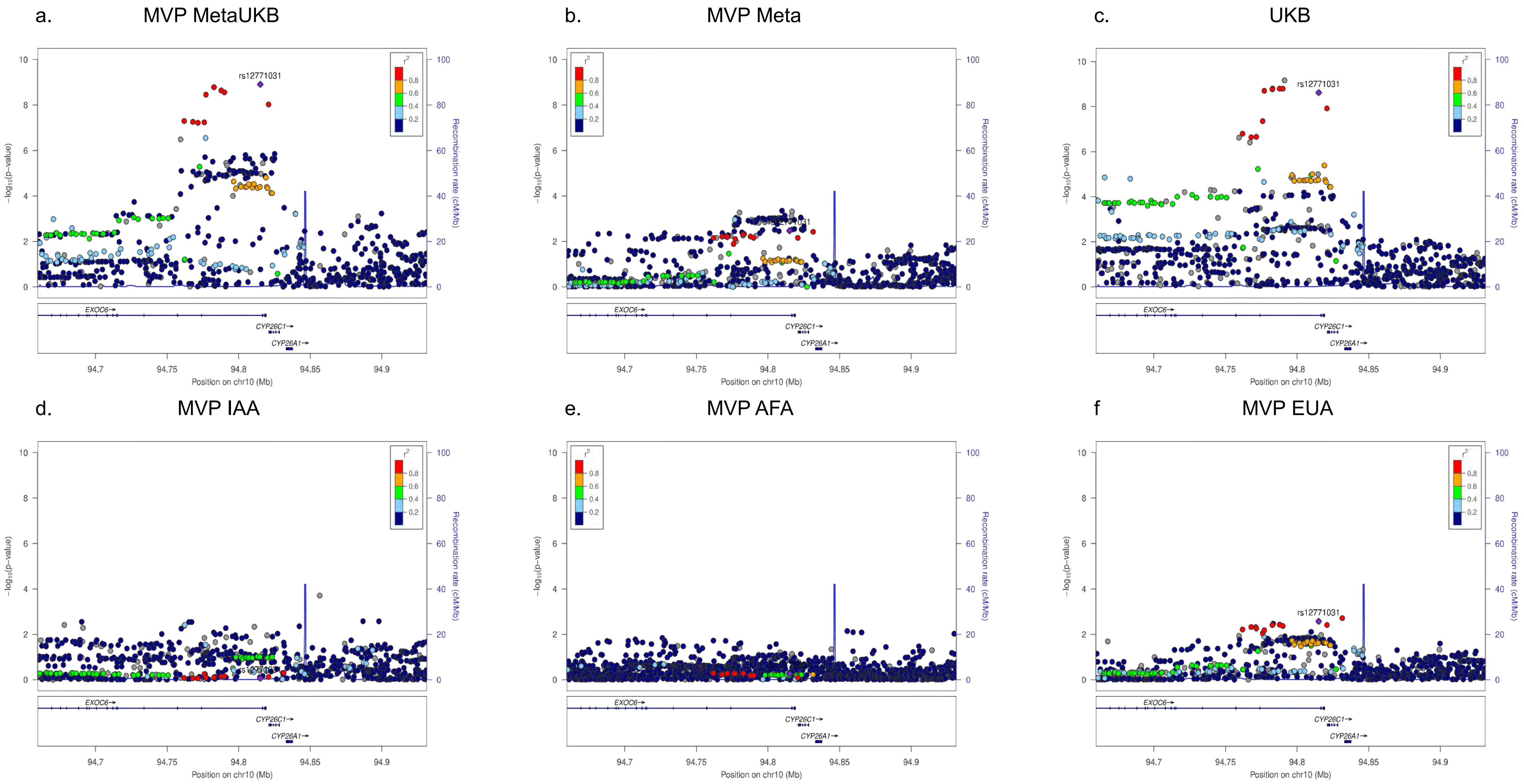

# locus076 | rs200532200

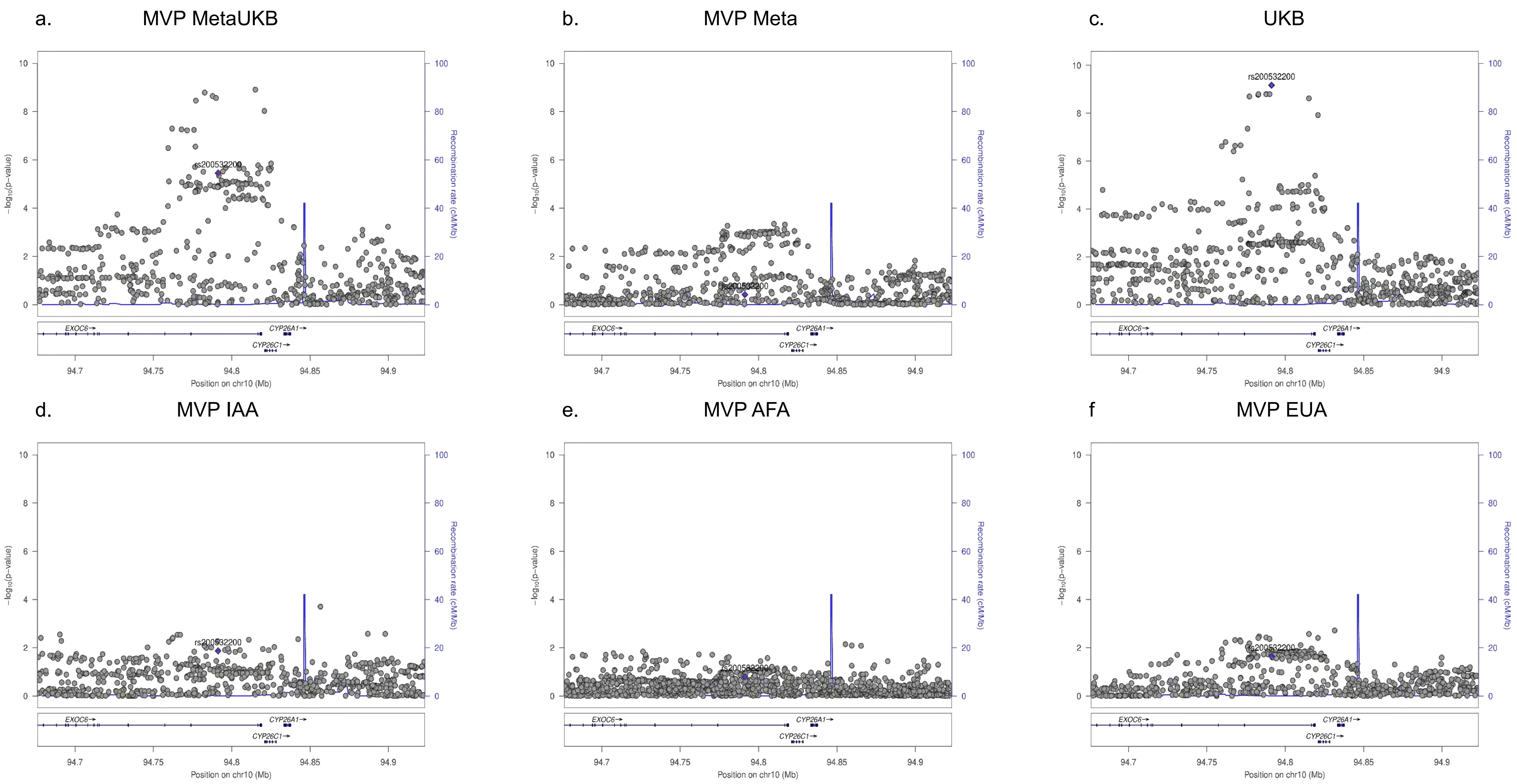

# locus077 | rs7895657

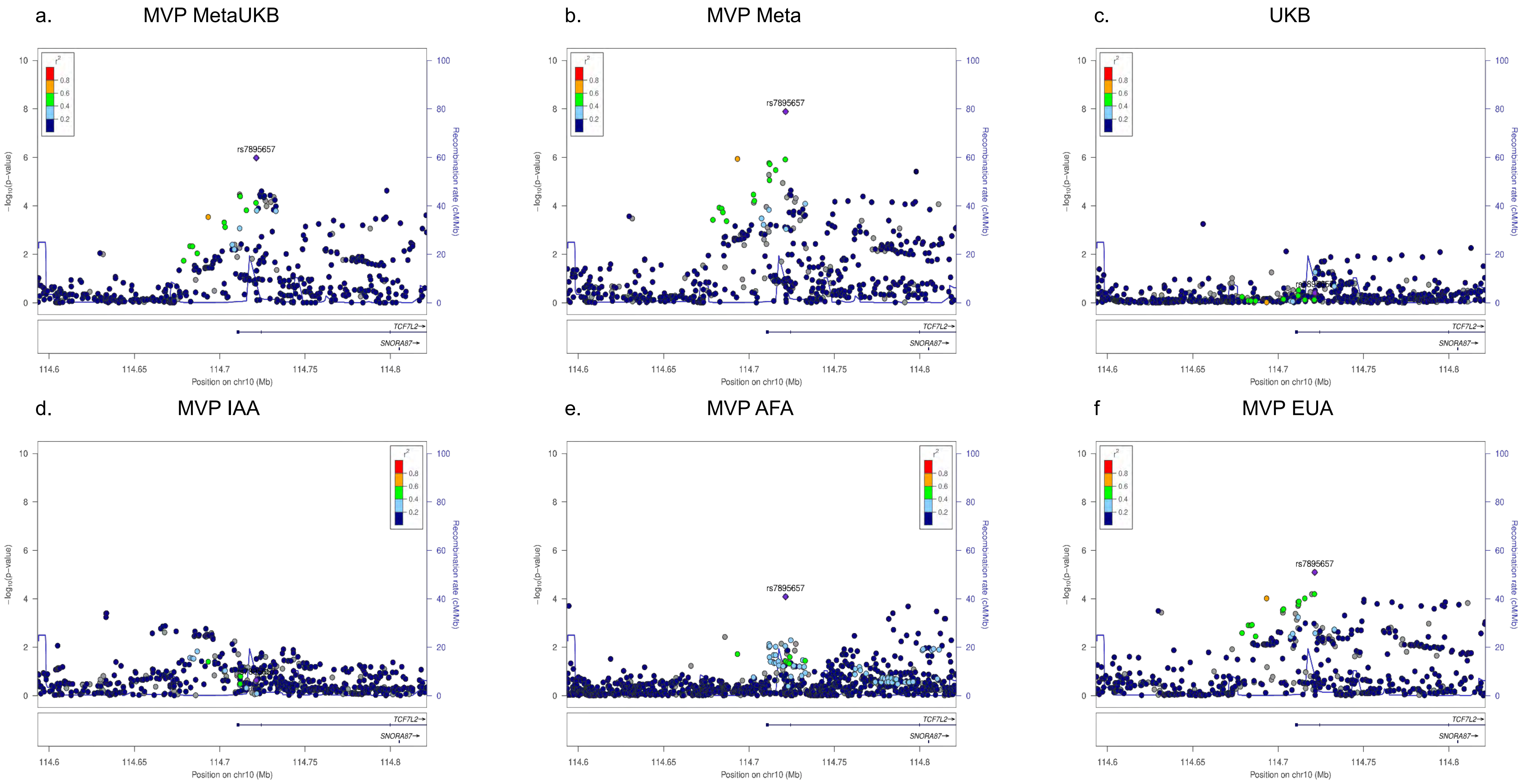

# locus078 | rs10901863

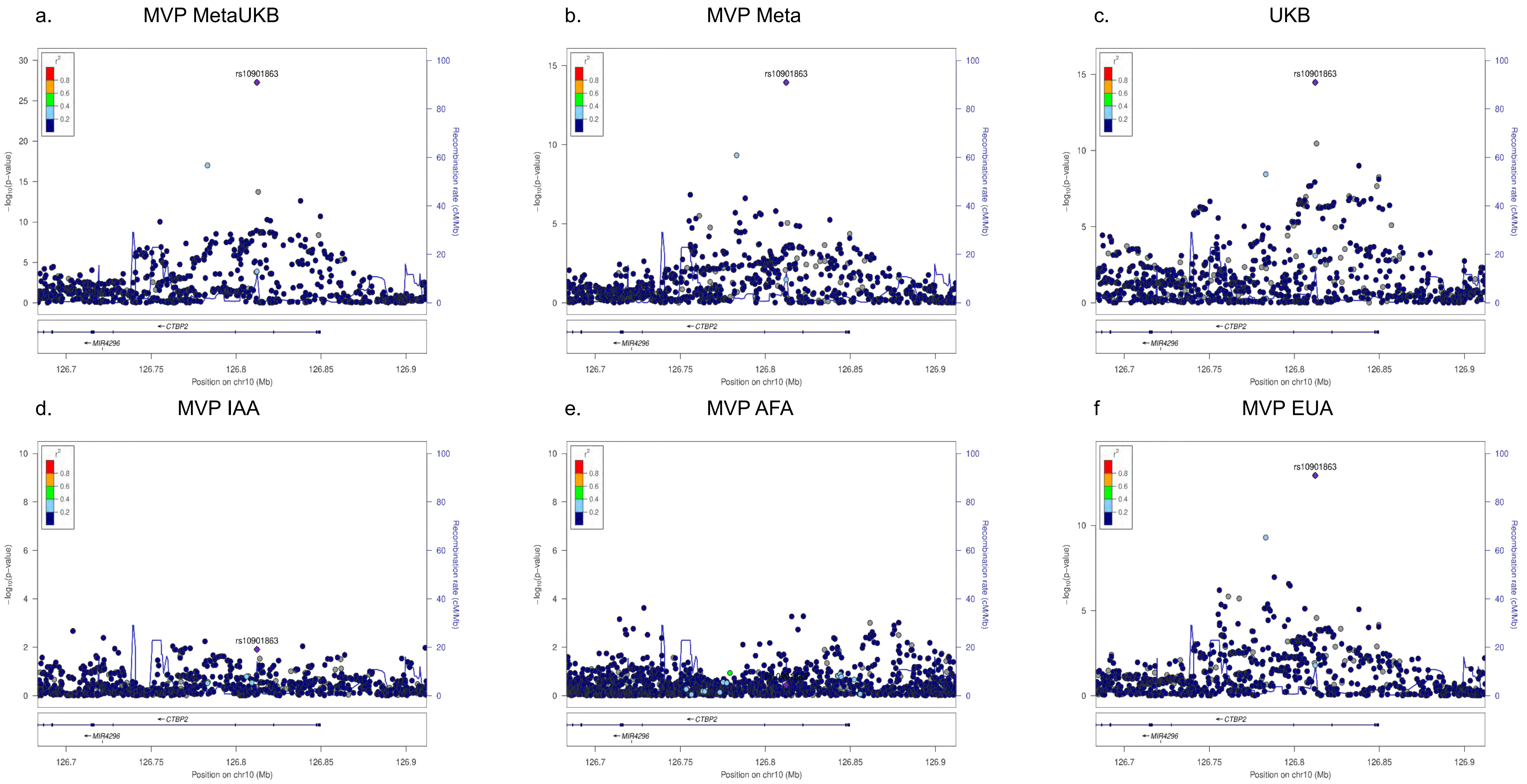

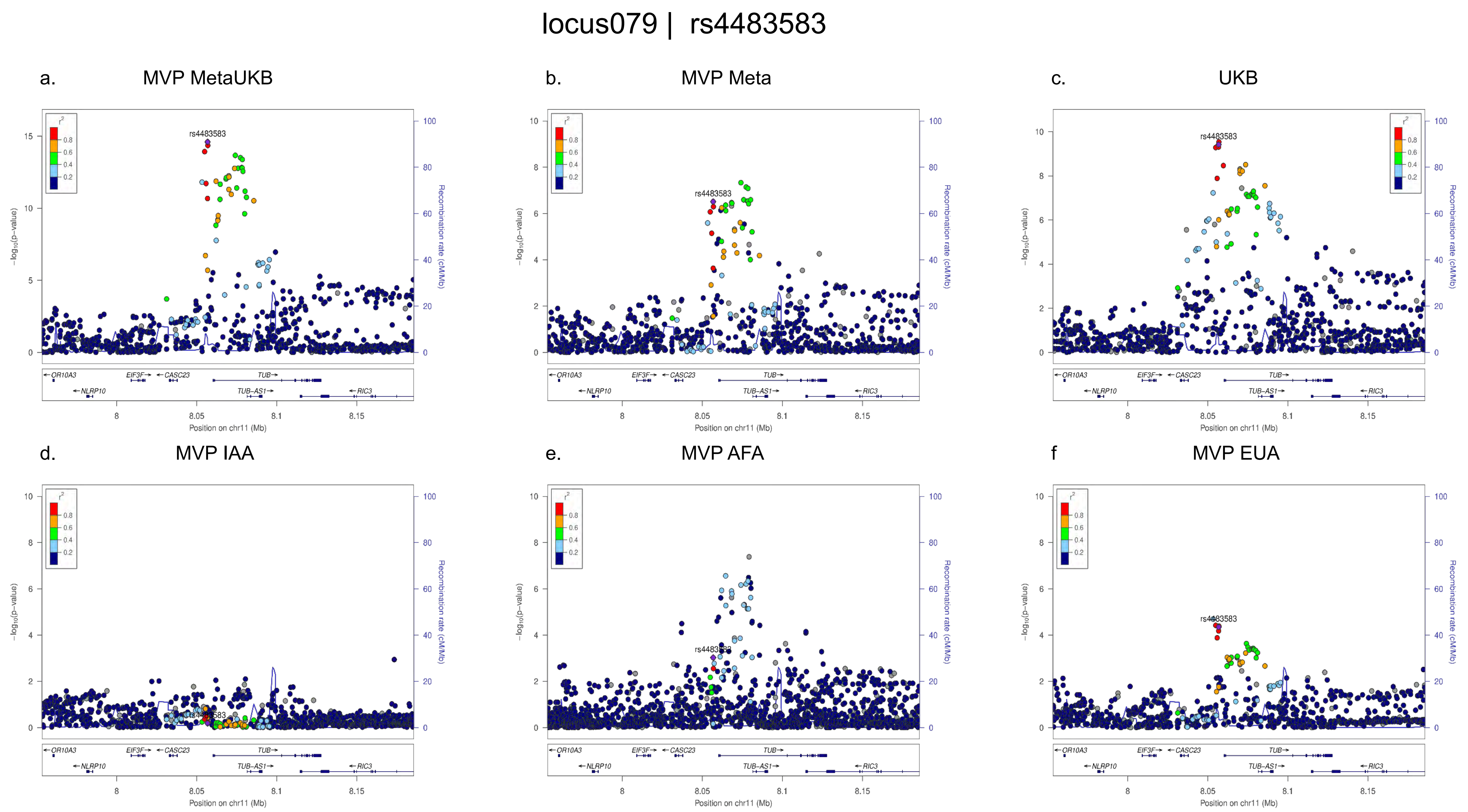
locus079 | rs4483583
a.
MVP MetaUKB
b.
MVP Meta
c.
UKB
d.
MVP IAA
e.
MVP AFA
f
MVP EUA
rs4483583
−log10(p−value)
Recombination rate (cM/Mb)
Position on chr11 (Mb)
OR10A3
NLRP10
EIF3F
CASC23
TUB
TUB−AS1
RIC3

# locus079 | rs55635402

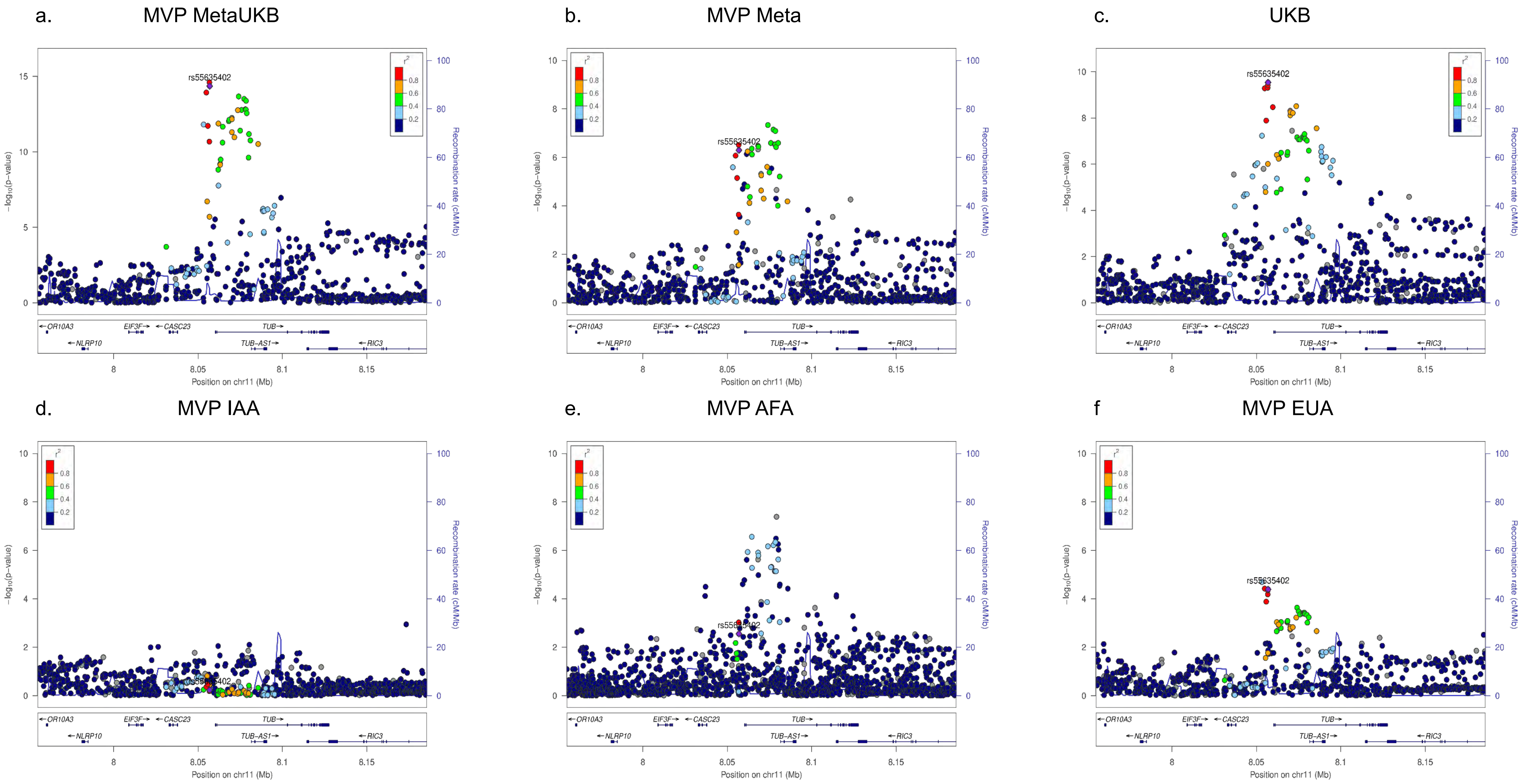

# locus079 | rs59174224

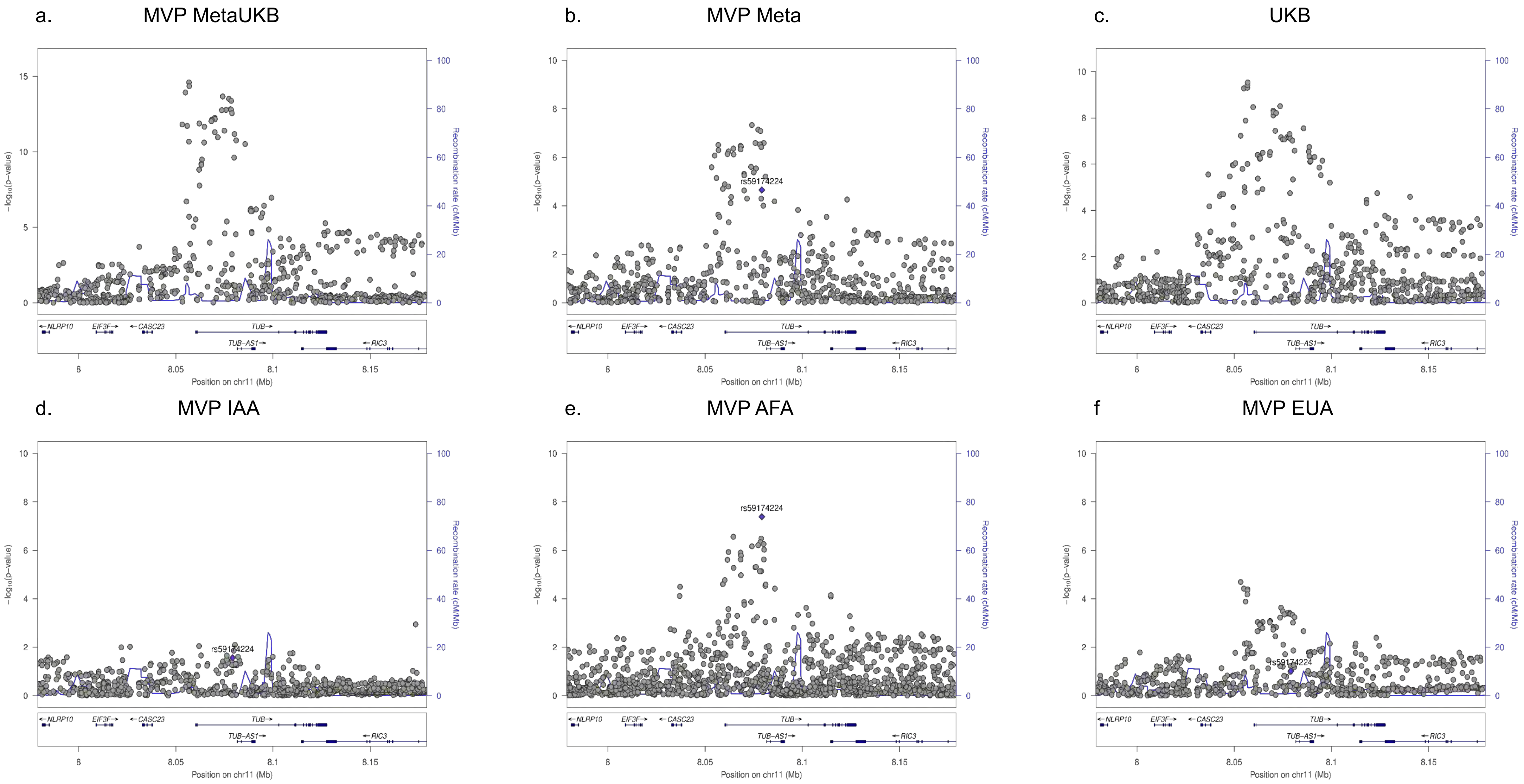

# locus080 | rs4910179

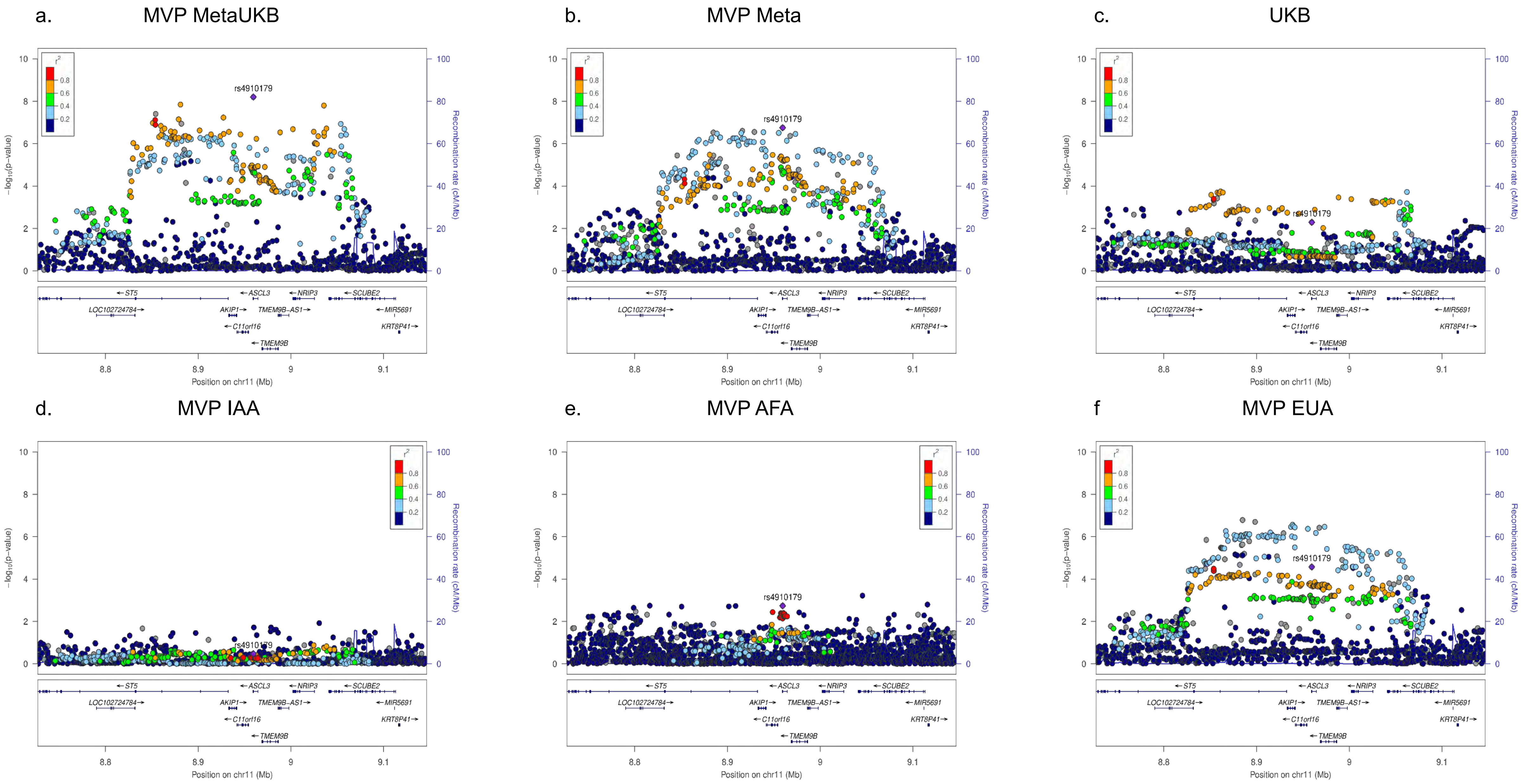

# locus081 | rs10831990

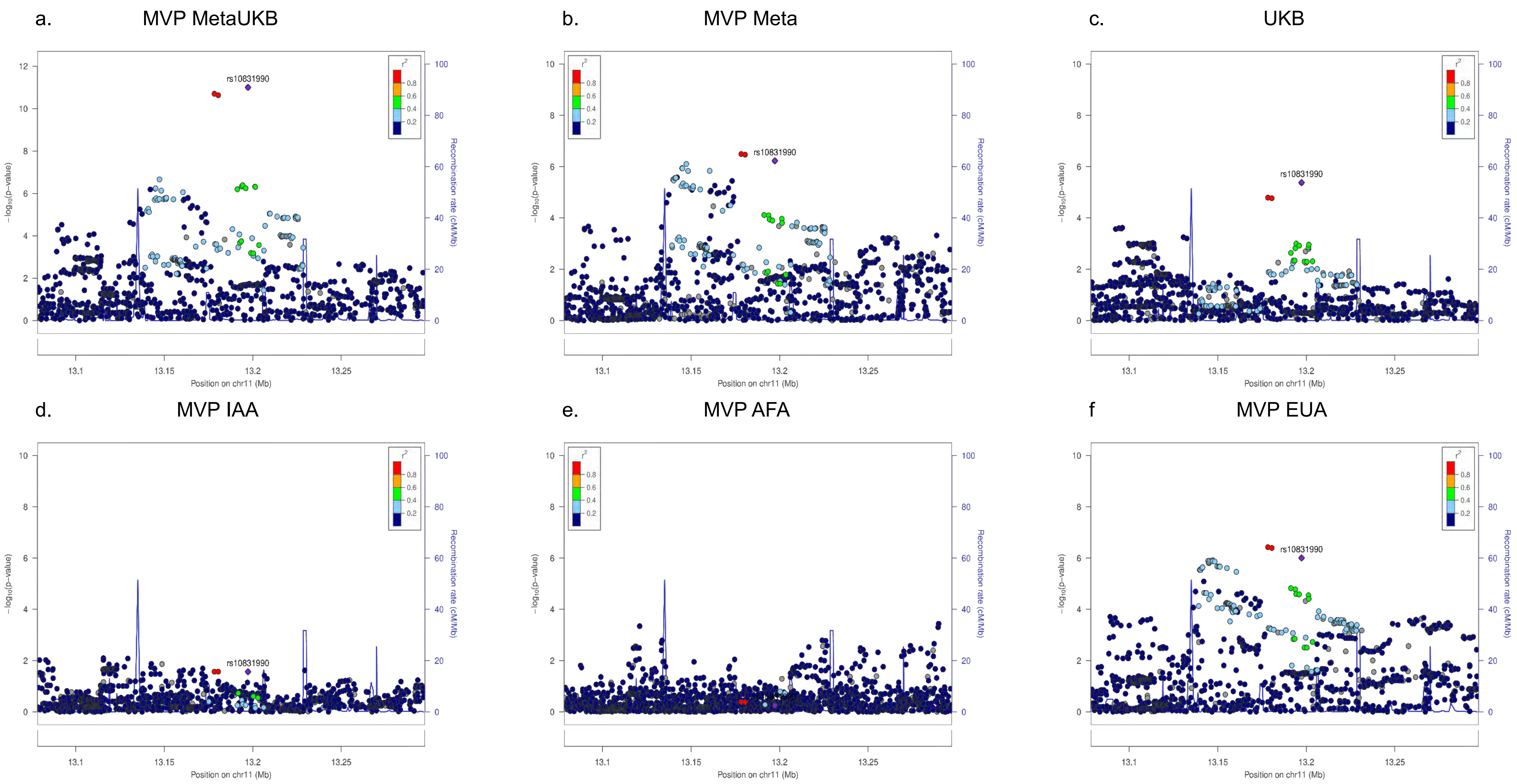

# locus082 | rs11023821

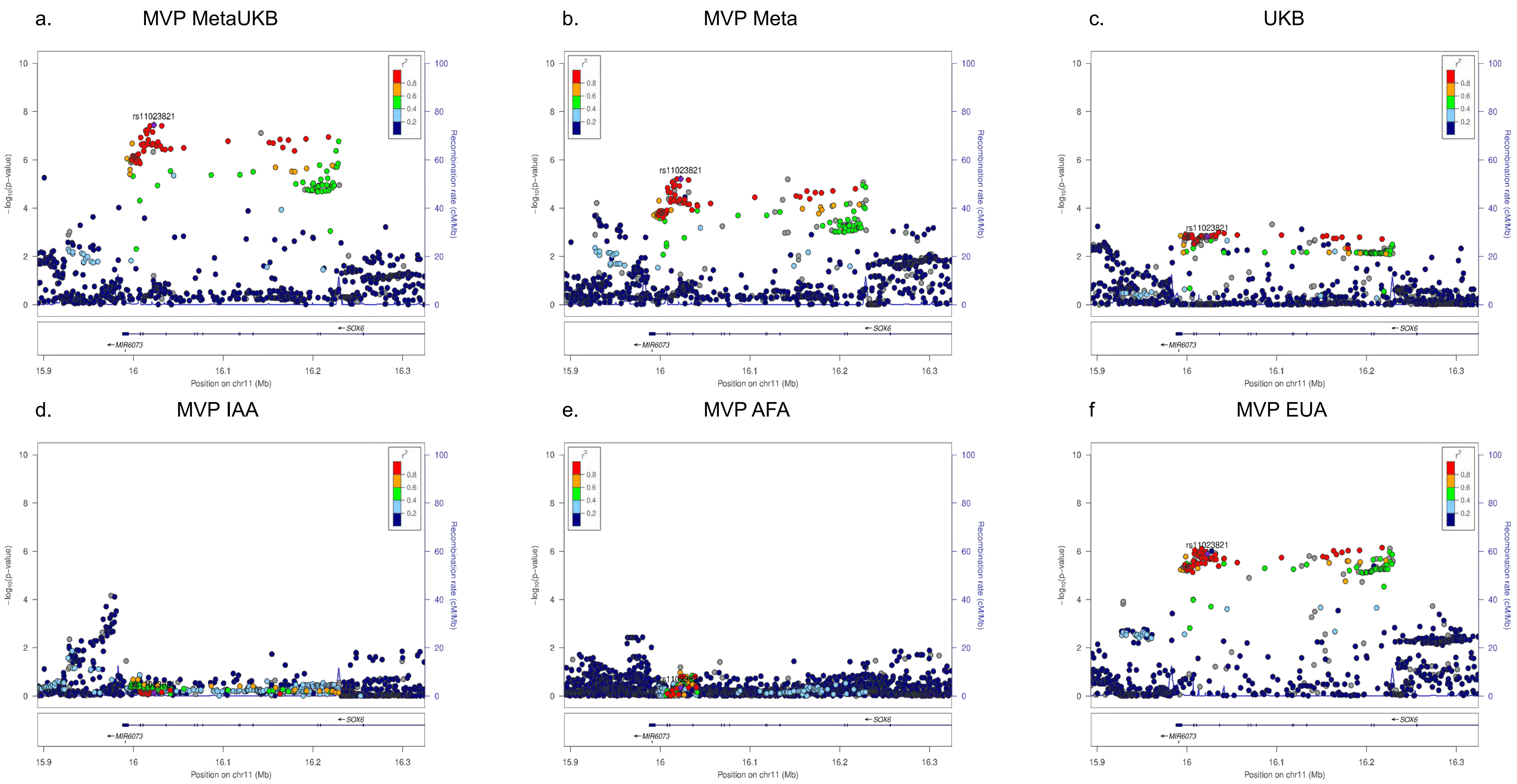

# locus083 | rs11026796

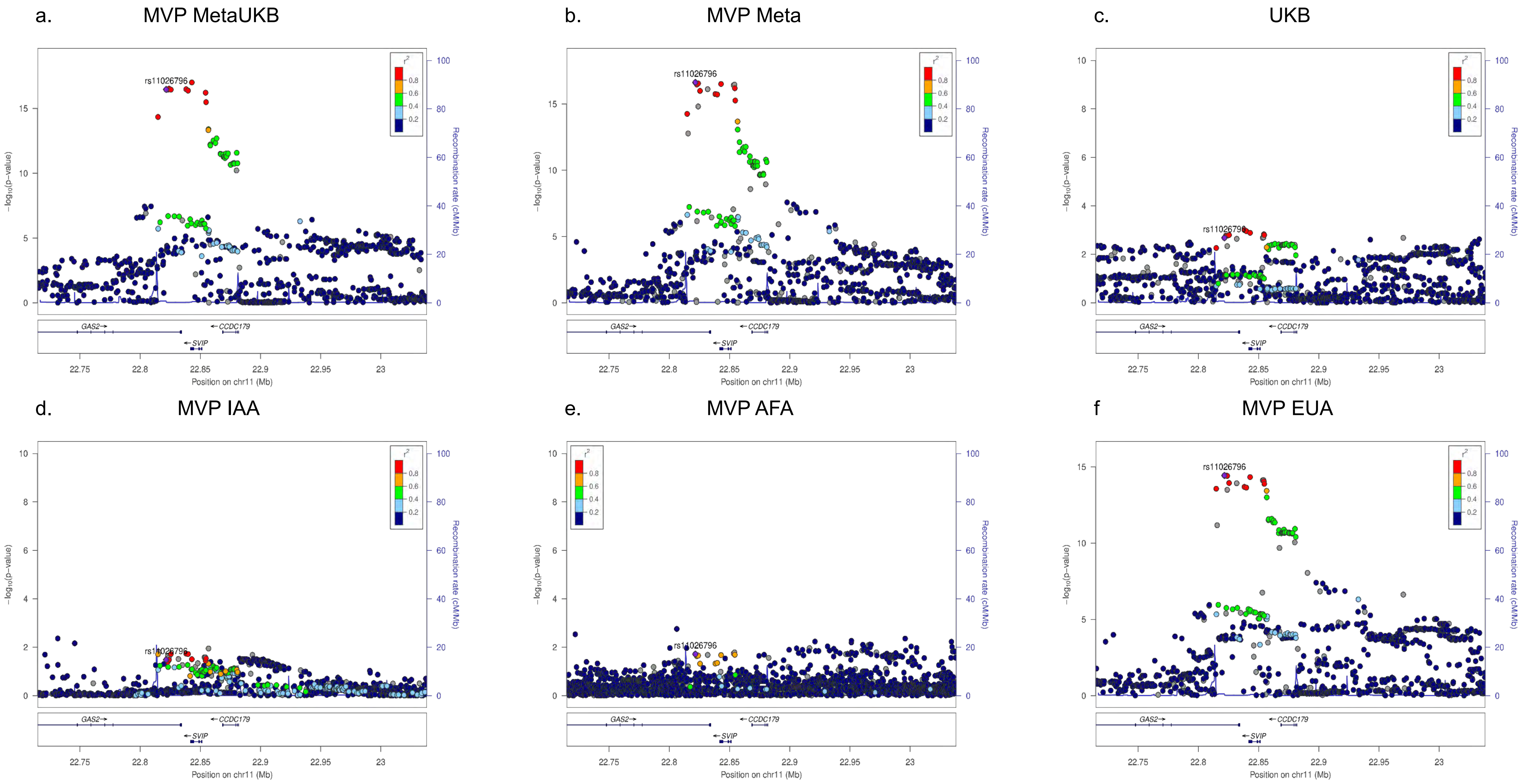

# locus083 | rs11543287

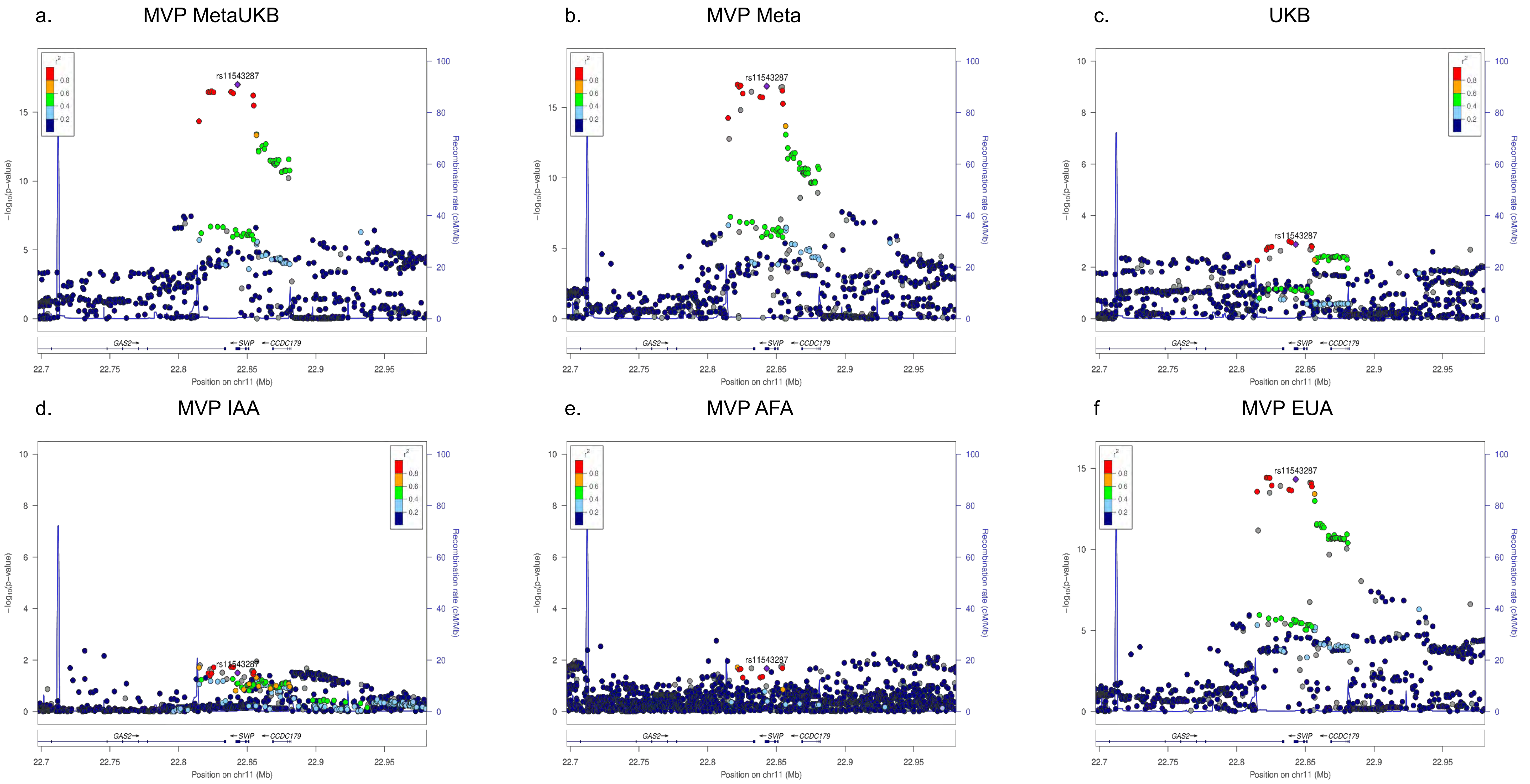

# locus084 | rs141403654

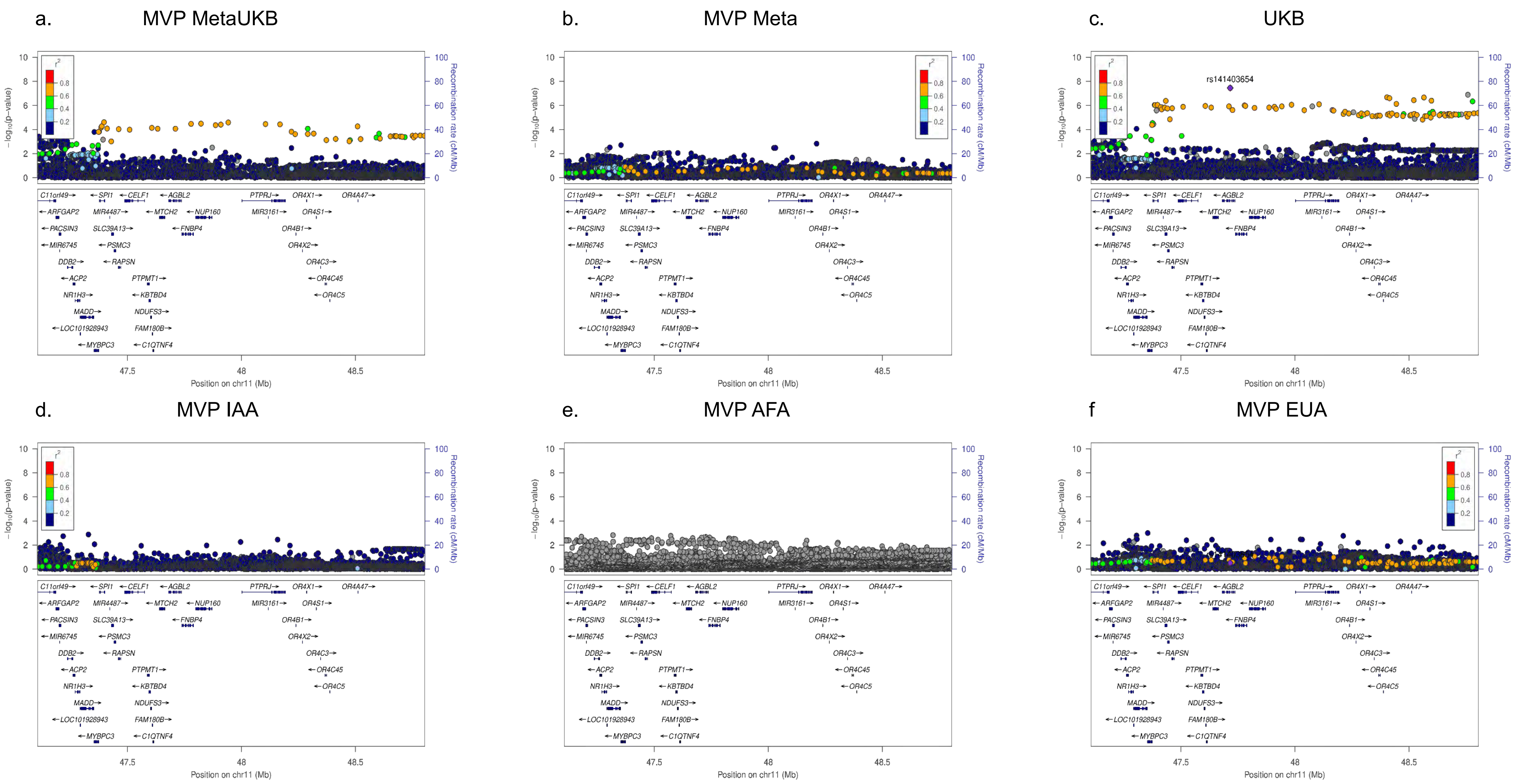

# locus085 | rs1126809

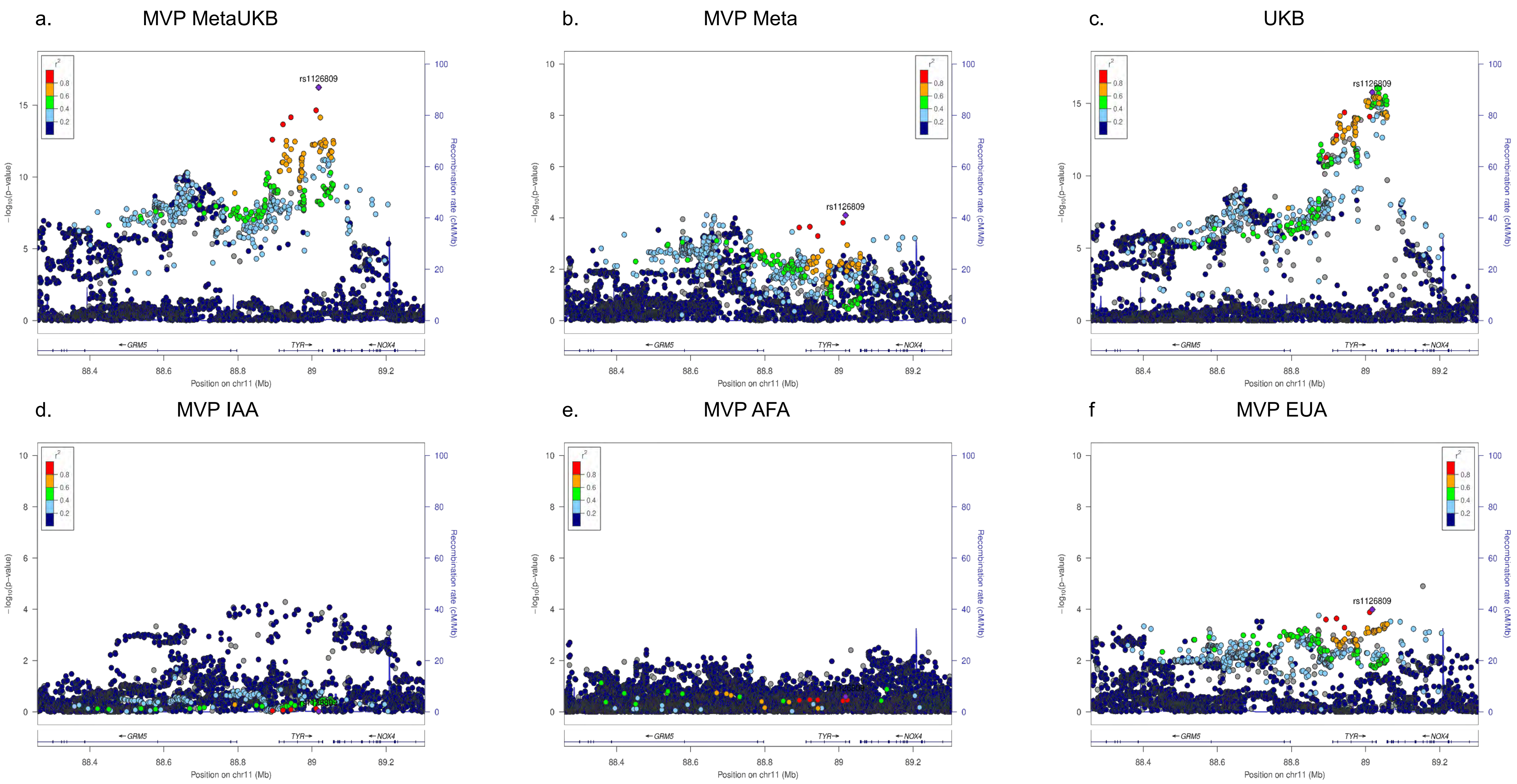

# locus085 | rs7951935

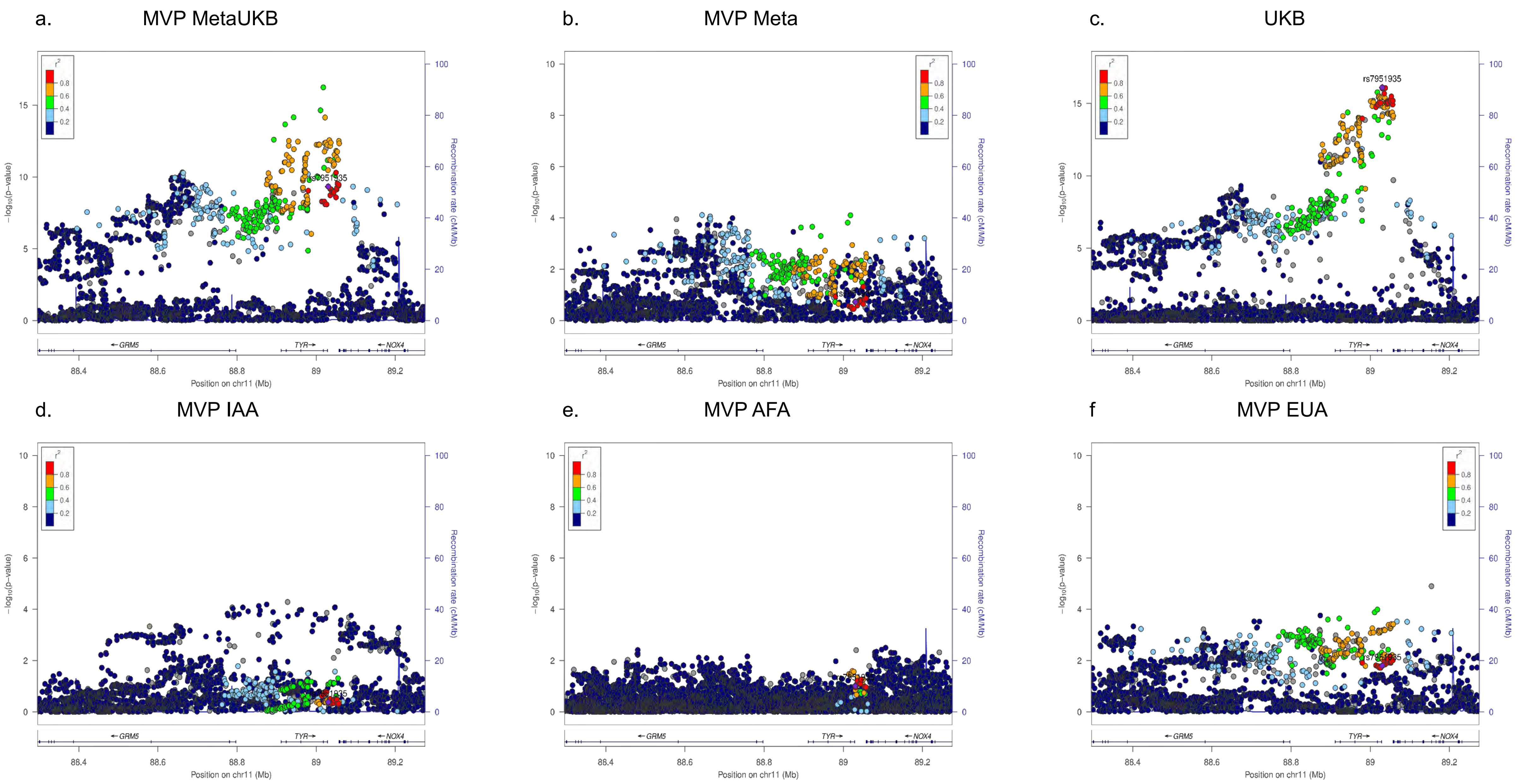

# locus086 | rs67307131

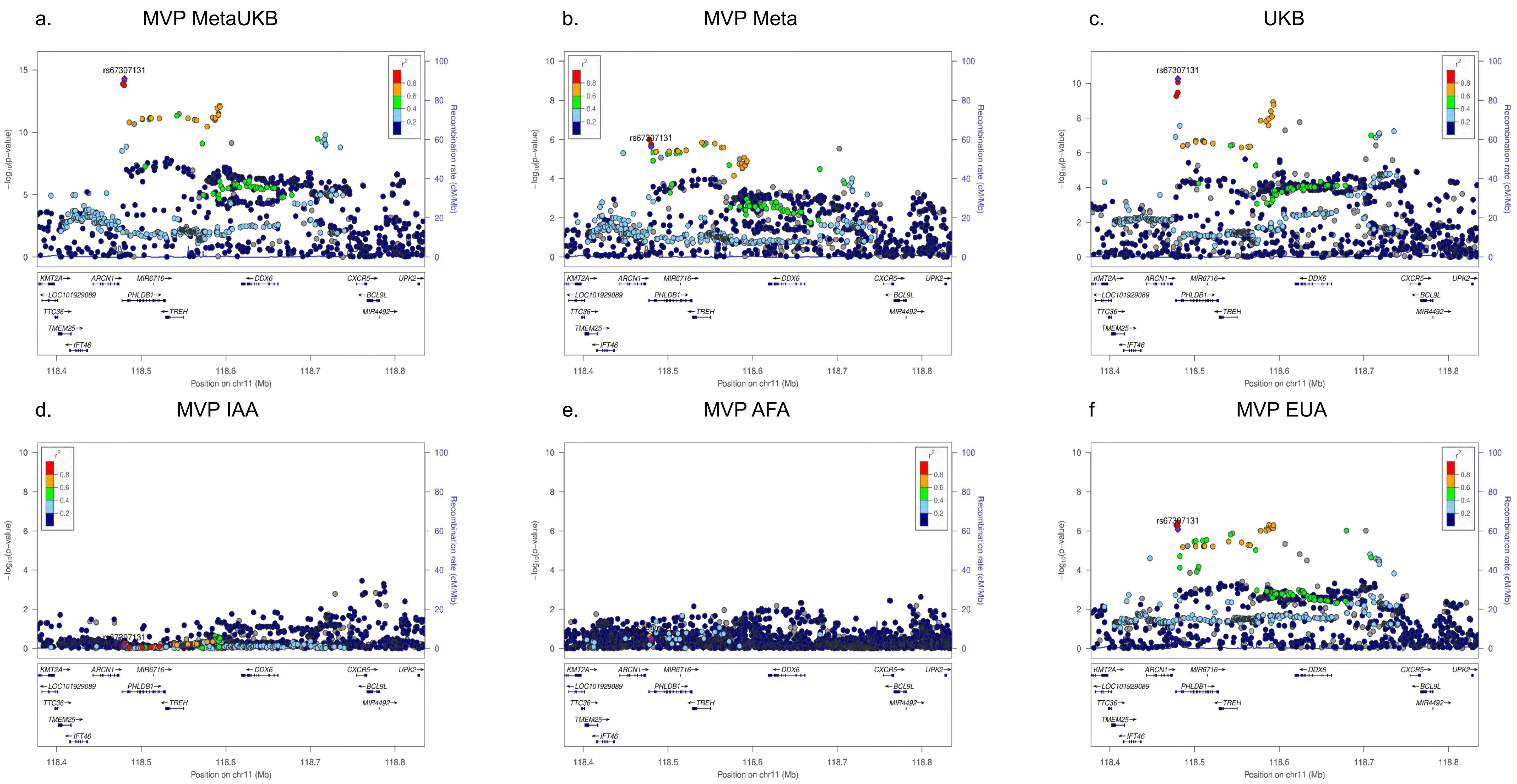

# locus087 | rs11048385

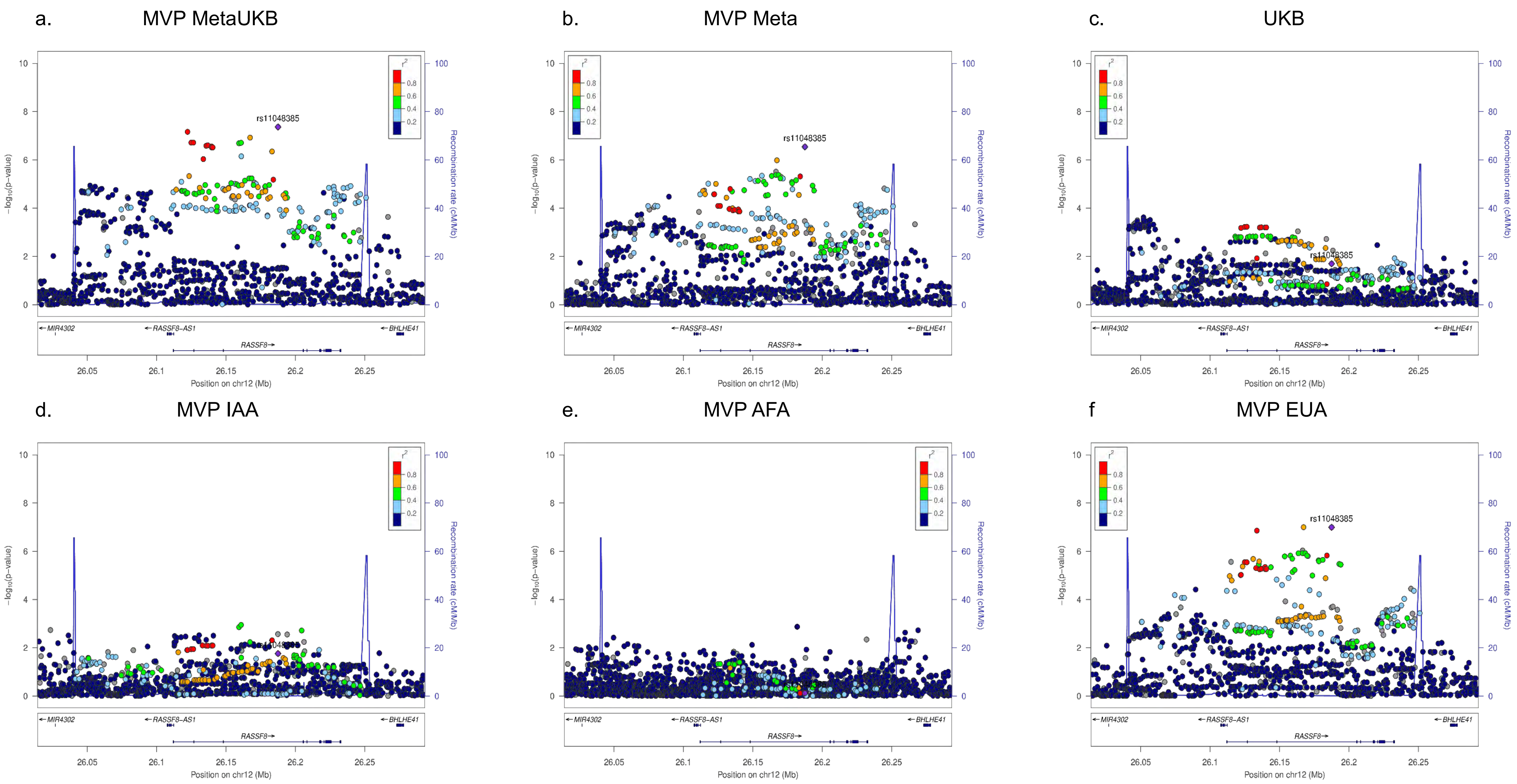

# locus088 | rs2111216

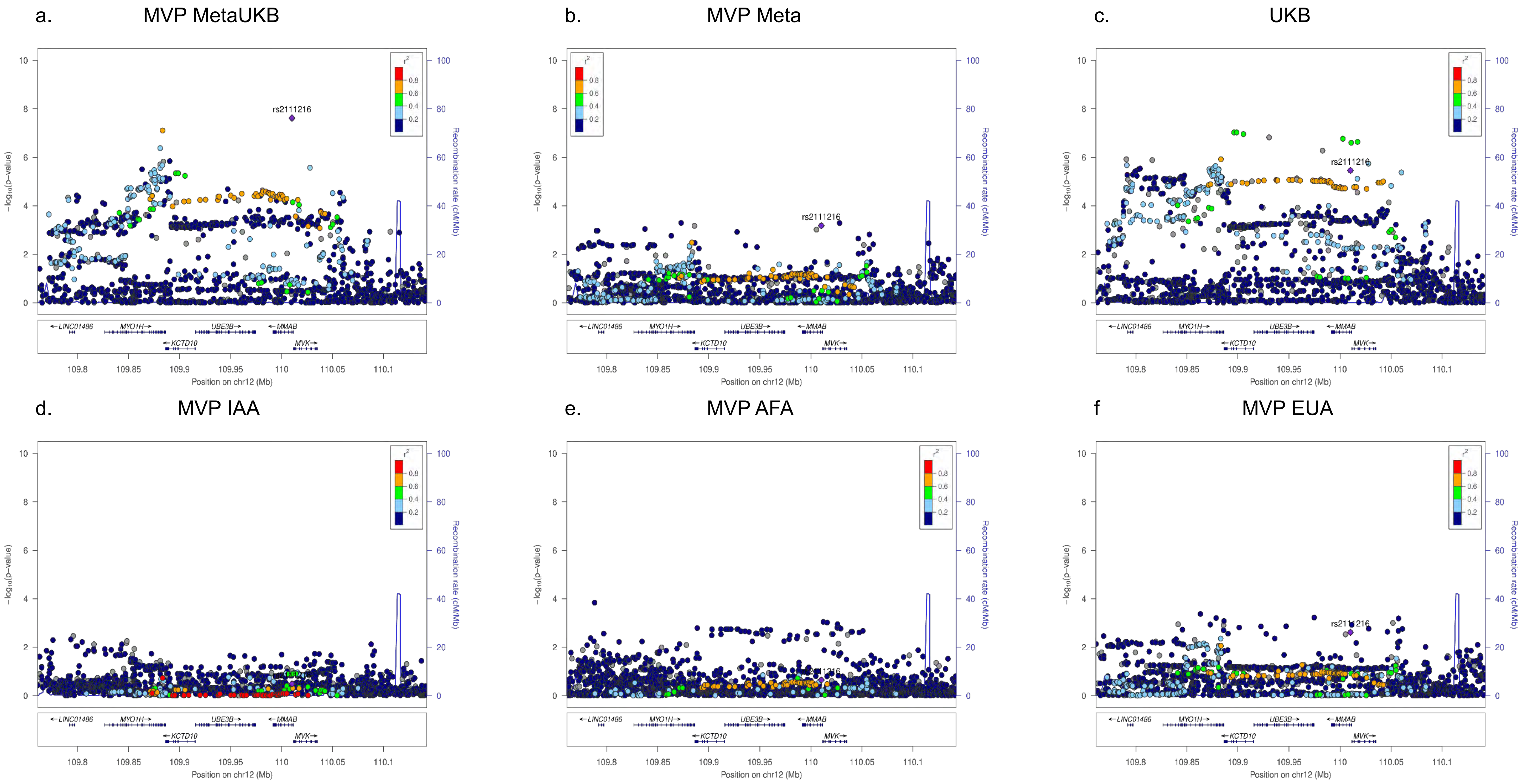

# locus089 | rs111516934

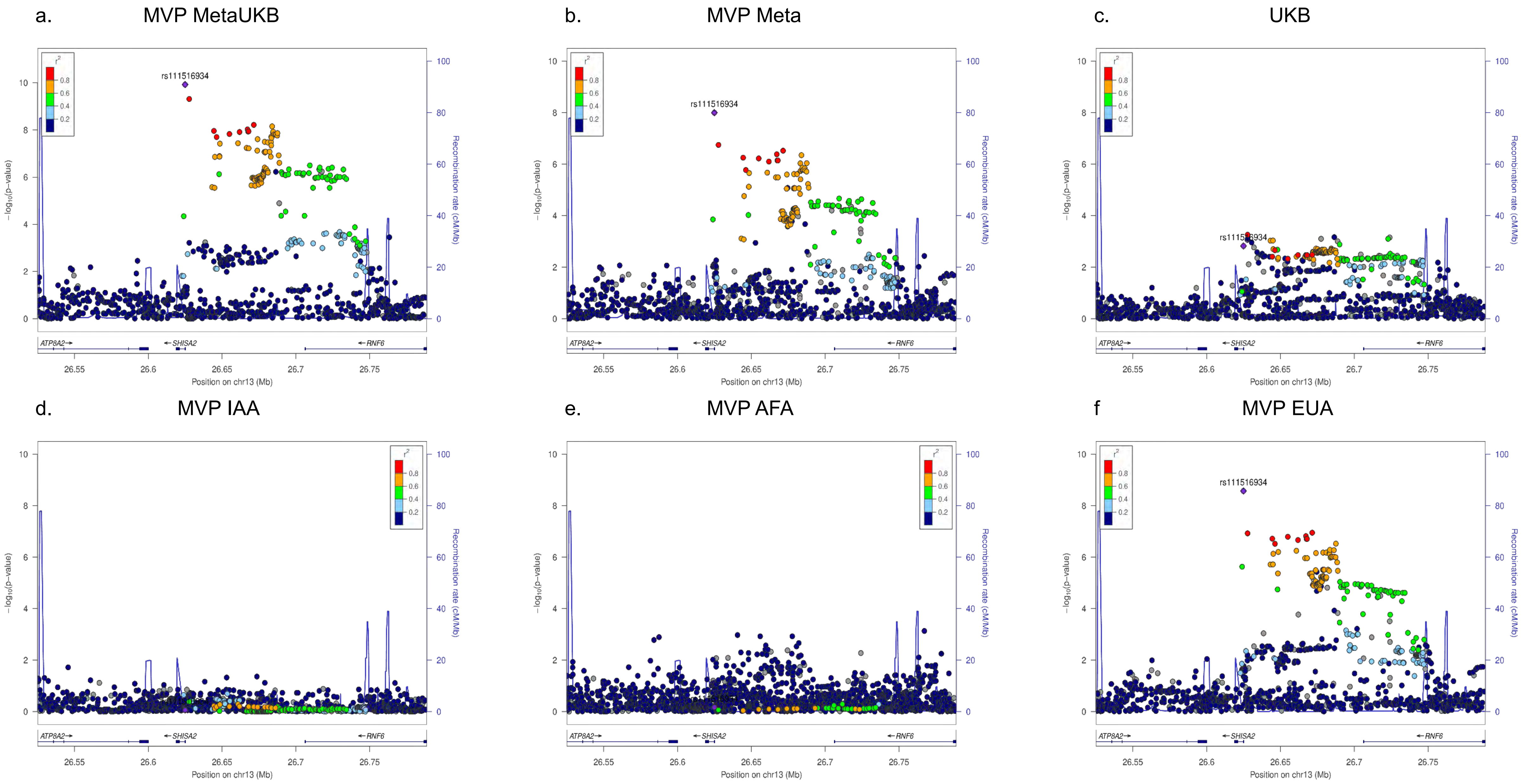

# locus090 | rs12552

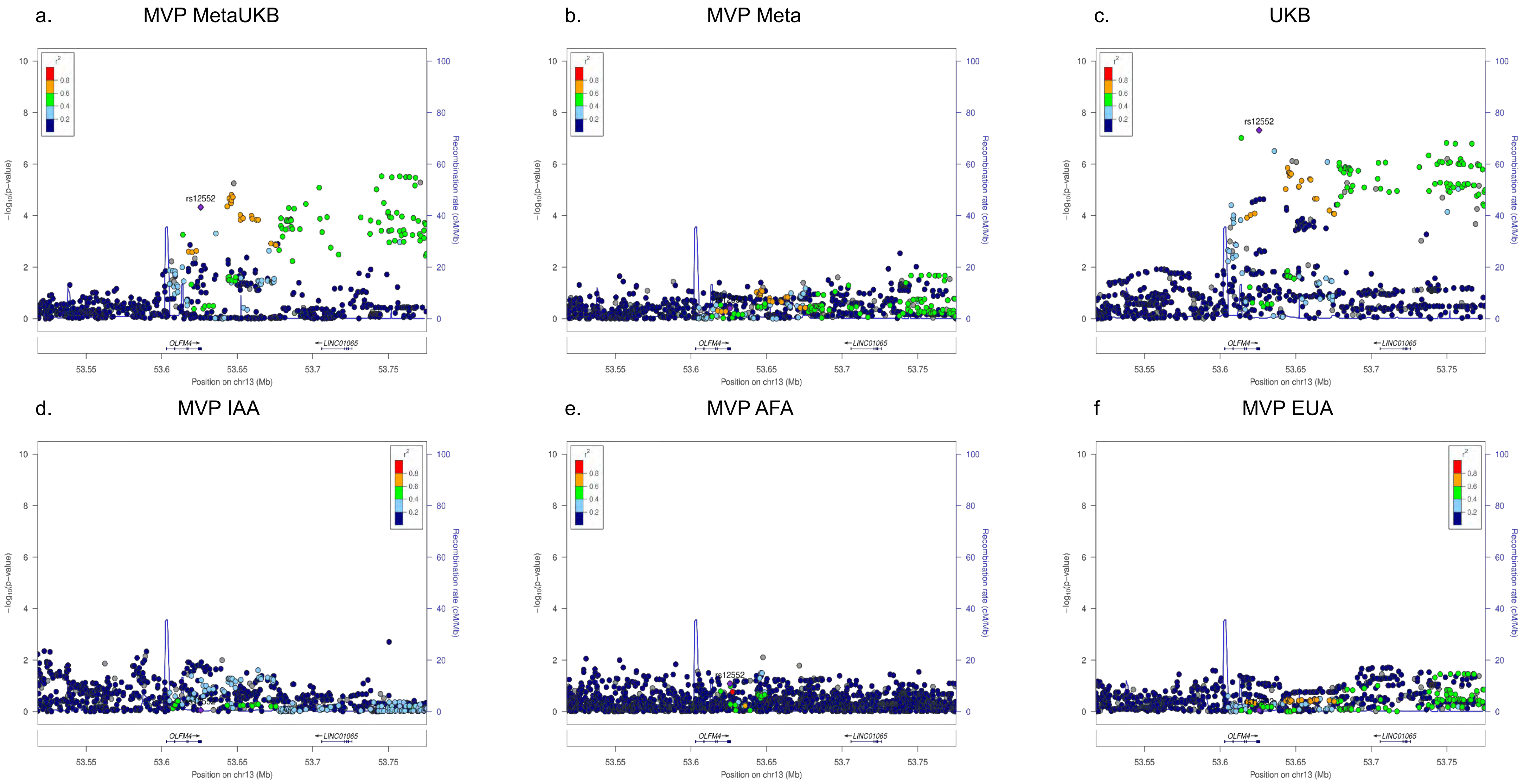

# locus091 | rs7329659

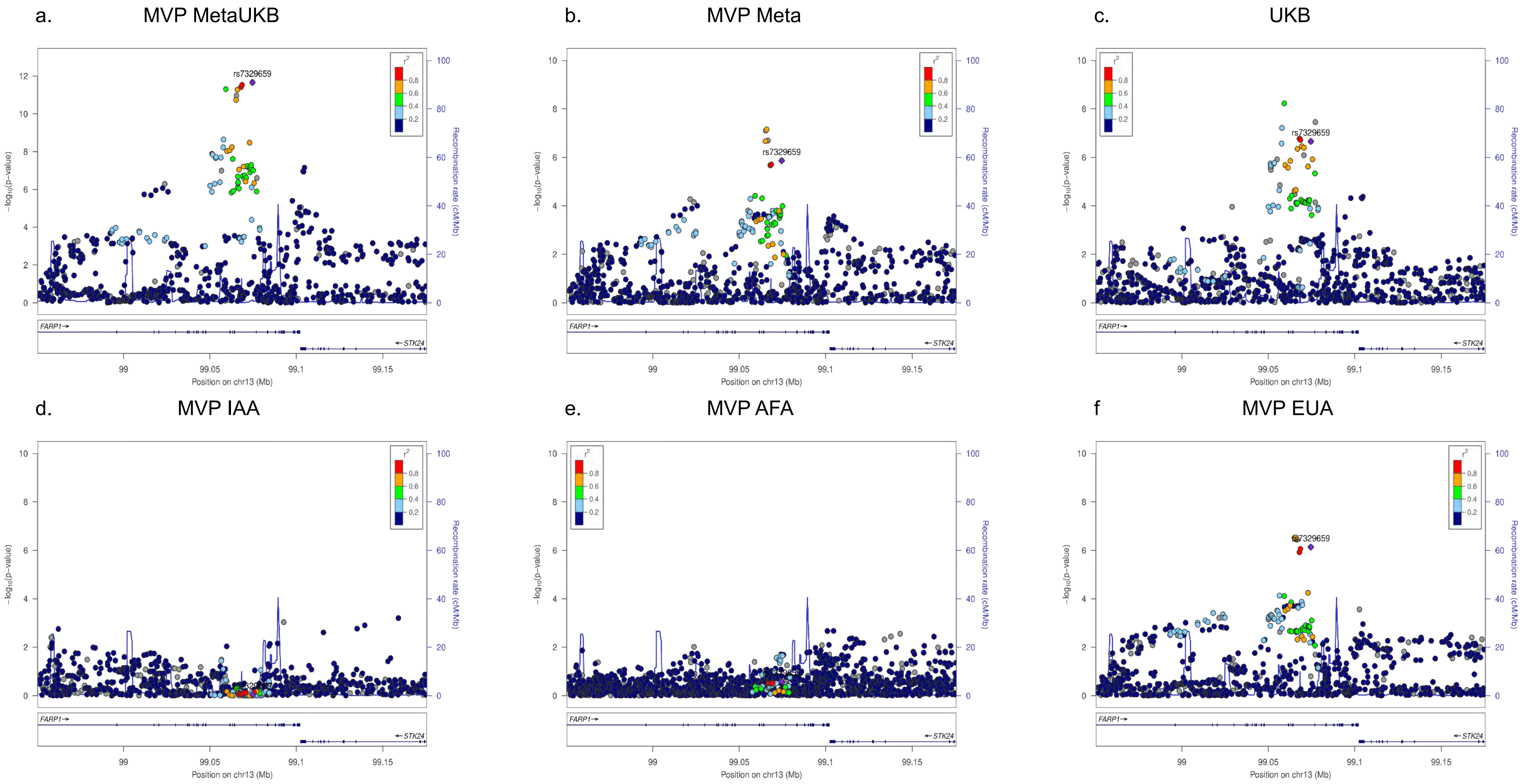

locus091 | rs9517282

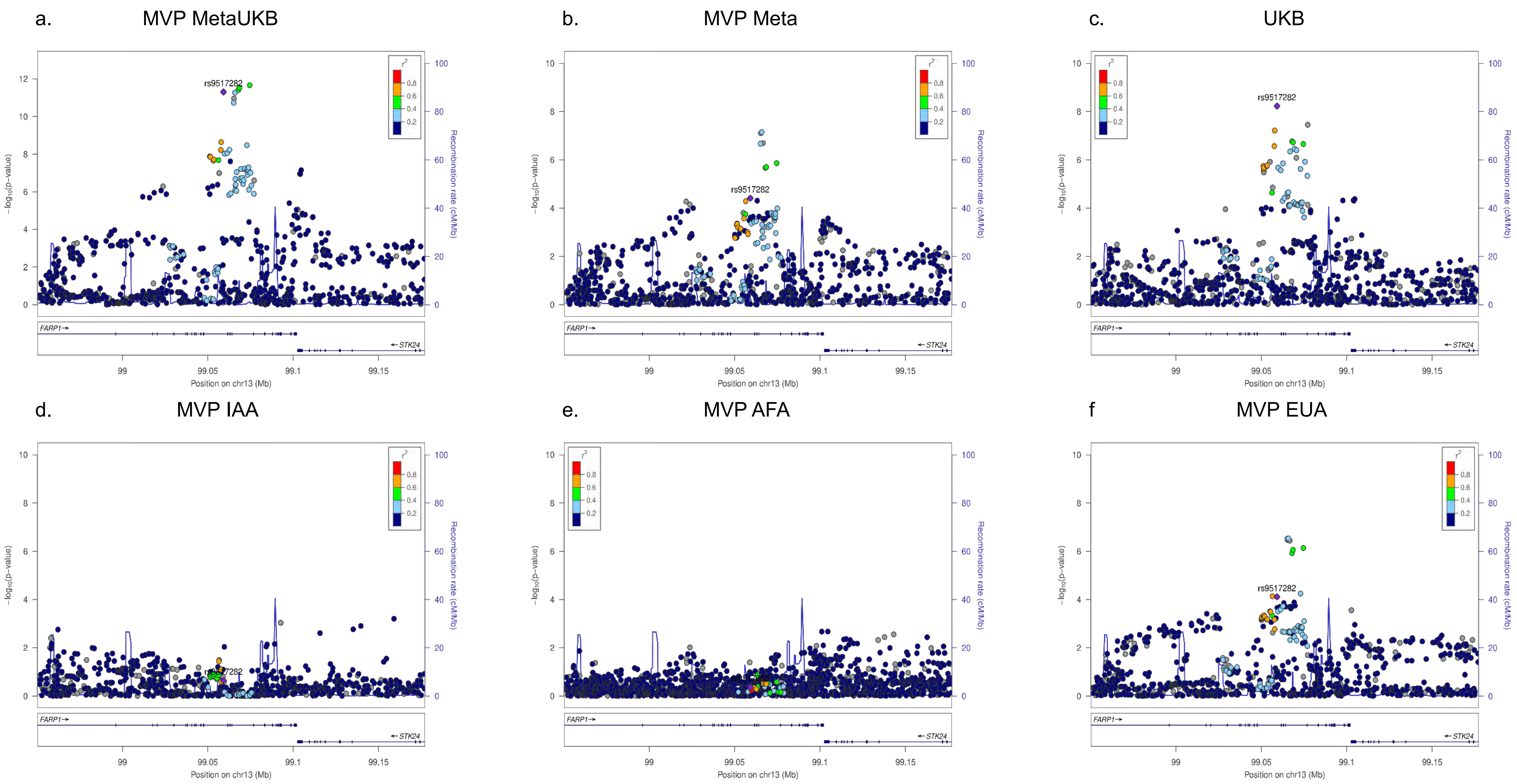

# locus092 | rs1566129

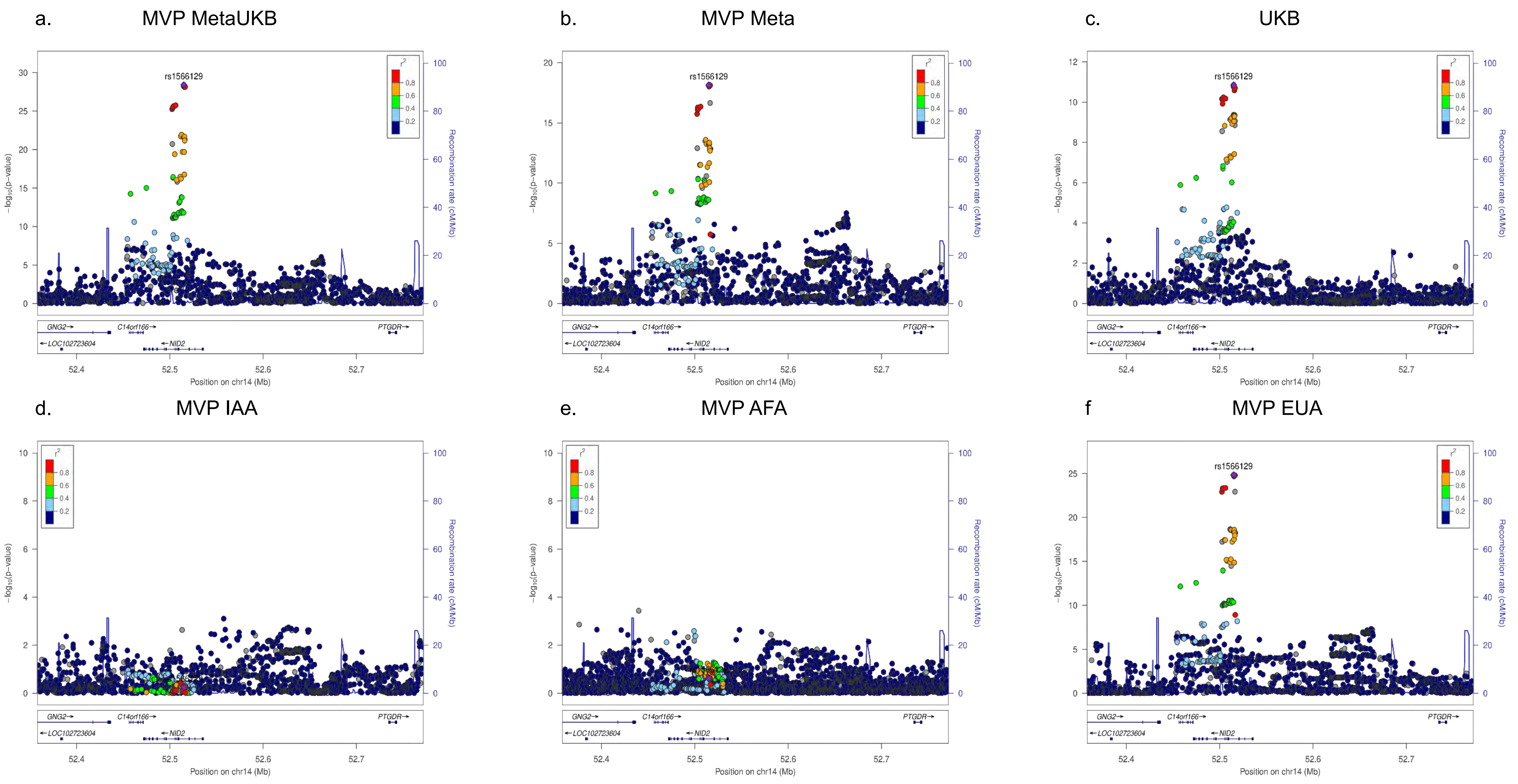

# locus092 | rs2749883

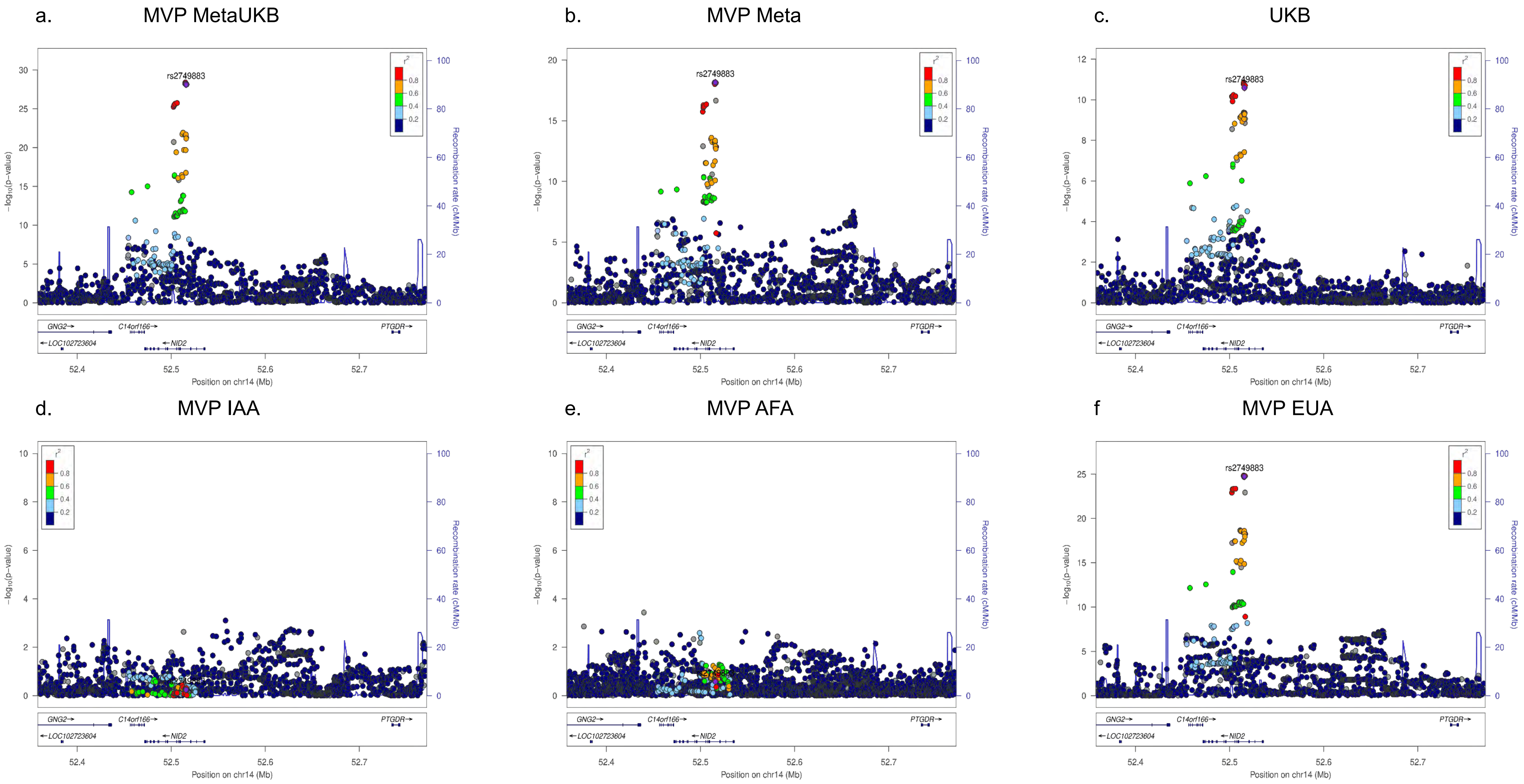

# locus093 | rs229650

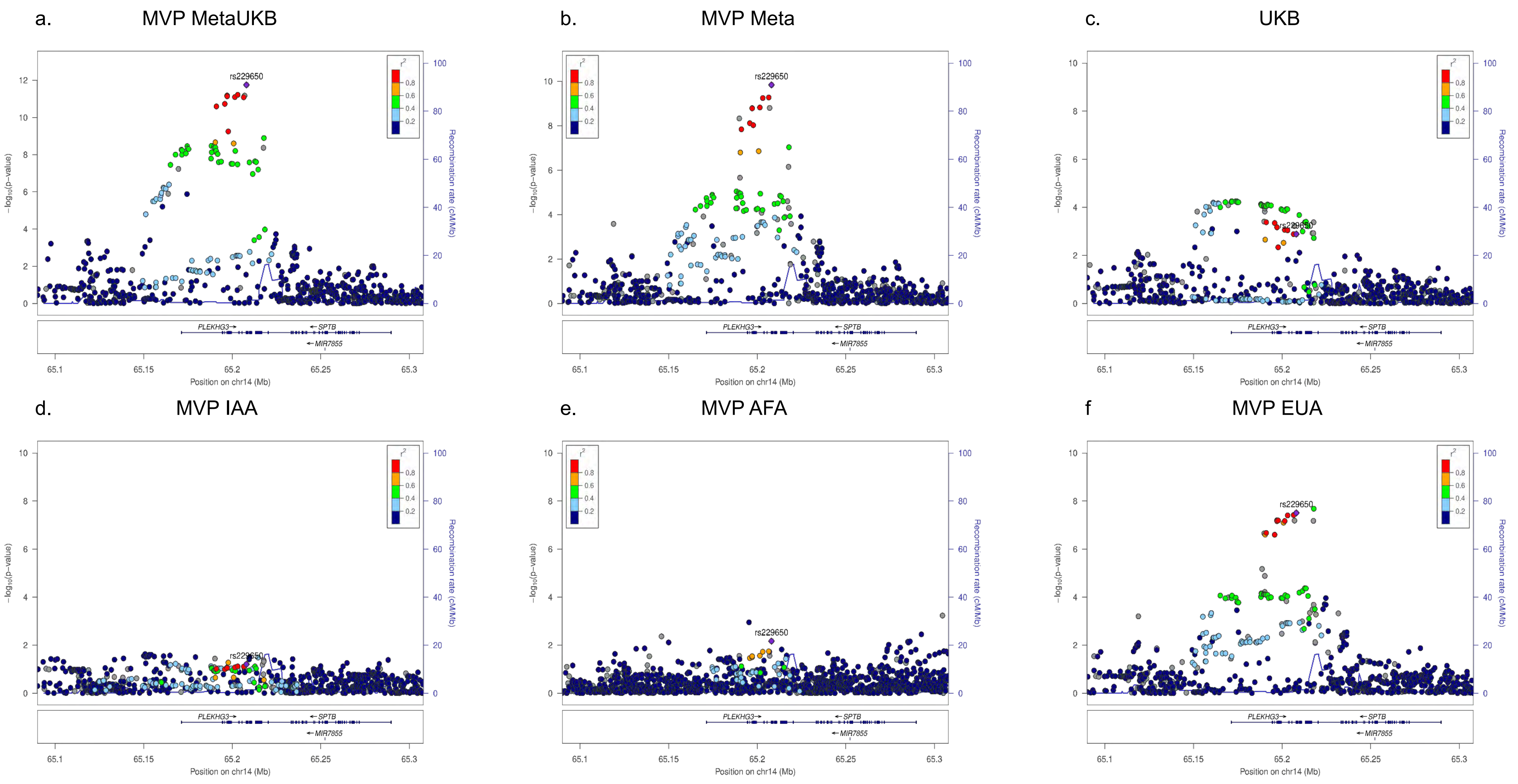

# locus093 | rs2357777

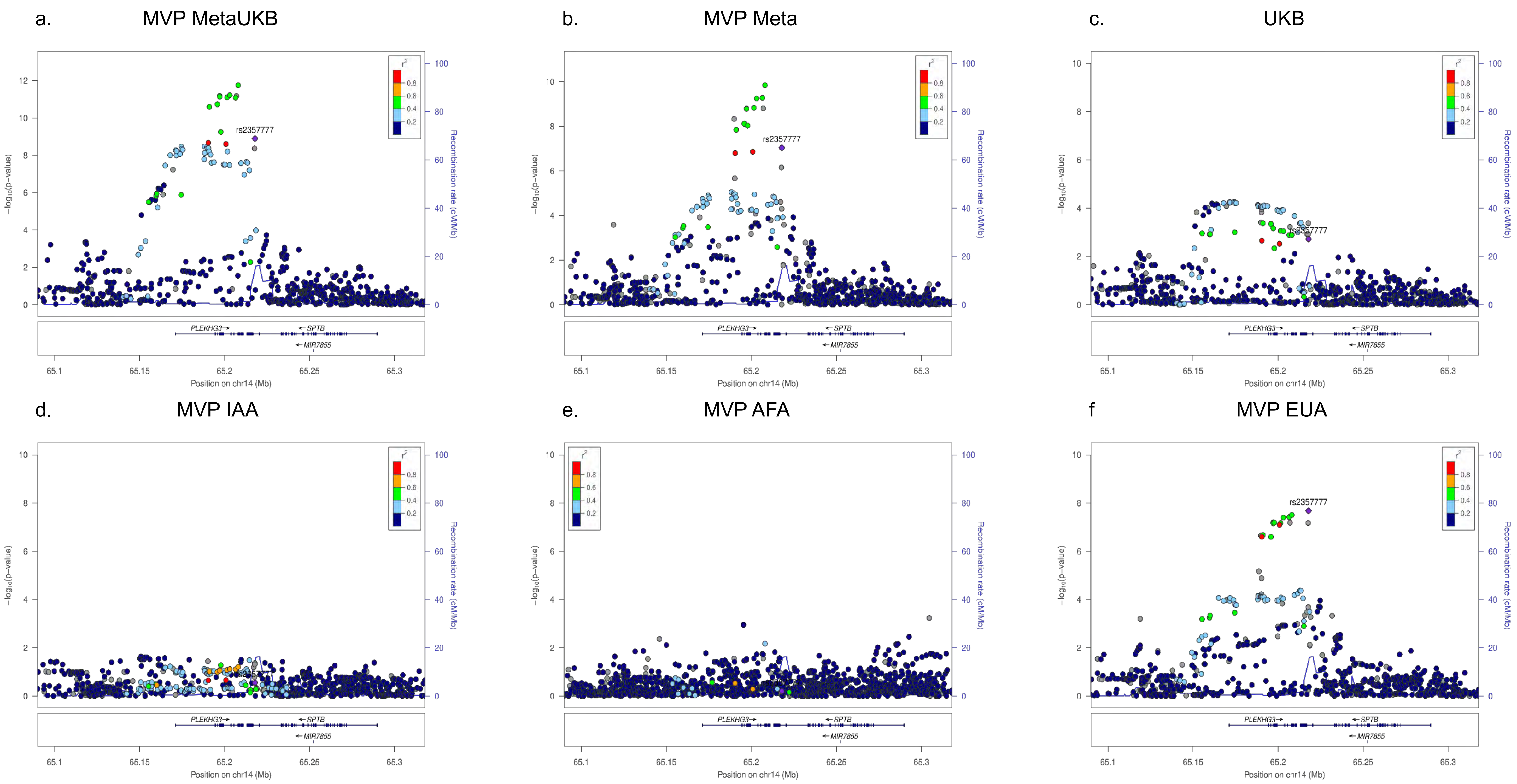

# locus094 | rs35417585

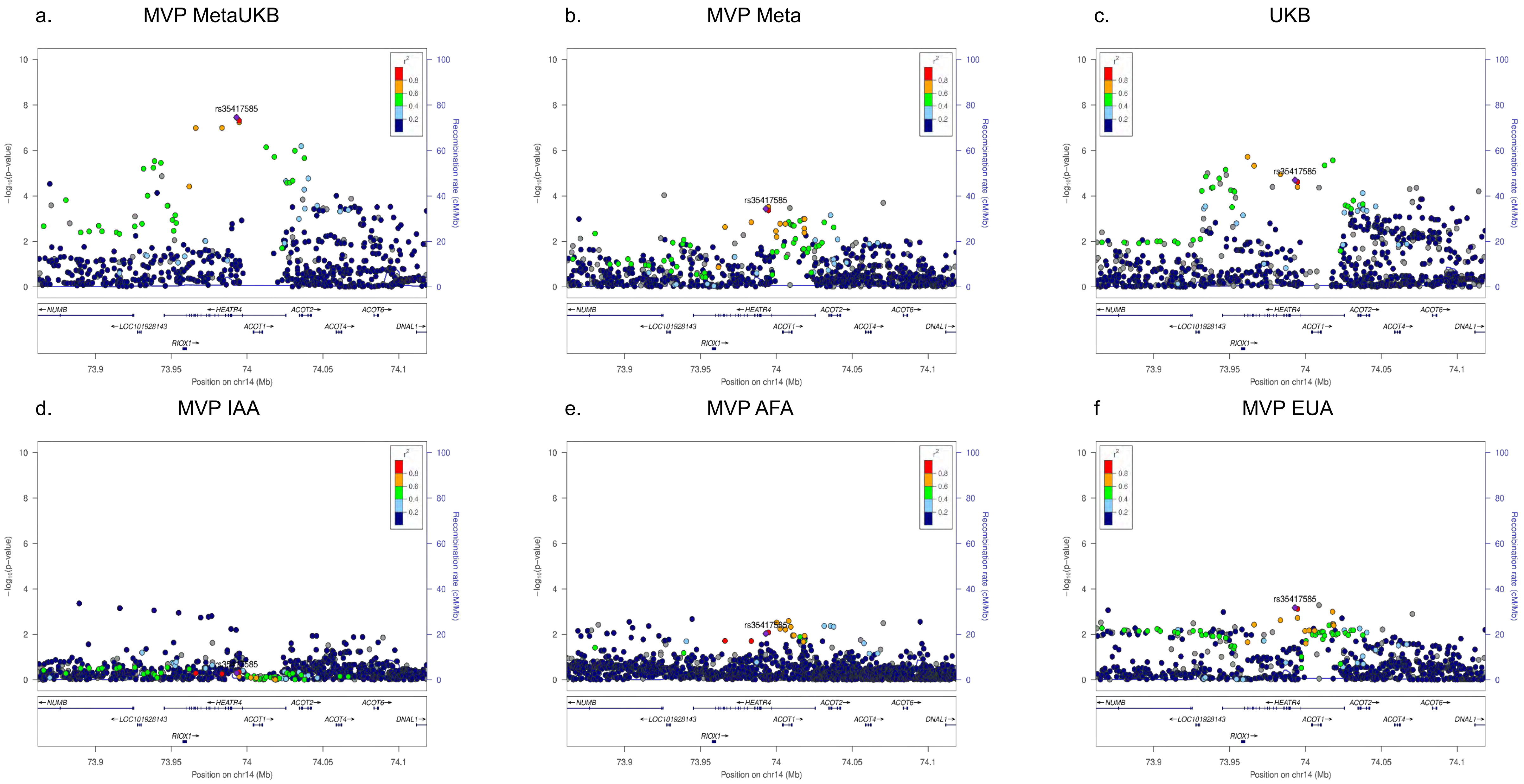

# locus095 | rs35499335

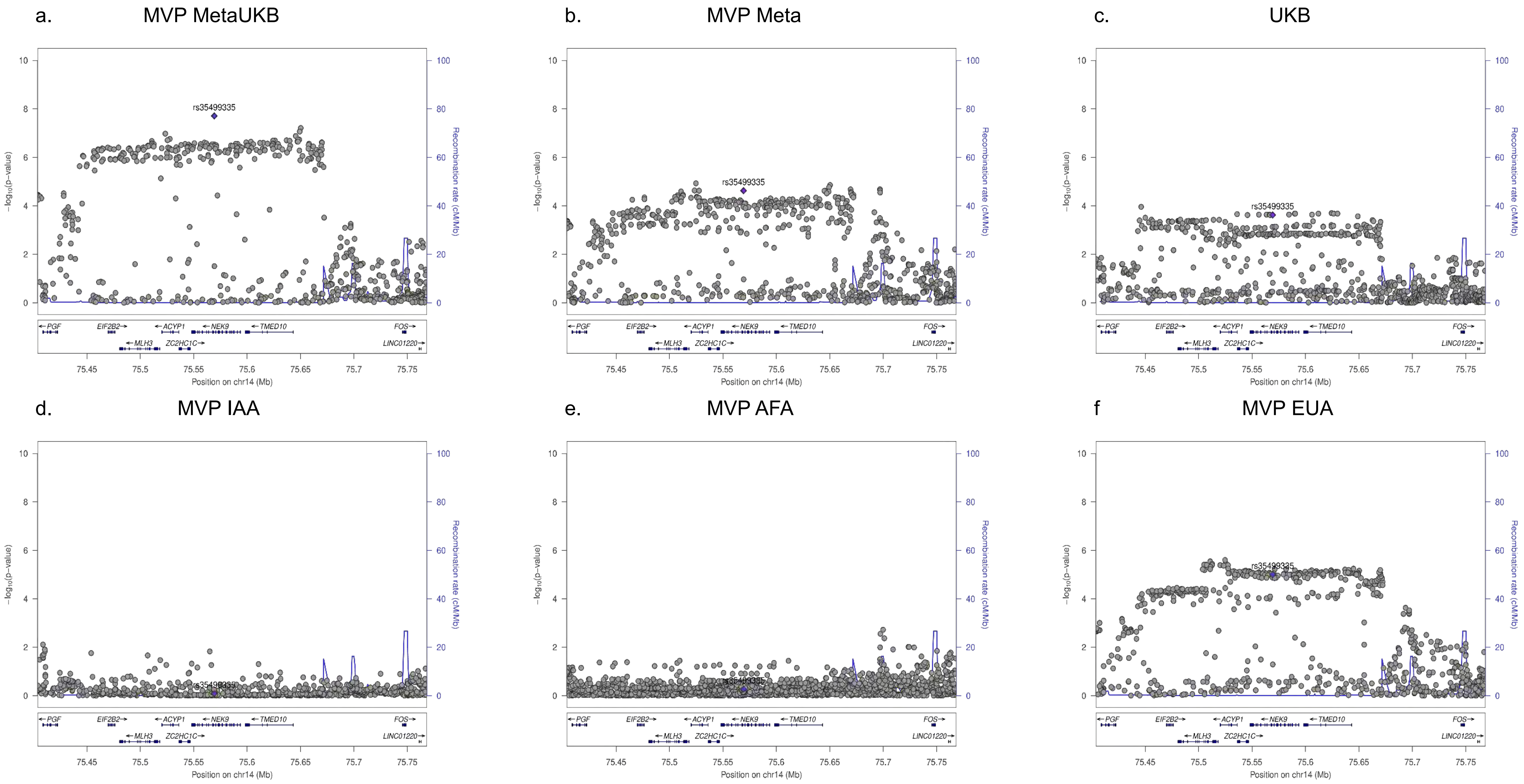

# locus096 | rs12588043

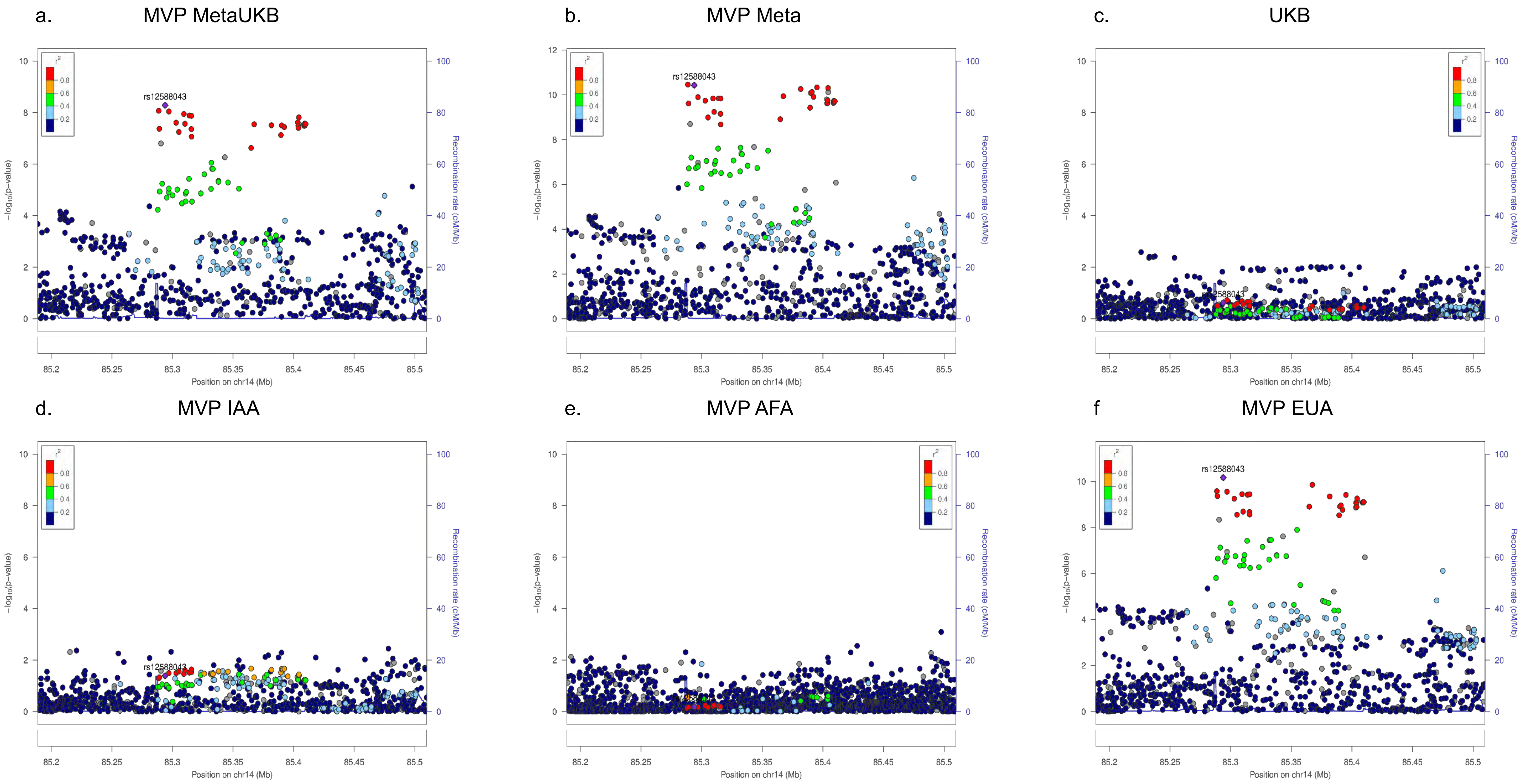

# locus096 | rs1887072

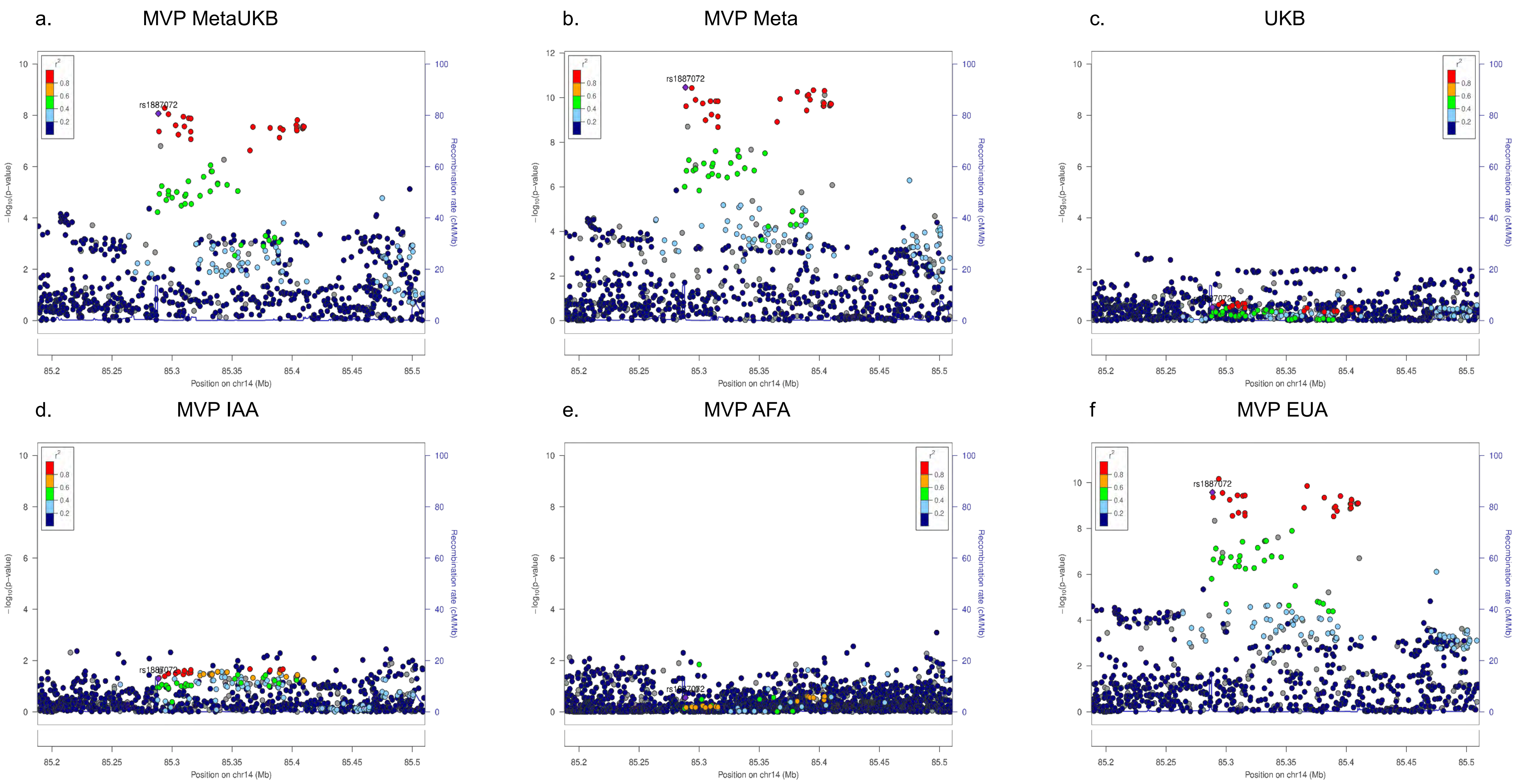

# locus097 |  rs1951486

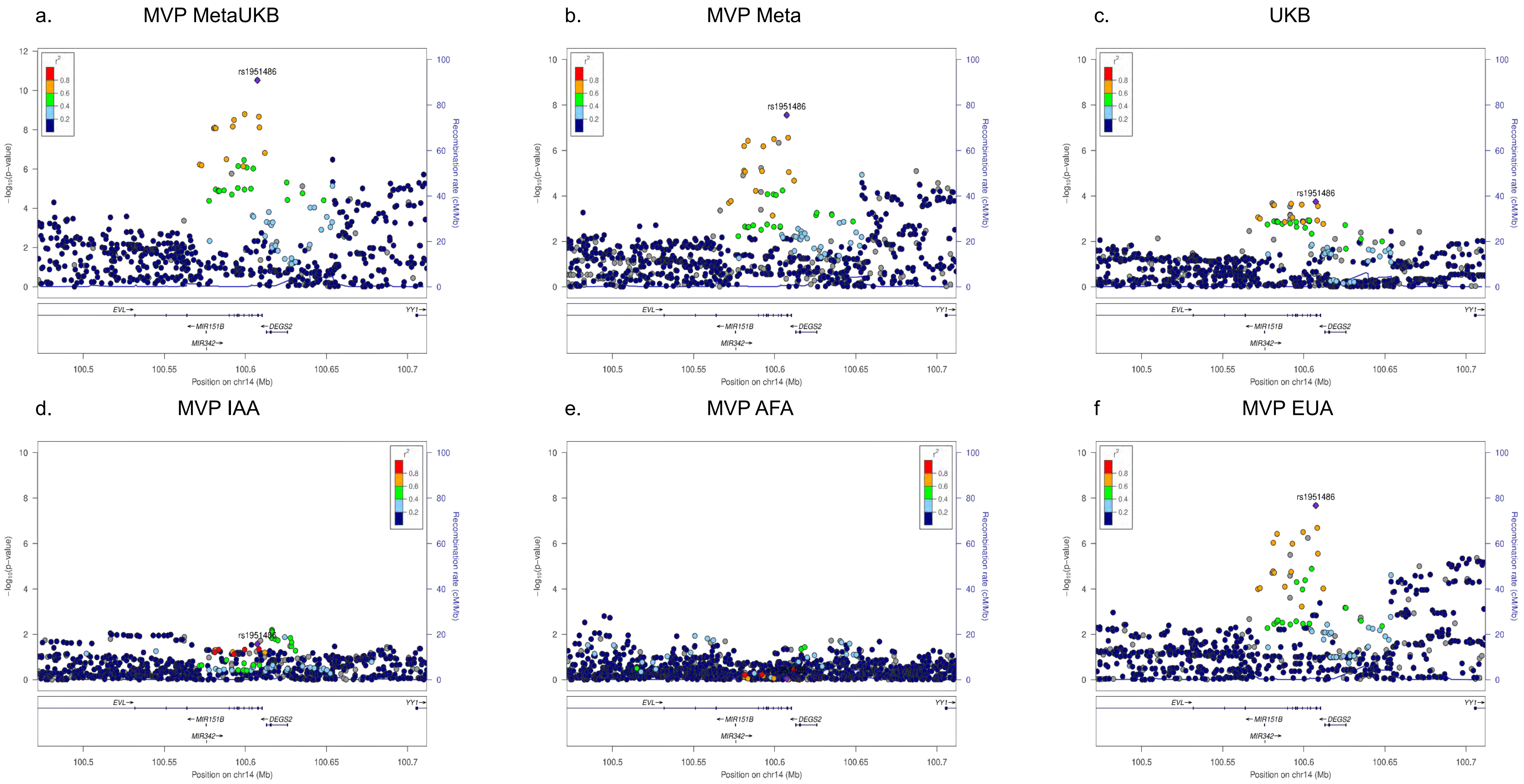

# locus098 | rs62015206

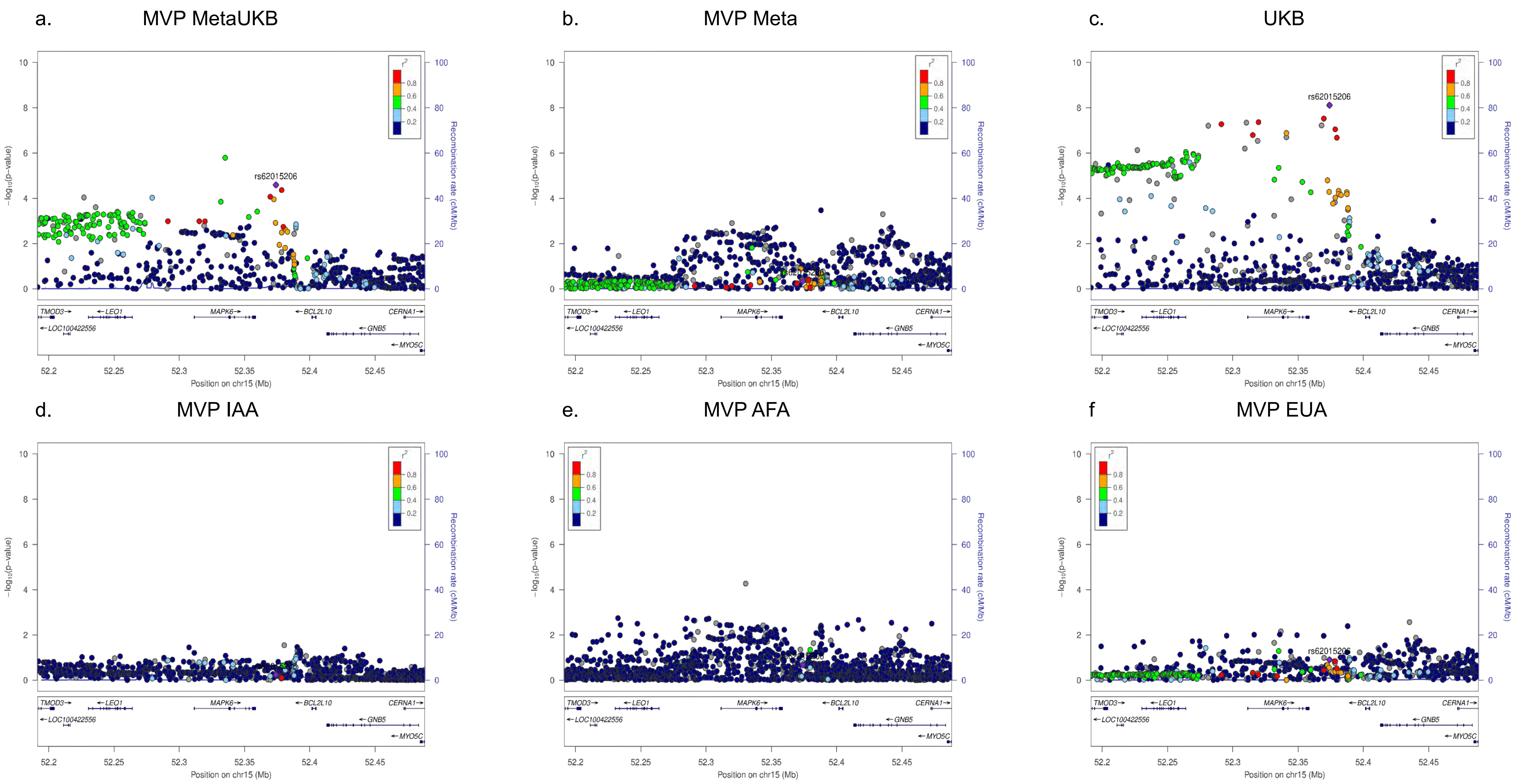

# locus099 | rs59425097

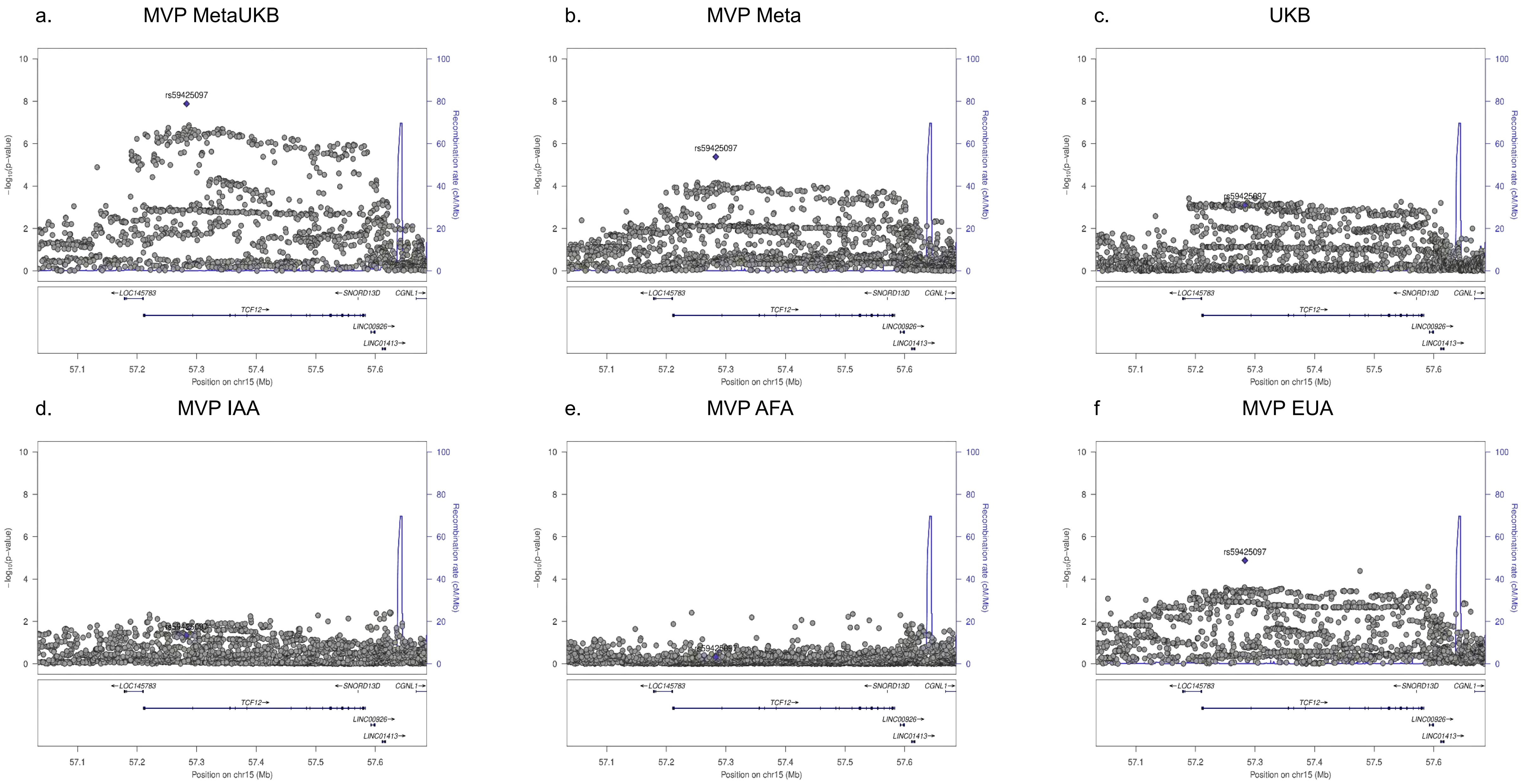

locus100 | rs11858525

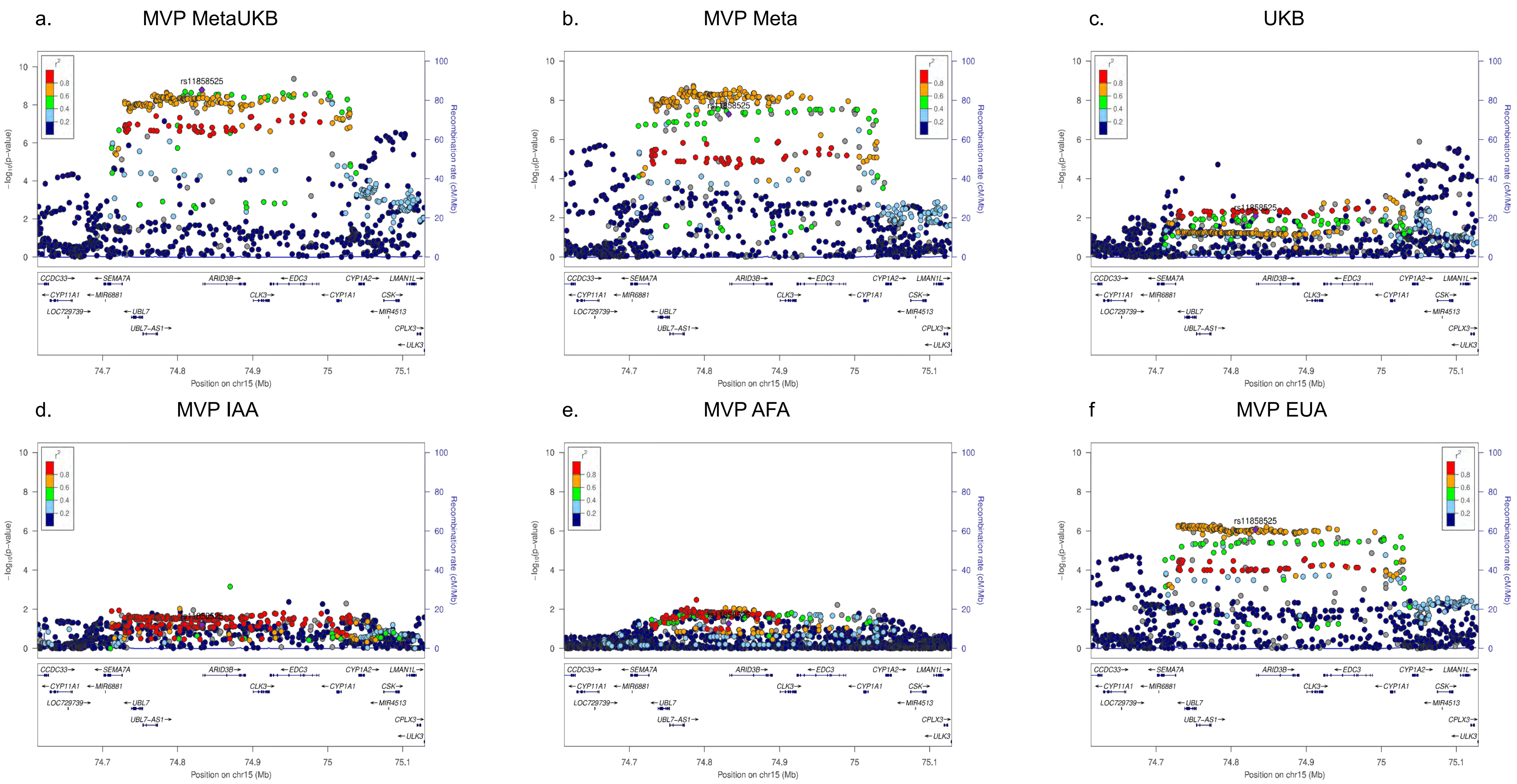

# locus100 | rs60777494

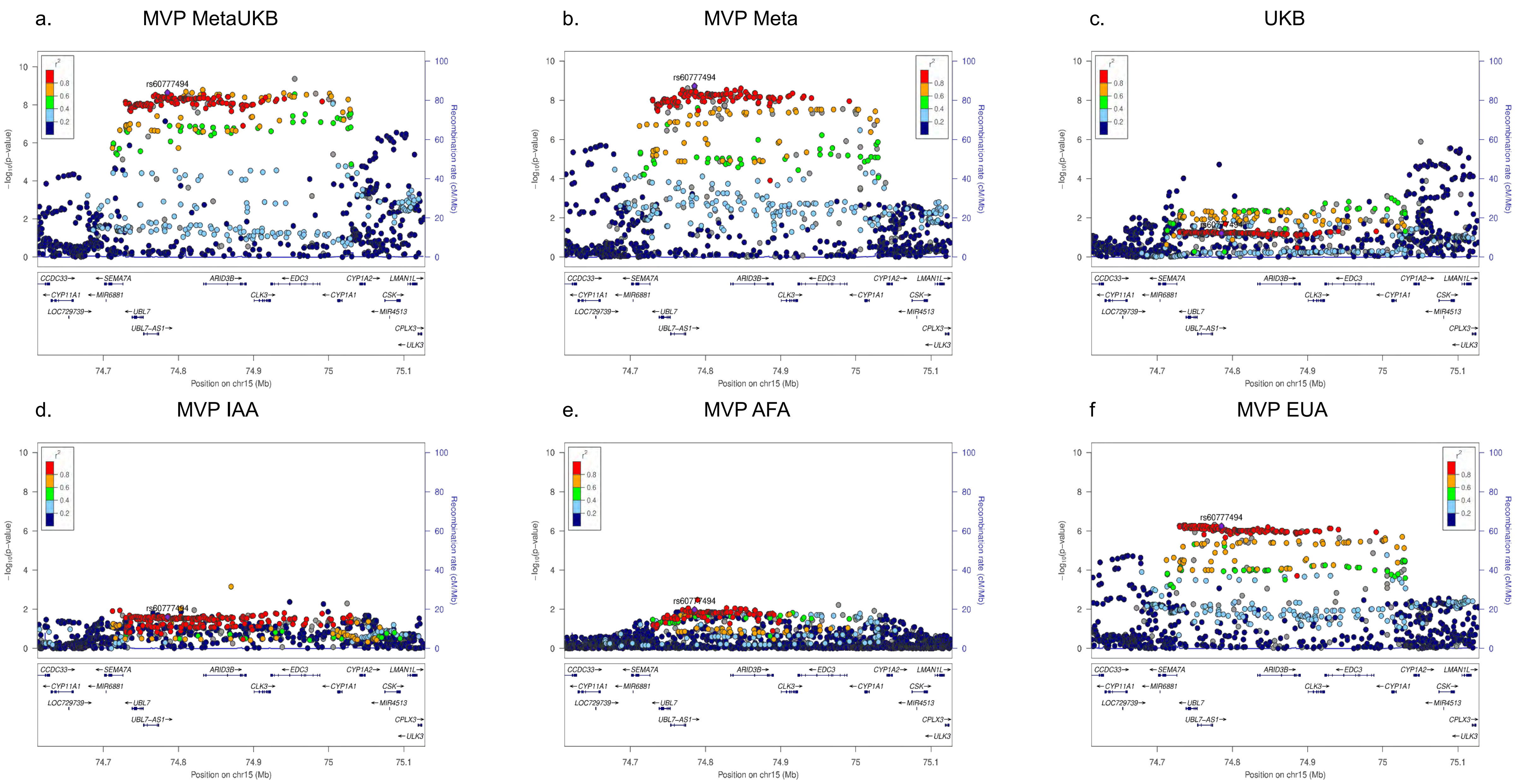

# locus101 | rs34593439

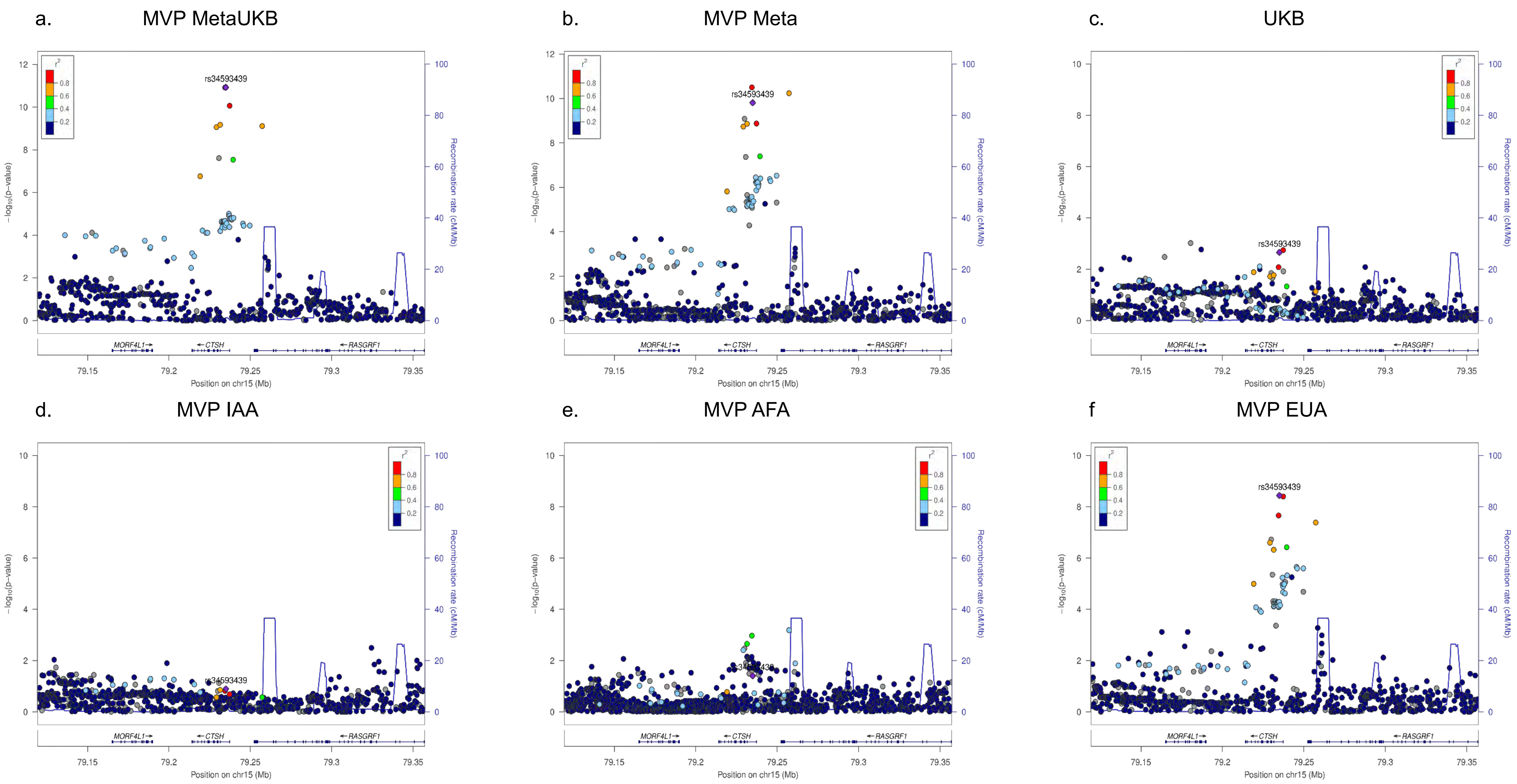

# locus101 | rs34843303

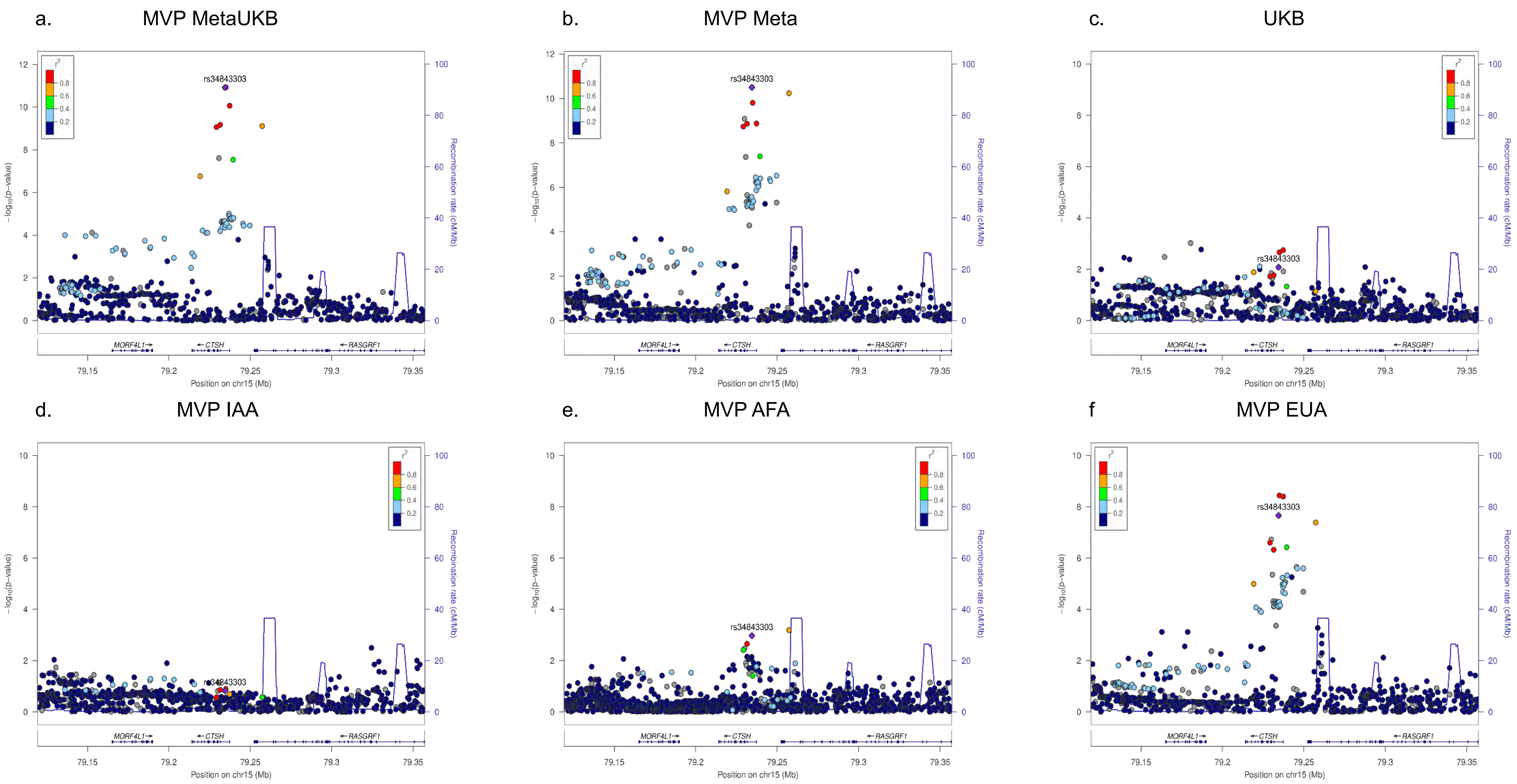

# locus102 | rs12441297

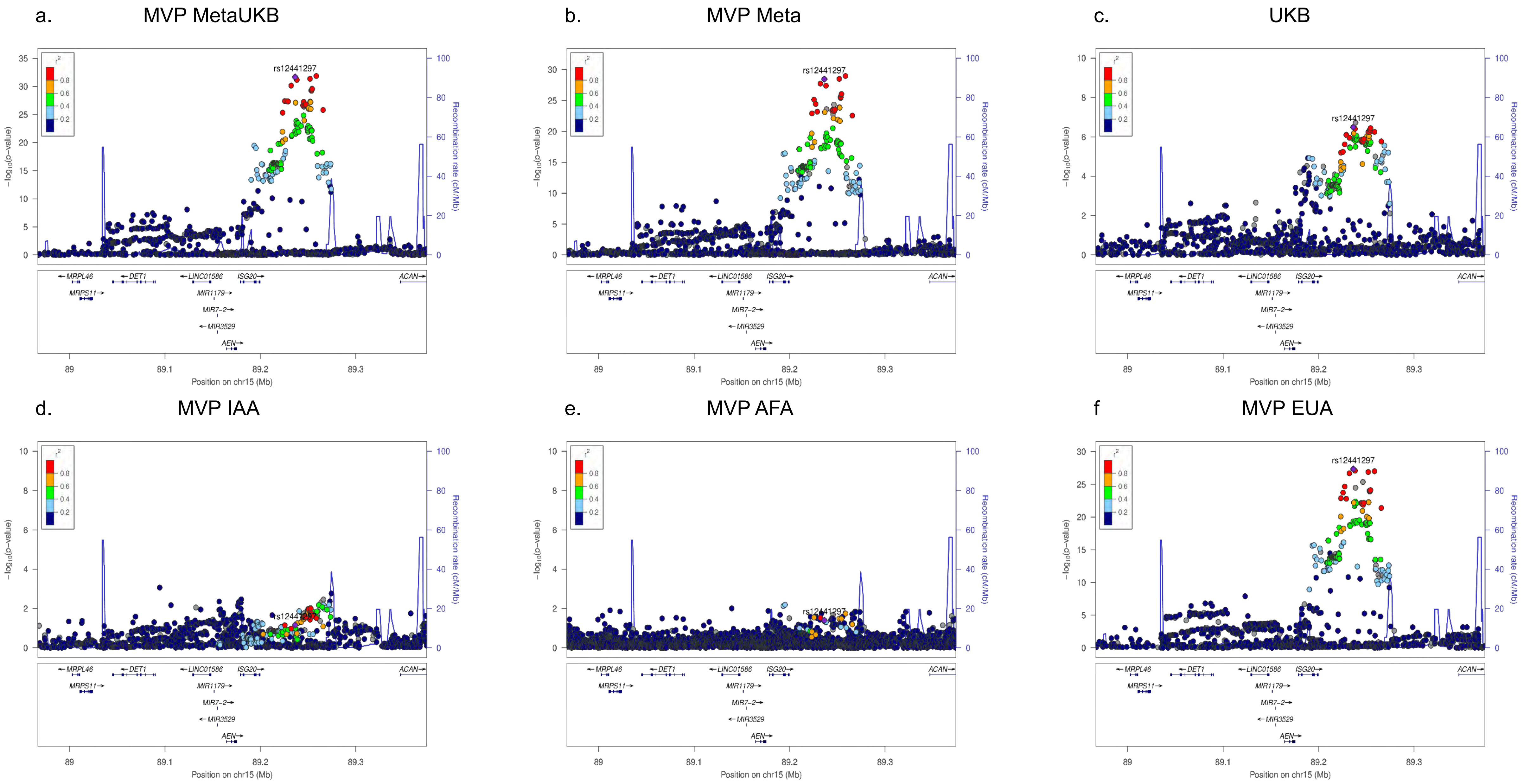

# locus102 | rs12594617

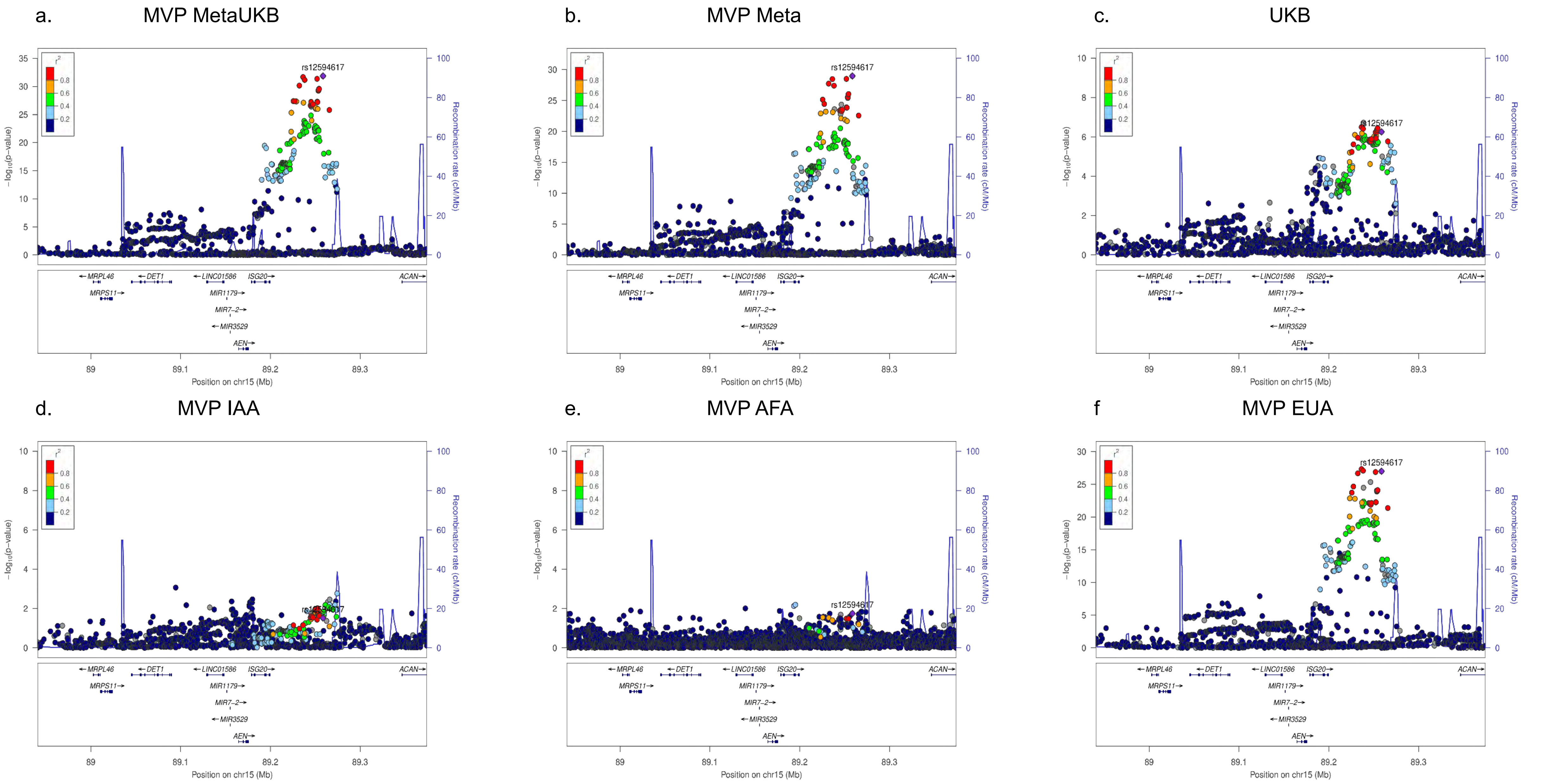

# locus103 | rs11643654

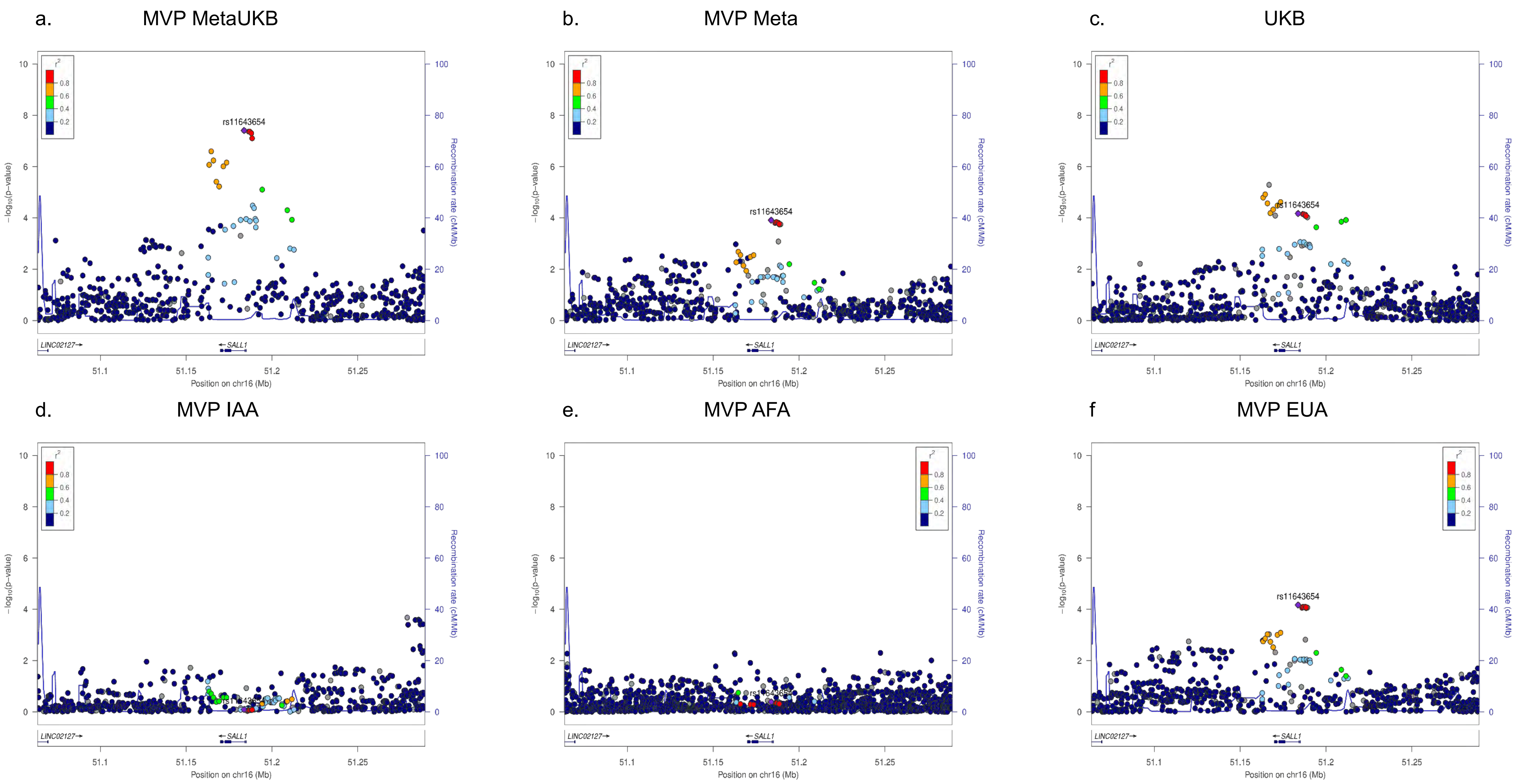

# locus104 | rs62033400

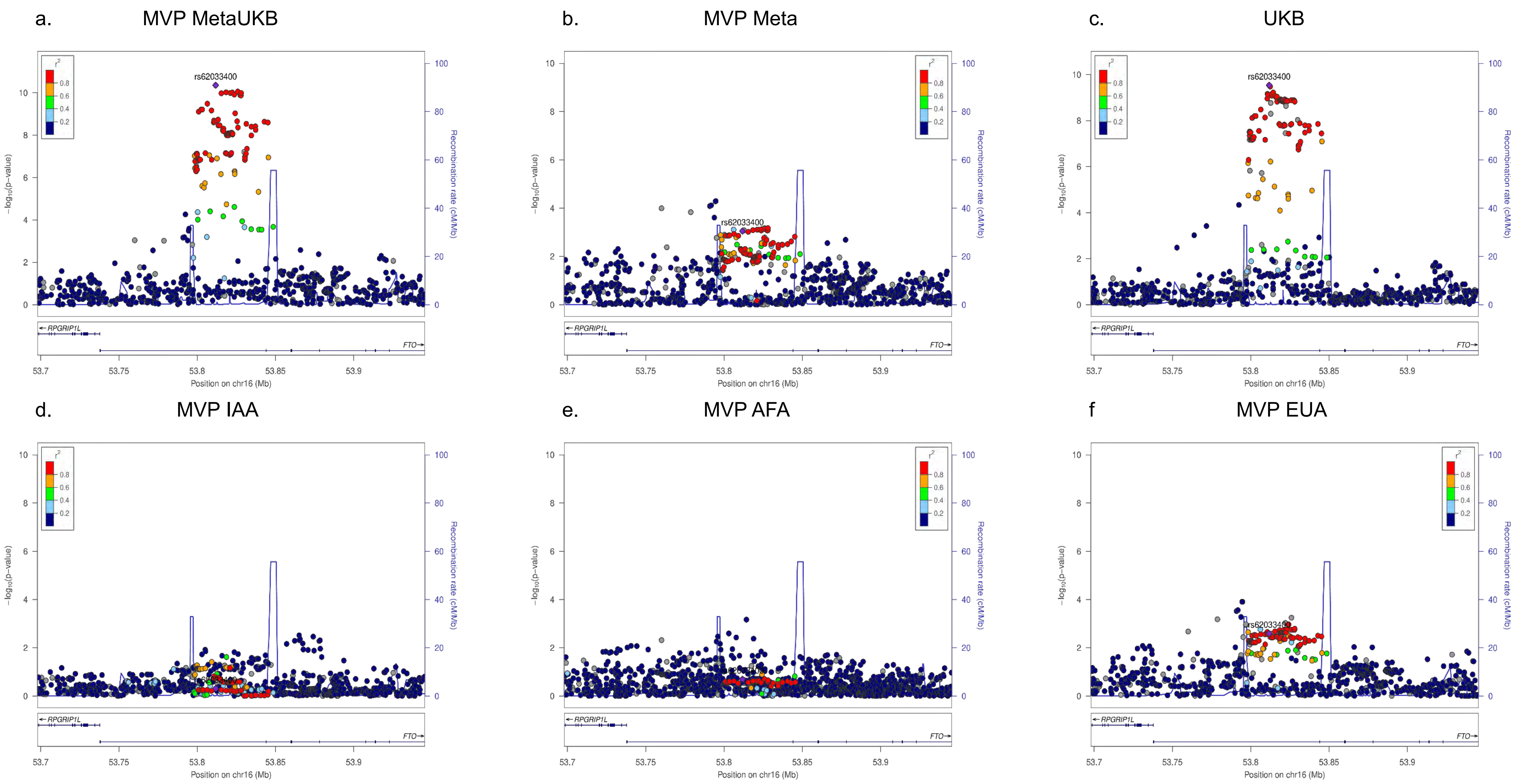

# locus105 | rs10492901

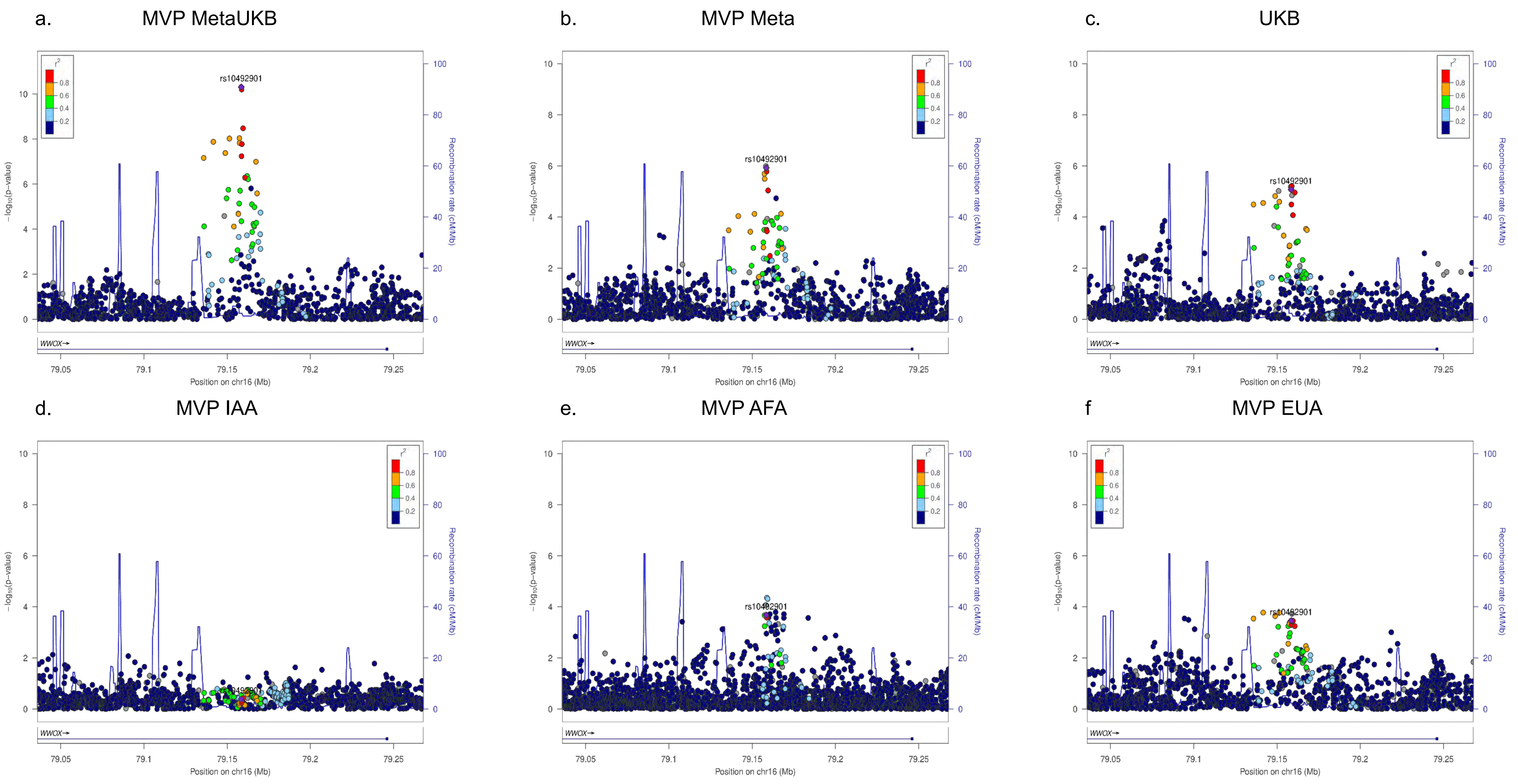

# locus106 | rs59164848

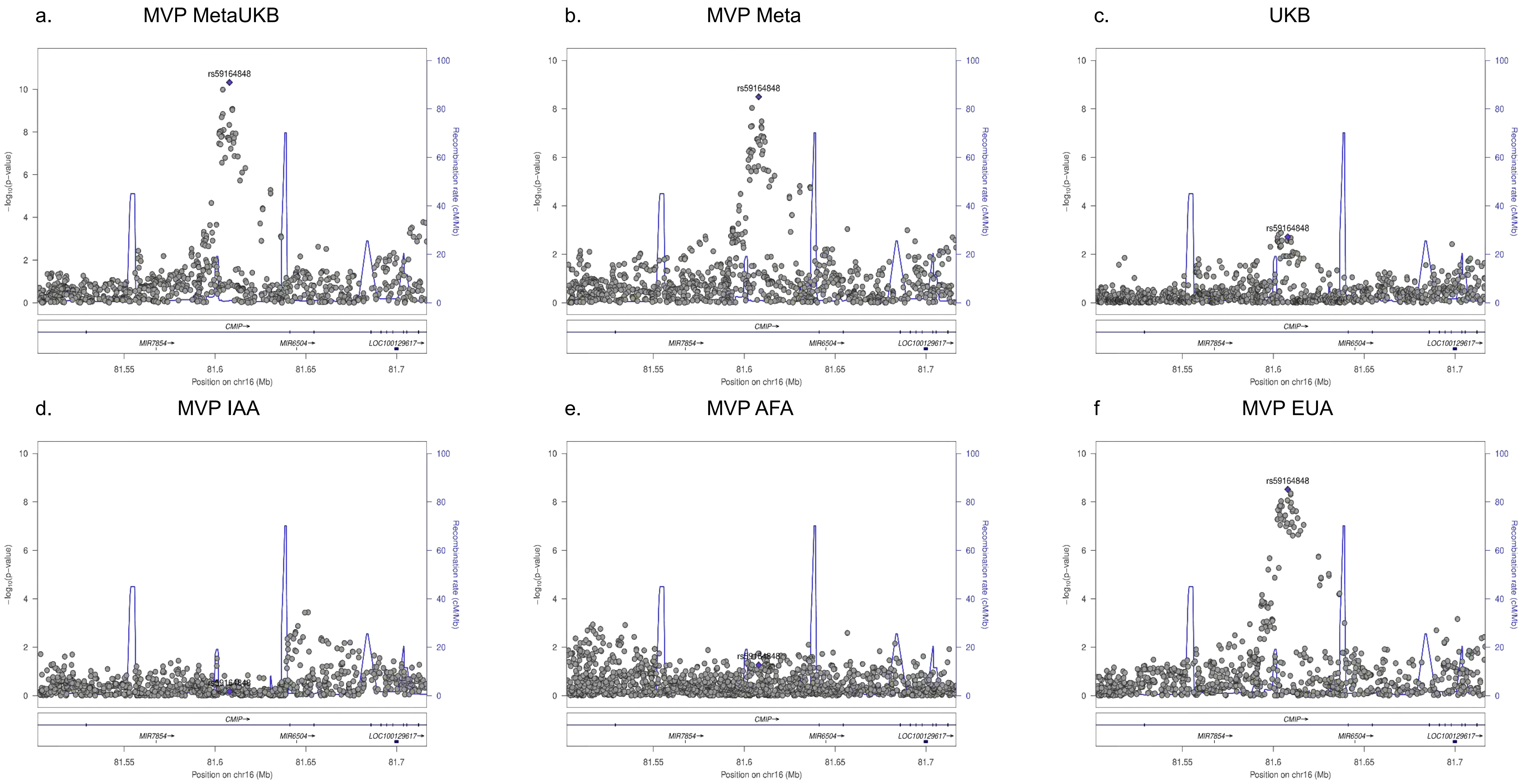

# locus107 | rs12938775

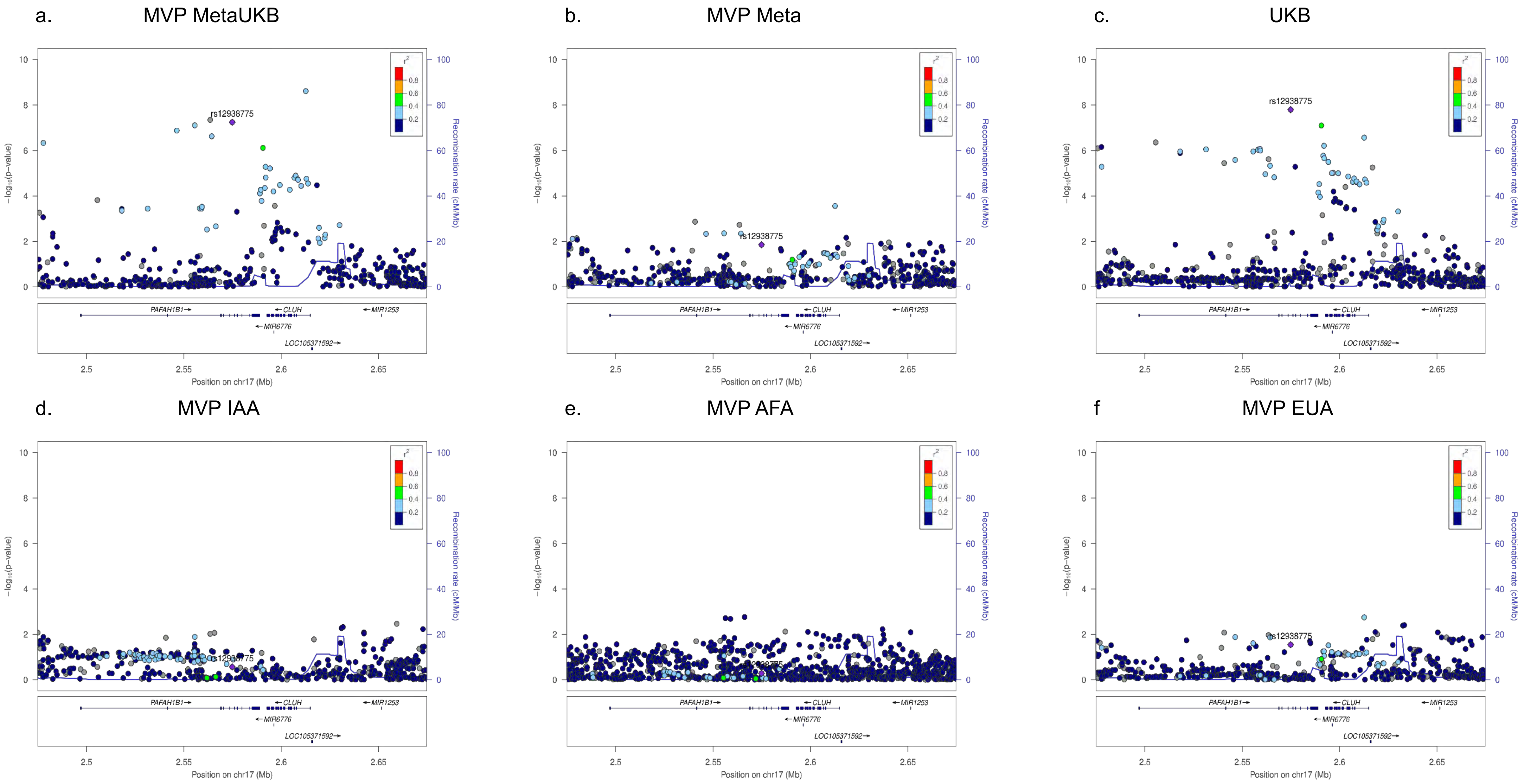

# locus107 | rs734957

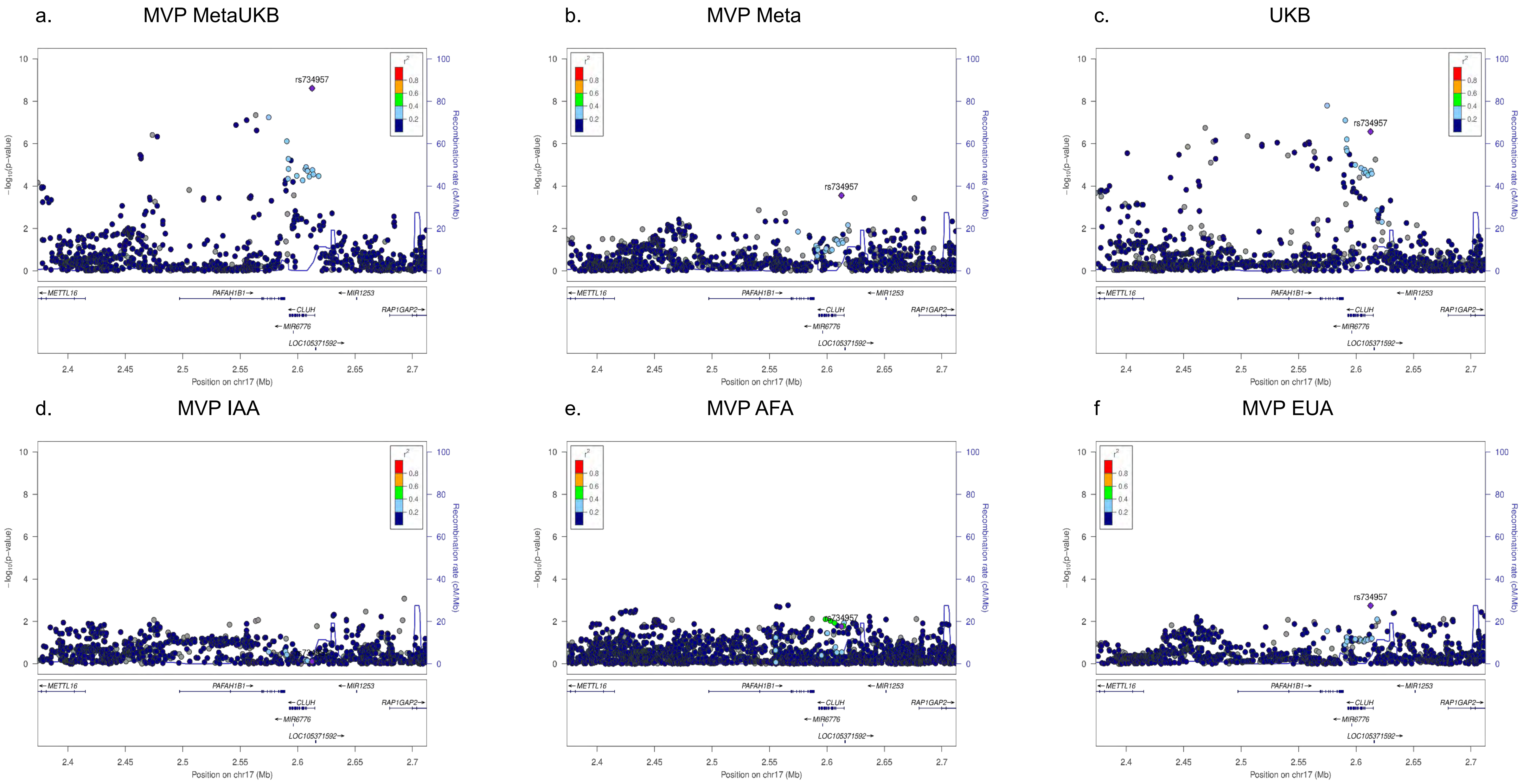

# locus108 | rs17671352

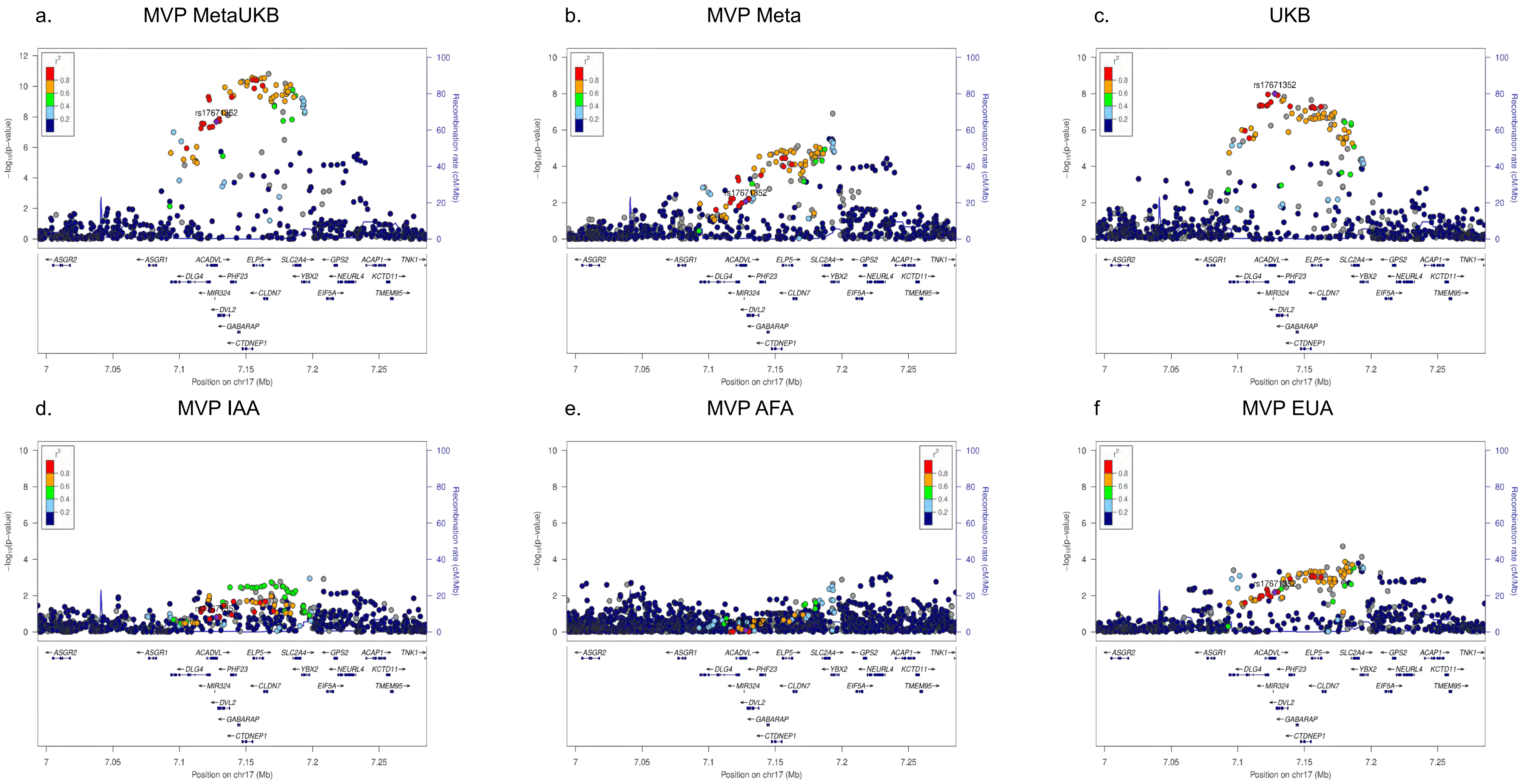

# locus108 | rs34958987

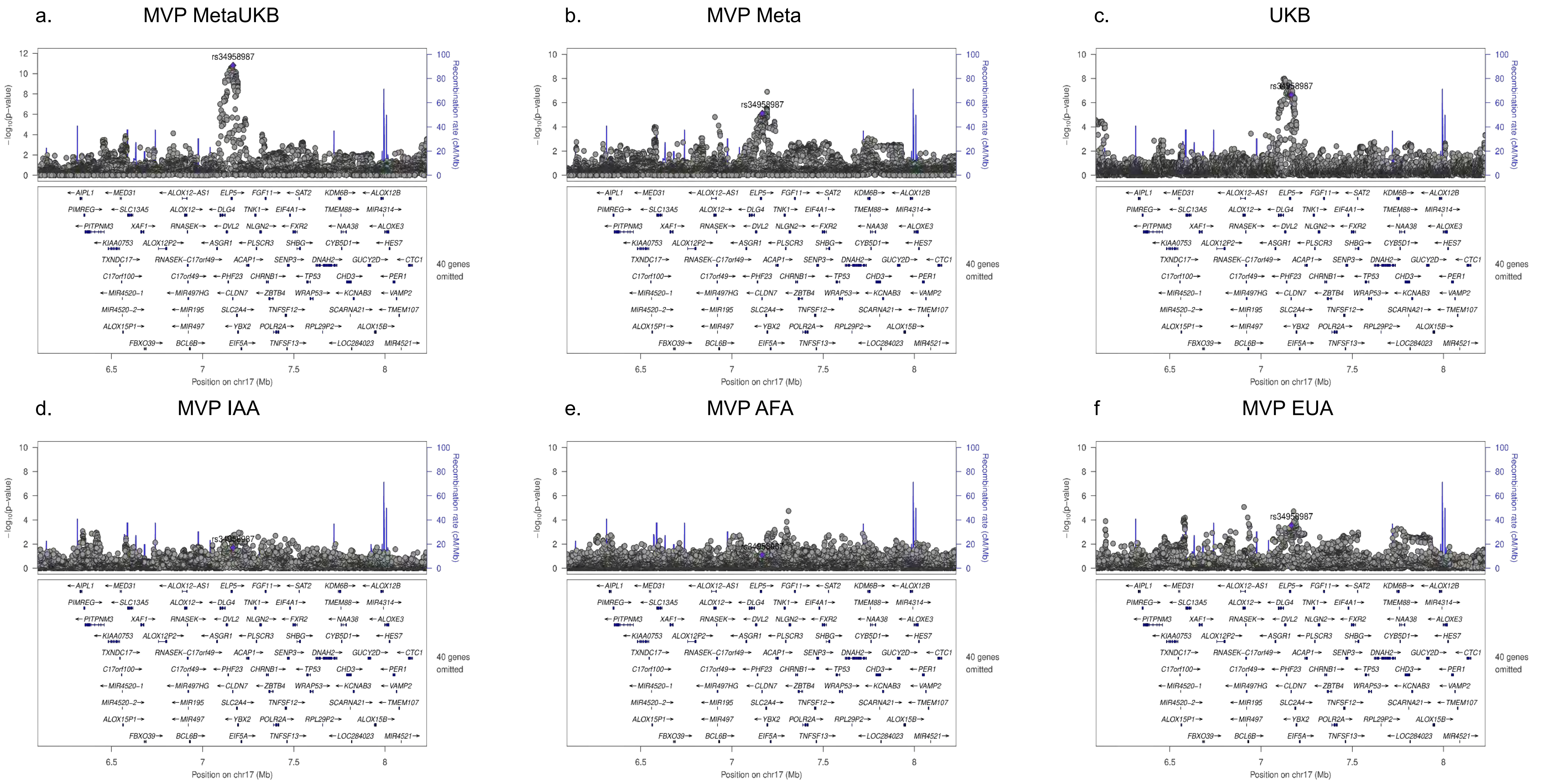

# locus109 | rs117283876

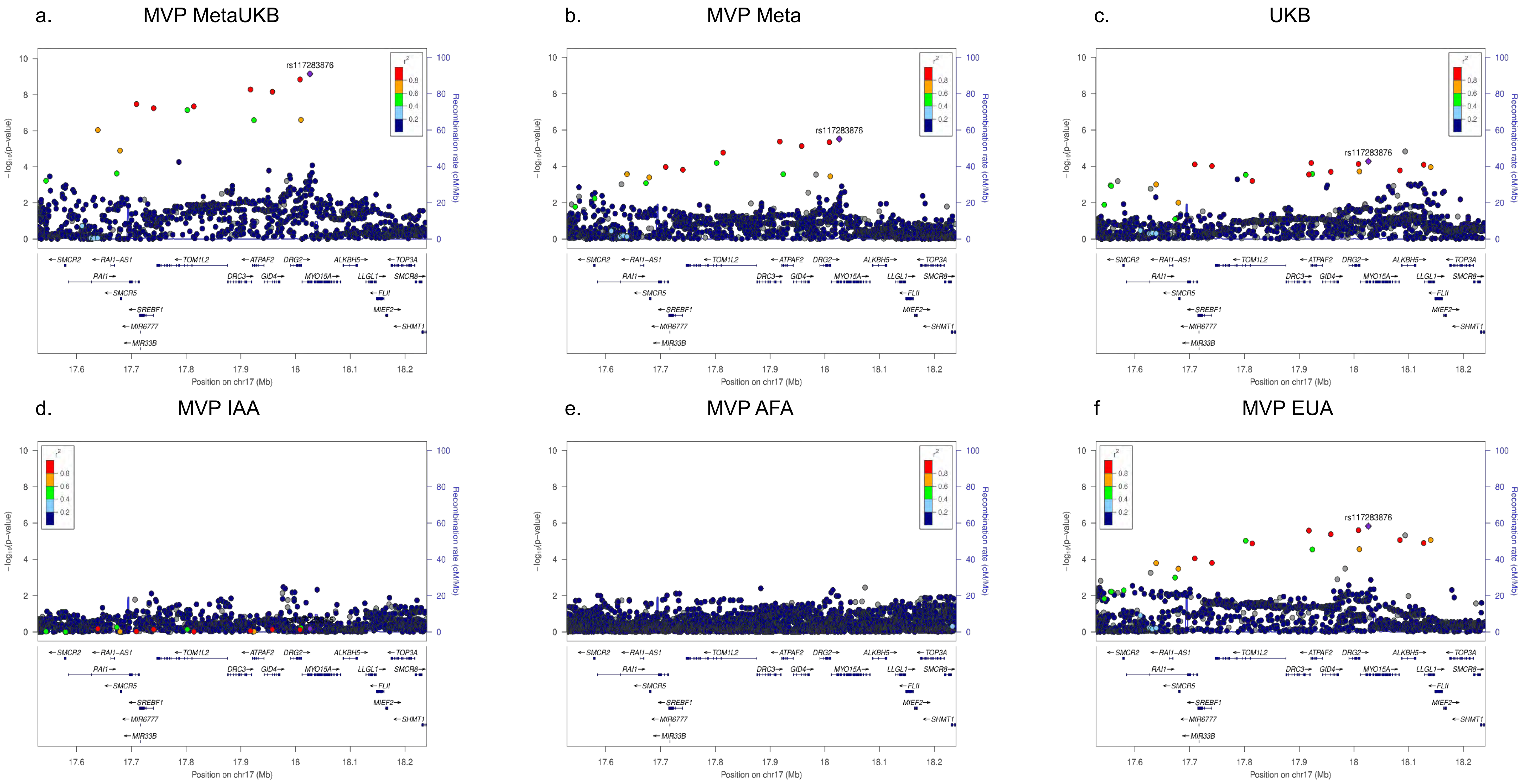

# locus110 | rs7405669

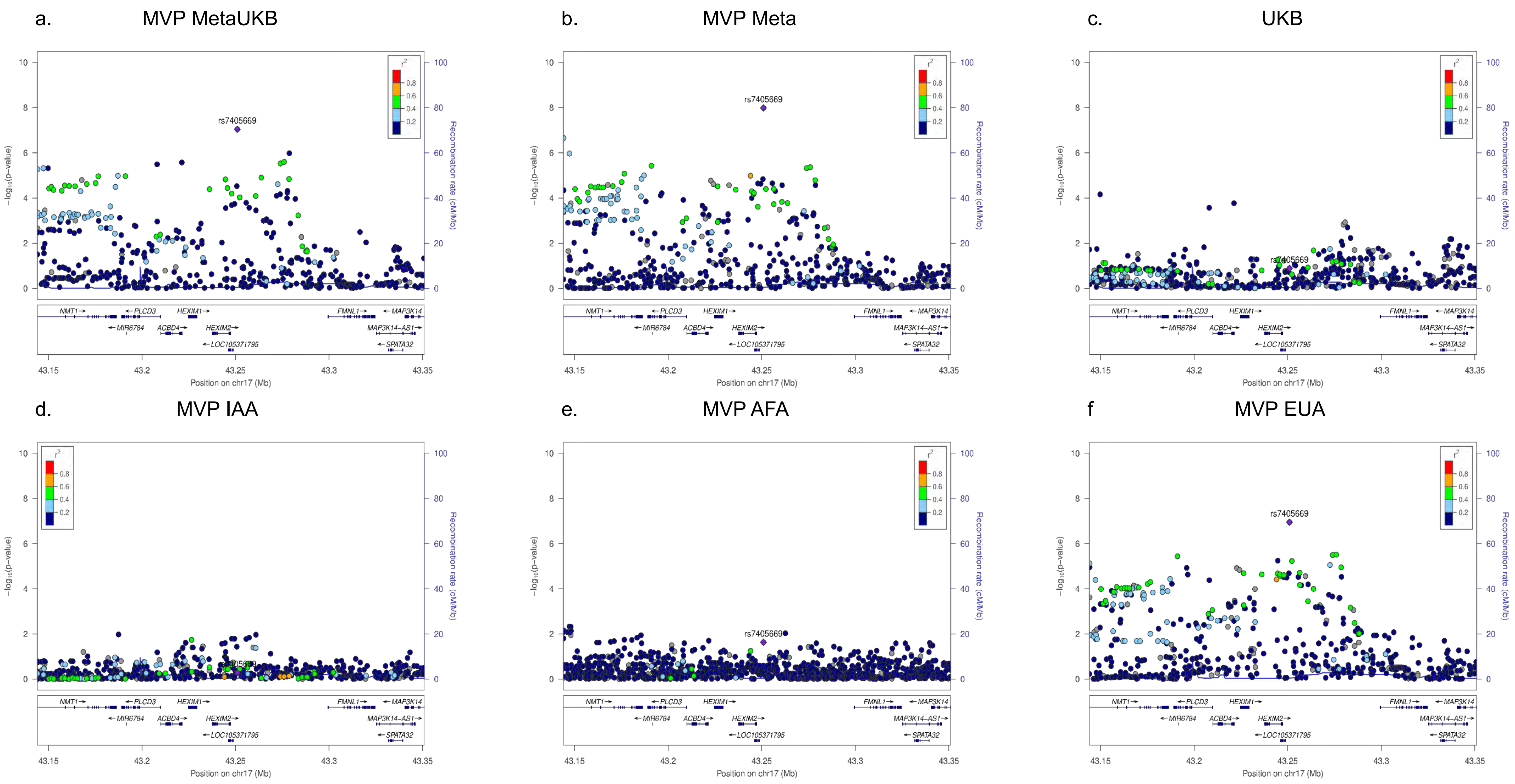

# locus111 | rs2458203

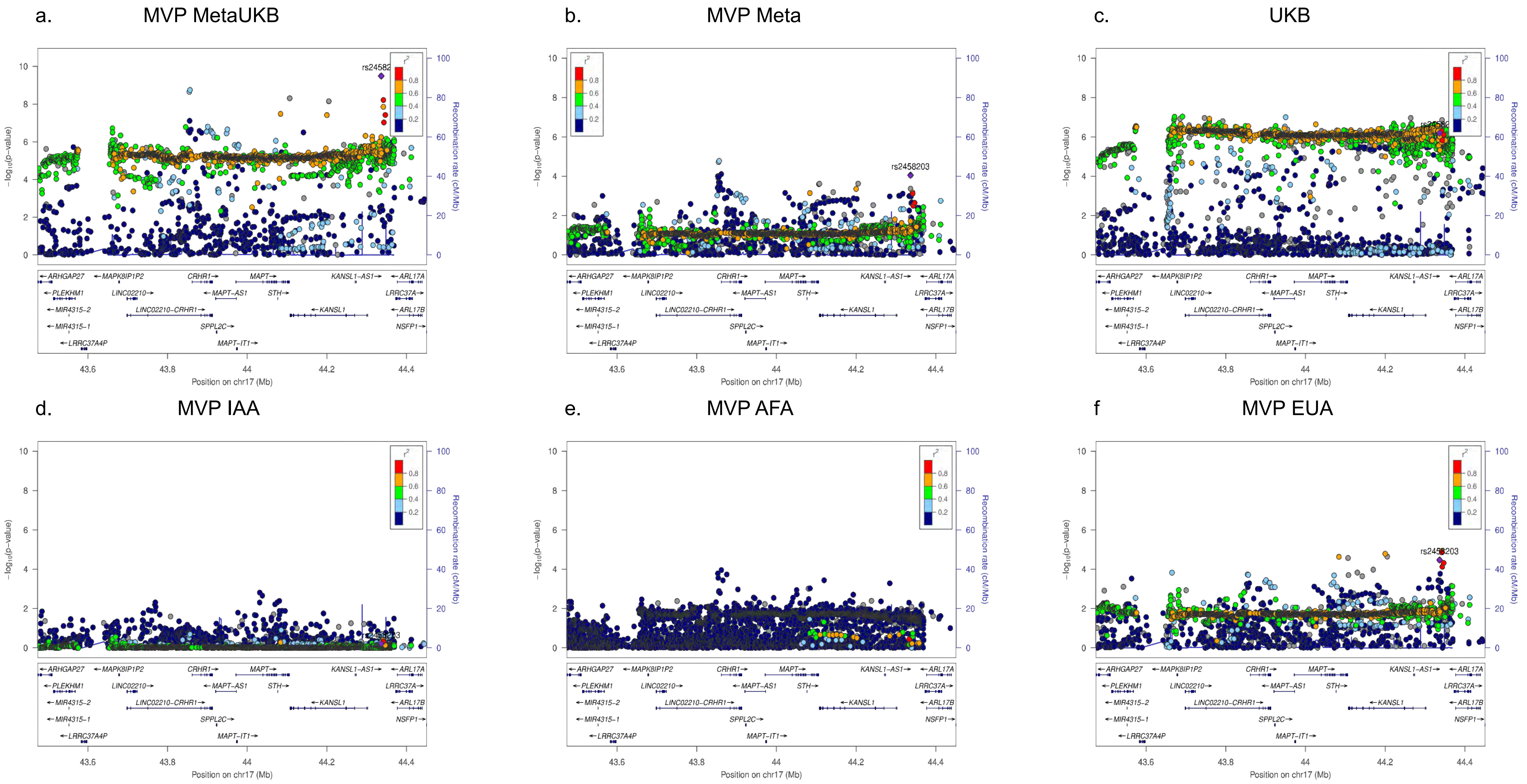

# locus112 | rs75992219

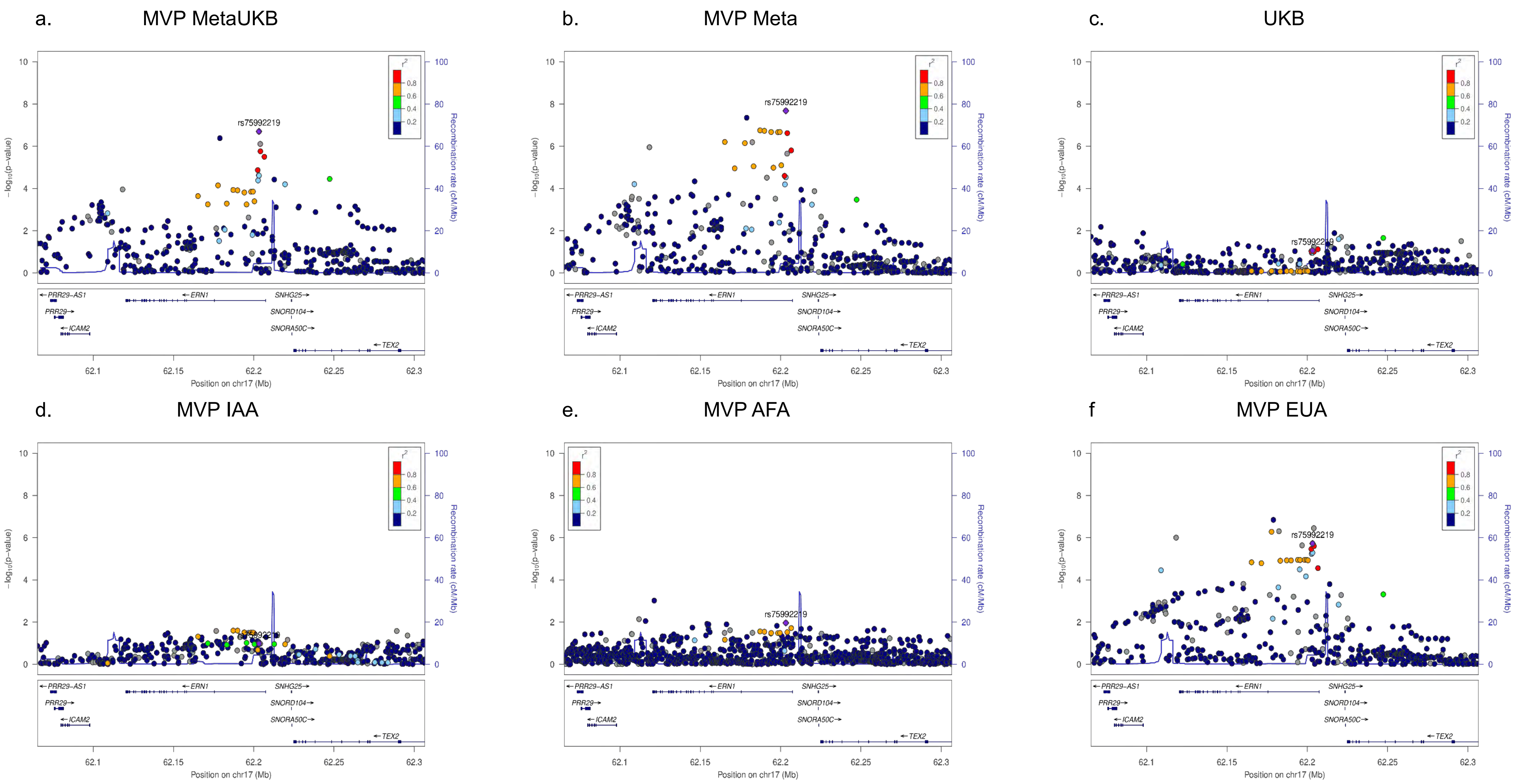

locus113 | rs56305452

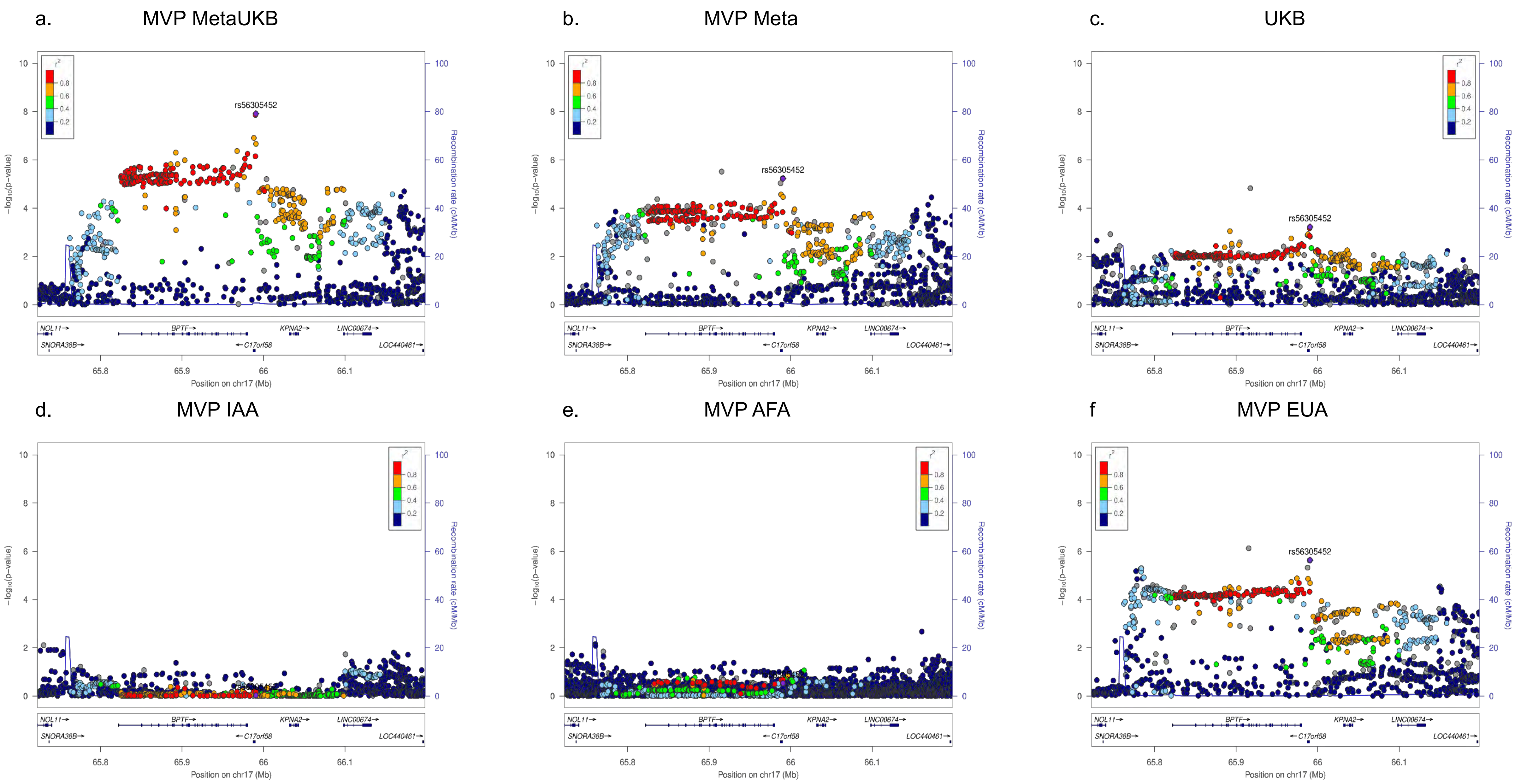

# locus114 | rs58038388

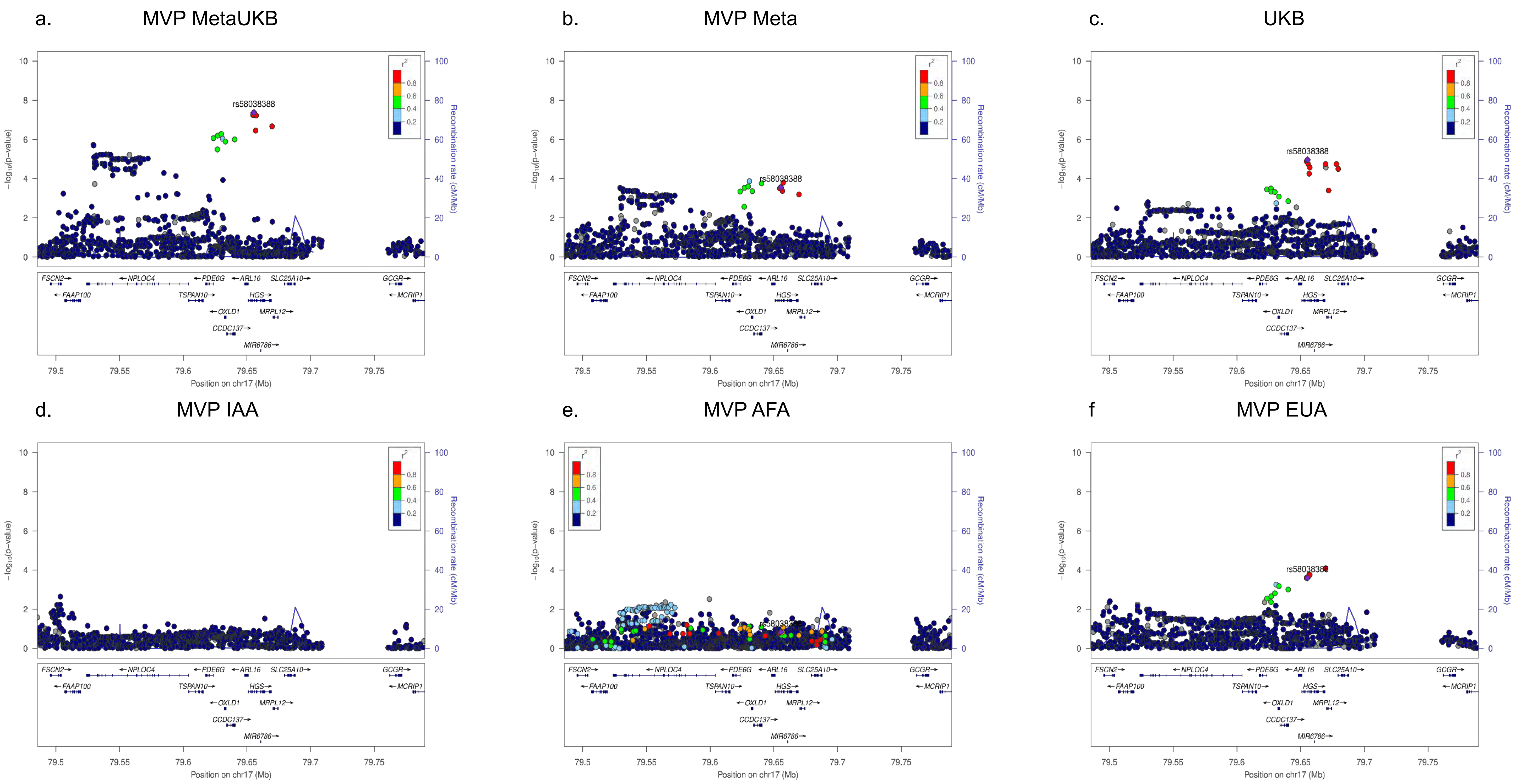

# locus115 | rs2228712

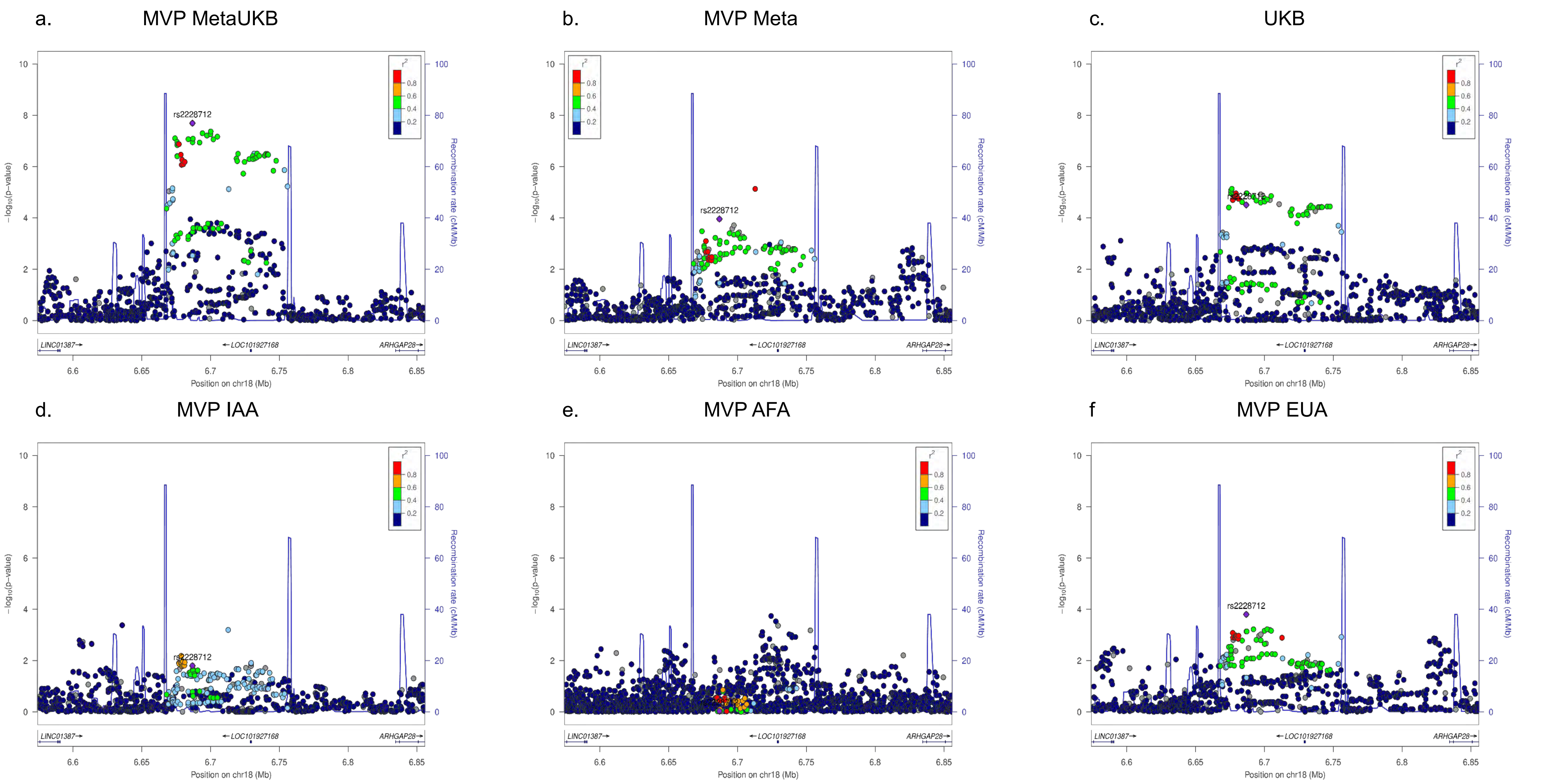

# locus116 | rs2852772

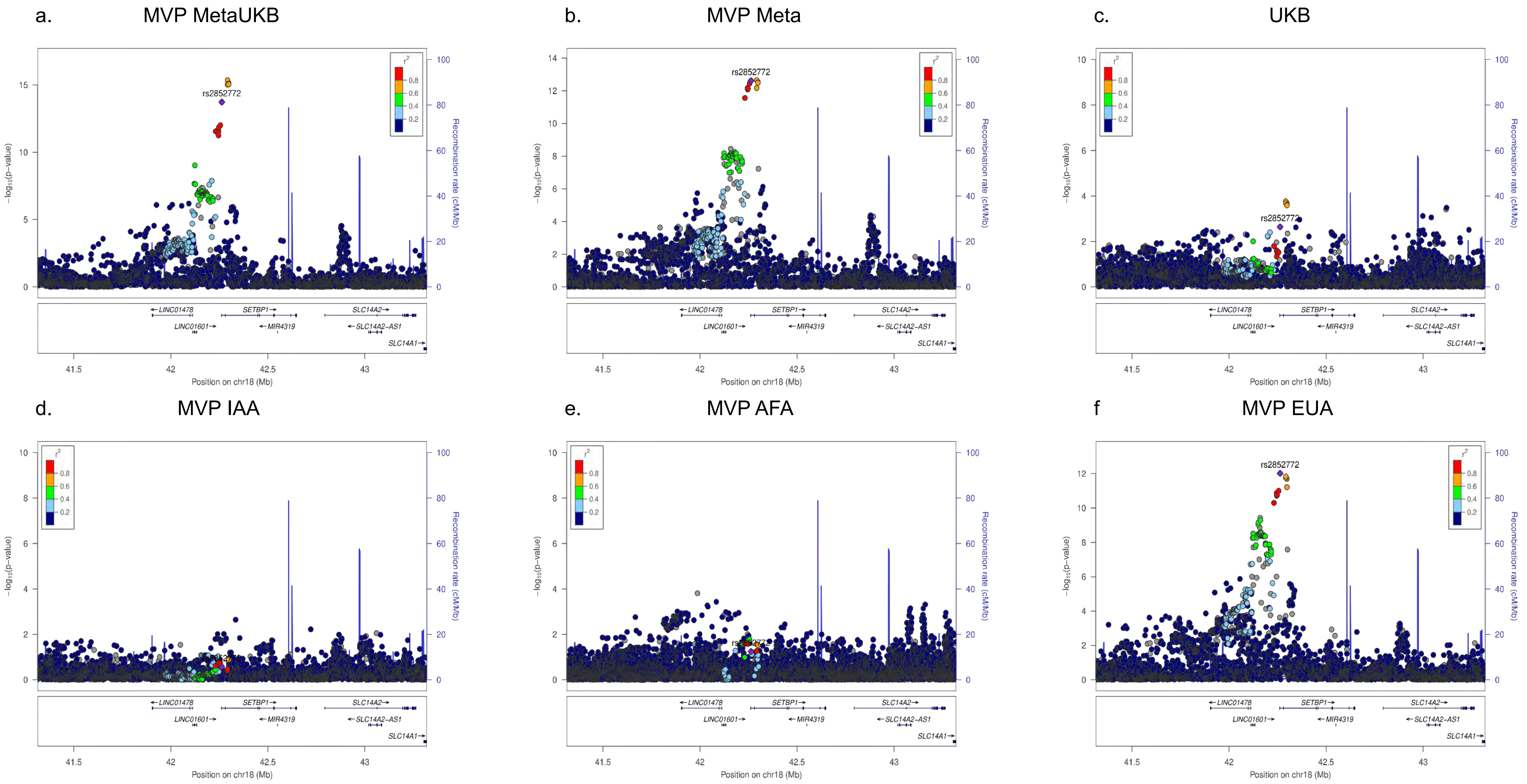

# locus116 | rs2852779

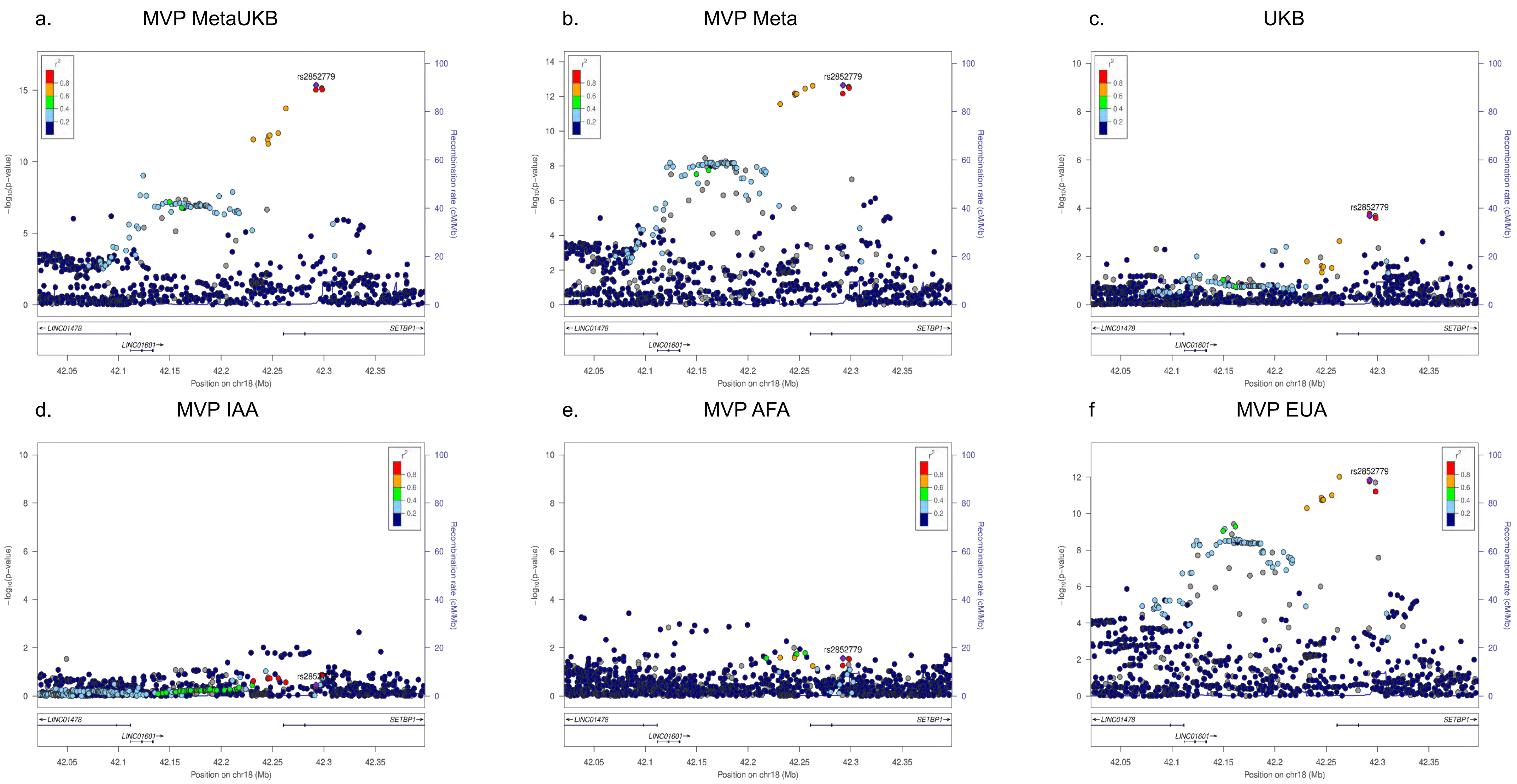

# locus117 | rs11152090

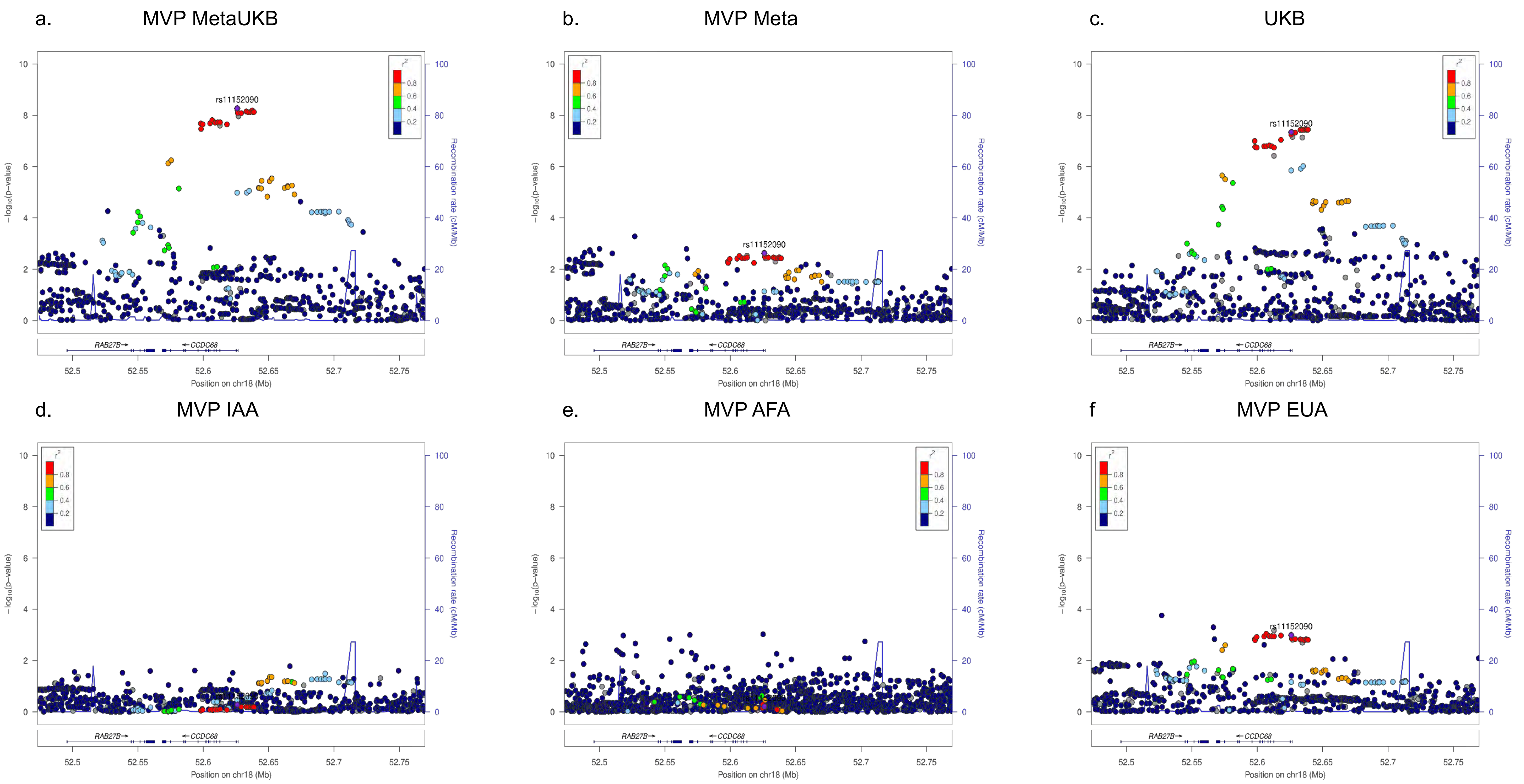

# locus117 | rs72932633

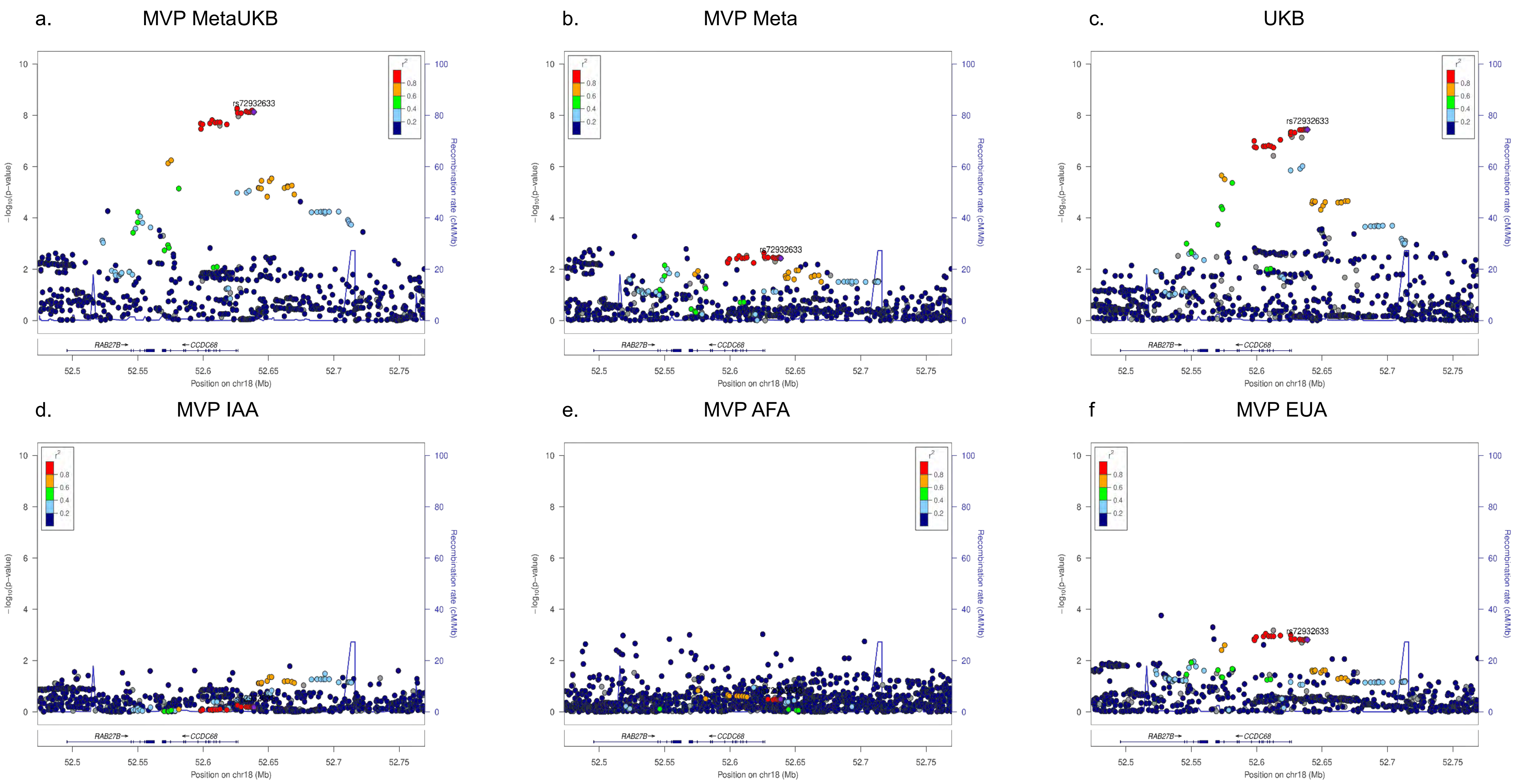

# locus118 | rs1031829

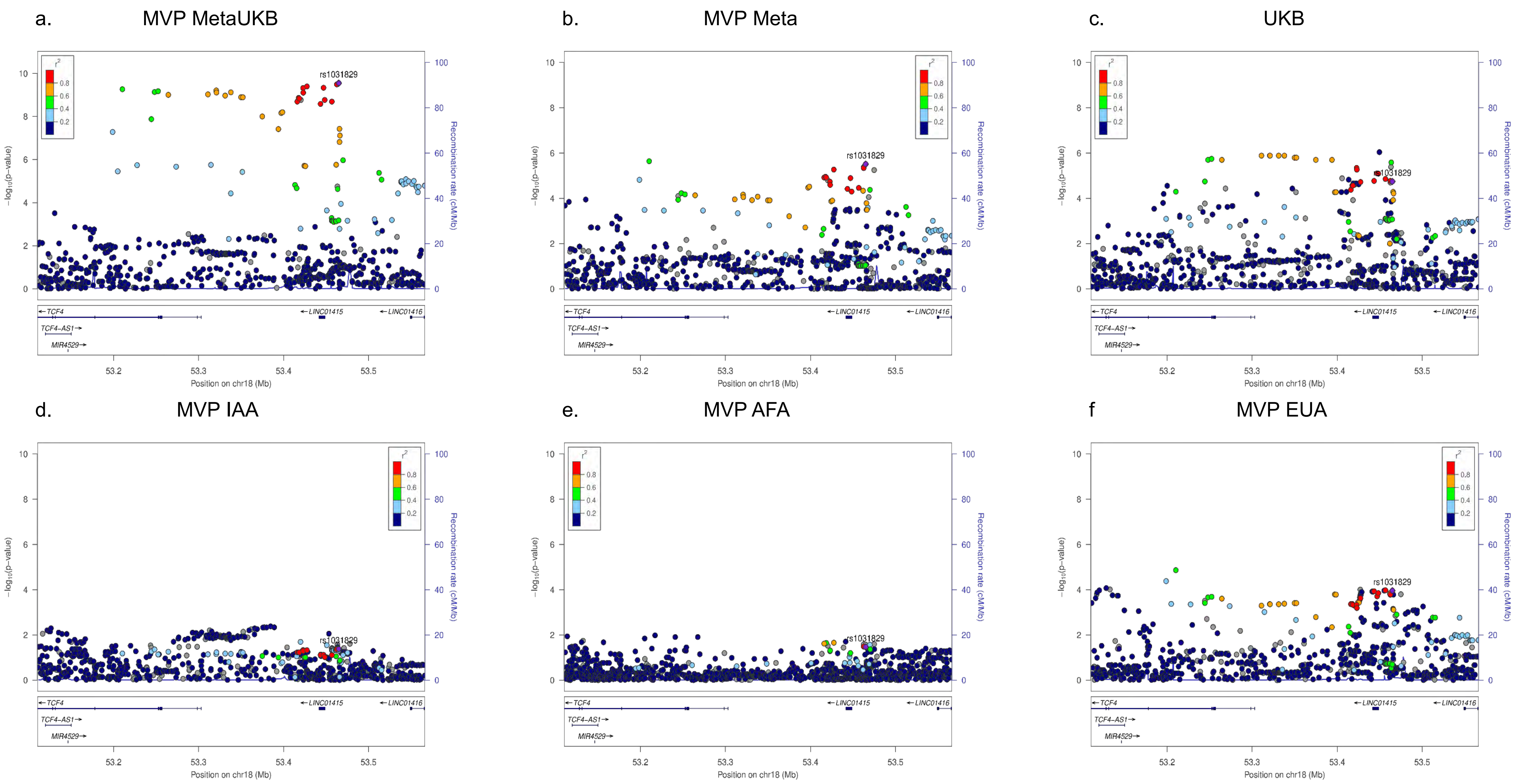

# locus119 | rs10421326

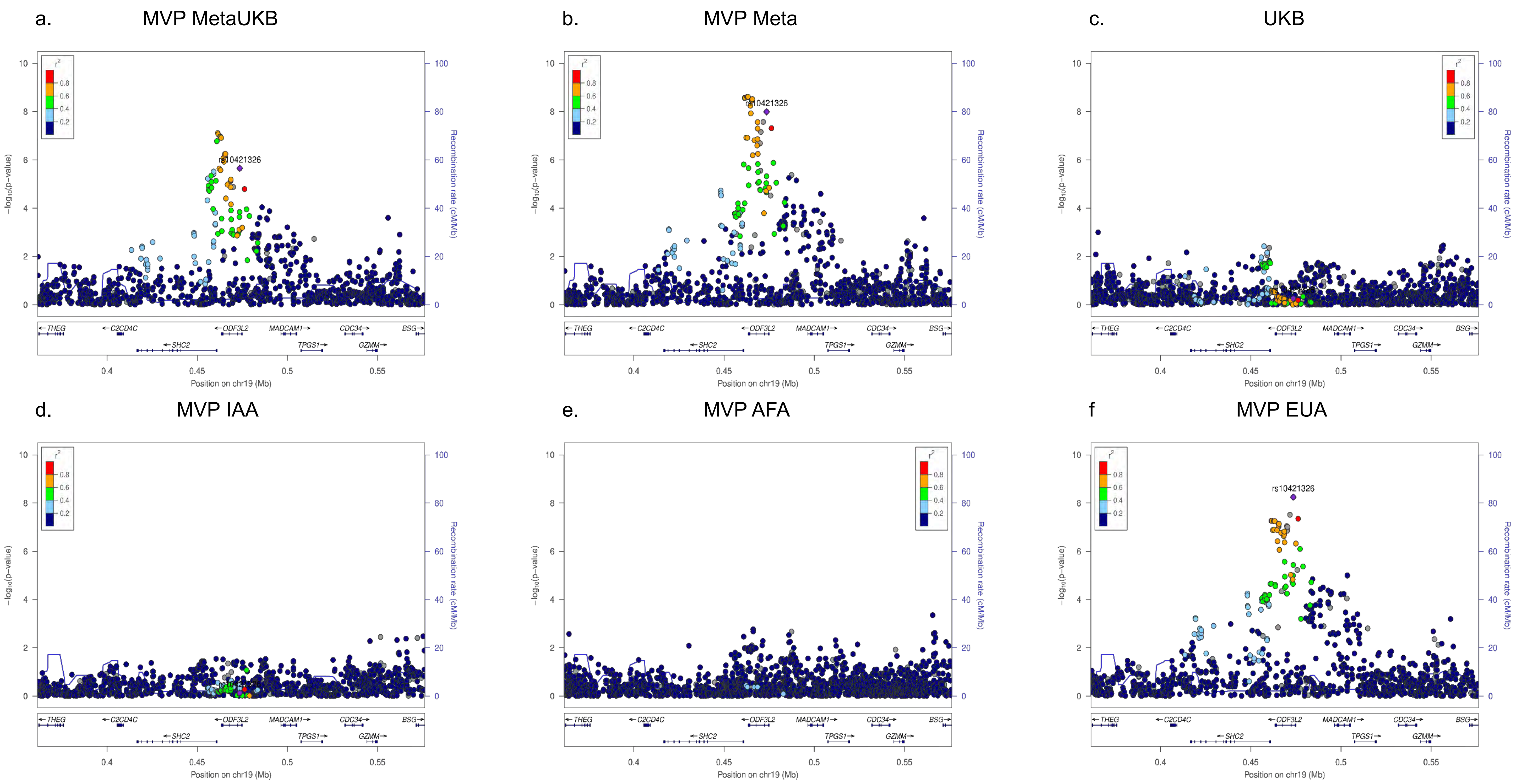

# locus119 | rs1968130

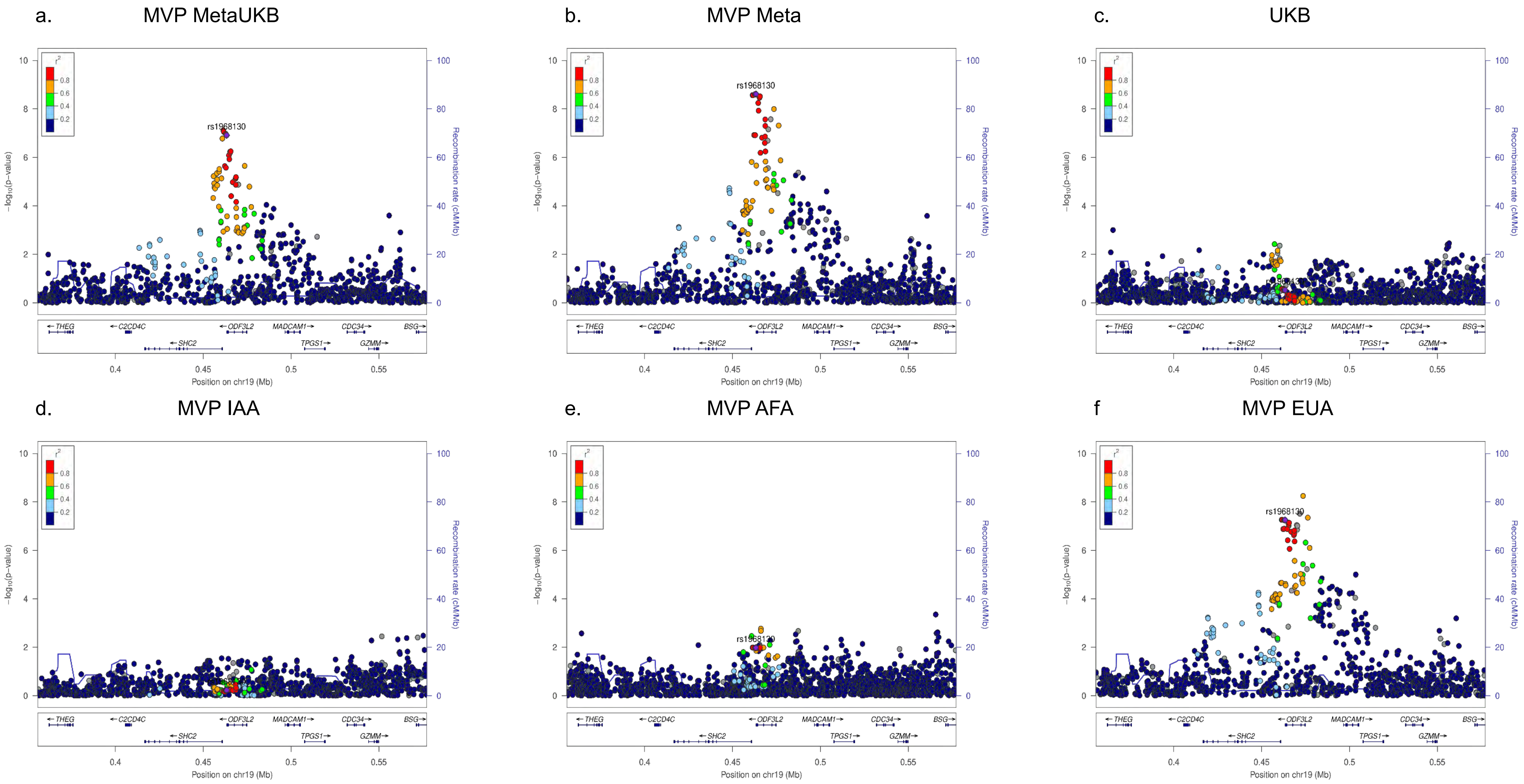

# locus120 | rs6041774

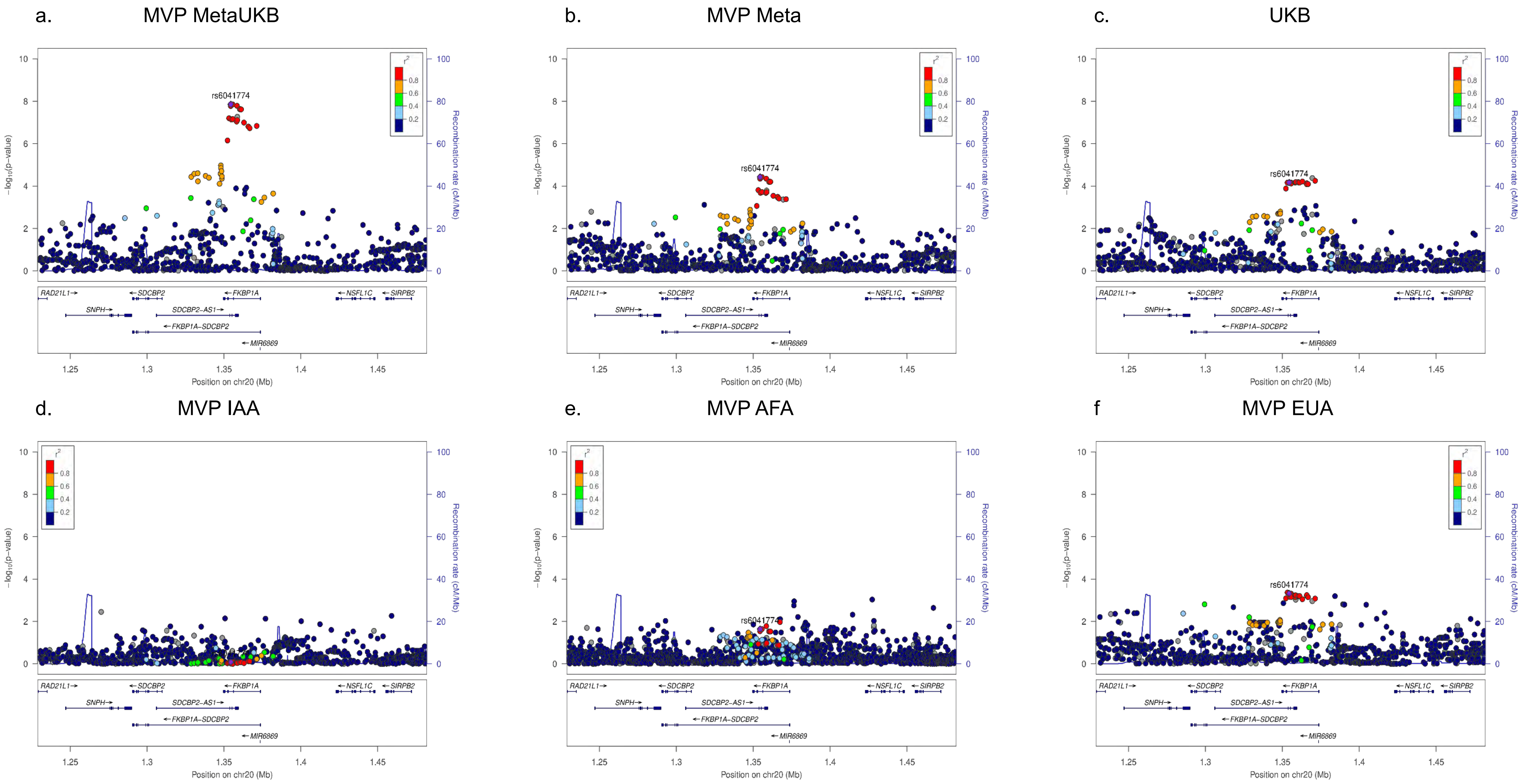

# locus121 | rs146478071

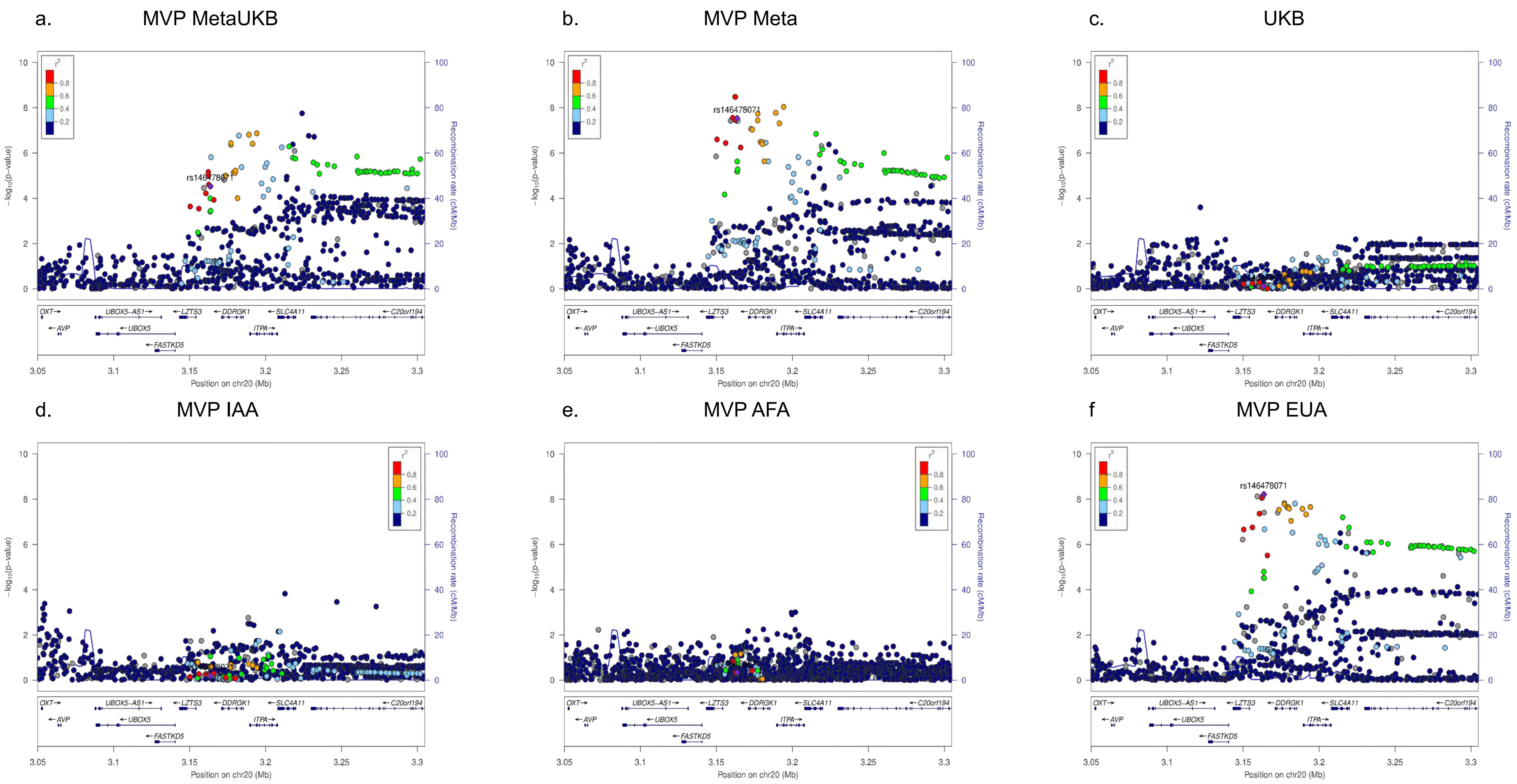

# locus121 | rs6051622

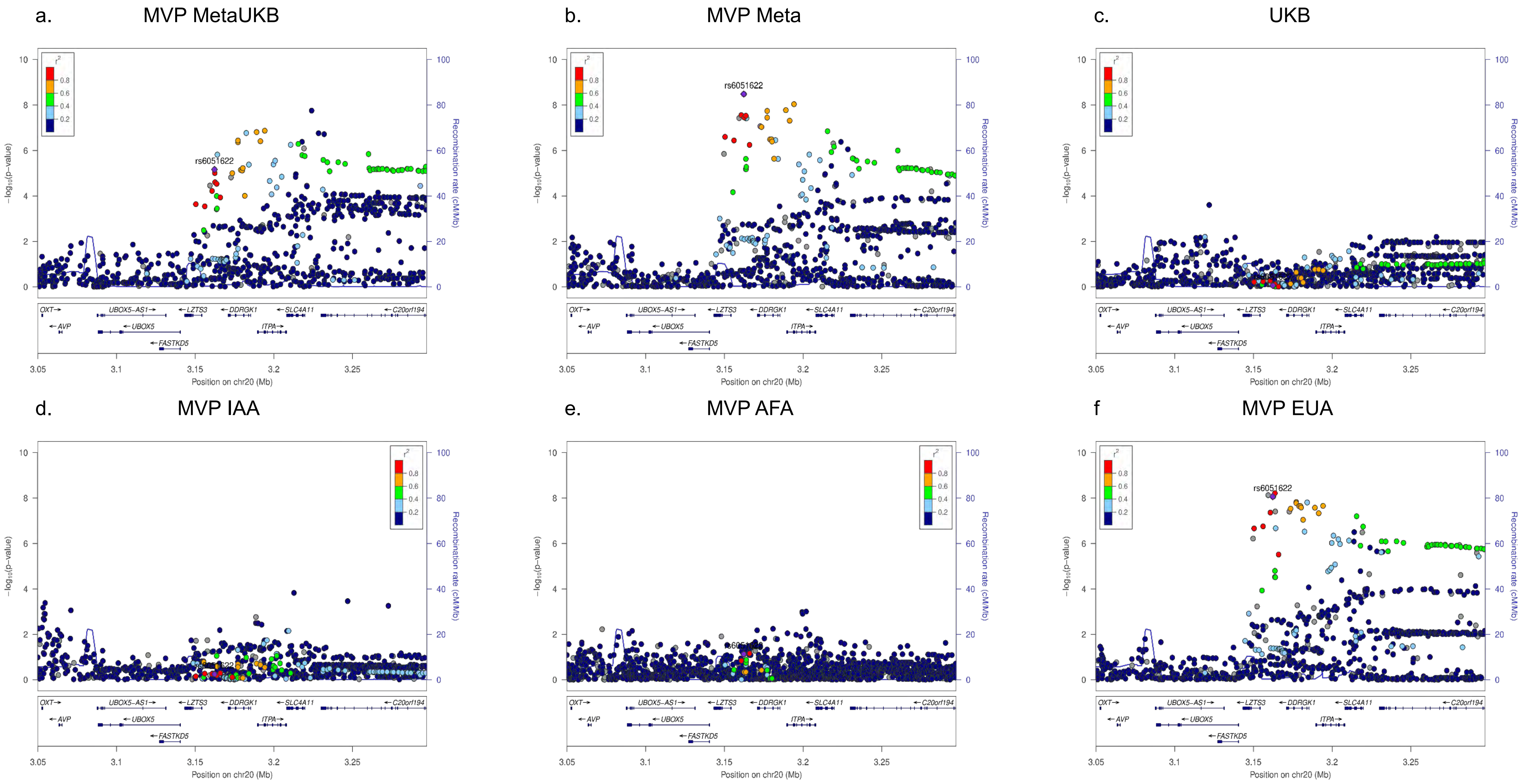

# locus122 | rs6115827

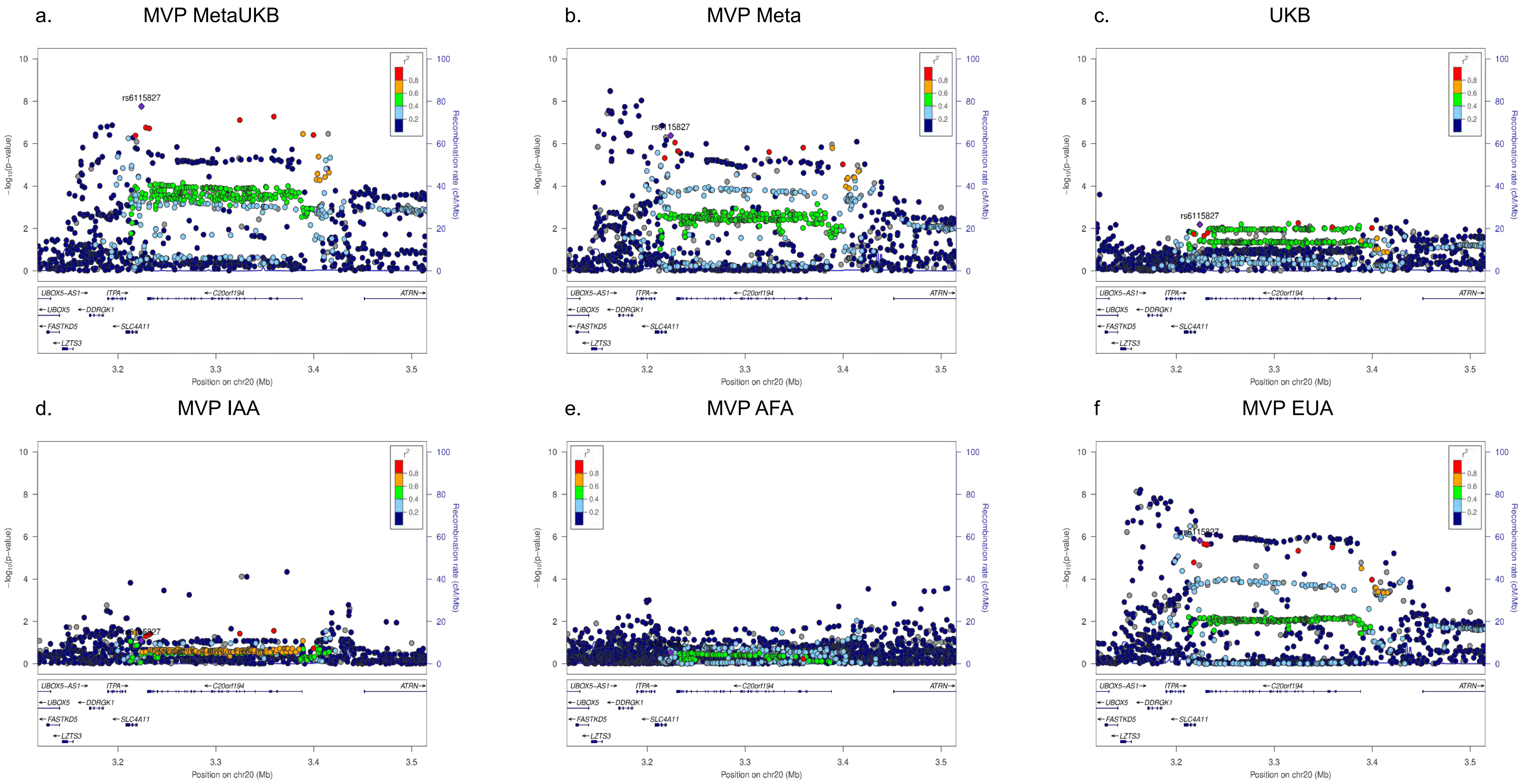

# locus123 | rs61734651

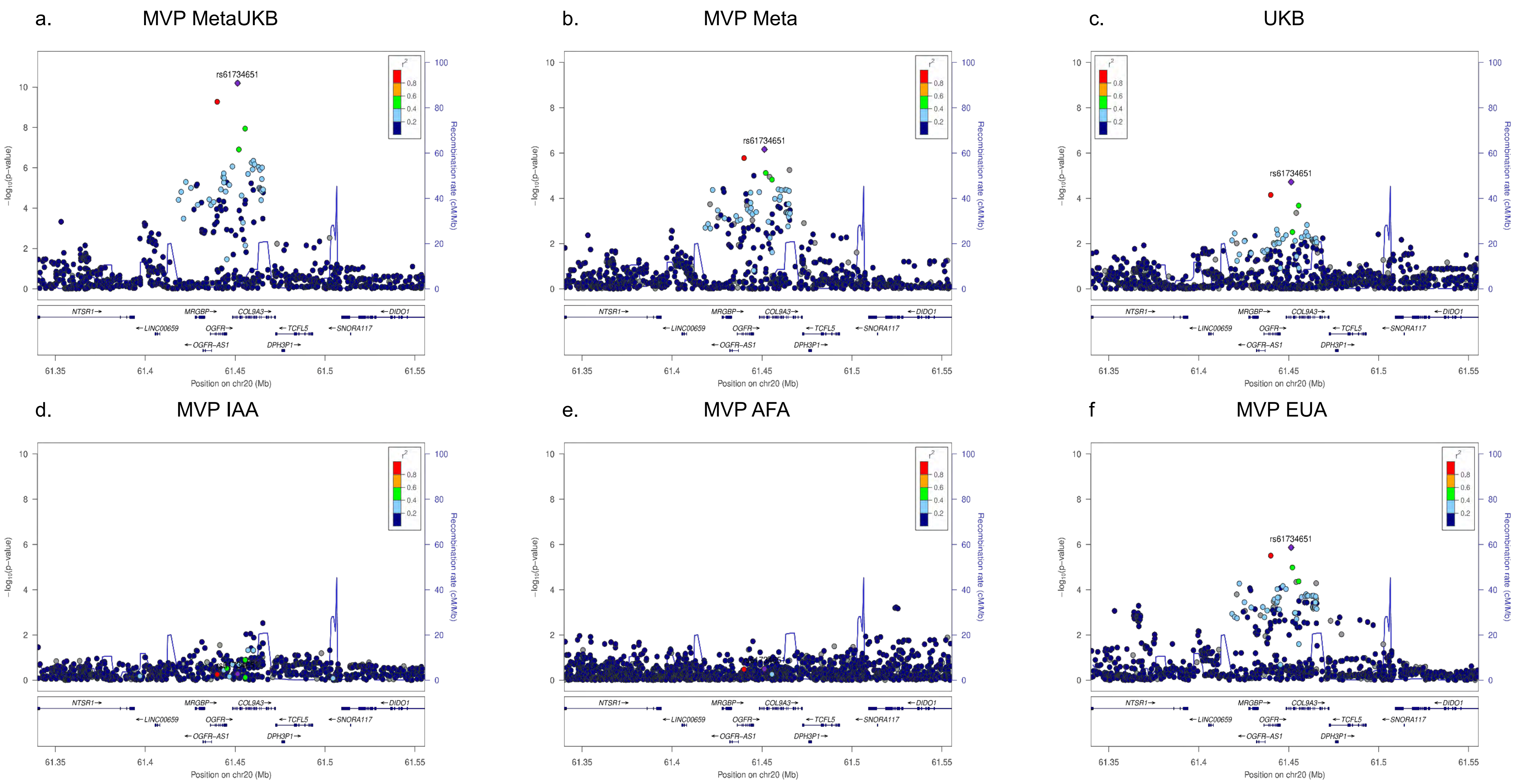

# locus124 | rs45598239

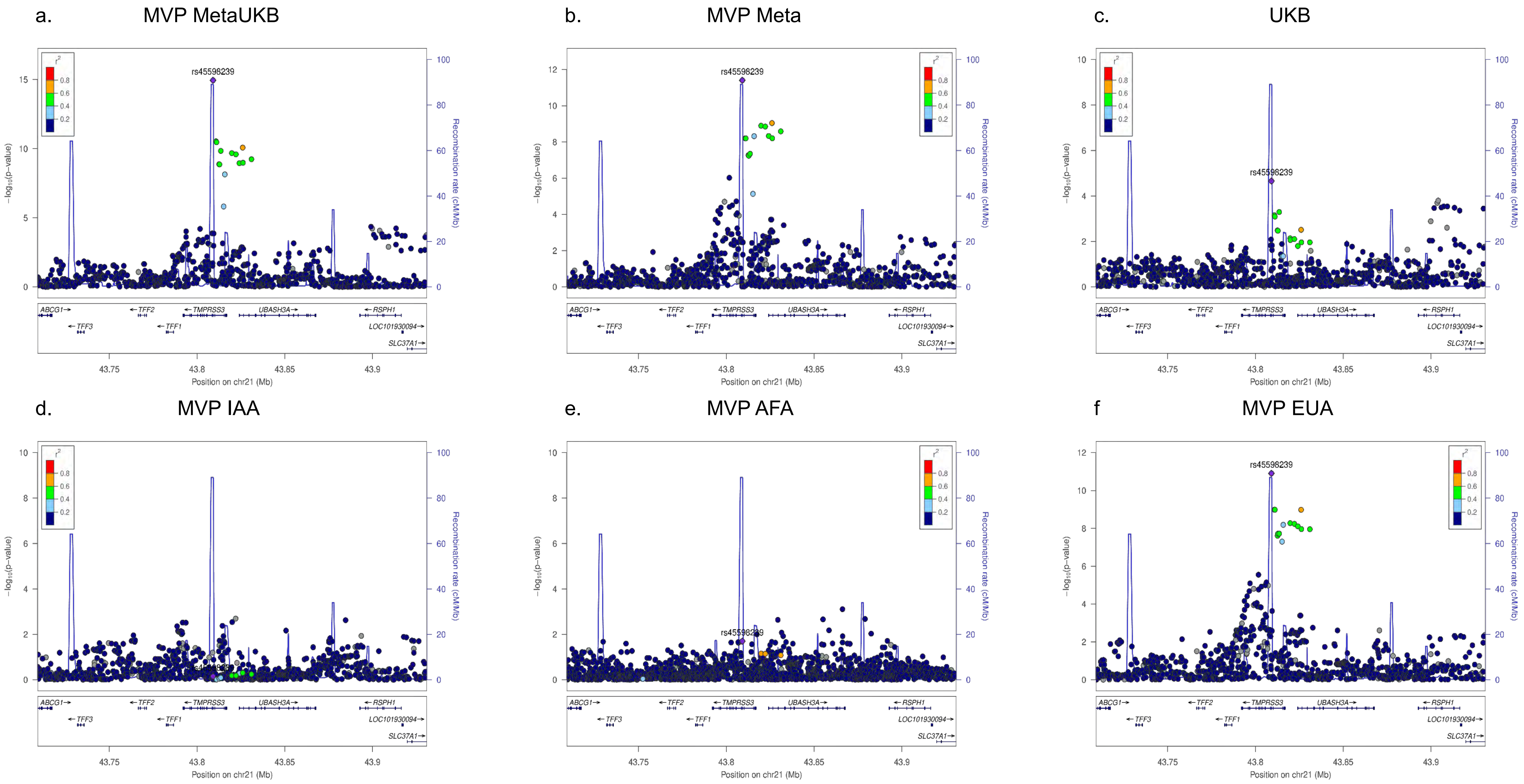

# locus125 | rs132929

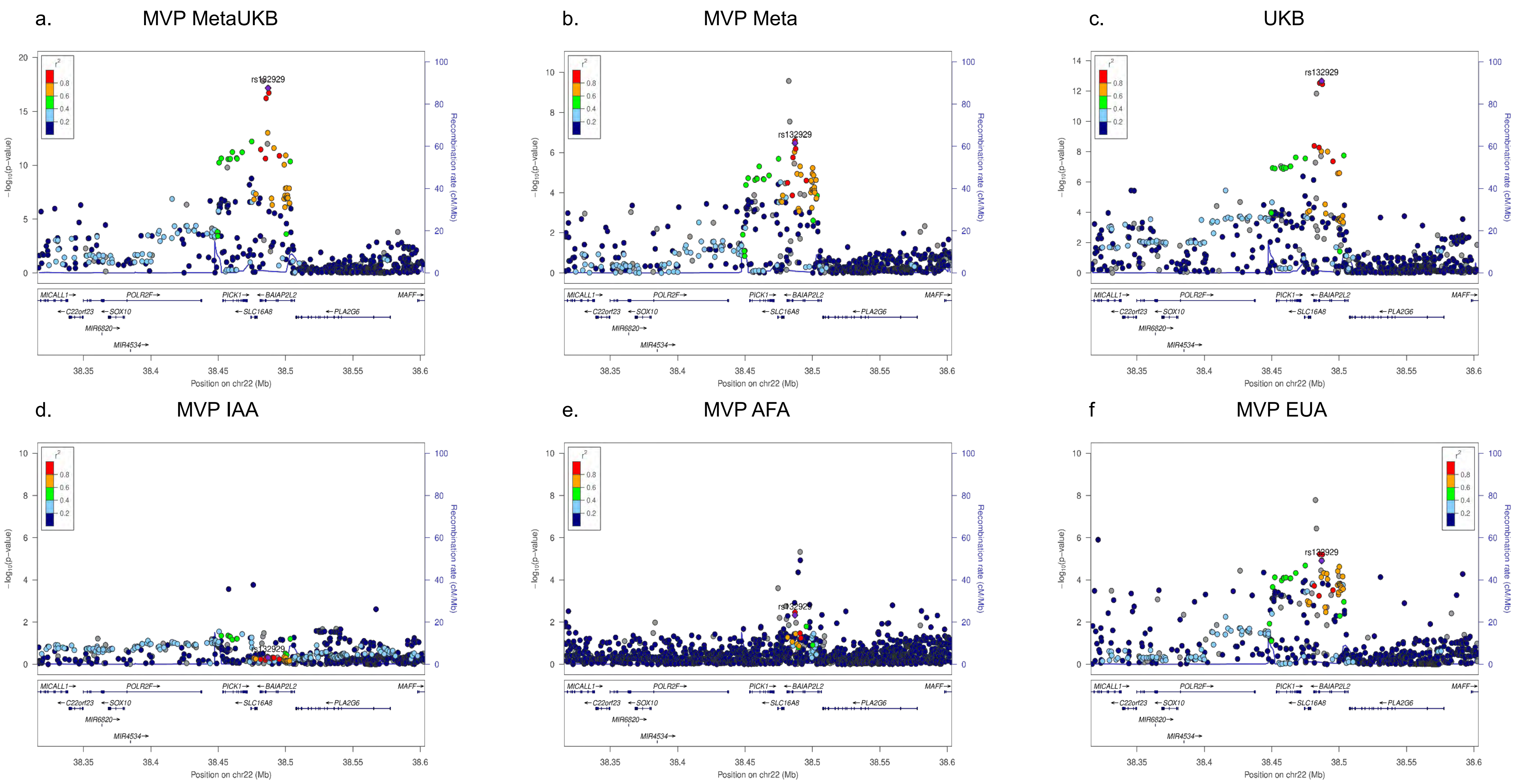

# locus125 | rs5756795

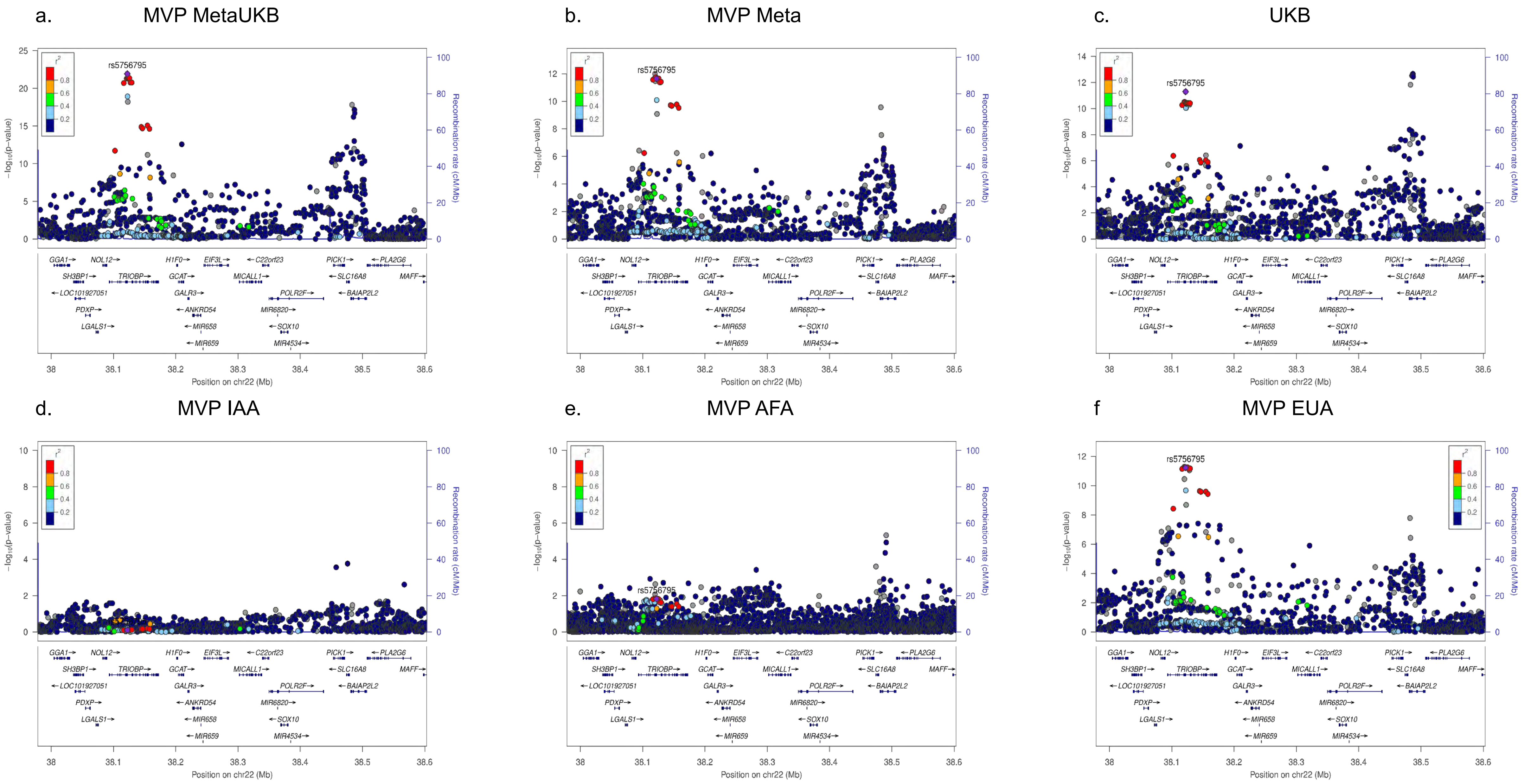

# locus125 | rs66500630

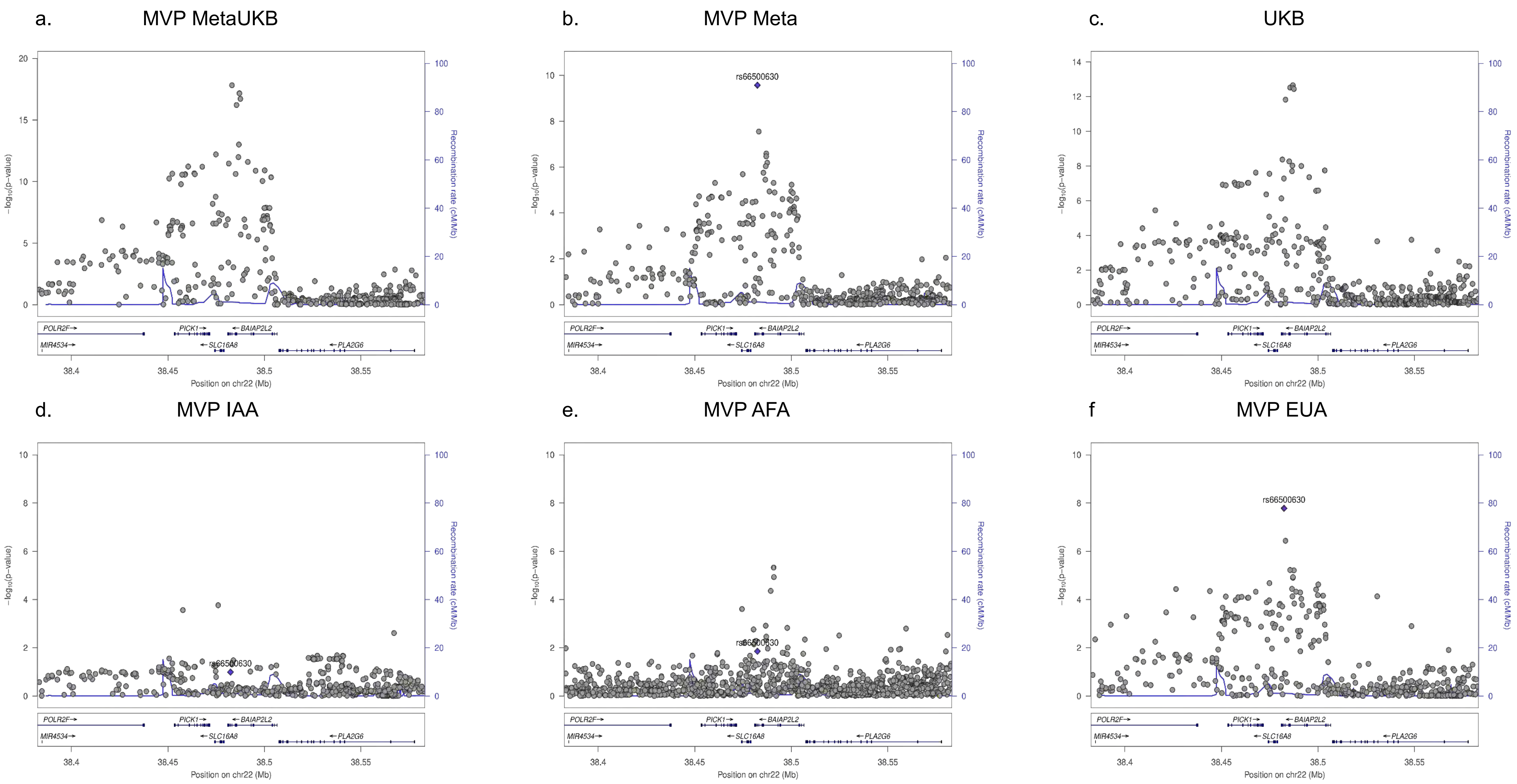

# locus125 | rs67890459

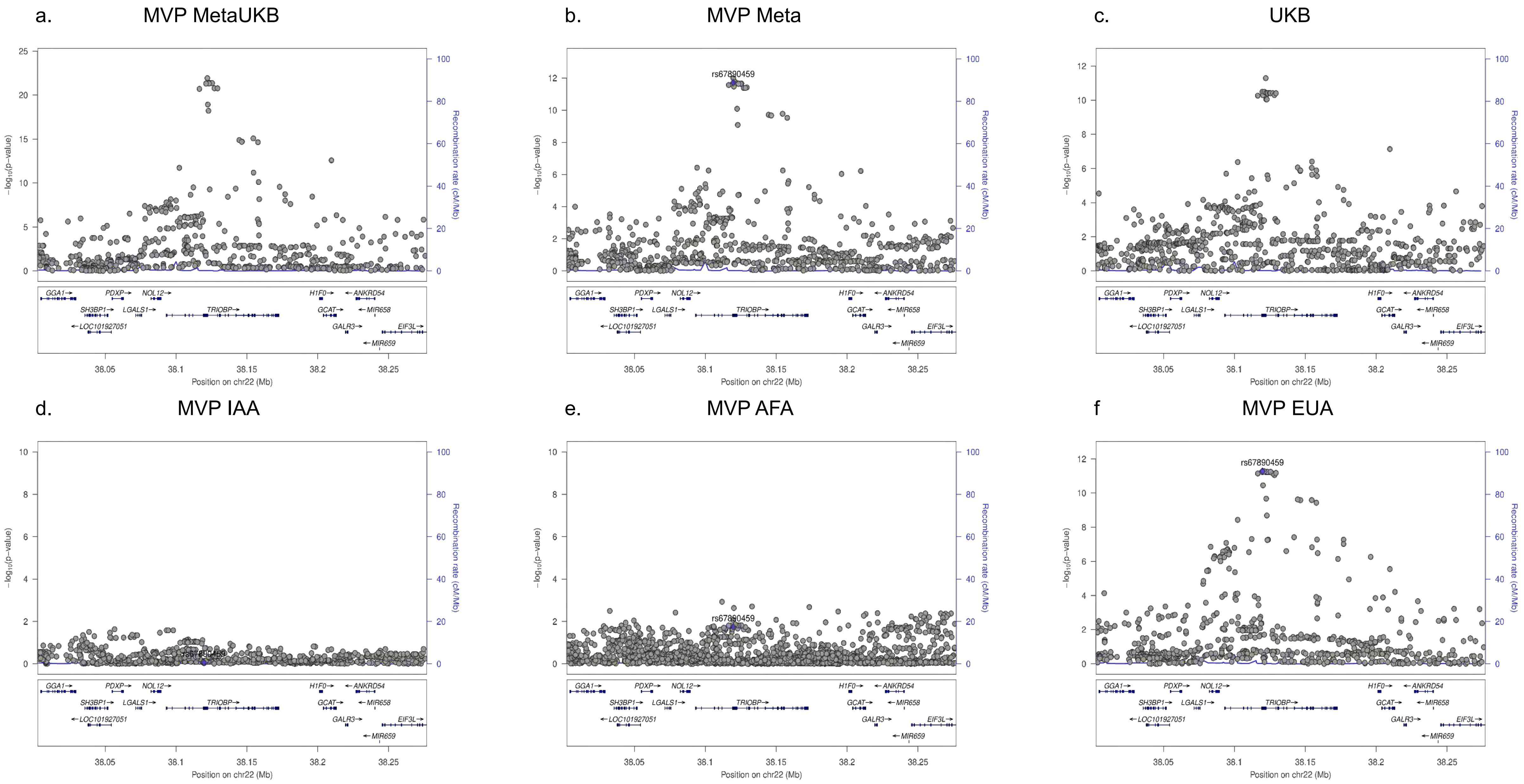

# locus125 | rs9610841

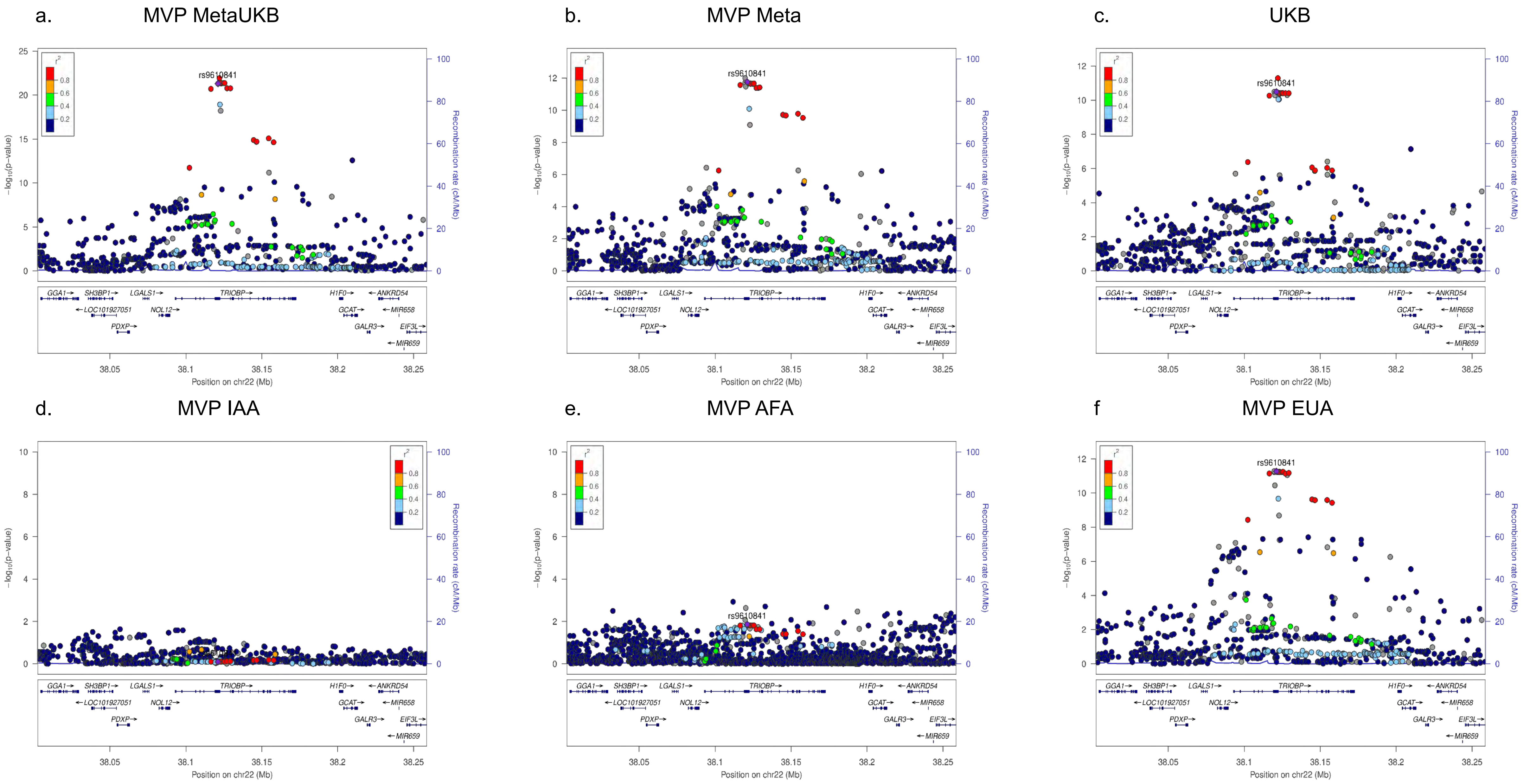

# locus126 | rs36062310

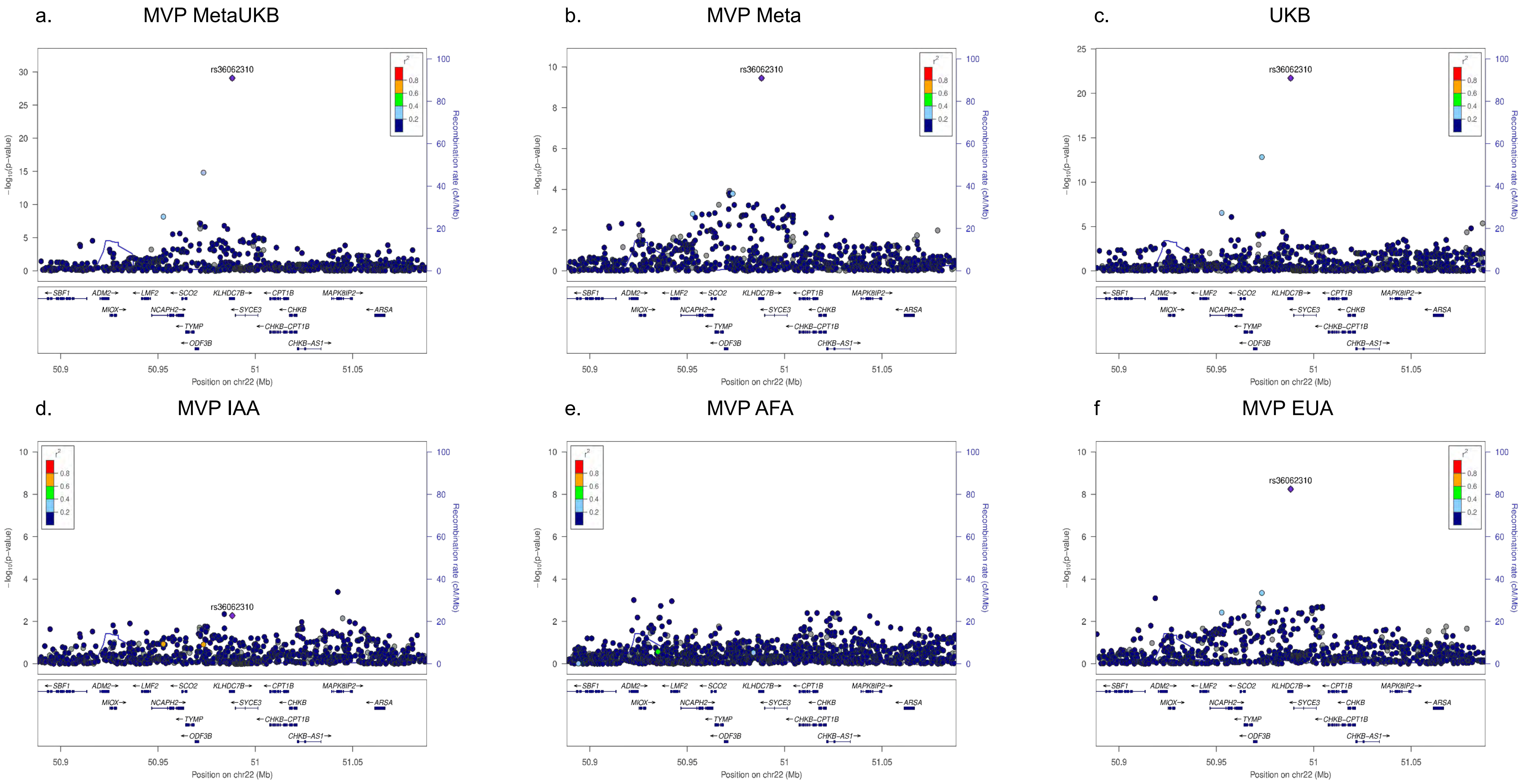

# locus127 | rs9284810

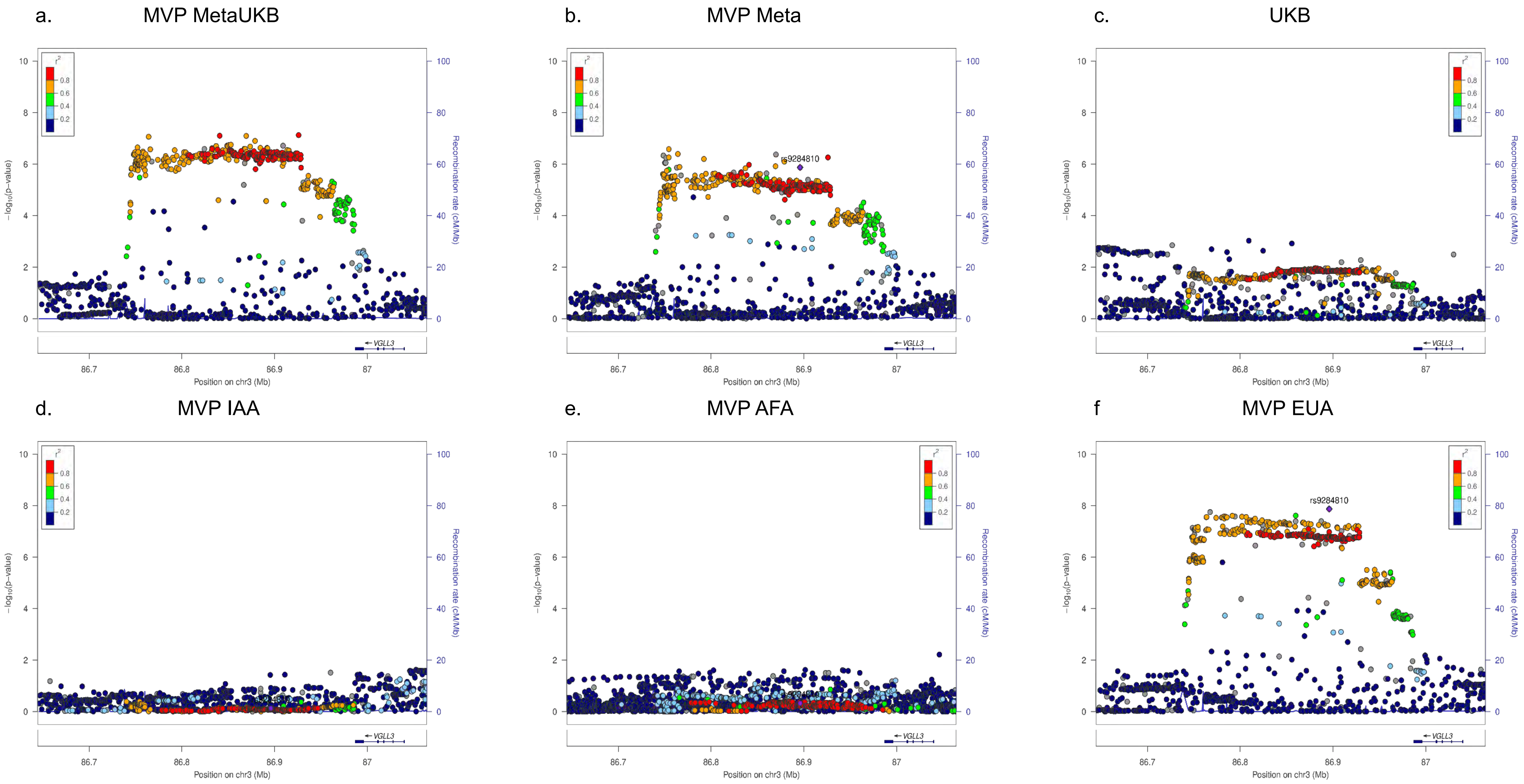

# locus128 | rs11958399

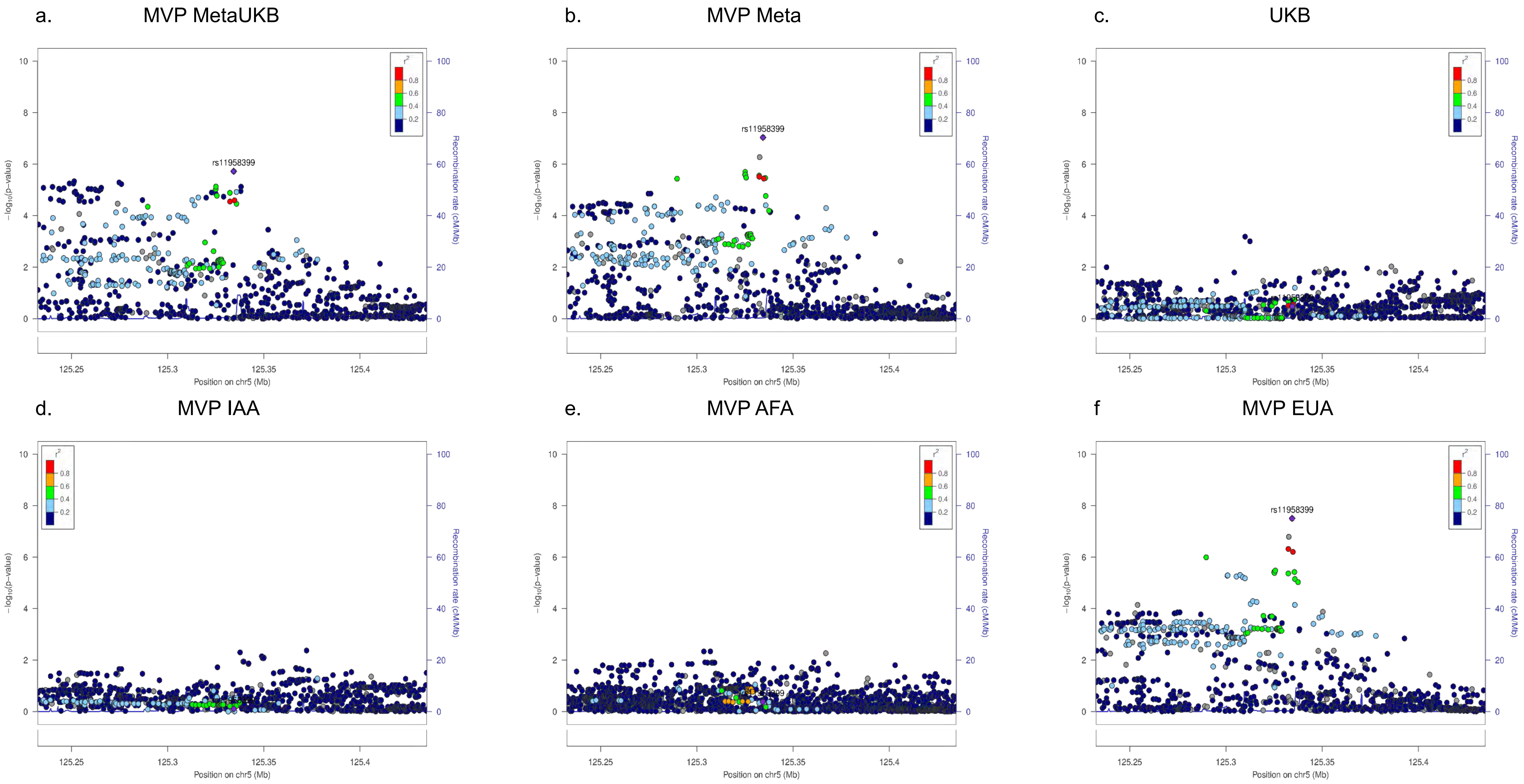

# locus129 | rs6044080

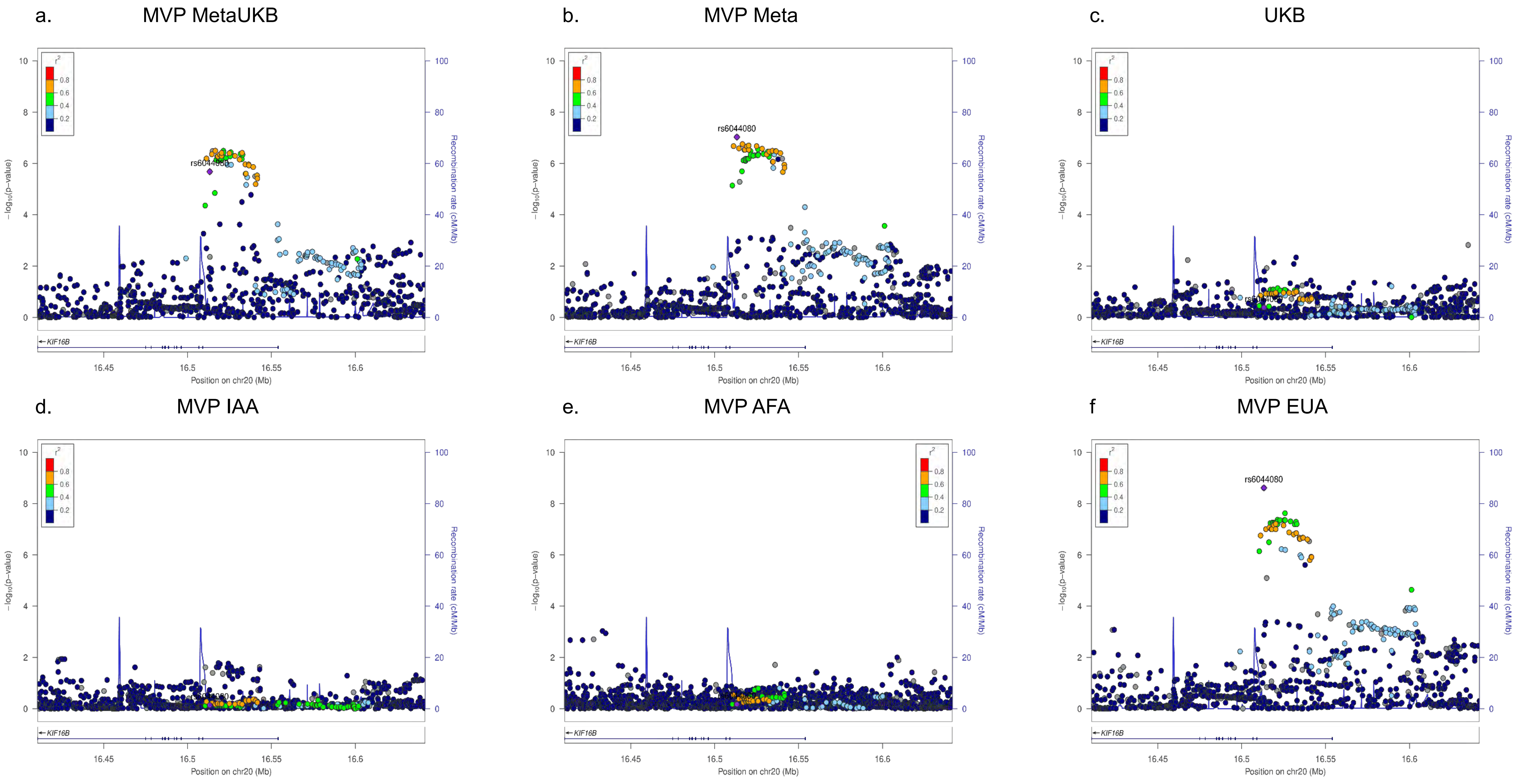

Supplement: Supplementary file 3 — Supplementary file3 (PDF 68.3 MB) [file 10162_2026_1044_MOESM3_ESM.pdf]
